# Development of a novel chemoenzymatic route to enantiomerically enriched β-adrenolytic agents. A case study toward propranolol, alprenolol, pindolol, carazolol, moprolol, and metoprolol†

**DOI:** 10.1039/d2ra04302e

**Published:** 2022-08-10

**Authors:** Paweł Borowiecki, Beata Zdun, Natalia Popow, Magdalena Wiklińska, Tamara Reiter, Wolfgang Kroutil

**Affiliations:** Laboratory of Biocatalysis and Biotransformation, Department of Drugs Technology and Biotechnology, Faculty of Chemistry, Warsaw University of Technology Koszykowa St. 75 00-662 Warsaw Poland pawel.borowiecki@pw.edu.pl; Institute of Chemistry, University of Graz, NAWI Graz, BioTechMed Graz, Field of Excellence BioHealth Heinrichstrasse 28 8010 Graz Austria

## Abstract

Efficient chemoenzymatic routes toward the synthesis of both enantiomers of adrenergic β-blockers were accomplished by identifying a central chiral building block, which was first prepared using lipase-catalyzed kinetic resolution (KR, Amano PS-IM) as the asymmetric step at a five gram-scale (209 mM conc.). The enantiopure (*R*)-chlorohydrin (>99% ee) subsequently obtained was used for the synthesis of a series of model (*R*)-(+)-β-blockers (*i.e.*, propranolol, alprenolol, pindolol, carazolol, moprolol, and metoprolol), which were produced with enantiomeric excess in the range of 96–99.9%. The pharmaceutically relevant (*S*)-counterpart, taking propranolol as a model, was synthesized in excellent enantiomeric purity (99% ee) *via* acetolysis of the respective enantiomerically pure (*R*)-mesylate by using cesium acetate and a catalytic amount of 18-Crown-6, followed by acidic hydrolysis of the formed (*S*)-acetate. Alternatively, asymmetric reduction of a prochiral ketone, namely 2-(3-chloro-2-oxopropyl)-1*H*-isoindole-1,3(2*H*)-dione, was performed using lyophilized *E. coli* cells harboring overexpressed recombinant alcohol dehydrogenase from *Lactobacillus kefir* (*E. coli*/Lk-ADH-Lica) giving the corresponding chlorohydrin with >99% ee. Setting the stereocenter early in the synthesis and performing a 4-step reaction sequence in a ‘one-pot two-step’ procedure allowed the design of a ‘step-economic’ route with a potential dramatic improvement in process efficiency. The synthetic method can serve for the preparation of a broad scope of enantiomerically enriched β-blockers, the chemical structures of which rely on the common α-hydroxy-*N*-isopropylamine moiety, and in this sense, might be industrially attractive.

## Abstract 2

Efficient chemoenzymatic routes toward both enantiomers of β-blockers were accomplished by identifying a central chiral building block prepared using either lipase-catalyzed kinetic resolution methodology or ADH-catalyzed biotranshydrogenation.

## Graphical abstract

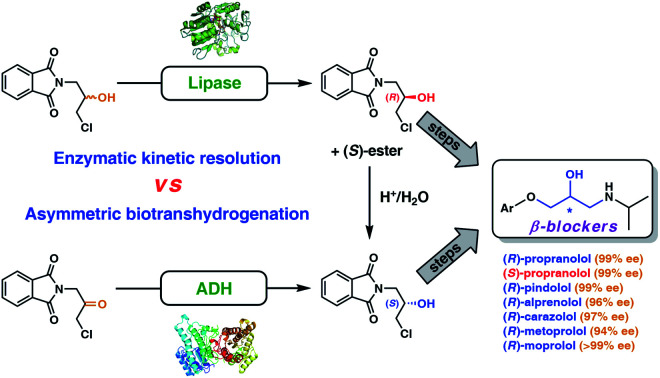

## Introduction

**Fig. 1:**
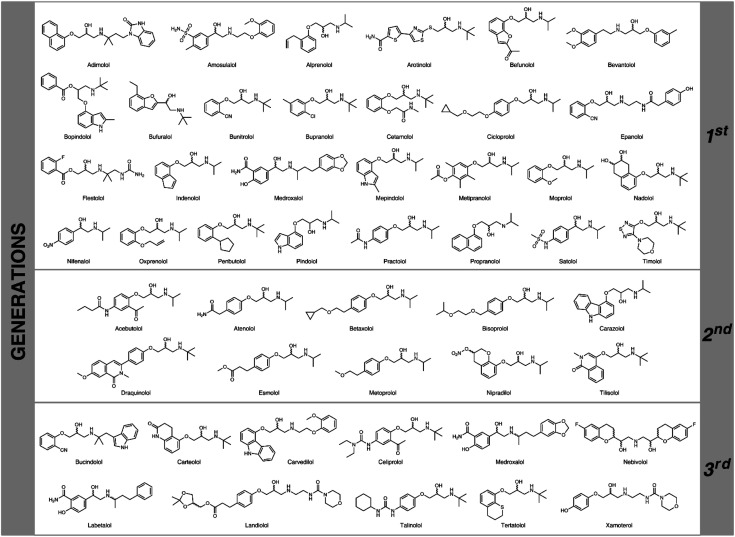
The representative examples of three generations of β-blocking agents.

Cardiovascular diseases (CVD) are a major global health issue as they occupy the first place (before cancers and chronic respiratory diseases) on the list of illnesses that are responsible for mortality in the human population (17.9 million deaths worldwide in 2017) according to ‘World Health Statistics 2020’ WHO report.^1^ Due to this fatal fact, adrenoreceptor blocking agents (also known as β-adrenolytic/β-adrenergic agents/antagonists), commonly termed β-blockers, are the active pharmaceutical ingredients of one of the largest best-selling groups of drugs commercially marketed these days for the management and treatment of CVD (Fig. 1). β-Adrenolytic drugs are used in both monotherapy and combined therapy of various CVD, including hypertension, myocardial infarction, cardiac arrhythmias, cardiomyopathy, angina pectoris, *etc.*^2^ Their mechanism of action includes competitive blocking of β-adrenergic receptors for the endogenous catecholamines (*i.e.*, adrenaline, noradrenaline, *etc.*), which prevents chronotropic, inotropic, and vasoconstrictor responses *in vivo*.^3^ Moreover, since interactions of β-blockers with β-adrenoreceptors are highly stereoselective, it is crucial to synthesize these compounds at the highest possible level of optical purity. In general, the primary biological activity of β-blocking agents resides mainly in the (*S*)-alcohol enantiomers, whereas the (*R*)-counterparts are several orders of magnitude less active and may cause potentially serious adverse effects.^4^

Undoubtedly, the most crucial chiral building blocks for the synthesis of non-racemic β-blockers are epihalohydrins and the related halo-1,2-propanediols^5^ as well as glycidol and the corresponding 2,3-epoxy alcohols or solketal derivatives.^6^ Until now, a plethora of chemo- and bio-catalytic synthetic efforts have been performed toward these versatile intermediates. However, a major drawback in the employment of common epihalohydrins or glycidyl esters in the preparation of β-blockers is the poor regioselectivity of the reactions performed with nucleophilic reagents. It is worth mentioning that among the most successful biocatalytic attempts are the lipase-catalyzed kinetic resolutions (KRs) of the respective racemates. In the last two decades, numerous highly efficient lipase-supported methods towards common non-racemic β-blockers, such as acebutolol,^7^ alprenolol,^8^ atenolol,^9^ bufuralol,^10^ bunitrolol,^11^ carteolol,^12^ carvedilol,^13^ cloranolol,^14^ esmolol,^15^ labetalol,^16^ metoprolol,^17^ moprolol,^18^ nifenalol,^19^ pindolol,^20^ practolol,^21^ propranolol,^22^ and satolol^22*b*^ were elaborated. Despite these creative efforts invested in the biocatalytic syntheses of non-racemic β-blockers, the reported methods lack generality since any selected API of this class requires an independent and strictly dedicated synthetic strategy. In general, most lipase-catalyzed attempts employ kinetic resolution of the respective racemic 1-aryloxy-3-chloro-2-propanols. Therefore, to obtain the appropriate chiral β-blocker, different intermediate needs to be used each time, and different reaction conditions (including diverse enzymes) have to be tailored/optimized toward a particular intermediate. Such an approach is rather time-consuming and unprofitable from an industrial point of view. Since no (bio)catalyst can ever be “universal”, organic chemists should seek to develop non-racemic intermediates useful for a range of different products and make the asymmetric induction or chiral resolution at early stages of the planned syntheses according to ‘the golden rule of chirotechnology’.^23^ This is especially true in the case of β-adrenolytic drugs, which structural core is to a great extent conservative.

In this study, our ultimate goal was to provide a practical chemoenzymatic synthesis for the preparation of enantiomerically enriched β-blockers using a single non-racemic key precursor obtained *via* biocatalytic methods. Although the desired pharmacological effects of β-blockers *in vivo* are due to their (*S*)-enantiomers, it is pivotal to access both stereoisomers of many of these APIs as well. This is due to an unrelenting interest in establishing a complete characterization of the separated pharmacological and pharmacokinetic profiles of enantiomers of β-blockers as well as their combinations before performing definitive commercial “chiral switching” of these drugs in the future. Noteworthy, the synthesis of (*R*)-β-blockers is also crucial because they exhibit relatively strong activity in blocking β_2_ receptors in ciliary processes, thus lowering intraocular pressure in patients affected with primary open-angle glaucoma and ocular hypertension.^5^ In this regard, the use of the lipase-catalyzed kinetic resolution of the appropriately selected racemic hydroxyl precursor and/or bioreduction of the respective prochiral carbonyl derivative mediated by stereocomplementary alcohol dehydrogenases (ADHs) were envisioned to be excellent tools for introducing the desired chirality and obtaining both enantiomers of title APIs.

## Results and discussion

In this work, we opt to design a general applicable synthetic route toward non-racemic β-blockers that would use readily available, inexpensive starting materials and biocatalysts as well as consist of a minimized number of steps prone to scalability. Moreover, our principal desire was to employ a chiral compound, which preparation in optically pure form (*via* resolution of its enantiomers or stereoselective bioreduction of the corresponding ketone) at the early stages of the synthesis could provide a common intermediate for all title APIs.

**Fig. 2:**
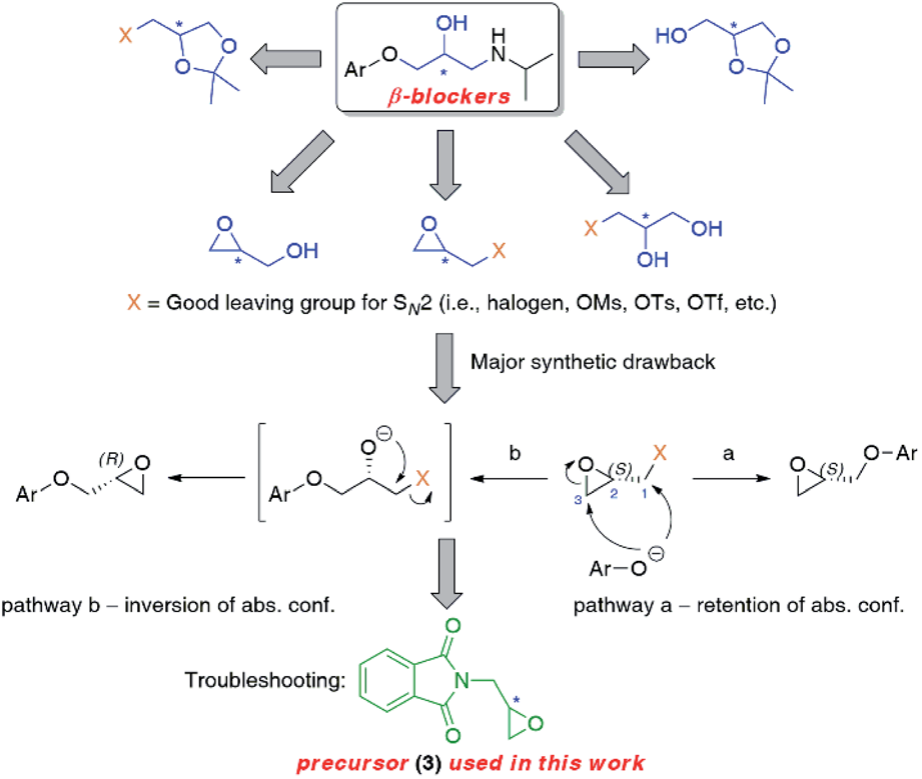
Retrosynthetic options towards non-racemic β-blockers possessing isopropylamine moiety based on glycidyl phthalimide (3) as a universal chiral intermediate.

Inspired by a review article on the synthetic approaches of rivaroxaban published by T. A. Fattah and A. Saeed,^24^ we envisioned that 2-[(2*S*)-oxiran-2-ylmethyl]-1*H*-isoindole-1,3(2*H*)-dione [also known as (*S*)-glycidyl phthalimide, (*S*)-(+)-3] would be a perfect building block not only for the reported therein anticoagulant drug but also for β-adrenolytic agents possessing an amino-alkanol side chain with characteristic terminal isopropylamine moiety (almost half of known β-blockers) (Fig. 2). Since the 1980s, glycidyl phthalimide (3) has been considered a valuable precursor for cardiac medicines of the aryloxypropanolamine type (2-propanoamino-1-arylethanols). Nevertheless, to the best of our knowledge and great surprise, there are no reports relating to the preparation of either optically active or even racemic β-blockers from that particular intermediate 3. Last but not least, enantiomerically enriched carazolol (8d) has never been synthesized using an enzymatic approach, and thus it seemed reasonable to fill both these gaps simultaneously.

**Scheme 1:**
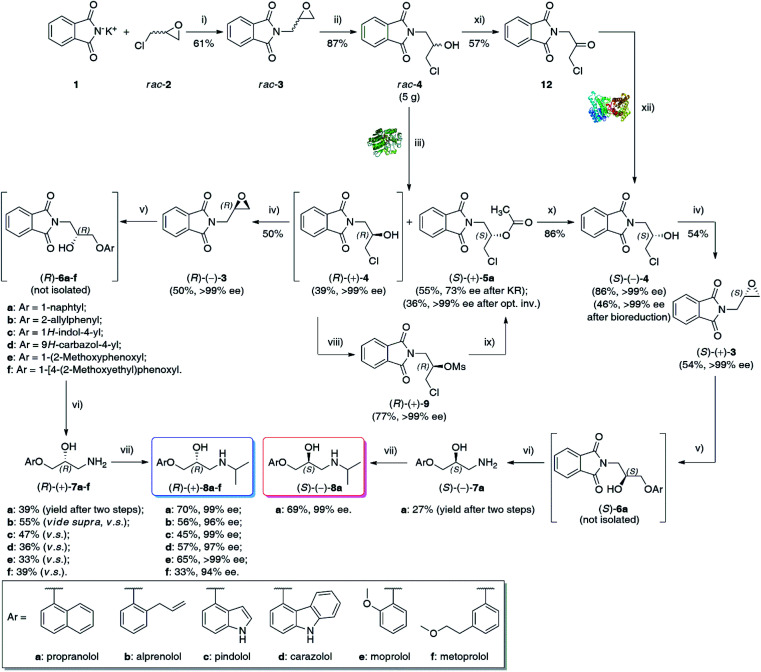
Chemoenzymatic synthesis of enantioenriched β-blockers 8a–f. Reagents and conditions: (i) *rac*-2 (7.0 equiv.), 24 h at 120 °C; (ii) 36% HCl, CHCl_3_, 30 min at 0–5 °C; (iii) vinyl acetate (3.0 equiv.), Amano PS-IM (25–50% w/w), TBME (20 mL/1 g of substrate *rac*-4), 24 h at 50 °C, 800 rpm (magnetic stirrer); (iv) anh. K_2_CO_3_ (2.0 equiv.), PhCH_3_, 24 h at 111 °C; (v) appropriate ArOH (1.0 equiv.), DBU (0.1 equiv.), xylene, for 24 h at 120 °C (under N_2_); (vi) anh. hydrazine (4.5 equiv.), xylene/2-PrOH (1 : 5 v/v), 2 h at 80 °C; (vii) acetone (3.0 equiv.), absolute EtOH, 45 min at RT, then NaBH_4_ (2.0 equiv.), 45 min at RT; (viii) MsCl (1.5 equiv.), Et_3_N (1.5 equiv.), dry CH_2_Cl_2_, 10 min at 0–5 °C, then 1 h at RT; (ix) AcOCs (4 equiv. for 0.16 mmol scale or 10 equiv. for 1.57 mmol scale), 18-Crown-6 (cat.), dry PhCH_3_, 120 h at 110 °C; (x) 98% H_2_SO_4_ (cat.), MeOH, 96 h at 35 °C; (xi) PCC (3.0 equiv.) added portion-wise over 15 min at 35 °C, CH_2_Cl_2_, then 24 h at 35 °C; (xii) 12 (65 mM final conc.), *E. coli*/Lk-ADH-Lica (25 mg mL^−1^), 1.0 mM NADH, 0.1 M Tris–HCl buffer (pH 7.5)/2-PrOH (90 : 10, v/v), DMSO (5% v/v), 48 h, 30 °C, 250 rpm (laboratory shaker).

Herein, we wish to report the chemoenzymatic method of the synthesis of (*S*)-(−)- and (*R*)-(+)-enantiomers of propranolol (8a), alprenolol (8b), pindolol (8c), carazolol (8d), moprolol (8e), and metoprolol (8f) from optically pure glycidyl phthalimide (3) as an intermediate. The key step of the synthetic pathway was the lipase-catalyzed kinetic resolution of racemic chlorohydrin *rac*-4 and/or asymmetric bioreduction of prochiral ketone 12 employing recombinant alcohol dehydrogenases (ADHs). Overall chemoenzymatic route for the preparation of enantiomeric β-adrenolytic agents using lipases as biocatalysts or ADH-catalyzed desymmetrization of prochiral ketone 12 is depicted in Scheme 1.

### Lipase-catalyzed KR of *rac*-5a and *rac*-4

The racemic substrate *rac*-4 and reference esters *rac*-5a–b required for biocatalytic investigations were obtained in a 2–3-step reaction sequence starting from cheap commercially available potassium phthalimide (1) and racemic epichlorohydrin (*rac*-2) (for details, see ESI†). The next critical issue was focused on synthesizing optically active key-intermediate 3. Initially, we performed lipase-catalyzed hydrolytic resolution of the corresponding acetate *rac*-5 under kinetically-controlled conditions following the procedure reported by Gelo and Šunjić.^25^ Disappointingly, in our hands, hydrolytic KR of *rac*-5 using native lipase from *Pseudomonas* (*Burkholderia*) *cepacia* (Amano PS) suspended in a homogenous mixture of 0.1 M aqueous K_2_HPO_4_ buffer (pH 7.5) and EtOH (90 : 10 v/v) failed as this protocol allowed to obtain faster-reacting enantiomer (*S*)-(−)-4 with a moderate enantiomeric excess (80% ee), and the remaining acetate (*R*)-(−)-5a with only 21% ee. In addition, this reaction proceeded with unsatisfactory enantioselectivity (*E* = 11) and low rate (21% conv. achieved after 72 h); therefore, we decided to examine modified hydrolytic conditions. However, additional optimization of this reaction was also unsuccessful (for details see ESI†).

Since hydrolytic KR of *rac*-5a was found troublesome due to the unfavorable enantiomeric purity of the resolved non-racemic products, we decided to change the synthetic strategy. In this regard, we applied a reverse variant of the above process – lipase-catalyzed transesterification of *rac*-4 using vinyl acetate as the acylating agent. Because such an approach has never been attempted before toward halohydrin derivative *rac*-4, we decided to implement a 5-step optimization procedure, which encompassed: (i) enzyme screening, (ii) medium selection, (iii) influence of temperature, (iv) kinetic analysis, and finally (v) validation of scalability of the enzymatic process. The influence of other factors commonly tested during enzyme-catalyzed reactions, such as (vi) influence of acyl group donor, (vii) type of enhancement-like additives and/or (viii) catalyst re-usability, were considered redundant. The optimization of each reaction parameter and up-scaling approach are discussed in detail in the following paragraphs.

In the first step of analytical-scale studies, to identify a lead candidate biocatalyst for the enantioselective acetylation of *rac*-4 carried out under kinetically-controlled conditions, we performed screening of a considerable number of commercially available enzyme preparations, including mostly lipases and one esterase (pig liver esterase, PLE) (for details, see ESI†). The enzymes were tested in a model reaction consisting of a solution of racemic substrate *rac*-4 (used at a 50 mg scale; 0.21 mmol) in a mixture of vinyl acetate as an irreversible acetyl donor and *tert*-butyl methyl ether (TBME) as the co-solvent. To push the equilibrium towards the side of the formation of the product (ester), we decided to use vinyl acetate with increased 3-fold molar excess in relation to *rac*-4. All the enzymatic reactions were stirred at 30 °C using a magnetic stirrer set at 800 rpm and regularly traced by GC analysis. The KR assays were terminated either when *ca.* 50% conversion was achieved (according to GC) or 72 h had passed in the case of slower reactions. After isolation and subsequent chromatographic purification of the resolution products, the evaluation of their enantiomeric excesses (% ee) and the *E*-values has been accomplished using HPLC on chiral column. The most promising results in terms of enzymes' catalytic activity were collected in Table 1. Those biocatalysts which turned out to be inactive toward *rac*-4 (*i.e.*, Amano M, Amano 10 Lipase M, Lipase AY Amano 30, and PLE) or exhibited residual activity (*i.e.*, Chirazyme L-2, C-3, TL-Immobead 150, Lipozyme RM IM, PS-Immobead 150, Amano PS) were omitted for clarity. The dataset indicates that lipase preparations manufactured from different bacteria species (*i.e.*, Chirazyme L-10, Lipozyme TL IM, Amano PS-IM, Amano PS-C II, and Amano AK) were the most efficient biocatalysts as they catalyzed the transformation of *rac*-4 with moderate-to-high enantioselectivity (*E* = 12–59). On the contrary, the fungi-originated biocatalysts (*i.e.*, Novozym 435, Lipozyme 435, and Chirazyme L-2, C-2) exhibited relatively high catalytic activity, allowing to reach >62% conversion after 72 h, albeit their mode of action was significantly less selective toward *rac*-4 enantiomers (*E* = 1), thus furnishing both optically active KR products, (*R*)-(+)-4 and (*S*)-(+)-5a, in a very low range of enantiomeric enrichment (7–15% ee). After several KR attempts with various enzymes in the presence of vinyl acetate, it was found that the most promising results in terms of the reaction rate and enantioselectivity were obtained when lipases from *Alcaligenes* sp. (Chirazyme L-10) and *Burkholderia cepacia* (BCL) immobilized either on ceramic (Amano PS-C II) or diatomaceous earth (Amano PS-IM) were applied as the biocatalysts, respectively. Among them, unquestionably the most active were Chirazyme L-10 and Amano PS-C II, which both catalyzed enantioselective transesterification of racemic alcohol *rac*-4 at high 54–58% conv., achieved after 24 h, and with the *E*-factor reaching up to 59.

**Table 1:** Lipase screening for the enantioselective transesterification of *rac*-4 with vinyl acetate under KR conditions in TBME

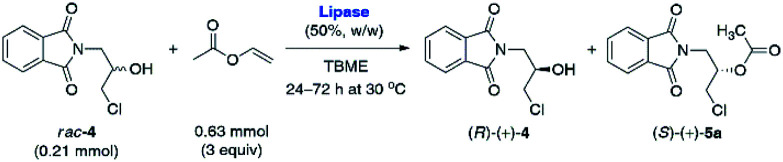						
Entry	Lipase preparation^a^	*t* (h)	Conv.^b^ (%)	ee_s_^c^ (%)	ee_p_^c^ (%)	*E* d
1	Novozym 435	72	63	12	7	1
2	Lipozyme 435	72	63	15	9	1
3	Chirazyme L-2, C-2	72	62	13	8	1
4	Chirazyme L-10	24	58	>99	73	32
5	Lipozyme TL IM	72	50	88	87	42
6	Amano PS-IM	72	55	>99	81	49
7	Amano PS-C II	24	54	>99	84	59
8	Amano AK	72	62	93	58	12

a Conditions: *rac*-4 50 mg, lipase 25 mg, TBME 1 mL, vinyl acetate 54 mg, 58 μL (3 equiv.), 30 °C, 800 rpm (magnetic stirrer).

b Based on GC, for confirmation, the % conversion was calculated from the enantiomeric excess of the unreacted alcohol (ee_s_) and the product (ee_p_) according to the formula conv. = ee_s_/(ee_s_ + ee_p_).

c Determined by chiral HPLC analysis.

d Calculated according to Chen *et al.*,^26^ using the equation: *E* = {ln[(1 − conv.)(1 − ee_s_)]}/{ln[(1 − conv.)(1 + ee_s_)]}.

Although Amano PS-IM displayed significantly lower catalytic activity toward *rac*-4, achieving 55% conv. after extended reaction time (72 h), its enantioselectivity was convincing, as the slower reacting stereoisomer (*R*)-(+)-4 was isolated in enantiomerically pure form (>99% ee). From the set of the screened lipases, it was the Amano PS-IM preparation that we selected for further optimization studies. Such proceeding was dictated by the fact that Amano PS-C II and Chirazyme L-10 lipases are no longer available at the enzyme suppliers, which could hinder further industrialization of the developed process.

Having determined that Amano PS-IM from *Burkholderia cepacia* could be a suitable lipase for the kinetic resolution of *rac*-4, other reaction parameters that affect the enzymatic catalysis were investigated. It is well-documented that the so-called ‘*medium engineering*’ is one of the most crucial factors for biocatalytic processes carried out with lipases in low-water systems (*i.e.*, neat organic solvents) as the nature of the reaction medium can markedly modulate enzyme's catalytic activity, selectivity, and thermal stability.^27^ Moreover, in some cases, an organic solvent can spectacularly change the lipases' regioselectivity^28^ and stereochemical preference,^29^ which may have practical application while planning synthetic campaigns. All these phenomena are a consequence of interactions between the solvent and enzyme molecules or solvent and enzyme–substrate complex, which might produce/promote conformational changes resulting in higher rigidity of the protein molecule (as a result of less water-induced hydrogen bonding in favor of stronger intra-protein electrostatic interactions), invading the active site, or altering the solvation of the transition state.^30^ Therefore, the next stage of enzymatic analytical-scale studies aimed to find the most suitable co-solvent system for the enantioselective transesterification of racemic alcohol *rac*-4 using 3 equiv. of vinyl acetate as acetyl donor. Similarly to the reaction conditions used in our previous contribution, the evaluation of the solvent impact on the reaction rate and stereochemical outcome was undertaken by suspending Amano PS-IM lipase in organic solvents of varying polarity values, within which the racemic substrate *rac*-4 was able to form homogenous solutions (Table 2).

**Table 2:** The co-solvent screening for (Amano PS-IM)-catalyzed KR of *rac*-4 with vinyl acetate after 72 hours

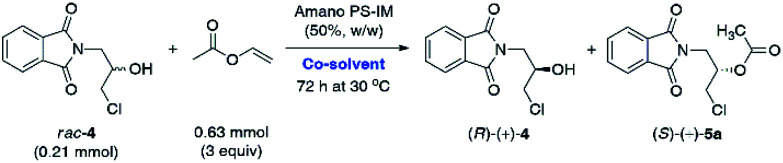					
Entry	Co-solvent^a^ (log *P*)^b^	Conv.^c^ (%)	ee_s_^d^ (%)	ee_p_^d^ (%)	*E* e
1	1,4-Dioxane (−0.31)	<5	N.D.^f^	N.D.^f^	N.D.^f^
2	CH_3_CN (0.17)	<5	N.D.^f^	N.D.^f^	N.D.^f^
3	Acetone (0.20)	23	29	95	52
4	THF (0.40)	31	39	88	23
5	Vinyl acetate (0.54)	56	74	93	22
6	TBME (0.96)	55	>99	81	49
7	*tert*-Amyl alcohol (1.09)	54	97	83	45
8	PhCH_3_ (2.52)	54	97	84	48

a Conditions: *rac*-4 50 mg, Amano PS-IM 25 mg, organic solvent 1 mL, vinyl acetate 54 mg, 58 μL (3 equiv.), 30 °C, 800 rpm (magnetic stirrer).

b Logarithm of the partition coefficient of a given solvent between *n*-octanol and water according to ChemBioDraw Ultra 13.0 software indications.

c Based on GC, for confirmation, the % conversion was calculated from the enantiomeric excess of the unreacted alcohol (ee_s_) and the product (ee_p_) according to the formula conv. = ee_s_/(ee_s_ + ee_p_).

d Determined by chiral HPLC analysis.

e Calculated according to Chen *et al.*,^26^ using the equation: *E* = {ln[(1 − conv.)(1 − ee_s_)]}/{ln[(1 − conv.)(1 + ee_s_)]}.

f Not determined.

To obtain comparable results, we decided to carry out all the KR reactions of *rac*-4 deliberately for 72 h. Among the eight solvents chosen, similarly high catalytic activity and enantioselectivity were obtained with moderately non-polar, water-immiscible solvents (log *P* 0.54–2.52), including already tested TBME as well as vinyl acetate, 2-methyl-2-butanol (*tert*-amyl alcohol), and toluene (PhCH_3_), respectively. Among all the media mentioned above, one of the highest enantiomeric purity (93% ee) of the formed acetate (*S*)-(+)-5a was afforded in neat vinyl acetate when the reaction was terminated at 56% conv. Interestingly, even higher enantiomeric enrichment of (*S*)-(+)-5a (95% ee) was observed when the KR was carried out in polar acetone as the co-solvent; however, the reaction proceeded sluggishly, affording a low 23% conv. of the starting material *rac*-4. In turn, lipase Amano PS-IM suspended in TBME appeared to be superior to the majority of other tested catalytic systems as KR of *rac*-4 resulted in the isolation of enantiomerically pure alcohol (*R*)-(+)-4 (>99% ee). On the contrary, a drastically unfavorable solvent effect on the reaction rate leading to almost total deactivation of the enzyme was observed in the case of highly polar (−0.31 < log *P* < 0.17), water-miscible solvents, such as 1,4-dioxane and CH_3_CN. A significant deterioration of the catalytic activity of Amano PS-IM in these cases is most likely due to the ability of the water-miscible organic solvents to strip off the essential structural water from the enzyme's surface, which causes hydration changes detrimental to the stability of the protein.^31^ Based on the above results, TBME was chosen as the optimal co-solvent in the following work.

In the next step, we investigated the effect of temperature on the outcome of the Amano PS-IM-catalyzed enantioselective KR of *rac*-4 (Table 3). The reason for performing this evaluation was two-fold: to overcome low reaction rates achieved in the previous attempts and, on the other hand, to identify if the enzyme can retain its initial enantioselectivity and stability in the reaction system at elevated temperatures.

**Table 3:** Temperature effect on (Amano PS-IM)-catalyzed KR of *rac*-4 with vinyl acetate in TBME

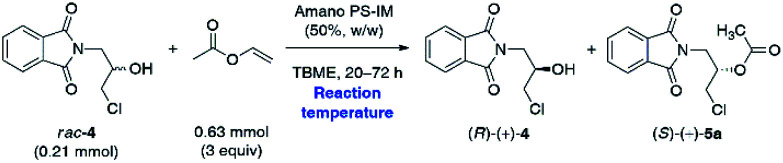						
Entry	Temp.^a^ (°C)	*t* (h)	Conv.^b^ (%)	ee_s_^c^ (%)	ee_p_^c^ (%)	*E* d
1	30	72	55	>99	81	49
2	40	20	52	96	88	61
3	50	20	56	>99	79	44

a Conditions: *rac*-4 50 mg, Amano PS-IM 25 mg, TBME 1 mL, vinyl acetate 54 mg, 58 μL (3 equiv.), 800 rpm (magnetic stirrer).

b Based on GC, for confirmation, the % conversion was calculated from the enantiomeric excess of the unreacted alcohol (ee_s_) and the product (ee_p_) according to the formula conv. = ee_s_/(ee_s_ + ee_p_).

c Determined by chiral HPLC analysis.

d Calculated according to Chen *et al.*,^26^ using the equation: *E* = {ln[(1 − conv.)(1 − ee_s_)]}/{ln[(1 − conv.)(1 + ee_s_)]}.

The analysis of the obtained data revealed that the reaction temperature appeared to impact the catalysis efficiency dramatically. The reactions carried out at 40 °C and 50 °C resulted in higher rates with the same enhanced enantiocontrol. The first increase in temperature from 30 °C to 40 °C led to a decrease in reaction time to reach >50% conv. from 72 h to 20 h, practically, with the maintenance of all values obtained in the kinetic resolution of *rac*-4. Notably, when Amano PS-IM was used at 40 °C, the *E*-value remained remarkably high; however, a slight drop in enantiomeric purity of the isolated alcohol (*R*)-(+)-4 (96% ee) was detected. A new increment in temperature to 50 °C was responsible for a decrease in the enantiomeric ratio to 44, although the reaction time required to reach 56% conv. was also reduced to 20 h. Increasing the temperature to 50 °C greatly enhanced the reaction rate, fulfilling the desire to shorten the reaction times, and at the same time, the enantiomeric purity of (*R*)-(+)-4 was maintained at desired >99% ee.

**Fig. 3:**
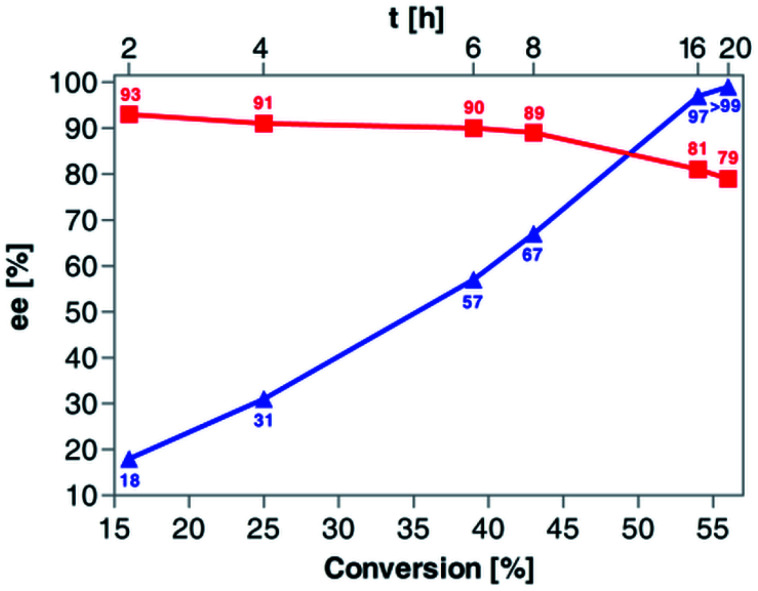
Dependence of enantiomeric excess (% ee) of (*S*)-(+)-5a (red curve, 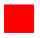) and (*R*)-(+)-4 (blue curve, 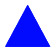) on the conversion degree of *rac*-4 during (Amano PS-IM)-catalyzed enantioselective acetylation with vinyl acetate in a TBME solution at 50 °C (magnetic stirrer, at 800 rpm).

Based on the above results, 50 °C was arbitrarily selected as the optimal temperature in the following work. Since our primary goal was to discover optimal conditions for KR of *rac*-4 that would allow us to achieve around 50% conv., and the recovery of the remaining alcohol (*R*)-(+)-4 and the resulting acetate (*S*)-(+)-5a with the highest possible optical purities, in the next step of the optimization studies, we decided to evaluate the reaction kinetics more in-depth (Fig. 3). For this purpose, six independent KR reactions were carried out under optimal conditions and terminated every 2 h for the first 8 h period and then after 16 h and 20 h, respectively. After convenient workup and chromatographic purification of each crude reaction mixture, the prepared samples were analyzed by chiral HPLC. The correlation plot covering the relationship between % ee-values of both KR products and the time profile of conversion of *rac*-4 in the reaction catalyzed by Amano PS-IM showed that the enzymatic process failed to obtain enantiomerically pure acetate (*S*)-(+)-5a as it was isolated with enantiomeric excess in the range of 79–93%. In turn, the remaining alcohol (*R*)-(+)-4 could be synthesized in enantiomerically pure form when the conversion reached >55%, which required proceeding with KR of *rac*-4 for at least 20 h.

Although the selected enzyme turned out to be moderately selective in the enantiomeric discrimination of *rac*-4, we found this process highly valuable for the synthesis of optically active β-blockers. Having optimized the conditions for enzymatic acylation, next 2.5 g and 5.0 g of the substrate *rac*-4 were used for each KR attempt (Table 4). To our delight, preparative-scale enantioselective transesterification of *rac*-4 with Amano PS-IM lipase afforded (*R*)-(+)-4 with >99% ee in 39–40% yield after 23–24 h regardless of the applied scale. These results showed that the reactions could be scaled up by a factor of 100 without any encountered obstacles, demonstrating the potential usefulness of the enzymatic protocol. Moreover, during scale-up studies, we have found that decreasing the amount of the lipase preparation by half (from 50% w/w to 25% w/w in ratio to substrate *rac*-4) has not affected the rate and stereochemical outcome of the KR process, which could potentially enhance the economic feasibility of the β-blockers production.

**Table 4:** Multigram-scale (Amano PS-IM)-catalyzed KR of *rac*-4 with vinyl acetate in TBME at 50 °C

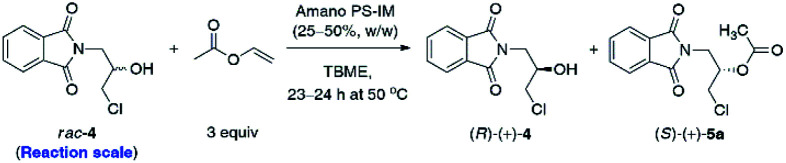						
Entry	Scale (g)	*t* (h)	Conv.^a^ (%)	ee_s_^b^ (%)/yield^c^ (%)	ee_p_^b^ (%)/yield^c^ (%)	*E* d
1	2.5^e^	23	57	>99/40	74/48	34
2	5.0^f^	24	58	>99/39	73/55	32

a Based on GC, for confirmation, the % conversion was calculated from the enantiomeric excess of the unreacted alcohol (ee_s_) and the product (ee_p_) according to the formula conv. = ee_s_/(ee_s_ + ee_p_).

b Determined by chiral HPLC analysis.

c Isolated yield after column chromatography using a gradient of a mixture of CHCl_3_/acetone (98 : 2, 95 : 5 v/v).

d Calculated according to Chen *et al.*,^26^ using the equation: *E* = {ln[(1 − conv.)(1 − ee_s_)]}/{ln[(1 − conv.)(1 + ee_s_)]}.

e Conditions: *rac*-4 2.5 g, Amano PS-IM 1.25 g (50% w/w), TBME 50 mL, vinyl acetate 2.7 g, 2.88 mL (3 equiv.), at 50 °C, 800 rpm (magnetic stirrer).

f Conditions: *rac*-4 5 g, Amano PS-IM 1.25 g (25% w/w), TBME 100 mL, vinyl acetate 5.4 g, 5.77 mL (3 equiv.), at 50 °C, 800 rpm (magnetic stirrer).

### Synthesis of enantioenriched (*R*)-(+)-β-blockers 8a–f

With enantiomerically pure chlorohydrin (*R*)-(+)-4 in hand, we have focused on optimizing the reaction conditions for the remaining steps of the synthesis. The major goal of these studies was to develop highly efficient and selective transformations to obtain enantiomeric products in high yields and preserved optical purity. After straightforward K_2_CO_3_-catalyzed cyclization of (*R*)-(+)-4 to enantiomerically pure epoxide (*R*)-(−)-3 (>99% ee) isolated in 50% yield, and the subsequent one-pot DBU-mediated regioselective ring-opening of the formed oxirane with various phenols (ArOH), followed by the removal of the phthalimido group employing hydrazine in isopropanol according to the classical Ing-Manske procedure,^32^ a set of (*R*)-configurated aryloxy amino alcohols (*R*)-(+)-7a–f were obtained in up to 55% yield after two steps.

Having released the amine moiety from the corresponding phthalimides, we focused our attention on introducing the terminal isopropyl group to afford title (*R*)-(+)-β-blockers. This task was achieved in a stepwise “one-pot” procedure applying the reductive amination protocol reported by Cardillo *et al.*,^33^ which involved treatment of an ethanolic solution of aminoalcohols (*R*)-(+)-7a–f with 3 equiv. of acetone, followed by *in situ* reductions of the formed Schiff bases with 2 equiv. of NaBH_4_. The reactions proceeded smoothly at room temperature, furnishing after 1.5 h the respective enantiomerically enriched β-blockers (*R*)-(+)-8a–f in the 33–70% yield range, respectively. It is worth mentioning that the elaborated four-step reaction sequence realized *via* double ‘one-pot’ procedures allowed to obtain propranolol [(*R*)-(+)-8a], pindolol [(*R*)-(+)-8c], and moprolol [(*R*)-(+)-8e] with excellent enantiomeric purities (99–99.9% ee). Unfortunately, in the case of other synthesized (*R*)-(+)-β-blockers, some racemization occurred during the syntheses as the % ee-values were less astonishing. Following the devised synthetic pathway, alprenolol [(*R*)-(+)-8b] was obtained with 96% ee; carazolol [(*R*)-(+)-8d] with 97% ee, and metoprolol [(*R*)-(+)-8f] with 94% ee, respectively.

### Synthesis of (*S*)-propranolol [(*S*)-(−)-8a]

From the retrosynthetic analysis of the planned synthesis, it must be mentioned that the (*S*)-configuration of the key-precursor 3 is required for the formation of medicinally relevant (*S*)-(−)-β-blockers. As this task could not be achieved in a straightforward manner *via* lipase-catalyzed kinetic resolution of the racemic substrates, *rac*-4 and *rac*-5a, we decided to selectively invert the absolute configuration of a determined stereogenic center of the hydroxyl group in (*R*)-(+)-4 to obtain desired (*S*)-(−)-4, and then the key (*S*)-(+)-3. For this purpose, we have tested two principal methods for chiral alcohol inversion: the Mitsunobu reaction (Table 5, entries 1 and 2) and the S_*N*_2-type reaction of the secondary mesylate with oxygen carboxylate nucleophiles (Table 5, entries 3 and 4).

**Table 5:** Inversion of the absolute configuration on the stereogenic center of enantiomerically pure chlorohydrin (*R*)-(+)-4*via* direct Mitsunobu esterification of (*R*)-(+)-4 (Method A–B) or indirect acetolysis of the respective mesylate (*R*)-(+)-9 (Method C–D)

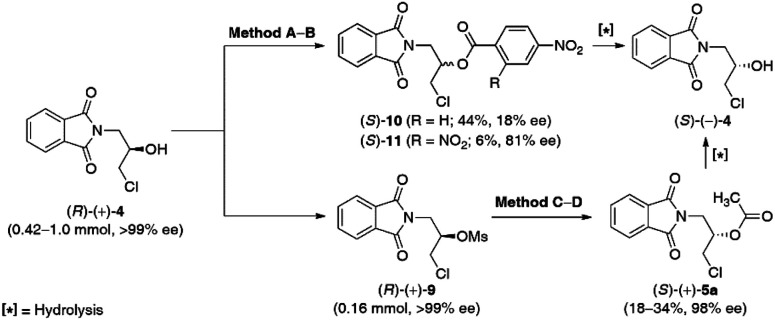					
Entry	Substrate	Method^a^	Conv.^b^ [%]	Yield^c^ [%]	ee_p_^d^ [%]
1	(*R*)-(+)-4 (>99% ee)	A	>99	44	18
2		B	N.D.^e^	6	81
3	(*R*)-(+)-9 (>99% ee)	C	70	18	98
4		D	92	34 (36)^f^	98 (>99)^f^

a Method A: (*R*)-(+)-4 (100 mg, 0.42 mmol, >99% ee), 4-nitrobenzoic acid (1.5 equiv.), DEAD (1.5 equiv.), Ph_3_P (1.5 equiv.), dry THF, 12 h, 25 °C; Method B: (*R*)-(+)-4 (239 mg, 1.00 mmol, >99% ee), 2,4-dinitrobenzoic acid (1 equiv.), (2-hydroxybenzyl)diphenylphosphine oxide (cat.), xylene, reflux with a Dean–Stark trap for 48 h; method C: (*R*)-(+)-9 (50 mg, 0.16 mmol, >99% ee), AcOCs (5.0 equiv.), 18-crown-6 (cat.), dry PhCH_3_, 120 h, 90 °C; method D: (*R*)-(+)-9 (50 mg, 0.16 mmol, >99% ee), AcOCs (5.0 equiv.), 18-crown-6 (cat.), dry PhCH_3_, 120 h, 110 °C.

b Determined by GC analysis using the calibration curve.

c Isolated yield after silica-gel column chromatography.

d Enantiomeric excess of optically active compounds, *i.e.*, (*S*)-10, (*S*)-11, and (*S*)-(+)-5a, determined by HPLC analysis.

e Not determined.

f Performed on 500 mg (1.57 mmol) scale of (*R*)-(+)-9.

Since the Mitsunobu reaction is a direct method for the optical inversion of compounds possessing hydroxylated stereocenters, we started the investigations with this approach toward (*R*)-(+)-4. The benchmark reaction consisted of triphenylphosphine (PPh_3_) as a reductant, diethyl azodicarboxylate (DEAD) as an aza-dienophilic oxidant, and the respective carboxylic acid as a nucleophile (*i.e.*, acetic or 4-nitrobenzoic acid). After the initial screening of the reaction conditions, it turned out that no progress was observed with acetic acid (not shown herein), while 4-nitrobenzoic acid with higher Brønsted acidity [p*K*_a_(H_2_O) = 3.4] led to >99% conv. of (*R*)-(+)-4 into the corresponding Mitsunobu-like 4-nitrobenzoate (*S*)-10 in 44% yield; however, with a very low 18% ee.

Classical Mitsunobu methodology requires using stoichiometric amounts of hazardous DEAD and highly toxic PPh_3_, which generate hydrazinedicarboxylate and triphenylphosphine oxide (wastes that can be difficult to separate from the reaction product); therefore, the inversion of chiral alcohol (*R*)-(+)-4 was simultaneously investigated under modified waste-free and redox-neutral Mitsunobu conditions reported by Beddoe *et al.*^34^ This protocol assumes the usage of 2,4-dinitrobenzoic acid [p*K*_a_(H_2_O) = 1.4] as an efficient coupling partner and the catalytic amount of (2-hydroxybenzyl)diphenylphosphine oxide. In this case, the inversion produces water as the sole by-product, which has to be azeotropically removed from either toluene or xylenes by using a Dean–Stark trap to prevent hydrolysis of the kinetically and thermodynamically unstable phosphonium salt intermediates, critical for the catalytic cycle. Unfortunately, the inverted ester product (*S*)-11 was formed in a yield of only 6% and an enantiomeric excess of 81%. The low yield of this process is mostly due to either inefficient removal of water from the reaction mixture and/or decomposition of the sensitive to the strong acids phthalimide-like substrate (*R*)-(+)-4 at elevated temperature.

Poor reactivity in the Mitsunobu reactions of the corresponding alcohol (*R*)-(+)-4, in conjunction with racemization problems, led us to develop an alternative method. To establish the optimal conditions for the preparation of (*S*)-(−)-4, the optically active chlorohydrin (*R*)-(+)-4 was subjected to an activation step with mesyl chloride in the presence of Et_3_N and DMAP in dry dichloromethane (CH_2_Cl_2_) as the solvent at room temperature. The conversion of the hydroxyl moiety in (*R*)-(+)-4 into a better leaving group proceeded with the retention of the absolute configuration to obtain optically active mesylate (*R*)-(+)-9 isolated by chromatography in 77% yield with >99% ee.

Next, the S_*N*_2 reaction of the mesylate (*R*)-(+)-9 with 5 equiv. of cesium acetate (CsOAc) as a nucleophile in the presence of the catalytic amount of 18-crown-6 in boiling PhCH_3_ for 120 h afforded the inverted acetate (*S*)-(+)-5a with 70% conv. and in 18% isolated yield (when the reaction was carried out at 90 °C) or with 92% conv. and in 34% isolated yield (when the reaction was carried out at 110 °C), respectively. Hopefully, in both cases, mesylate (*R*)-(+)-9 was entirely resistant to basic elimination and underwent facile acetolysis with the acetate ion, avoiding drastic racemization to yield (*S*)-(+)-5a with 98% ee. Surprisingly, repeating the reaction using a half-gram-scale of the substrate (*R*)-(+)-9 afforded (*S*)-(+)-5a in 36% yield and >99% ee (Table 5, entry 4).

In the next step, we applied H_2_SO_4_-catalyzed hydrolysis of (*S*)-(+)-5a in methanol, which proved to be very efficient in removing the acetyl group under mild conditions (35 °C), leading to the desired (*S*)-(−)-4 in 86% isolated yields without any racemization. Finally, employing optimized conditions for (*R*)-(+)-β-blockers, chlorohydrin (*S*)-(−)-4 was functionalized toward (*S*)-propranolol [(*S*)-(−)-8a, 99% ee] in 10% total yield after 5-steps.

### Bioreduction of α-chloroketone 12 catalyzed by whole cells

Since we envisioned that (*S*)-(+)-3 could be directly accessible from α-chloroketone 12*via* enzymatic reduction and cyclization of the resulting chlorohydrin (*S*)-(−)-4, our next step was to investigate stereoselective transfer-biohydrogenation of prochiral ketone 12, employing various whole-cell biocatalysts possessing alcohol dehydrogenases (ADHs) (Table 6). To identify the most active and selective enzyme preparation, we tested a set of lyophilized microbial cells occurring either as wild-type microorganisms or *Escherichia coli* cells containing the recombinant proteins (*E. coli*/ADHs) (for details, see ESI†).

**Table 6:** An analytical-scale studies on stereoselective reduction of 2-(3-chloro-2-oxopropyl)-1*H*-isoindole-1,3(2*H*)-dione (12, 10 mM) with different biocatalysts after 48 h

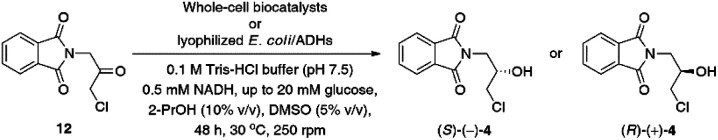				
Entry	Biocatalyst^a^	Strain	Conv.^b^ [%]	ee_p_^c^ [%] (config.^d^)
1	*Komagataella phaffi*/*Pichia pastoris*	ATCC 76273	0	N.D.^e^
2	*Pseudomonas* sp.	DSM 6978	14	N.D.^e^
3	*Arthrobacter* sp.	DSM 7325	89	71 (*R*)
4	Isolate *Actinomyces* sp. SRB-AN040	FCC025	>99	68 (*R*)
5	Isolate *Actinomyces* sp. SRB-AN053	FCC027	0^f^	N.D.^e^
6	Isolate *Actinomyces* sp. ARG-AN024	FCC014	75	66 (*R*)
7	Isolate ARG-AN025	FCC015	97	35 (*R*)
8	Isolate USA-AN012	FCC021	96	60 (*R*)
9	*E. coli*/RasADH	—g	53^f^	N.D.^e^
10	*E. coli*/SyADH	—g	<5	N.D.^e^
11	*E. coli*/ADH-A	—g	30^f^	N.D.^e^
12	*E. coli*/LB-ADH	—g	<5	N.D.^e^
13	*E. coli*/Lk-ADH	—g	13	N.D.^e^
14	*E. coli*/Lk-ADH Prince	—g	83	94 (*S*)
15	*E. coli*/Lk-ADH-Lica	—g	>99	>99 (*S*)

a Reaction conditions: lyophilized biocatalyst (10 mg), 20 mM glucose, 0.5 mM NADH, 0.1 M Tris–HCl buffer (pH 7.5)/2-PrOH (500 μL, 90 : 10, v/v), DMSO (5% v/v), 48 h, 30 °C, 250 rpm (laboratory shaker).

b Conversion (%) (*i.e.*, consumption of substrate 12) and products yields (*i.e.*, formation of *non-rac*-4) were determined by GC and HPLC (for confirmation) analyses using the calibration curve.

c Determined for *non-rac*-4 by HPLC analysis on a chiral stationary phase.

d Absolute configuration of *non-rac*-4 established by comparison of HPLC picks elution order with enantiomeric standards. Major enantiomer is shown in parentheses.

e Not determined.

f A complex mixture of several products was observed, including the starting material 12 and the product *non-rac*-4.

g Reaction conducted without glucose.

The model ADH-catalyzed bioreductions of 12 were performed under standard biocatalytic conditions using 10 mM of the prochiral substrate and 10 mg of lyophilized cells suspended in 100 mM Tris–HCl buffer (pH 7.5) in the presence of 0.5 mM of the external nicotinamide cofactor, without air access for 48 h at 30 °C and 250 rpm (laboratory shaker). Since NADPH is more costly and less stable than the non-phosphorylated counterpart NADH, the experiments were performed in the presence of NADH taking the risk of losing some reactivity as, *i.e.*, most of the studied ADHs are NADPH dependent (except *E. coli*/ADH-A with recombinant protein originating from *Rhodococcus ruber*). To devise the most straightforward biocatalytic system for an economically feasible bioprocess, we decided to use 10% v/v of cheap 2-propanol (2-PrOH) as a sacrificial hydride source for NAD(P)H-recycling. Moreover, 2-PrOH acts as a co-solvent, which helps dissolve water-insoluble compounds and increase their concentrations in an aqueous reaction media. In our case, to enhance the solubility of ketone 12 in Tris–HCl buffer/2-PrOH (90 : 10, v/v), we were forced to also add 5% v/v of DMSO. Moreover, in the case of the reactions conducted with wild-type microbial cells, the supplemental 20 mM of glucose was provided just in case to support an alternative NAD(P)H-recycling system *via* the Embden–Meyerhof–Parnas (EMP) metabolic pathway.

Screening experiments revealed that the prochiral substrate 12 was probably too sterically-congested for most of the tested whole-cell biocatalysts to be transformed in the active sites of the ADHs. Nevertheless, there might also be other reasons for the low catalytic activity of the examined *E. coli*/ADHs preparations since even *E. coli*/RasADH and *E. coli*/SyADH, which are known as very efficient biocatalysts for the reduction of “bulky–bulky” substrates,^35^ failed as well. This can also be attributed to negative consequences of the absence of NADPH, or an unsuitable cofactor regeneration system tailored for the catalytic system. Nevertheless, one of the used *E. coli*/ADH preparation, namely *E. coli*/Lk-ADH-Lica, turned out to be very efficient biocatalysts furnishing stereoselective reduction of the carbonyl group present in 12, leading to the formation of desired chlorohydrin (*S*)-(−)-4 with >99% conv. and >99% ee. Although it is well-known that ADH from *Lactobacillus kefir* is NADPH-dependent anti-Prelog (*R*)-specific enzyme, the change in CIP priority order around the chiral center resulted in (*S*)-alcohol.

To our delight, scaling-up of the *E. coli*/Lk-ADH-Lica-catalyzed stereoselective bioreduction of 12 allowed us to obtain enantiomerically pure (*S*)-(−)-4 (>99% ee) with an excellent >99% conv.; however, in only 46% isolation yield. The relatively low isolation yield can be explained by the mass loss during the workup procedure and chromatographic purification; nevertheless, we believe this method is potentially more attractive in terms of industrial application than the ‘KR-optical inversion route’. It dramatically shortened the synthetic pathway toward title APIs by cutting three difficult steps, which provided the key-precursor (*S*)-(−)-4 in a low 9.3% overall yield from *rac*-4. The benefits of such a synthetic strategy encompass minimizing workups and waste generation by eliminating purification of synthetic intermediates as well as time invested (‘time economy’) and processing (‘pot economy').

## Conclusions

In conclusion, a novel chemoenzymatic route for the synthesis of non-racemic β-blockers was elaborated. The key step was the asymmetric synthesis of the enantiopure 2-[(2*S*)-oxiran-2-ylmethyl]-1*H*-isoindole-1,3(2*H*)-dione, which was realized either *via* lipase-catalyzed kinetic resolution of the respective racemic phthalimido-chlorohydrin and the subsequent 3-step optical inversion strategy or stereoselective bioreduction of the corresponding prochiral ketone, followed by intramolecular base-promoted epoxide ring formation. Optimization of the KR process allowed selecting the most efficient biocatalytic system, which consisted of commercial Amano PS-IM lipase suspended in *tert*-butyl methyl ether and 3 equiv. of vinyl acetate as an acyl donor. The (Amano PS-IM)-catalyzed kinetic resolution of racemic chlorohydrin performed at 5 g-scale for 24 h at 50 °C, furnished enantiomerically pure 2-[(2*R*)-2-hydroxypropyl]-1*H*-isoindole-1,3(2*H*)-dione. Three-step stereoinversion approach toward the optically active (*R*)-chlorohydrin-synthon using acetolysis of the corresponding mesylate employing AcOCs in the presence of 18-crown-6, followed by acid-catalyzed hydrolysis of the formed (2*S*)-1-chloro-3-(1,3-dioxo-1,3-dihydro-2*H*-isoindol-2-yl)propan-2-yl acetate allows the formation of enantiomerically pure (*S*)-counterpart with >99% ee. Alternatively, the biocatalytic anti-Prelog reduction of corresponding prochiral ketone to (*S*)-chlorohydrin-synthon was successfully conducted with high enantioselectivity (>99% ee) and 46% yield using *E. coli*/Lk-ADH-Lica preparation containing recombinant protein originating from *Lactobacillus kefir*. Finally, six exemplary β-blockers, namely propranolol, alprenolol, pindolol, carazolol, moprolol, and metoprolol, were obtained with very high enantiomeric excess in the range of 96–99.9% after five-step chemical route.

## Author contributions

P. B.: conceptualization, methodology, validation, investigation, formal synthesis and analysis, data curation and processing, writing – original draft, writing – review & editing & revision, visualization, supervision, project administration, funding acquisition; B. Z.: investigation, formal synthesis and analysis; N. P.: investigation; M. W.: investigation; T. R.: investigation; W. K.: writing – original draft, proofreading.

## Conflicts of interest

The authors declare no conflicts of interest. All authors have approved the final version of the manuscript.

## Supplementary Material

RA-012-D2RA04302E-s001
