# Supplementary material for: Development of a novel chemoenzymatic route to enantiomerically enriched β-adrenolytic agents. A case study toward propranolol, alprenolol, pindolol, carazolol, moprolol, and metoprolol
Source: RSC Adv. 2022 Aug 10;12(34):22150–60. doi: 10.1039/d2ra04302e (PMC9364081; doi:10.1039/d2ra04302e)
Supplement: RA-012-D2RA04302E-s001 [file RA-012-D2RA04302E-s001.pdf]

Electronic Supplementary Material (ESI) for RSC Advances.
This journal is © The Royal Society of Chemistry 2022

Supporting Information

for

Development of Novel Chemoenzymatic Route to Enantiomerically Enriched β-Adrenolytic Agents. A Case Study Toward Propranolol, Alprenolol, Pindolol, Carazolol, Moprolol, and Metoprolol

Paweł Borowiecki,[a,*] Beata Zdun,[a] Natalia Popow,[a] Magdalena Wiklińska,[a] Tamara Reiter[b] and Wolfgang Kroutil[b]

[a] Warsaw University of Technology, Faculty of Chemistry, Koszykowa St. 75, 00-662 Warsaw, Poland.
[b] Institute of Chemistry, University of Graz, NAWI Graz, BioTechMed Graz, Field of Excellence BioHealth, Heinrichstrasse 28, 8010 Graz, Austria

* Corresponding author: Dr. Paweł Borowiecki (Email: pawel.borowiecki@pw.edu.pl; Website: http://lbb-wut-borowiecki.ch.pw.edu.pl/)

Table of contents

1. Materials and methods ……………………………..……………..…….…… S2–S4
2. Experimental synthetic procedures and analytical data for obtained compounds ….. S4–S26
3. Discussion on the results of synthetic procedures ……………………..…….…… S27–S30
Table S1. Enzyme screening for hydrolytic KR of *rac*-**5a** ……………………..…..…. S28
Table S2. Co-solvent screening for (NovoCor AD L)-catalyzed KR of *rac*-**5a** ………..… S29
Table S3. Conditions screening for the benchmark synthesis of *rac*-**6a** …………….……… S29
Table S4. Optimal analytical separation conditions of synthesized compounds by chiral columns – Chiralcel OD-H or Chiralpak AD-H (Diacel) ………...……............… S31–S32
Copies of HPLC chromatograms ……….……………………………….......... S33–S59
Copies of NMR, HRMS, FTMS, and IR spectra ……………………….………... S60–S115
References ………………………………………..…..………………….….. S116

1. Materials and methods

Reagents and solvents were purchased from various commercial sources (Sigma Aldrich, POCH) and were used without further purification. High-performance liquid chromatography (HPLC)-grade solvents were purchased from POCH (Poland); diethylamine (99+%, extra pure) was purchased from Across Organics; Cat. No: 149450010. β-Nicotinamide adenine dinucleotide, disodium salt, hydrate, 95+%, reduced form (NADH) was purchased from Across Organics; Cat. No.: 271100010. Commercial-grade lyophilized powders of lipase from *Candida antarctica B* (CAL-B) {Novozym 435 – immobilized on the macroporous acrylic resin [poly (methyl methacrylate-co-butyl methacrylate)], specified activity: >10000 U/g or 10 PLU/mg, water content 1.4%, and Lipozyme 435 – immobilized on Lewatit VP OC 1600, both purchased from Novozymes A/S (Bagsvaerd, Denmark); and Chirazyme L-2, c.-f., C2, Lyo. – carrier-fixed on (carrier 2), specified activity: 150 kU, and Chirazyme L-2, c.-f., C3, Lyo. – carrier-fixed on (carrier 3), specified activity: 150 kU, both enzymes purchased from Roche Diagnostics (Mannheim, Germany)}, lipase from *Burkholderia* (formerly *Pseudomonas*) *cepacia* [Amano PS – native lipase, specified activity: >23.000 U/g, purchased from Amano Pharmaceutical Co., Ltd. (Japan); Amano PS-Immobead 150 – immobilized on Immobead 150 particles, specified activity: 900 U/g, purchased from Sigma (cat. nr.: 54327); Amano PS-IM – immobilized on diatomite, specified activity: 500 U/g, purchased from Sigma (cat. nr.: 709603); Amano PS-C II – immobilized on ceramic, purchased from Amano Pharmaceutical Co., Ltd. (Japan)], lipase from *Pseudomonas fluorescens* [Amano AK – native lipase, specified activity: >20.000 U/g, purchased from Amano Pharmaceutical Co., Ltd. (Japan)], lipase from *Mucor javanicus* [Amano Lipase M – native lipase, specified activity: >10.000 U/g, purchased from Sigma (cat. nr.: 534803)], lipase from *Thermomyces lanuginosus* [Lipase, immobilized on Immobead 150 from *Thermomyces lanuginosus* (*Thermomyces lanuginosus*-Immobead 150), specified activity: ≥3000 U/g, purchased from Sigma (cat. nr.: 76546), and Lipozyme TL IM – immobilized on silica gel (a silica granulated), specified activity: 170 IUN/g, purchased from Novozymes (Bagsvaerd, Denmark)], lipase from *Rhizomucor miehei* [Lipozyme RM IM – commercially immobilized, specified activity: 150 IU/g, purchased from Novozymes A/S (Bagsvaerds, Denmark)], lipase from *Alcaligenes sp.* [Chirazyme L-10 – purchased from Boehringer], lipase from *Candida rugosa* [Lipase AYS Amano (‘Amano’ 30) – specified activity: >30 000 U/g, purchased from Amano

Pharmaceutical Co., Ltd.], esterase from porcine liver [PLE – native enzyme, lyophilized powder, specified activity: ≥15 units/mg solid, purchased from Sigma (cat. nr.: E3019)]. All commercial formulations of enzymes studied herein were used without any pre-treatment. Wild-type microorganisms, that are: *Komagataella phaffi*/*Pichia pastoris* (ATCC 76273), *Pseudomonas* sp. (DSM 6978), *Arthrobacter* sp. (DSM 7325), isolate *Actinomyces* sp. SRB-AN040 (FCC025), isolate *Actinomyces* sp. SRB-AN053 (FCC027), isolate *Actinomyces* sp. ARG-AN024 (FCC014), isolate ARG-AN025 (FCC015), and isolate USA-AN012 (FCC021), have been prepared under standard cultivation conditions and later lyophilized as appropriate. The following recombinant alcohol dehydrogenases (ADHs) overexpressed in *E. coli* cells and later lyophilized were prepared as in the given literature references: *Ralstonia* sp. (*E. coli*/RasADH[1]), *Sphingobium yanoikuyae* (*E. coli*/SyADH[2]), *Rhodococcus ruber* (*E. coli*/ADH-A[3]), *Lactobacillus brevis* (*E. coli*/LB-ADH[4]), *Lactobacillus kefir* (*E. coli*/Lk-ADH-Lica,[5] *E. coli*/Lk-ADH,[6] *E. coli*/Lk-ADH Prince[7]). Analytical scale enzymatic reactions were performed in thermo-stated glass vials (V = 4 mL) placed in Chemglass CG-1991-04 GOD Anodized Aluminum Reaction Block, 48 Position, 19 mm Hole Depth, For Circular Top Hot Plate Stirrer. Melting points, uncorrected, were determined with a commercial apparatus on samples contained in rotating capillary glass tubes open on one side (1.35 mm inner diam. and 80 mm length). Analytical thin-layer chromatography was carried on TLC aluminum plates (Merck) covered with silica gel of 0.2 mm thickness film containing a fluorescence indicator green 254 nm (F_{254}), and using either of the visualizing agents such as shortwave UV light (254 nm), iodine, or ninhydrin with heat as developing agents. Preparative separations were carried out by column chromatography using thick-walled glass columns and silica gel (230–400 mesh) with grain size 40–63 μm. The chromatographic analyses (GC) were performed with an Agilent Technologies 6850 instrument equipped with a flame ionization detector (FID) and fitted with HP-50+ (30 m) semipolar column (50 % phenyl–50 % methylpolysiloxane); Helium (2 mL/min) was used as carrier gas; retention times (t_R) are given in minutes under these conditions. The enantiomeric excesses (% ee) of kinetic resolution products were determined by HPLC analysis performed on Shimadzu CTO-10ASV chromatograph equipped with STD-20A UV detector and/or Shimadzu LC-40 Nexera equipped with a photodiode array detector (PAD) and/or Shimadzu Nexera-*i* (LC-2040C 3D) equipped with a photodiode array detector (PAD) and Chiralcel OD-H chiral column packed with cellulose tris (3,5-

dimethylphenylcarbamate) coated on 5μm silica-gel (4.6 mm × 250 mm, from Diacel Chemical Ind., Ltd.) or Chiralpak AD-H chiral column packed with amylose tris (3,5-dimethylphenylcarbamate) coated on 5μm silica-gel (4.6 mm × 250 mm, from Diacel Chemical Ind., Ltd.) and equipped with dedicated pre-columns (4 mm × 10 mm, 5 m particle size) using mixtures of *n*-hexane/*i*-PrOH or *n*-hexane/EtOH/DEA as mobile phase in the appropriate ratios given in experimental section [both the mobile phase composition as well as the flow rate were fine-tuned for each analysis (see **Table S4**)]; the wavelength of UV detection was set at 254 nm (for β-blocker precursors) or 232 nm (for β-blockers), respectively; the HPLC analyses were executed in isothermal (30 °C) manner. Optical rotations ([α]) were measured with a PolAAr 32 polarimeter in a 2 dm long cuvette using the sodium D line (λ = 589 nm); the units of the specific rotation are (deg × mL)/(g × dm). ^{1}H NMR (500 MHz) and ^{13}C NMR (126 MHz) spectra were recorded on a Varian NMR System 500 MHz spectrometer; ^{1}H and ^{13}C chemical shifts (δ) are reported in parts per million (ppm) relative to the solvent signals {$CDCl_3$, δ_H (residual $CHCl_3$) 7.26 ppm, δ_C 77.16 ppm and/or DMSO-d_6, δ_H [residual $(CD_3)_2SO$] 2.49 ppm with HDO at 3.30 ppm, δ_C 40.45 ppm}. Chemical shifts are quoted as s (singlet), d (doublet), dd (doublet of doublets), t (triplet), q (quartet), m (multiplet), and br. s. (broad singlet); coupling constants (*J*) are reported in Hertz. Mass spectrometry was recorded on Micro-mass ESI Q-TOF spectrometer with MSI concept 1H (EI, 70eV ionization) for MS analysis and on Q Exactive Hybrid Quadrupole-Orbitrap Mass Spectrometer, ESI source: electrospray with spray voltage 4.00 kV for FTMS analysis; all samples were prepared by dilution of MeOH (0.5 mL) and additives of mixtures of CH_3CN/MeOH/H_2O (50:25:25, v/v/v) + 0.5% formic acid each.

2. Synthetic procedures

2.1. General procedure for the synthesis of 2-(oxiran-2-ylmethyl)-1*H*-isoindole-1,3(2*H*)-dione (*rac*-3)

Potassium phthalimide **1** (10 g, 53.99 mmol) was suspended in epichlorohydrin *rac*-**2** (35.5 g, 0.38 mol, 30 mL) and stirred for 24 h at 120 °C. The excess of epichlorohydrin was removed under reduced pressure using a rotary evaporator, and the resulting yellowish solid was suspended in MeOH (50 mL) and refluxed for 15 min. After this time, the undissolved cream solid was filtered off, and the permeate was concentrated

by half of the volume and placed in the fridge until white solid precipitated. After filtration of the solid, the crude product was additionally treated with $CHCl_3$ (25 mL), the undissolved solid was filtered off, the filtrate was concentrated, and the formed solid was recrystallized from MeOH (25 mL) to receive the desired phthalimide epoxide *rac*-**3** (6.68 g, 32.88 mmol, 61%) as a white powder.

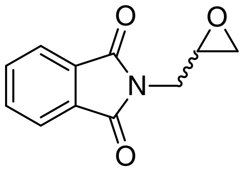

Mp 94–96 °C (MeOH) [lit.[8] 95–97 °C (MeOH)]; R_f [$CHCl_3$/acetone (95:5, v/v)] 0.82; ^{1}H NMR (500 MHz, $CDCl_3$): δ 2.68 (dd, *J*=4.9, 2.5 Hz, 1H), 2.75–2.90 (m, 1H), 3.24 (tdd, *J*=5.0, 5.0, 3.9, 2.5 Hz, 1H), 3.72–3.85 (m, 1H), 3.91–4.04 (m, 1H), 7.62–7.79 (m, 2H), 7.81–7.96 (m, 2H); ^{13}C NMR (126 MHz, $CDCl_3$): δ 39.7, 46.1, 49.1, 123.4, 131.9, 134.1, 168.0; IR (nujol): ν_{max} = 3440, 1772, 1712, 1606, 1430, 1395, 1349, 1306, 1254, 1193,1170, 1153, 1138, 1078, 1044, 983, 964, 900, 848, 824, 788; FTMS (ESI-TOF) *m/z*: $[M+H]^+$ Calcd for $C_{11}H_{10}NO_3^+$ m/z: 204.0655, Found 204.0655; GC [220–260 (10 °C/min)]: t_R = 2.823 min; HPLC [*n*-hexane-*i*-PrOH (90:10, v/v); f = 0.8 mL/min; λ=254 nm (Chiralcel OD-H)]: t_R = 18.340 min (*R*-isomer) and 19.546 min (*S*-isomer).

2.2. General procedure for the synthesis of 2-(3-chloro-2-hydroxypropyl)-1*H*-isoindole-1,3(2*H*)-dione (*rac*-4)

To a solution of 2,3-epoxypropylphthalimide *rac*-**3** (8.2 g, 40.36 mmol) in $CHCl_3$ (200 mL) cooled to 0–5 °C, a 36% HCl (60 mL) was added dropwise with stirring. The reaction mixture was stirred for 30 min at 0–5 °C, and after that, a solution was washed with brine (2 × 175 mL), and back-extracted with CH_2Cl_2 (3 × 150 mL). The combined organic layers were dried over anhydrous Na_2SO_4, and after filtration of the drying agent and removal of the organic solvents *in vacuo* pure 3-chloro-1-phthalimidopropan-2-one *rac*-**4** (8.40 g, 35.05 mmol, 87%) was obtained as a white powder.

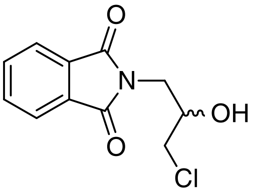

Mp 90–91 °C (CH_2Cl_2) [lit.[9] 91–92 °C (Et_2O/hexane)]; R_f [$CHCl_3$/acetone (95:5, v/v)] 0.55; ^{1}H NMR (500 MHz, $CDCl_3$): δ 2.99 (br. s., 1H), 3.52–3.74 (m, 2H), 3.79–4.01 (m, 2H), 4.09–4.25 (m, 1H), 7.64–7.79 (m, 2H), 7.79–7.98 (m, 2H); ^{13}C NMR (126 MHz, $CDCl_3$): δ 41.6, 47.2, 69.7, 123.5, 131.8, 134.2, 168.6; IR (nujol): ν_{max} = 3464, 1772, 1692, 1608, 1439, 1400, 1308, 1236, 1213, 1178, 1130, 1080, 1040, 981, 956,

872, 844, 796, 720, 707, 691, 641; FTMS (ESI-TOF) *m/z*: $[M+H]^+$ Calcd for $C_{11}H_{11}ClNO_3^+$ m/z: 240.0422, Found 240.0422; GC [220–260 (10 °C/min)]: t_R = 4.267 min; HPLC [*n*-hexane-*i*-PrOH (90:10, v/v); f = 0.8 mL/min; λ=254 nm (Chiralcel OD-H)]: t_R = 23.187 min (*S*-isomer) and 31.089 min (*R*-isomer) or t_R = 25.341 min (*S*-isomer) and 34.474 min (*R*-isomer).

2.3. General procedure for the synthesis of racemic esters *rac*-5a–b

To a solution of the 2-(3-chloro-2-hydroxypropyl)-1*H*-isoindole-1,3(2*H*)-dione *rac*-**4** (500 mg, 2.09 mmol) in dry CH_2Cl_2 (5 mL), Et_3N (317 mg, 3.13 mmol, 0.38 mL, 1.5 equiv) and DMAP (15 mg, 0.12 mmol) were added. The mixture was cooled to 0–5 °C in an ice bath. Next, the solution of the appropriate acyl chloride (1.5 equiv) in dry CH_2Cl_2 (2 mL) was added dropwise to the reaction mixture by using a syringe. Afterward, the cooling bath was removed, and the resulting mixture was stirred at room temperature for 12 h. The crude mixture was diluted with CH_2Cl_2 (10 mL), subsequently quenched with H_2O (20 mL), the water phase was extracted with CH_2Cl_2 (3 × 10 mL), and the combined organic layer was washed with saturated water solution of $NaHCO_3$ (40 mL), brine (40 mL), and dried over anhydrous $MgSO_4$. After evaporation of the residuals of solvent under reduced pressure, the crude product was purified by double column chromatography on silica gel using a mixture of $CHCl_3$/acetone (95:5, v/v) for *rac*-**5a** and $CHCl_3$/acetone (98:2, v/v) for *rac*-**5b** as eluent, respectively, thus obtaining desired esters as a white solid (in the case of *rac*-**5a**) or yellowish oil (in the case of *rac*-**5b**).

1-Chloro-3-(1,3-dioxo-1,3-dihydro-2*H*-isoindol-2-yl)propan-2-yl acetate (*rac*-5a).

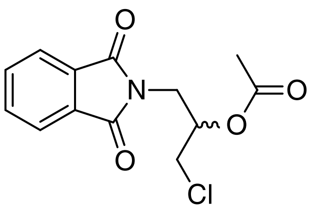

Yield 64% (373 mg); mp 71–74 °C ($CHCl_3$/acetone) [lit.[10] 96–97 °C (CH_2Cl_2)]; R_f [$CHCl_3$/acetone (95:5, v/v)] 0.87; ^1H NMR (500 MHz, $CDCl_3$): δ 2.04 (s, 3H), 3.61 (dd, *J*=12.0, 6.1 Hz, 1H), 3.73 (dd, *J*=12.0, 4.7 Hz, 1H), 3.95–4.03 (m, 2H), 5.26–5.34 (m, 1H), 7.68–7.78 (m, 2H), 7.81–7.90 (m, 2H); ^{13}C NMR (126 MHz, $CDCl_3$): δ 20.8, 38.9, 43.5, 70.9, 123.5, 131.8, 134.2, 168.0, 170.2; IR (nujol): ν_{max} = 3637, 3481, 3071, 3030, 2948, 1779, 1751, 1724, 1611, 1506, 1468, 1423, 1396, 1375, 1316, 1264, 1228, 1191, 1172, 1153, 1121, 1089, 1044, 940, 914, 896, 796, 756, 724; FTMS (ESI-TOF) *m/z*: $[M+H]^+$ Calcd for $C_{13}H_{13}ClNO_4^+$ m/z: 282.0528, Found 282.0528; GC [220–260 (10 °C/min)]: t_R = 4.606 min; HPLC [*n*-hexane-*i*-PrOH

(90:10, v/v); f = 0.8 mL/min; λ = 254 nm (Chiralcel OD-H)]: t_R = 21.263 min (*S*-isomer) and 23.011 min (*R*-isomer).

1-Chloro-3-(1,3-dioxo-1,3-dihydro-2*H*-isoindol-2-yl)propan-2-yl butanoate (*rac*-5b).

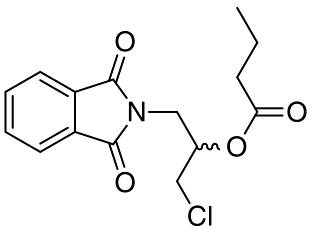

Yield 48% (308 mg); yellowish oil; R_f [$CHCl_3$/acetone (98:2, v/v)] 0.80; ^1H NMR (500 MHz, $CDCl_3$): δ 0.86 (t, *J*=7.4, 3H), 1.49–1.67 (m, 2H), 2.27 (t, *J*=7.4, 2H), 3.62 (dd, *J*=12.0, 6.4 Hz, 1H), 3.73 (dd, *J*=12.0, 4.4 Hz, 1H), 3.90–4.06 (m, 2H), 5.33 (tt, *J*=6.30, 4.34 Hz, 1H), 7.66–7.78 (m, 2H), 7.80–7.91 (m, 2H); ^{13}C NMR (126 MHz, $CDCl_3$): δ 13.5, 18.1, 35.9, 39.1, 43.6, 70.5, 123.5, 131.8, 134.2, 168.0, 172.8; IR (nujol): ν_{max} = 3473, 3024, 2968, 2939, 2879, 1776, 1724, 1613, 1468, 1430, 1396, 1313, 1247, 1172, 1088, 1038, 1010, 940, 915, 905, 795, 756, 724; FTMS (ESI-TOF) *m/z*: $[M+H]^+$ Calcd for $C_{15}H_{17}ClNO_4^+$ m/z: 310.0841, Found 310.0841; HPLC: Not resolvable on available chiral column.

2.4. General procedure for hydrolytic EKR of racemic acetate *rac*-5a – *enzyme screening*

To a solution of *rac*-**5a** (50 mg, 0.18 mmol) in CH_3CN (100 μL) and 0.1 M K_2HPO_4 buffer (1 mL, pH 7.5), the respective enzyme preparation [25 mg (50% wt/wt with regards to substrate *rac*-**5a** for solid enzymes) or 200 μL (ca. 20% v/v with regards to a reaction medium for liquid enzymes) were added at one portion. The reaction mixture was stirred (500 rpm, IKA RCT basic) in a thermo-stated glass vial (*V* = 4 mL) at 40 °C for 72 h. After this time, the content of the vial was extracted with $CHCl_3$ (3 × 1.5 mL), the combined organic layer was dried over anhydrous $MgSO_4$, the drying agent was filtered off, and the remaining permeate was concentrated under a vacuum. Next, the oil residue was purified by column chromatography on silica gel using a mixture of $CHCl_3$/acetone (98:2, 95:5 v/v) as the eluent, thus affording the respective resolution products [alcohol (*S*)-(–)-**4** and the acetate (*R*)-(–)-**5a**]. Next, to obtain necessary information concerning the values of %-conversion, enantiomeric excess (% ee), and enantioselectivity factor (*E*), the HPLC analyses were performed for both EKR products. The results of hydrolytic EKR are collected in **Table S1**. Screening conditions for co-solvent were performed in analogy to this procedure, and the results are shown in **Table S2**.

2.5. General procedure for analytical-scale lipase-catalyzed KR of *rac*-4 – *enzyme screening*

In a typical enzymatic procedure, racemic chlorohydrin *rac*-**4** (50 mg, 0.21 mmol) was dissolved in TBME (1 mL). Subsequently, vinyl acetate (54 mg, 0.63 mmol, 58 μL) and the respective lipase preparation (25 mg, 50%, w/w ratio to substrate *rac*-**4**) were added at once. The thus composed reaction mixture was stirred (800 rpm) in a thermo-stated glass vial (V = 4 mL), which was placed in an anodized aluminum reaction block heated at 30 °C. The progress of the EKR process was monitored by GC analysis until the required conversion was achieved (ca. 50-65%) depending on the biocatalytic-reaction system used. The samples were prepared by withdrawing the suspension (50 μL) from the reaction mixture, diluting it with a portion of TBME (2 mL), and centrifugation the enzyme using a laboratory centrifuge (6000 rpm). A small amount taken from the supernatant was diluted with AcOEt (1 mL) and subjected directly onto GC column to analyze the % conversion. If the conversion achieved an appropriate value, the reaction was then terminated by enzyme filtration on a Schott funnel under vacuum, washing it with TBME (2 mL), and evaporating the volatiles using rotavap. Both the remaining chlorohydrin (*R*)-(+)-**4** and the respective acetate (*S*)-(+)-**5a** present in the crude residue were separated by column chromatography on SiO_2 gel using a mixture of $CHCl_3$/acetone (95:5, v/v), thus obtaining desired optically active products, which enantiomeric purity was characterized by HPLC equipped with a chiral column. In this regard, the appropriate samples were prepared as follows: after evaporation of the volatiles, the crude oil (2-4 mg) was dissolved in a mixture of *n*-hexane/2-PrOH (1.5 mL, 3:1, v/v) and analyzed. For additional data, see **Table 1** in the main manuscript.

2.6. General procedure for analytical-scale (Amano PS-IM)-catalyzed KR of *rac*-4 – *co-solvent screening*

The reaction mixture containing *rac*-**4** (50 mg, 0.21 mmol), the appropriate organic solvent (1 mL), vinyl acetate (54 mg, 0.63 mmol, 58 μL), and Amano PS-IM lipase (25 mg, 50%, w/w ratio to substrate *rac*-**4**) was stirred (800 rpm, IKA RCT basic) in a thermo-stated glass vial (V = 4 mL) at 30 °C. Further manipulations were carried out by analogy with the previous procedure reported for the enzyme screening (see section *enzyme screening* above). For additional data, see **Table 2** in the main manuscript.

2.7. General procedure for analytical-scale (Amano PS-IM)-catalyzed KR of *rac*-4 – *temperature effect*

The reaction mixture containing *rac*-**4** (50 mg, 0.21 mmol), TBME (1 mL), vinyl acetate (54 mg, 0.63 mmol, 58 μL) and Amano PS-IM lipase (25 mg, 50%, w/w ratio to substrate *rac*-**4**) was stirred (800 rpm, IKA RCT basic) independently in a thermostated glass vial ($V = 4$ mL) at 30 °C, 40 °C and 50 °C. The reactions conducted at 50 °C were carried out for different time intervals, terminating after 2 h, 4 h, 6 h, 8 h, 16 h, and 20 h. Further manipulations were carried out by analogy with the previous procedures reported for the enzyme screening and co-solvent screening (see section *enzyme screening* and *co-solvent screening* above), respectively. For additional data, see **Table 3** and **Figure 3** in the main manuscript.

2.8. General procedure for 2.5 gram-scale (Amano PS-IM)-catalyzed KR of *rac*-4

To a solution of racemic *N*-protected amino chlorohydrin *rac*-**4** (2.5 g, 10.43 mmol) in TBME (50 mL), vinyl acetate (2.7 g, 31.29 mmol, 2.88 mL) and Amano PS-IM lipase [1.25 g, 50% w/w (catalyst/substrate *rac*-**4**)] were added at one portion. The reaction mixture was stirred (800 rpm, IKA RCT basic) in a round-bottomed flask (100 mL) equipped with a Teflon-coated magnetic stir bar (2 cm × 5 mm, 2 g) at 50 °C until the required 57% conversion was achieved (ca. 23 h). Next, the enzyme was removed by filtration and washed with TBME (2 × 50 mL). The filtrate was condensed to 50 mL volume, and the precipitated solid was filtered off, washed with a portion of TBME (10 mL), and dried under vacuum to yield the first crop of optically active chlorohydrin (*R*)-(+)-**4** (830 mg). The second crop of (*R*)-(+)-**4** (170 mg) was obtained after cooling the solution in the fridge for a few hours. Next, the volatiles were evaporated from the permeate under reduced pressure, and the crude residue was purified by column chromatography on SiO_2 (150 g of silica gel was taken) using gradient of $CHCl_3$/acetone (98:2, 95:5 v/v) mixture as an eluent, thus affording enantioenriched 2-[(2*R*)-2-hydroxypropyl]-1*H*-isoindole-1,3(2*H*)-dione {(*R*)-(+)-**4**, 1.01 g, 4.21 mmol, 40% isolated yield, >99% ee, $[\alpha]_D^{29} = +30.00$ (*c* 1.00, EtOH); lit.[11] $[\alpha]_D^{20} = -14.47$ (*c* 0.48, EtOH) for (*S*)-**4** obtained in 95% ee} and (2*S*)-1-(1,3-dioxo-1,3-dihydro-2*H*-isoindol-2-yl)propan-2-yl acetate [(*S*)-(+)-**5a**, 1.41 g, 5.00 mmol, 48% isolated yield, 74% ee]. For additional data, see **Table 4** in the main manuscript.

2.9. General procedure for 5 gram-scale (Amano PS-IM)-catalyzed KR of *rac*-4

To a solution of *rac*-**4** (5 g, 20.86 mmol) in TBME (100 mL), vinyl acetate (5.4 g, 62.59 mmol, 5.77 mL) and Amano PS-IM lipase [1.25 g, 25% w/w (catalyst/substrate *rac*-**4**)] were added at one portion. The reaction mixture was stirred (800 rpm, IKA RCT basic) in a round-bottomed flask (250 mL) equipped with a Teflon-coated magnetic stir bar (2.5 cm × 1 cm, 6.6 g) at 50 °C for 24 h (until 57% conv. was reached). Next, the enzyme was removed by filtration and washed with TBME (2 × 50 mL). The volatiles were evaporated from the permeate under reduced pressure, and the crude residue was purified by column chromatography on SiO_2 (400 g of silica gel was taken) using gradient of $PhCH_3$/acetone (50:1, 25:1, 15:1 v/v) mixture as an eluent, thus affording enantioenriched 2-[(2*R*)-2-hydroxypropyl]-1*H*-isoindole-1,3(2*H*)-dione [(*R*)-(+)-**4**, 1.96 g, 8.18 mmol, 39% isolated yield, >99% ee] and (2*S*)-1-(1,3-dioxo-1,3-dihydro-2*H*-isoindol-2-yl)propan-2-yl acetate [(*S*)-(+)-**5a**, 3.21 g, 11.40 mmol, 55% isolated yield, 73% ee, $[\alpha]_D^{27}$ = +20.50 (*c* 1.00, $CHCl_3$)]. Attention: the optically active ester (*S*)-(+)-**5a** was isolated as a white solid with mp 90–92 °C ($CHCl_3$/acetone). For additional data, see **Table 4** in the main manuscript.

2.10. General procedure for the synthesis of racemic nitrobenzoates ('*Mitsunobu esters*') *rac*-10–11

To a mixture of racemic chlorohydrin *rac*-**4** (100 mg, 0.42 mmol), 4-nitrobenzoic acid (70 mg, 0.42 mmol) or 2,4-dinitrobenzoic acid (89 mg, 0.42 mmol), and DMAP (20 mg, 0.17 mmol) in CH_2Cl_2 (3 mL), EDCI hydrochloride (88 mg, 0.46 mmol) was added in one portion at room temperature. Next, the reaction mixture was stirred at 30 °C for 3 h. After this time, the content of the flask was diluted with CH_2Cl_2 (10 mL, washed with H_2O (4 × 10 mL), and the aqueous layer was back-extracted with CH_2Cl_2 (2 × 20 mL). The combined organic phases were rewashed with H_2O (30 mL) and brine (30 mL), dried over anhydrous $MgSO_4$, filtered, and permeate was concentrated *in vacuo*. The residue was purified by silica gel chromatography using mixture of $CHCl_3$/acetone (98:2, v/v) to provide the corresponding esters: 1-chloro-3-(1,3-dioxo-1,3-dihydro-2*H*-isoindol-2-yl)propan-2-yl 4-nitrobenzoate (*rac*-**10**, 134 mg, 0.35 mmol, 83%) and 1-chloro-3-(1,3-dioxo-1,3-dihydro-2*H*-isoindol-2-yl)propan-2-yl 2,4-dinitrobenzoate (*rac*-**11**, 156 mg, 0.36 mmol, 86%).

1-Chloro-3-(1,3-dioxo-1,3-dihydro-2*H*-isoindol-2-yl)propan-2-yl 4-nitrobenzoate (*rac*-10).

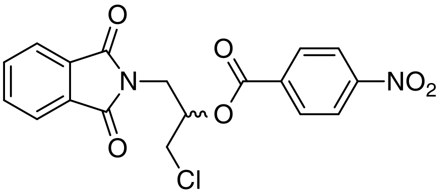

Mp 121–123 °C ($CHCl_3$/acetone) for *rac*-**10** and mp 89–91 °C (hexane/acetone) for (*S*)-**10**; R_f [$CHCl_3$/acetone (98:2, v/v)] 0.69 or R_f [$PhCH_3$/acetone (15:1, v/v)] 0.58 or R_f [hexane/acetone (4:1, v/v)] 0.16; ^{1}H NMR (500 MHz, $CDCl_3$): δ 3.78–3.85 (m, 1H), 3.88–3.95 (m, 1H), 4.10 (dd, *J*=14.4, 3.7 Hz, 1H), 4.21–4.29 (m, 1H), 5.53–5.59 (m, 1H), 7.70–7.77 (m, 2H), 7.80–7.89 (m, 2H), 8.15–8.21 (m, 2H), 8.24–8.31 (m, 2H); ^{13}C NMR (126 MHz, $CDCl_3$): δ 39.0, 43.3, 72.4, 123.6, 131.0, 131.7, 134.3 (2C), 134.7, 150.7, 164.0, 167.9 (2C); IR (nujol): ν_{max} = 2928, 1720, 1460, 716; FTMS (ESI-TOF) *m/z*: $[M+H]^+$ Calcd for $C_{18}H_{14}ClN_2O_6^+$ m/z: 389.0535, Found 389.0533; UV/VIS: λ_{max} = 219 nm (EtOH); HPLC [*n*-hexane-*i*-PrOH (90:10, v/v); f = 0.8 mL/min; λ = 219 nm (Chiralcel OD-H)]: t_R = 69.254 min (*S*-isomer) and 74.709 min (*R*-isomer).

1-Chloro-3-(1,3-dioxo-1,3-dihydro-2*H*-isoindol-2-yl)propan-2-yl 2,4-dinitrobenzoate (*rac*-11).

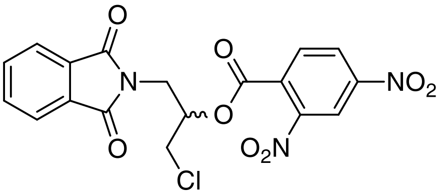

Mp 138–140 °C ($CHCl_3$/acetone); R_f [$CHCl_3$ (100)] 0.67; ^{1}H NMR (500 MHz, $CDCl_3$): δ 3.76 (dd, *J*=12.2, 5.6 Hz, 1H), 3.83–3.91 (m, 1H), 4.07–4.14 (m, 1H), 4.16–4.24 (m, 1H), 5.57–5.67 (m, 1H), 7.73–7.79 (m, 2H), 7.84–7.90 (m, 2H), 7.99–8.05 (m, 1H), 8.53–8.59 (m, 1H), 8.78–8.82 (m, 1H); ^{13}C NMR (126 MHz, $CDCl_3$): δ 39.0, 43.1, 74.1, 119.8, 123.8, 128.0, 131.5, 131.9, 132.7, 134.5, 147.6, 149.1, 163.4, 168.3; IR (nujol): ν_{max} = 1716, 724; FTMS (ESI-TOF) *m/z*: $[M+H]^+$ Calcd for $C_{18}H_{13}ClN_3O_8^+$ m/z: 434.0386, Found 434.0385; HPLC [*n*-hexane-*i*-PrOH (78:22, v/v); f = 1.0 mL/min; λ = 217 nm (Chiralpak AD-H)]: t_R = 54.362 min (*R*-isomer) and 60.823 min (*S*-isomer).

2.11. General procedure for the inversion of the absolute configuration in (*R*)-(+)-4 using Mitsunobu reaction

2.11.1. Method A: Diethyl azodicarboxylate (DEAD, 153 mg, 0.63 mmol, 104 µL, as 40% toluene solution) diluted in anhydrous THF (1.5 mL) was added dropwise at 0–5

°C to a stirred solution of Ph_3P (164 mg, 0.63 mmol), 4-nitrobenzoic acid (105 mg, 0.63 mmol), and optically active chlorohydrin (*R*)-(+)-**4** (100 mg, 0.42 mmol, >99% ee) in THF (2 mL). The vigorous stirring at 25 °C was continued for 12 h to ensure complete conversion of the starting material according to TLC analysis [$CHCl_3$/acetone (98:2, v/v) or $PhCH_3$/acetone (15:1, v/v)]. Afterward, the residual volatile was removed under reduced pressure, and the crude product was purified by silica-gel column chromatography eluting with a gradient of hexane/acetone (4:1, 3:1 v/v) mixture to give (*S*)-**10** (72 mg, 0.18 mmol, 44% isolated yield, 18% ee) as white solid.

2.11.2. Method B: A suspension of optically active chlorohydrin (*R*)-(+)-**4** (239 mg, 1.00 mmol, >99% ee), 2,4-dinitrobenzoic acid (212 mg, 1.00 mmol), and the catalytic amount of (2-hydroxybenzyl)diphenylphosphine oxide (62 mg, 0.20 mmol) in xylene (12.5 mL) was heated to reflux in a Dean-Stark apparatus and stirred for 48 h. Next, the reaction mixture was cooled to room temperature, diluted with EtOAc (30 mL), washed with 1 M NaOH (aq) solution (2 × 20 mL), and then with brine (20 mL). The combined organic phase was dried over anhydrous $MgSO_4$, filtered, and concentrated *in vacuo*. The crude residue was purified by silica-gel column chromatography eluting with $CHCl_3$ (100%) to give (*S*)-**11** (25 mg, 0.06 mmol, 6% isolated yield, 81% ee) as a white solid.

2.12. General procedure for the inversion of the absolute configuration in (*R*)-(+)-4 using 'AcOCs-based strategy' (see Steps 1–3 below)

2.12.1. Step 1 [Synthesis of (2*R*)-1-chloro-3-(1,3-dioxo-1,3-dihydro-2*H*-isoindol-2-yl)propan-2-yl methanesulfonate (*R*)-(+)-9]: To a stirred solution of chlorohydrin (*R*)-(+)-**4** (1 g, 4.18 mmol, >99% ee) in dry CH_2Cl_2 (20 mL) cooled to 0–5 °C were added methanesulfonyl chloride (716 mg, 6.26 mmol, 484 μL) and Et_3N (634 mg, 6.26 mmol, 764 μL) in one portion. After 10 min of stirring, the reaction mixture was warmed to room temperature and stirred for an additional 1 h. Afterward, the reaction mixture was diluted with CH_2Cl_2 (40 mL), and the organic phase was washed with saturated $NaHCO_3$ (3 × 60 mL) and back-extracted with CH_2Cl_2 (3 × 60 mL). The combined organic layer was quenched with brine (60 mL), dried over anhydrous $MgSO_4$, concentrated, and the residue was purified by silica gel column

chromatography using $CHCl_3$/acetone (95:5, v/v) as eluent to afford corresponding optically active mesylate (*R*)-(+)-**9** {1.07 g, 3.37 mmol, 81%, >99% ee, $[\alpha]_D^{33}$ = +15.31 (*c* 0.98, $CHCl_3$)} as white solid.

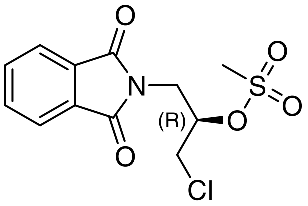

Mp 114–116 °C ($CHCl_3$/acetone); R_f [$CHCl_3$/acetone (95:5, v/v)] 0.78; ^{1}H NMR (500 MHz, $CDCl_3$): δ 3.04 (s, 3H), 3.71–3.78 (m, 1H), 3.79–3.86 (m, 1H), 3.95 (dd, *J*=14.4, 3.7 Hz, 1H), 4.14 (dd, *J*=14.7, 7.8 Hz, 1H), 5.12–5.21 (m, 1H), 7.71–7.77 (m, 2H), 7.85–7.91 (m, 2H); ^{13}C NMR (126 MHz, $CDCl_3$): δ 38.4, 39.5, 43.7, 76.5, 123.5, 131.8, 134.4, 167.9; IR (nujol): ν_{max} = 2932, 1772, 1708, 1464, 1400, 1376, 1176, 1032, 1000, 972, 936, 896, 796, 724; FTMS (ESI-TOF) *m/z*: $[M+H]^+$ Calcd for $C_{12}H_{13}ClNO_5S^+$ m/z: 318.0198, Found 318.0201; GC [220–260 (10 °C/min)]: t_R = 11.247 min; UV/VIS: λ_{max} = 219 nm (EtOH); HPLC [*n*-hexane-*i*-PrOH (85:15, v/v); f = 0.8 mL/min; λ = 219 nm (Chiralcel OD-H)]: t_R = 66.959 min (*S*-isomer) and 77.314 min (*R*-isomer) or HPLC [*n*-hexane-*i*-PrOH (90:10, v/v); f = 1.0 mL/min; λ = 216 nm (Chiralpak AD-H)]: t_R = 47.821 min (*S*-isomer) and 50.660 min (*R*-isomer).

2.12.2. Step 2 [Synthesis of acetate (*S*)-(+)-5a from mesylate (*R*)-(+)-9]:

Step 2a (50 mg-scale): The optically active mesylate (*R*)-(+)-**9** (50 mg, 0.16 mmol, >99% ee) was dissolved in dry $PhCH_3$ (10 mL). Then AcOCs (151 mg, 0.63 mmol) and a catalytic amount of 18-Crown-6 (5 mg) were added in one portion under a protective atmosphere of argon. The mixture was stirred for 120 h at 110 °C and afterward poured into H_2O (15 mL). This mixture was extracted with AcOEt (3 × 15 mL). The collected organic phases were dried over anhydrous $MgSO_4$, and the solvent was evaporated. The crude product was purified by column chromatography on silica gel using $CHCl_3$ (100%) as an eluent to afford optically active acetate (*S*)-(+)-**5a** (15 mg, 53.3 μmol, 34%, 98% ee) as white solid. Other analyses were consistent with *rac*-**5a**.

Step 2b (500 mg-scale): The optically active mesylate (*R*)-(+)-**9** (500 mg, 1.57 mmol, >99% ee) was dissolved in dry $PhCH_3$ (50 mL). Then AcOCs (3.02 g, 15.74 mmol) and a catalytic amount of 18-Crown-6 (100 mg) were added in one portion under a

protective atmosphere of argon. The mixture was stirred for 120 h at 110 °C and afterward poured into H_2O (150 mL). This mixture was extracted with AcOEt (3 × 150 mL). The collected organic phases were dried over anhydrous $MgSO_4$, and the solvent was evaporated. The crude product was purified by column chromatography on silica gel using $CHCl_3$ (100%) as an eluent to afford optically active acetate (*S*)-(+)-**5a** (159 mg, 0.56 mmol, 36%, >99% ee) as white solid. Other analyses were consistent with *rac*-**5a**.

2.12.3. Step 3 [Hydrolysis of acetate (*S*)-(+)-5a to (*S*)-(–)-4]:

To a solution of optically active acetate (*S*)-(+)-**5a** (200 mg, 0.71 mmol, >99% ee) in MeOH (5 mL) 98% H_2SO_4 (60 μL) was added in one portion. The resulting mixture was stirred for 24 h at 35 °C, and after this time, another portion of conc. H_2SO_4 (200 μL) was added, and the reaction was continued for an additional 72 h until the substrate was completely consumed according to TLC analysis. Next, the volatile was evaporated under vacuum, and the crude oil was subjected to column chromatography and eluted with a mixture of $CHCl_3$/acetone (98:2, v/v), thus affording desired alcohol (*S*)-(–)-**4** (147 mg, 0.61 mmol, 86%, >99% ee).

2.13. General procedure for the synthesis of 2-[(2*R*)-oxiran-2-ylmethyl]-1*H*-isoindole-1,3(2*H*)-dione [(*R*)-(–)-3]

2.13.1. Method A: To the solution of 2-[(2*R*)-2-hydroxypropyl]-1*H*-isoindole-1,3(2*H*)-dione (*R*)-(+)-**4** (50 mg, 0.21 mmol, >99% ee) in $PhCH_3$ (1 mL) anhydrous K_2CO_3 (58 mg, 0.42 mmol) was added in one portion, and the reaction mixture was stirred at reflux for 24 h. Afterward, the content of the flask was filtered through a short Celite pad, the filtrate cake was rinsed with $PhCH_3$ (10 mL), and the filtrate was evaporated to dryness under reduced pressure to yield the desired product (*R*)-(–)-**3** (32 mg, 0.16 mmol, 76%, >99% ee) as white solid [mp 97–100 °C ($PhCH_3$), lit.[12] 98–100 °C (AcOEt)], which was further used without purification.

2.13.2. Method B: To the solution of optically active chlorohydrin (*R*)-(+)-**4** (2 g, 8.35 mmol, >99% ee) in $PhCH_3$ (40 mL) was added anhydrous K_2CO_3 (2.31 g, 16.69 mmol), and the reaction mixture was stirred at reflux for 24 h. Afterward, the content of the flask was filtered through a short pad of Celite. The filtrate cake was

additionally rinsed with $PhCH_3$ (400 mL), and the permeate was evaporated to dryness under reduced pressure in a rotavap. Since traces of unreacted substrate (*R*)-(+)-**4** remained in the crude mixture, the purification procedure was accomplished by column chromatography employing $CHCl_3$/acetone (95:5, v/v) as the eluent to afford the desired optically active epoxide (*R*)-(–)-**3** {841 mg, 4.14 mmol, 50%, >99% ee, $[\alpha]_D^{27}$ = –13.50 (*c* 1.00, $CHCl_3$)} as white solid [mp 99–100 °C ($CHCl_3$/acetone)}. The synthesis of the enantiomeric counterpart was performed on a 0.21 mmol scale in analogy to this procedure, thus affording optically active epoxide (*S*)-(+)-**3** {23 mg, 0.11 mmol, 54%, >99% ee) as white solid as well.

2.14. General procedure for the synthesis of racemic 1-aryloxy-3-phthalimide-2-propanols *rac*-6a–b

2.14.1. Method A: To a solution of racemic 2-(oxiran-2-ylmethyl)-1*H*-isoindole-1,3(2*H*)-dione *rac*-**3** (50 mg, 0.25 mmol) and the appropriate phenolic compound (ArOH, 0.9 equiv) in $PhCH_3$ (30 mL), anhydrous K_2CO_3 (2.7 equiv) was added in one portion, and the thus composed reaction mixture was stirred at reflux for 16 h. Afterward, the content of the flask was filtered through a short Celite pad, the filtrate cake was rinsed with $PhCH_3$ (6 mL), Et_2O (6 mL), AcOEt (6 mL), and acetone (100 mL), and the permeate was evaporated to dryness under reduced pressure to afford an orange oil. After purification of the crude oil residue on silica gel column chromatography using a gradient of a mixture of *n*-hexane/AcOEt (3:1, 1:1, v/v), the respective products *rac*-**6a**–**b** were yielded as solids.

2.14.2. Method B: A mixture of racemic *N*-(2,3-epoxypropyl)-phthalimide *rac*-**3** (50 mg, 0.25 mmol), 1-naphthol (36 mg, 0.25 mmol), xylene (200 µL) and DBU (4 mg, 24.61 µmol, 3.67 µL) was stirred under N_2 at 120° C for 24 h. Next, the crude reaction mixture was condensed under vacuum and purified by column chromatography using a gradient of the mixture of *n*-hexane/AcOEt (3:1, 1:1, v/v) to afford *rac*-**6a**.

2-[2-Hydroxy-3-(naphthalen-1-yloxy)propyl]-1*H*-isoindole-1,3(2*H*)-dione (*rac*-6a).

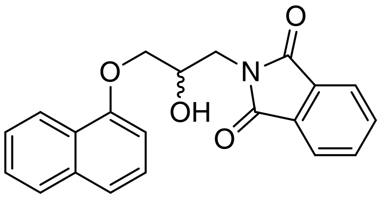

Method A: Yield 71% (61 mg); Method B: Yield 93% (80 mg); yellowish solid; mp 154–156 °C (*n*-hexane/AcOEt) [lit.[13] 152–153 °C (EtOH)]; R_f [*n*-hexane/AcOEt (1:1, v/v)] 0.62; ^{1}H NMR (500 MHz, $CDCl_3$): δ 4.01–4.19 (m, 2H) 4.19–4.34 (m, 2H) 4.37–4.58 (m, 1H) 6.74–6.92 (m, 1H) 7.30–7.57 (m, 4H) 7.62–7.98 (m, 5H) 8.27–8.29 (m, 1H); ^{13}C NMR (126 MHz, $CDCl_3$): δ 41.5, 69.0, 69.9, 105.0, 121.0, 121.7, 123.5, 125.4, 125.7, 126.5, 127.5, 131.9, 134.1, 134.5, 154.0, 168.8; IR (nujol): ν_{max} = 3459, 1764, 1712, 1576, 1509, 1267, 1248, 1124, 1108, 1080, 1054, 930, 793, 777, 714; FTMS (ESI-TOF) *m/z*: $[M+H]^+$ Calcd for $C_{21}H_{18}NO_4^+$ m/z: 348.1231, Found 348.1106.

2-{(2-Hydroxy-3-[2-(prop-2-en-1-yl)phenoxy]propyl}-1*H*-isoindole-1,3(2*H*)-dione (*rac*-6b).

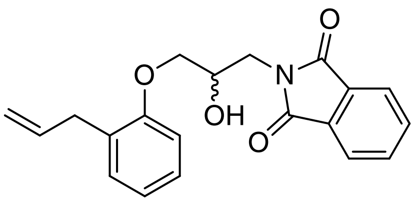

Method A: Yield 24% (20 mg); white solid; mp 84–86 °C (*n*-hexane/AcOEt); R_f [*n*-hexane/AcOEt (1:1, v/v)] 0.51; ^{1}H NMR (500 MHz, $CDCl_3$): δ 1.64 (br. s., 1H), 3.43 (d, *J*=5.4 Hz, 2H), 3.88–4.17 (m, 4H), 4.24–4.37 (m, 1H), 5.00–5.15 (m, 2H), 6.00 (ddt, *J*=17.0, 10.4, 6.4, 6.4 Hz, 1H), 6.82–6.87 (m, 1H), 6.90–6.97 (m, 1H), 7.11–7.23 (m, 2H), 7.68–7.78 (m, 2H), 7.83–7.93 (m, 2H); ^{13}C NMR (126 MHz, $CDCl_3$): δ 34.8, 41.1, 68.8, 69.6, 111.3, 115.3, 121.2, 123.4, 127.5, 128.5, 130.2, 132.0, 134.1, 137.3, 156.1, 168.7; IR (nujol): ν_{max} = 3506, 1771, 1692, 1635, 1600, 1498, 1400, 1311, 1289, 1248, 1126, 1063, 1024, 945, 923, 844, 752, 724, 714; FTMS (ESI-TOF) *m/z*: $[M+H]^+$ Calcd for $C_{20}H_{20}NO_4^+$ m/z: 338.1387, Found 338.1519.

2.15. General procedure for the "*one-pot*" synthesis of optically active 1-aryloxy-3-amine-2-propanols 7a–f

A mixture of 2-{[(2*R*)-oxiran-2-yl]methyl}-1*H*-isoindole-1,3(2*H*)-dione [(*R*)-(–)-**3**, 1.02 g, 5.0 mmol, >99% ee], the appropriate phenol (5.0 mmol), xylene (4 mL) and DBU (76 mg, 0.5 mmol, 75 µL) was stirred under argon at 120 °C for 24 h. After cooling to 80 °C, a portion of 2-PrOH (20 mL) and anhydrous hydrazine (1 mL, ca. 4.5 equiv) were added at once, and the mixture was vigorously stirred at 80 °C for 2 h. The reaction mixture was cooled to room temperature and 0.5N aq. NaOH (50 mL) was

added, and the aqueous layer was back-extracted with AcOEt (100 mL) [Attention! In the case of product (*R*)-(+)-**7c** and (*R*)-(+)-**7e**, the work-up procedure was extended of additional extraction with a portion of AcOEt (100 mL) and $CHCl_3$ (100 mL)]. Next, the extract was washed with 0.5N aq. NaOH (50 mL), brine (50 mL), dried over anhydrous $MgSO_4$, the drying agent was filtered off, and the permeate was evaporated *in vacuo* [Attention! In the case of product (*R*)-(+)-**7c** and (*R*)-(+)-**7e**, the extract was washed with 0.5N aq. NaOH (100 mL) and brine (100 mL)]. The crude oil was purified by column chromatography using an eluent system composed of $CHCl_3$/MeOH/Et_3N (50:50:2, v/v/v), affording desired products (*R*)-(+)-**7a**–**f**. Attention (i): in the case of (*R*)-(+)-**7a**, the thus obtained brownish solid was further washed with cold Et_2O (25 mL), and after filtration, the residue of Et_3N was evaporated under high-vacuum conditions (p=0.05 mmHg, at 30 °C for 4 h). Attention! (ii): in the case of (*R*)-(+)-**7c**, the thus obtained brownish solid was further dissolved in MeOH (0.2 mL), and a portion of cold Et_2O (25 mL) was added at once to a stirred solution. After 5 min of stirring, the precipitated brownish solid was filtered off under suction and dried using high-vacuum. The synthesis for (*S*)-(–)-**7a** with reversed stereochemistry at asymmetric carbon atom was performed in analogy to this procedure.

2-(2-Hydroxy-3-(naphthalen-1-yloxy)propyl)-1*H*-isoindole-1,3(2*H*)-dione (*rac*-7a).

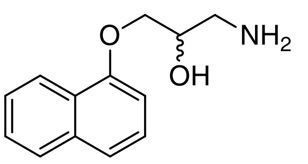

Yield 39% (388 mg) [yield after two steps]; pale brownish solid; mp 104–104.5 °C (Et_2O) [lit.[14] 97–99 °C (Et_2O)]; R_f [$CHCl_3$/MeOH/Et_3N (50:50:2, v/v/v)] 0.27; ^{1}H NMR (500 MHz, DMSO-d_6): δ 2.71 (dd, J=13.0, 6.6 Hz, 1H), 2.82 (dd, J=12.7, 4.9 Hz, 1H), 3.88 (quin, J=5.5 Hz, 1H), 4.02– 4.09 (m, 1H), 4.10–4.17 (m, 1H), 6.96 (d, J=7.3 Hz, 1H), 7.33–7.60 (m, 4H), 7.82–7.92 (m, 1H), 8.20–8.31 (m, 1H); ^{13}C NMR (126 MHz, DMSO-d_6): δ 44.9, 70.5, 70.6, 105.1, 119.8, 121.8, 125.0, 125.1, 126.2, 126.4, 127.4, 134.0, 154.2; IR (nujol): ν_{max} = 3364, 3278, 1592, 1580, 1509, 1312, 1272, 1240, 1213, 1181, 1127, 1100, 1073, 1020, 990, 822, 796, 768, 736; FTMS (ESI-TOF) *m/z*: $[M+H]^+$ Calcd for $C_{13}H_{16}NO_2^+$ m/z: 218.1176, Found 218.1176; The value of optical rotation for (*R*)-(+)-**7a**: $[\alpha]_D^{30}$ = +8.00 (*c* 0.25, EtOH). The synthesis of enantiomeric counterpart was performed on 0.47 mmol scale in analogy to this procedure, thus affording optically active amino alcohol (*S*)-(–)-**7a** {28 mg, 0.13 mmol, 27%, with undetermined % ee-values) as white solid.

2-[2-Hydroxy-3-(2-(prop-2-en-1-yl)phenoxy)propyl]-1*H*-isoindole-1,3(2*H*)-dione (*rac*-7b).

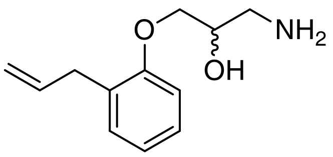

Yield 55% (529 mg) [yield after two steps]; yellowish oil (for *rac*-**7b**); 54–56 °C ($CHCl_3$/MeOH/Et_3N) [for (*R*)-(+)-**7b**]; R_f [$CHCl_3$/MeOH/Et_3N (50:50:2, v/v/v)] 0.55; ^{1}H NMR (500 MHz, DMSO-d_6): δ 2.59 (dd, *J*=13.0, 6.6 Hz, 1H), 2.72 (dd, *J*=12.7, 4.9 Hz, 1H), 3.32 (d, *J*=6.9 Hz, 2H), 3.73 (dd, *J*=6.9, 4.9 Hz, 1H), 3.88 (qd, *J*=9.9, 5.6 Hz, 2H), 4.94–5.09 (m, 2H), 5.95 (dd, *J*=17.1, 10.3 Hz, 1H), 6.83–6.88 (m, 1H), 6.90–6.95 (m, 1H), 7.06–7.12 (m, 1H), 7.13–7.19 (m, 1H); ^{13}C NMR (126 MHz, DMSO-d_6): δ 34.1, 45.0, 70.3, 70.7, 111.7, 115.6, 120.4, 127.5, 128.1, 129.6, 137.2, 156.3; IR (nujol): ν_{max} = 3367, 3074, 2928, 1639, 1600, 1585, 1492, 1452, 1321, 1290, 1248, 1121, 1054, 1032, 994, 914, 752, 653; FTMS (ESI-TOF) *m/z*: [M+H]$^+$ Calcd for $C_{12}H_{18}NO_2^+$ m/z: 208.1332, Found 208.1332; The values of optical rotations for enantiomerically enriched compounds are as follows: for (*R*)-(+)-**7b**: $[\alpha]_D^{30}$ = +24.00 (*c* 0.25, EtOH).

2-[2-Hydroxy-3-(1*H*-indol-4-yloxy)propyl]-1*H*-isoindole-1,3(2*H*)-dione (*rac*-7c).

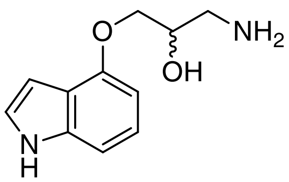

Yield 47% (450 mg) [yield after two steps]; brownish solid; mp 97–100 °C (Et_2O); R_f [$CHCl_3$/MeOH/Et_3N (50:50:2, v/v/v)] 0.27; ^{1}H NMR (500 MHz, CD_3OD): δ 1.90 (br. s., 1H), 2.81 (dd, *J*=14.1, 7.2 Hz, 1H), 2.93 (dd, *J*=13.0, 7.1 Hz, 1H), 3.09 (dd, *J*=13.0, 3.2 Hz, 1H), 3.17 (q, *J*=7.3 Hz, 1H), 4.00–4.19 (m, 2H), 6.47–6.52 (m, 1H), 6.53–6.59 (m, 1H), 6.91–7.05 (m, 2H), 7.09–7.13 (m, 1H); ^{13}C NMR (126 MHz, CD_3OD): δ 45.0, 70.4, 71.3, 99.7, 101.3, 106.3, 120.3, 123.1, 124.2, 139.4, 153.6; IR (nujol): ν_{max} = 3376–2724, 1583, 1548, 1292, 1241, 1130, 1086, 744, 726; FTMS (ESI-TOF) *m/z*: [M+H]$^+$ Calcd for $C_{11}H_{15}N_2O_2^+$ m/z: 207.1128, Found 207.1129; The values of optical rotations for enantiomerically enriched compounds are as follows: for (*R*)-(+)-**7c**: $[\alpha]_D^{32}$ = +48.00 (*c* 0.06, EtOH).

2-[3-(9*H*-Carbazol-4-yloxy)-2-hydroxypropyl]-1*H*-isoindole-1,3(2*H*)-dione (*rac*-7d).

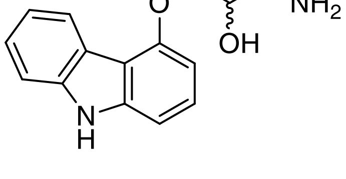

Yield 36% (433 mg) [yield after two steps]; brown solid; mp 138–140 °C ($CHCl_3$/MeOH/Et_3N) [lit.[15] 141–143 °C

(AcOEt)]; R_f [$CHCl_3$/MeOH/Et_3N (50:50:2, v/v/v)] 0.29; ^{1}H NMR (500 MHz, CD_3OD): δ 1.90 (br. s., 1H), 2.77 (q, J=7.3 Hz, 1H), 2.98–3.04 (m, 1H), 3.09–3.20 (m, 1H), 4.13–4.30 (m, 2H), 6.44–6.75 (m, 1H), 7.00–7.54 (m, 6H), 8.13–8.34 (m, 1H); ^{13}C NMR (126 MHz, CD_3OD): δ 45.2, 70.5, 71.1, 101.6, 105.3, 111.3, 119.9, 123.6, 124.0, 125.5, 125.8, 127.5, 140.9, 143.1, 156.5; IR (nujol): ν_{max} = 3408, 2721, 1706, 1604, 1496, 1347, 1299, 1264, 1220, 1153, 1108, 962, 755, 720; FTMS (ESI-TOF) *m/z*: $[M+H]^+$ Calcd for $C_{15}H_{17}N_2O_2^+$ m/z: 257.1285, Found 257.1284; The values of optical rotations for enantiomerically enriched compounds are as follows: for (*R*)-(+)-**7d**: $[\alpha]_D^{32}$ = +12.00 (*c* 0.13, EtOH).

1-Amino-3-(2-methoxyphenoxy)propan-2-ol (*rac*-7e).

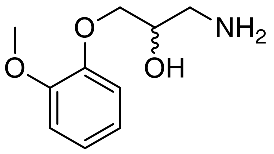

Yield 33% (322 mg) [yield after two steps]; beige solid; mp 104–106 °C ($CHCl_3$/MeOH/Et_3N) [lit.[16] 107–108.5 °C (MeOH/*i*-Pr_2O) for *rac*-**7e** or lit.[17] 91–93 °C (AcOEt) for (*R*)-**7e**]; R_f [$CHCl_3$/MeOH/Et_3N (50:50:2, v/v/v)] 0.35; ^{1}H NMR (500 MHz, $CDCl_3$): δ 2.41 (br. s., 3H), 2.79–3.00 (m, 2H), 3.84 (s, 3H), 3.93–4.08 (m, 3H), 6.82–7.00 (m, 4H); ^{13}C NMR (126 MHz, $CDCl_3$): δ 44.3, 55.9, 70.5, 72.6, 112.0, 114.9, 121.1, 122.1, 148.3, 149.9; IR (nujol): ν_{max} = 2924, 1508, 1460, 1376, 1252, 1120, 1032, 732; FTMS (ESI-TOF) *m/z*: $[M+H]^+$ Calcd for $C_{10}H_{16}NO_3^+$ m/z: 198.1125, Found 198.1125; The values of optical rotations for enantiomerically enriched compound is as follows: for (*R*)-(+)-**7e**: $[\alpha]_D^{27}$ = +3.50 (*c* 1.00, MeOH) (lit.: no data).

1-Amino-3-[4-(2-methoxyethyl)phenoxy]propan-2-ol (*rac*-7f).

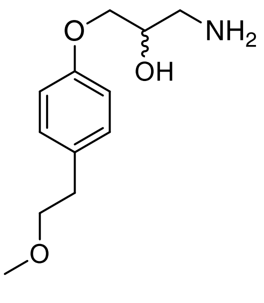

Yield 39% (441 mg) [yield after two steps]; beige solid; mp 70–71 °C ($CHCl_3$/MeOH/Et_3N) [lit.[18] 98–99 °C (AcOEt or EtOH)]; R_f [$CHCl_3$/MeOH/Et_3N (50:50:2, v/v/v)] 0.43; ^{1}H NMR (500 MHz, DMSO-d_6): δ 1.77 (t, J=2.0 Hz, 1H), 1.81–1.90 (m, 1H), 1.93–2.04 (m, 4H), 2.75 (t, J=6.9 Hz, 2H), 2.98 (t, J=5.6 Hz, 1H), 3.06–3.20 (m, 3H), 6.08–6.14 (m, 2H), 6.35–6.43 (m, 2H); ^{13}C NMR (126 MHz, DMSO-d_6): δ 34.5, 44.8, 57.8, 70.2, 70.3, 73.1, 114.2, 129.7, 130.8, 157.1; IR (nujol): ν_{max} = 2960, 1512, 1460, 1376, 1252, 1116; FTMS (ESI-TOF) *m/z*: $[M+H]^+$ Calcd for $C_{12}H_{20}NO_3^+$ m/z: 226.1438, Found 226.1437; The values of optical rotations for enantiomerically enriched compounds are as follows: for (*R*)-(+)-**7f**: $[\alpha]_D^{29}$ = +6.50 (*c* 1.00, MeOH) {lit.[18] $[\alpha]_D^{25}$ = +6.80 (*c* 1.00, MeOH) reported for (*R*)-**7f** in >99% ee}.

2.16. General procedure for the "*one-pot*" synthesis of optically active ß-blockers 8a–f

To a solution of optically active amino alcohol (*R*)-(+)-**7a** (100 mg; 0.46 mmol) in absolute EtOH (920 μL), acetone (80 mg; 1.38 mmol, 102 μL) was slowly dropped and after 45 min of stirring at room temperature $NaBH_4$ (35 mg; 0.92 mmol) was directly added. The mixture was stirred for a further 45 min at room temperature, then treated with 1 M HCl (552 μL) and successively with 1 M NaOH (276 μL). The crude mixture was evaporated to dryness, and a portion of AcOEt (4 mL) was added. The resulting suspension was alkalized to pH 9 with a 1M NaOH (4 mL) solution and subsequently extracted with AcOEt (2 × 4 mL). The organic layer was dried over anhydrous $MgSO_4$, and after filtering off the drying agent, the solvent was evaporated. The resulting crude API was purified by column chromatography on silica gel using a mixture of AcOEt/MeOH/25% $NH_{3aq.}$ (85:15:5, v/v/v) as an eluting solvent system, affording enantiomerically enriched propranolol (*R*)-(+)-**8a** (84 mg, 70% yield, 99% ee). The above procedure was extended toward the rest of the 1-aryloxy-3-amine-2-propanols (*R*)-(+)-**7b**–**f** yielding corresponding APIs (*R*)-(+)-**8b**–**f**. Attention: (*R*)-(+)-**8f** was purified by column chromatography using $CHCl_3$/MeOH (90:10, v/v) as eluent (whereas collection of the fractions was supported by monitoring with ninhydrin spray) and recrystallized additionally from Et_2O. The synthesis of optically active propranolol with reversed stereochemistry (*S*)-(–)-**8a** was also performed according to the above-mentioned protocol leading to (*S*)-(–)-**8a** (82 mg, 69% yield, 99% ee).

1-[(Naphthalen-1-yl)oxy]-3-[(propan-2-yl)amino]propan-2-ol (Propranolol, *rac*-8a).

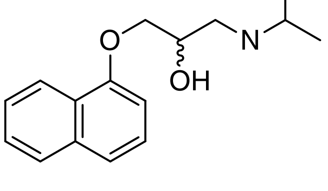

Yield 70% (84 mg); light-beige solid; mp 88–89 °C (AcOEt/MeOH/25% $NH_{3aq.}$) [lit.[19] 88–89 °C (Et_2O)]; R_f [AcOEt/MeOH/25% $NH_{3aq.}$ (85:15:5, v/v/v)] 0.56; ^{1}H NMR (500 MHz, $CDCl_3$): δ 1.12 (dd, *J*=6.1, 1.2 Hz, 6H), 2.74 (br. s., 1H), 2.83–2.92 (m, 2H), 3.01 (dd, *J*=12.2, 3.4 Hz, 1H), 4.10–4.16 (m, 1H), 4.17–4.23 (m, 2H), 6.80–6.85 (m, 1H), 7.37 (t, *J*=7.8 Hz, 1H), 7.42–7.53 (m, 3H), 7.77–7.83 (m, 1H), 8.22–8.28 (m, 1H); ^{13}C NMR (126 MHz, $CDCl_3$): δ 22.9, 23.1, 49.1, 49.5, 68.5, 70.7, 104.9, 120.7, 121.8, 125.3, 125.6, 125.9, 126.5, 127.5, 134.5, 154.3; IR (nujol): ν_{max} = 3274, 1600, 1584, 1508, 1403, 1266, 1238, 1155, 1104, 1066, 1022, 996, 876, 788, 764, 733; FTMS (ESI-TOF) *m/z*: $[M+H]^+$ Calcd for $C_{16}H_{22}NO_2^+$ m/z:

260.1645, Found 260. 1644; HPLC [*n*-hexane-EtOH-DEA (95:5:0.1, v/v/v); f = 0.5 mL/min; λ = 232 nm]: t_R = 27.581 min (*R*-isomer) and 46.009 min (*S*-isomer) or [*n*-hexane-EtOH-DEA (90:10:0.2, v/v/v); f=1.0 mL/min; λ = 232 nm (Chiralcel OD-H)]: t_R = 8.410 min (*R*-isomer) and 11.110 min (*S*-isomer); The values of optical rotations for enantiomerically enriched compounds are as follows: for (*R*)-(+)-**8a**: $[\alpha]_D^{32}$ = +23.63 (*c* 0.28, EtOH, for 99% ee) and for (*S*)-(–)-**8a**: $[\alpha]_D^{29}$ = –10.26 (*c* 1.17, EtOH, for 99% ee) {lit.[20] $[\alpha]_D^{25}$ = –10.50 (*c* 1.50, EtOH) reported for (*S*)-**8a** in 99.1% ee}.

1-[(Propan-2-yl)amino]-3-[2-(prop-2-en-1-yl)phenoxy]propan-2-ol (Alprenolol, *rac*-8b).

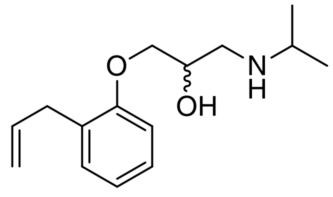

Yield 56% (67 mg); white solid; mp 60–62 °C (AcOEt/MeOH/25% $NH_{3aq.}$) [lit.[21] 62.4 °C (no data)]; R_f [AcOEt/MeOH/25% $NH_{3aq.}$ (85:15:5, v/v/v)] 0.55; ^{1}H NMR (500 MHz, $CDCl_3$): δ 1.10 (d, *J*=5.9 Hz, 6H), 2.22–2.57 (m, 3H), 2.77 (dd, *J*=12.0, 7.6 Hz, 1H), 2.84 (dt, *J*=12.4, 6.3 Hz, 1H), 2.90 (dd, *J*=12.2, 3.9 Hz, 1H), 3.40 (dd, *J*=6.4, 1.5 Hz, 2H), 3.95–4.01 (m, 2H), 4.01–4.08 (m, 1H), 5.02 (dq, *J*=7.6, 1.7 Hz, 1H), 5.05 (t, *J*=1.5 Hz, 1H), 5.93–6.04 (m, 1H), 6.83–6.87 (m, 1H), 6.89–6.94 (m, 1H), 7.12–7.16 (m, 1H), 7.17–7.21 (m, 1H); ^{13}C NMR (126 MHz, $CDCl_3$): δ 22.9, 23.0, 34.7, 48.9, 49.4, 68.6, 70.5, 111.3, 115.2, 120.9, 127.5, 128.5, 130.0, 137.2, 156.3; IR (nujol): ν_{max} = 3272, 1635, 1600, 1587, 1492, 1339, 1248, 1187, 1123, 1084, 1032, 990, 916, 898, 748; FTMS (ESI-TOF) *m/z*: $[M+H]^+$ Calcd for 250.1802, Found 250.1800; HPLC [*n*-hexane-EtOH-DEA (95:5:0.1, v/v/v); f = 0.5 mL/min; λ = 232 nm (Chiralcel OD-H)]: t_R = 10.832 min (*R*-isomer) and 15.817 min (*S*-isomer); The values of optical rotations for enantiomerically enriched compounds are as follows: for (*R*)-(+)-**8b**: $[\alpha]_D^{33}$ = +2.33 (*c* 0.22, EtOH, for 96% ee) {lit.[22] $[\alpha]_D^{20}$ = –14.10 (*c* 3.80, EtOH) reported for (*S*)-**8b** with undetermined % ee-values}.

1-[(1*H*-Indol-4-yl)oxy]-3-[(propan-2-yl)amino]propan-2-ol (Pindolol, *rac*-8c).

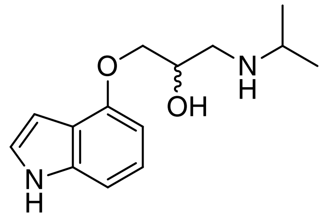

Yield 45% (54 mg); beige solid; mp 159–161 °C (AcOEt/MeOH/25% $NH_{3aq.}$) [lit.[23] 170 °C (no data)]; R_f [AcOEt/MeOH/25% $NH_{3aq.}$ (85:15:5, v/v/v)] 0.45; ^{1}H NMR (500 MHz, CD_3OD): δ 1.18 (dd, *J*=6.4, 2.5 Hz, 6H), 1.90 (s, 1H), 2.01 (s, 1H), 2.85 (dd, *J*=11.7, 8.8 Hz, 1H), 2.98–3.08 (m, 2H), 4.05–4.11 (m, 1H), 4.12–4.23 (m, 2H), 6.49–6.52 (m, 1H), 6.54 (d, *J*=3.9 Hz,

1H), 6.97–7.04 (m, 2H), 7.11 (d, *J*=3.4 Hz, 1H); ^{13}C NMR (126 MHz, CD_3OD): δ 21.6, 24.2, 50.4, 50.6, 69.2, 71.7, 99.5, 101.2, 106.1, 120.2, 122.9, 124.0, 139.2, 153.5; IR (nujol): ν_{max} = 3303–2724, 1687, 1643, 1617, 1585, 1572, 1506, 1414, 1283, 1248, 1213, 1153, 1130, 1096, 1057, 1045, 965, 933, 914, 895, 879, 841, 819, 760, 749, 720, 650; FTMS (ESI-TOF) *m/z*: $[M+H]^+$ Calcd for 249.1598, Found 249.1597; HPLC [*n*-hexane-EtOH-DEA (90:10:0.2, v/v/v); f = 1.0 mL/min; λ = 264 nm (Chiralcel OD-H)]: t_R = 16.822 min (*R*-isomer) and 97.907 min (*S*-isomer) or [*n*-hexane-EtOH-DEA (80:20:0.2, v/v/v); f = 1.0 mL/min; λ = 264 nm]: t_R = 7.080 min (*R*-isomer) and 26.055 min (*S*-isomer); The values of optical rotations for enantiomerically enriched compounds are as follows: for (*R*)-(+)-**8c**: $[\alpha]_D^{33}$ = +15.22 (*c* 0.23, EtOH, for 99% ee) {lit.[24] $[\alpha]_D^{20}$ = –11.80 (*c* 1.00, EtOH) reported for (*S*)-**8c** in >99% ee}.

1-[(9*H*-Carbazol-4-yl)oxy]-3-[(propan-2-yl)amino]propan-2-ol (Carazolol, *rac*-8d).

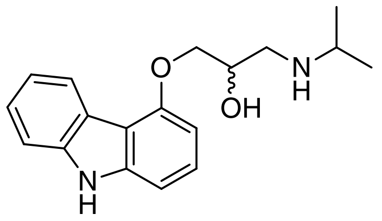

Yield 57% (67 mg); white solid; mp 145–146 °C (AcOEt/MeOH/25% $NH_{3aq.}$) [lit.[25] 147–148 °C (AcOEt/MeOH)]; R_f [AcOEt/MeOH/25% $NH_{3aq.}$ (85:15:5, v/v/v)] 0.56; ^{1}H NMR (500 MHz, CD_3OD): δ 1.09 (m, 6H), 1.15–1.24 (m, 1H), 1.91–1.98 (m, 1H), 2.78–2.92 (m, 2H), 2.98–3.08 (m, 1H), 4.02–4.15 (m, 1H), 4.17–4.30 (m, 2H), 6.60–6.66 (m, 1H), 7.03–7.08 (m, 1H), 7.08–7.16 (m, 1H), 7.20–7.35 (m, 2H), 7.36–7.48 (m, 1H), 8.24–8.33 (m, 1 H); ^{13}C NMR (126 MHz, CD_3OD): δ 22.1, 22.1, 50.3, 51.0, 69.6, 71.7, 101.7, 105.2, 111.3, 113.7, 119.9, 123.6, 124.0, 125.8, 127.6, 140.8, 143.0, 156.5; IR (nujol): ν_{max} = 3395, 3297, 1605, 1586, 1504, 1346, 1333, 1304, 1263, 1213, 1175, 1096, 1013, 982, 783, 754, 724; FTMS (ESI-TOF) *m/z*: $[M+H]^+$ Calcd for $C_{18}H_{23}N_2O_2^+$ m/z: 299.1754, Found 299.1753; HPLC [*n*-hexane-EtOH-DEA (80:20:0.2, v/v/v); f = 0.5 mL/min; λ = 220 nm (Chiralcel OD-H)]: t_R = 22.211 min (*S*-isomer) and 25.415 min (*R*-isomer); The values of optical rotations for enantiomerically enriched compounds are as follows: for (*R*)-(+)-**8d**: $[\alpha]_D^{33}$ = +6.67 (*c* 0.15, AcOH, for 97% ee) {lit.[26] $[\alpha]_D^{22}$ = –17.60 (*c* 1.00, AcOH) reported for (*S*)-**8d** with undetermined % ee-values}.

1-(2-Methoxyphenoxy)-3-[(propan-2-yl)amino]propan-2-ol (Moprolol, *rac*-8e).

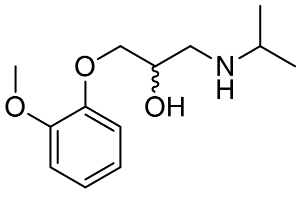

Yield 65% (71 mg); white solid; mp 104–106 °C (AcOEt/MeOH/25% $NH_{3aq.}$) [lit.[27] 81–82 °C (2,2,4-trimethylpentane) or lit.[28] 82–83 °C (Et_2O/hexane) for (*S*)-**8e**]; R_f [AcOEt/MeOH/25% $NH_{3aq.}$ (85:15:5, v/v/v)] 0.64; ^{1}H NMR (500 MHz, $CDCl_3$): δ 1.08 (dd, *J*=6.4, 2.0 Hz, 6H), 2.65 (br. s., 1H), 2.71–2.91 (m, 3H), 3.85 (s, 3H), 3.97–4.02 (m, 1H), 4.02–4.09 (m, 2H), 6.83–7.00 (m, 4H); ^{13}C NMR (126 MHz, $CDCl_3$): δ 23.1, 49.0, 49.5, 55.9, 68.5, 73.1, 112.0, 114.9, 121.1, 122.0, 148.4, 149.9; IR (nujol): ν_{max} = 2920, 1592, 1508, 1460, 1376, 1256, 1124, 1028, 740; FTMS (ESI-TOF) *m/z*: $[M+H]^+$ Calcd for $C_{13}H_{22}NO_3^+$ 240.1594, Found 240.1593; HPLC [*n*-hexane-EtOH-DEA (90:10:0.1, v/v/v); f = 1.2 mL/min; λ = 220 nm (Chiralcel OD-H)]: t_R = 5.020 min (*R*-isomer) and 19.876 min (*S*-isomer); The values of optical rotations for enantiomerically enriched compounds are as follows: for (*R*)-(+)-**8e**: $[\alpha]_D^{28}$ = +2.00 (*c* 1.00, $CHCl_3$, for >99% ee) {lit.[28] $[\alpha]_D^{27}$ = –5.60 (*c* 0.50, $CHCl_3$) reported for (*S*)-**8e** in 96% ee}.

1-[4-(2-Methoxyethyl)phenoxy]-3-(propan-2-ylamino)propan-2-ol (Metoprolol, *rac*-8f).

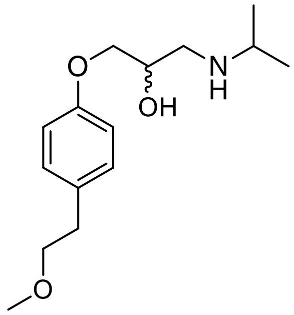

Yield 33% (39 mg); white crystals; mp 50–52 °C (Et_2O) [lit.[29] 35 °C ($CHCl_3$) or lit.[30] 45–47 °C (Et_2O)]; R_f [AcOEt/MeOH/25% $NH_{3aq.}$ (85:15:5, v/v/v), vis. ninhydrin stain] 0.58 or R_f [$CHCl_3$/MeOH (90:10, v/v), vis. ninhydrin stain] 0.29; ^{1}H NMR (500 MHz, $CDCl_3$): δ 1.10 (d, *J*=6.1 Hz, 6H), 2.73 (dd, *J*=12.1, 7.9 Hz, 1H), 2.78–2.92 (m, 4H), 3.35 (s, 3H), 3.56 (t, *J*=7.1 Hz, 2H), 3.90–3.99 (m, 2H), 4.00–4.07 (m, 1H), 6.82–6.87 (m, 2H), 7.18–7.16 (m, 2H); ^{13}C NMR (126 MHz, $CDCl_3$): δ 23.0, 35.4, 49.2, 49.4, 58.8, 68.5, 70.7, 74.0, 114.6, 129.9, 131.6, 157.3; IR (nujol): ν_{max} = 2924, 1460; FTMS (ESI-TOF) *m/z*: $[M+H]^+$ Calcd for $C_{15}H_{26}NO_3^+$ m/z: 268.1907, Found 268.1907; HPLC [*n*-hexane-EtOH-DEA (90:10:0.1, v/v/v); f = 1.2 mL/min; λ = 220 nm (Chiralcel OD-H)]: t_R = 4.366 min (*R*-isomer) and 6.145 min (*S*-isomer); The values of optical rotations for enantiomerically enriched compounds are as follows: for (*R*)-(+)-**8f**: $[\alpha]_D^{27}$ = +8.52 (*c* 1.35, $CHCl_3$, for 94% ee) {lit.[31] $[\alpha]_D^{26}$ = +8.70 (c, 10.0, $CHCl_3$) reported for (*R*)-**8f** in 94.4% ee or lit.[32] $[\alpha]_D^{22}$ = –8.78 (*c* 10.00, $CHCl_3$) reported for (*S*)-**8f** in 91% ee}.

2.17. General procedure for the synthesis of 2-(3-chloro-2-oxopropyl)-1*H*-isoindole-1,3(2*H*)-dione (12)

To a solution of the respective alcohol *rac*-**4** (240 mg, 1.0 mmol) in CH_2Cl_2 (10 mL) pyridinium chlorochromate (PCC, 646 mg, 3.0 mmol, 3.0 eqiuv) was added portion-wise over a period of 15 min at 35 °C. After 24 h of vigorous stirring, a portion of Celite (500 mg) was added, followed by $CHCl_3$ (20 mL). The heterogeneous slurry mixture was stirred for 5 min, then filtered over a pad of Celite and washed with $CHCl_3$ (3 × 20 mL). Evaporation of the filtrate under reduced pressure afforded a crude residue, which was further purified by column chromatography on SiO_2 using pure $CHCl_3$ as the eluent, thus affording the respective ketone **12**.

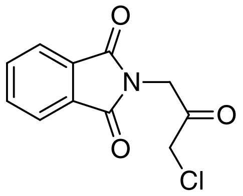

Yield 57% (135 mg); white solid; mp 141 °C ($CHCl_3$) [lit.[33] 139.5 °C ($CHCl_3$)]; R_f [$CHCl_3$ (100%)] 0.64; ^{1}H NMR (500 MHz, $CDCl_3$): δ 4.22 (s, 2H), 4.76 (s, 2H), 7.75 (dd, *J*=4.89, 2.93 Hz, 2H), 7.88 (dd, *J*=4.89, 2.93 Hz, 2H); ^{13}C NMR (126 MHz, $CDCl_3$): δ 44.8, 46.3, 123.8, 132.1, 134.5, 167.6, 195.6; IR (nujol): ν_{max} = 2924, 1720, 1464, 1420, 1192, 1100, 1064, 968, 772, 712; FTMS (ESI-TOF) *m/z*: $[M+H]^+$ Calcd for $C_{11}H_9ClNO_3^+$ m/z: 238.0267, Found 238.0266; GC [220–260 (10 °C/min)]: t_R = 4.012 min.

2.18. General procedure for the synthesis of 2-{3-chloro-2-[(trimethylsilyl)oxy]propyl}-2,3-dihydro-1*H*-isoindole-1,3-dione (*rac*-13)

To a solution of *rac*-**4** (100 mg, 0.42 mmol) in CH_2Cl_2 (2 mL) *N,O*-bis(trimethylsilyl)acetamide (BSA, 300 mg, 365 μL, 1.47 mmol, 3.5 eqiuv) was added in one portion at ambient temperature. After 30 min of stirring, the reaction mixture was diluted with CH_2Cl_2 (3 mL) and washed with H_2O (3 × 3 mL). The organic layer was dried over anhydrous $MgSO_4$. The drying agent was filtered off, and the remaining permeate was concentrated under high vacuum to remove an excess of BSA silylating reagent. The crude mixture was re-dissolved in $CHCl_3$ (500 μL) and purified by column chromatography on silica gel using a mixture of $CHCl_3$/acetone (98:2, v/v) as an eluent to afford desired product *rac*-**13** (75 mg, 0.24 mol, 58%) as white solid.

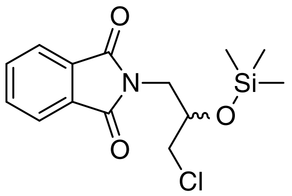

Mp 69–70 °C ($CHCl_3$/acetone); R_f [$CHCl_3$/acetone (98:2, v/v)] 0.65; ^{1}H NMR (500 MHz, $CDCl_3$): δ 0.06 (s, 9H), 3.43–3.57 (m, 2H), 3.75–3.88 (m, 2H), 4.19 (s, 1H),

7.68–7.77 (m, 2H), 7.82–7.91 (m, 2H); ^{13}C NMR (126 MHz, $CDCl_3$): δ 0.1 (3C), 42.1, 47.1, 70.4, 123.5 (4C), 132.1, 134.3, 168.3 (2C); IR (nujol): ν_{max} = 2856, 1772, 1724, 1460, 1376, 1252, 1100, 1020, 948, 876, 848, 756, 724; FTMS (ESI-TOF) *m/z*: $[M+H]^+$ Calcd for $C_{14}H_{19}ClNO_3Si^+$ m/z: 312.0817, Found 312.0818; GC [220–250 (10 °C/min)]: t_R = 3.647 min.

2.19. General procedure for the stereoselective bioreduction of prochiral ketone 12 – *biocatalysts screening*

Each of the lyophilized whole-cell biocatalysts including wild-type microorganisms and/or *E. coli* cells with overexpressed recombinant ADHs (10 mg), were suspended in 0.1 M Tris–HCl buffer (350 μL; pH 7.5) with an additional portion of NADH [50 μL was taken from the 5 mM stock solution prepared in Tris–HCl buffer (1 mL) to obtain 0.5 mM final conc. of cofactor], and preincubated for 30 min at 30 °C. In the case of wild-type microorganisms, glucose (50 μL) was added from a 0.2 M stock solution prepared by dissolving carbohydrate (36 mg) in 0.1 M Tris-HCl (pH 7.5, 1 mL) to obtain 20 mM final conc., and a final volume of 0.5 mL. In the case of *E. coli*/ADHs, instead of glucose, pure 0.1 M Tris–HCl buffer (50 μL; pH 7.5) was added to obtain a final volume of 0.5 mL. Then, ketone **12** [50 μL was taken from the 100 mM stock solution prepared in 2-propanol (1 mL) supplemented with DMSO (5% v/v) to obtain 10 mM final conc. of the substrate **12**] was added, and the reaction mixture was shaken at 30 °C and 250 rpm for 48 h without air access. After incubation, the enzymatic reaction was stopped by extracting the content of the vial with AcOEt (3 × 1 mL), while the combined organic phase was washed additionally with H_2O (1.5 mL) and dried over anhydrous $MgSO_4$. Next, the filtrate was centrifuged (5 min, 6000 rpm), and the obtained supernatant was transferred into two separate HPLC vials and concentrated under a vacuum. The oil residue in the first vial was used to determine % conv. by using GC analysis after derivatization of the crude mixture with BSA reagent (see protocol below). The reaction % conv. were calculated from the peak area calibrated with the mixtures of the standard compounds. The other portion of oil residue placed in the second vial was re-dissolved in an HPLC-grade mixture of hexane/2-PrOH (90:10 v/v, 1.5 mL), and the sample was analyzed by HPLC on a chiral stationary phase to establish % ee of the optically active alcohols (*S*)-(–)-**4** or (*R*)-(+)-**4** (see the Supporting Information). For additional data, see **Table 6** in the main manuscript.

2.20. General procedure for the derivatization of the samples for GC analyses with BSA as silylation reagent

To a vial containing oil residue after enzymatic reactions, a solution of *N,O*-bis(trimethylsilyl)acetamide (BSA, 15 mg, 71.3 μmol, 18 μL) in CH_2Cl_2 (100 μL) was added in one portion. After 20 min of vigorous vortexing of the reaction mixture at room temperature, the aliquot of the sample was directly analyzed using GC.

2.21. General procedure for preparative-scale bioreduction of 12 using *E. coli*/Lk-ADH-Lica

E. coli/Lk-ADH-Lica (50 mg) was suspended in 0.1 M Tris–HCl buffer (1.75 mL; pH 7.5) containing NADH (2.83 mg, 1.0 mM final concentration) and preincubated for 30 min at 30 °C. Then, ketone **12** (31 mg, 0.13 mmol) and 2-propanol (200 μL, 10% v/v) supplemented with DMSO (100 μL, 5.0% v/v) were added to the mixture. The reaction was shaken at 30 °C and 250 rpm for 48 h. After incubation, the enzymatic reaction was stopped by filtering off the cells under vacuum and rinsing the filtrate cake with AcOEt (3 × 5 mL). Next, a portion of $PhCH_3$ (15 mL) was added to the permeate, and the water was azeotropically evaporated using rotavap. The crude oil residue was purified by short-pad column chromatography (Pasteur pipette terminated with cotton wool and filled with SiO_2 gel) using a mixture of $CHCl_3$/acetone (95:5, v/v), thus obtaining desired optically active product (*S*)-(–)-**4** (14.3 mg, 0.06 mmol, 46% isolated yield, >99% ee) as a white solid.

3. Discussion on the results of synthetic procedures

3.1. Synthesis of the Racemic Starting Materials *rac*-4 and *rac*-5a–b

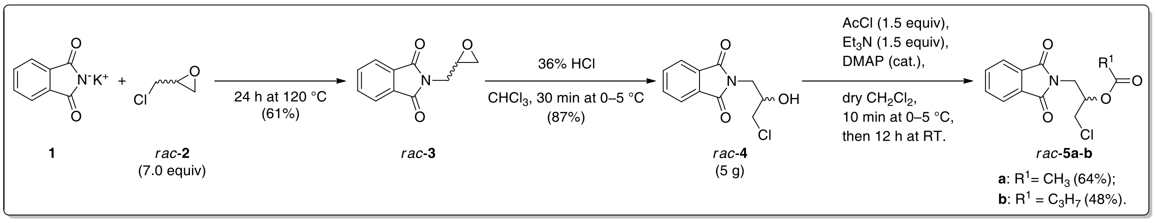

The racemic substrate *rac*-**4** and reference esters *rac*-**5a**–**b** required for biocatalytic investigations were obtained in a 2–3-step reaction sequence starting from cheap commercially available potassium phthalimide (**1**) and racemic epichlorohydrin (*rac*-**2**). In the first step, racemic glycidyl phthalimide (*rac*-**3**) was prepared by regioselective ring-opening of *rac*-**2** carried out with **1** under both catalyst- and solvent-free conditions. The epichlorohydrin (*rac*-**2**) was used in substantial 7-fold molar excess with respect to **1** because, in this case, *rac*-**2** also acted very well as a solvent. After stirring a neat reaction mixture at 120 °C for 24 h, the desired product *rac*-**3** was isolated in a high 61% yield after recrystallization from MeOH. Next, phthalimide epoxide *rac*-**3** was treated with 36% HCl to afford the desired racemic chlorohydrin, namely 2-(3-chloro-2-hydroxypropyl)-1*H*-isoindole-1,3(2*H*)-dione (*rac*-**4**), in an excellent 87% yield without the necessity of using a purification procedure. Notably, the regioselective HCl-mediated ring-opening of oxirane *rac*-**3** was carried out in cold $CHCl_3$ solution (0–5 °C) and resulted exclusively in the formation of a secondary alcohol *rac*-**4** according to NMR indications. Although using strong acidic conditions, the reaction proceeded solely at sterically less hindered electrophilic carbon atom of *rac*-**3** without formation of undesired regioisomer (primary alcohol).

Subsequently, *rac*-**4** was esterified by the use of standard [4-dimethylaminopyridine (DMAP)]-catalyzed acylation conditions within 1.5-fold molar excess of the respective acyl chlorides and triethylamine (Et_3N) as a base. The corresponding racemic acetate *rac*-**5a** and butyrate *rac*-**5b** were obtained in the 48–64% yield range, respectively. With a racemic chlorohydrin *rac*-**4** and its esters *rac*-**5a**–**b** in hand, our next task was to elaborate reliable analytical methods (GC and HPLC) for enzymatic experiments to follow the reactions progress (i.e., % conv.) and stereochemical outcome (i.e., % ee), respectively. For both compounds, *rac*-**4** and *rac*-**5a**, we found adequate conditions for a good baseline separation of their enantiomers. Unfortunately, in the case of racemic butyrate *rac*-**5b** separation of the enantiomers was not feasible on the chiral phases available. Thus this ester could not be utilized directly as an analytical standard to evaluate the reactions' enantioselectivity. Moreover, owing to partial thermal decomposition of the *rac*-**5b** during gas chromatography analysis, we decided to abandon further studies with this derivative as the results of the conversion rates established on the basis of GC would be unreliable.

3.2. Lipase-Catalyzed Hydrolytic KR of *rac*-5a

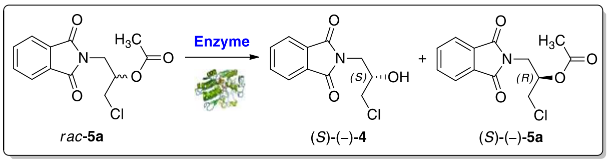

We screened a set of 34 various enzyme preparations, including fungal and bacterial lipases, esterases, and proteases. For the most promising biocatalyst, we have also tested 9 different co-solvents as reaction media. To our great disappointment, all the attempted EKR of *rac*-**5** turned out to be inefficient in terms of the rate and enantioselectivity, leading to moderate enantiomeric excesses of (*S*)-(–)-**4** (up to 84% ee) and (*R*)-(–)-**5a** (up to 90% ee).

Table S1. Enzyme screening for hydrolytic KR of *rac*-**5a** in KPi buffer (pH 7.5)/CH_3CN at 40 °C for 72 h.

Entry	Enzyme preparation[a]	Conv.[b] [%]	ee_s[c] [%]	ee_p[c] [%]	*E*[e]
1	Novozym 435	0	N.D.[f]	N.D.[f]	N.D.[f]
2	Lipozyme 435	0	N.D.[f]	N.D.[f]	N.D.[f]
3	Chirazyme L-2, C-2	0	N.D.[f]	N.D.[f]	N.D.[f]
4	Chirazyme L-2, C-3	0	N.D.[f]	N.D.[f]	N.D.[f]
5	Lipozyme CALB L	0	N.D.[f]	N.D.[f]	N.D.[f]
6	Chirazyme L-5	66	81	42	6
7	NovoCor AD L	67	90	45	7
8	Immozyme CAL-A-T2-150	<10	N.D.[f]	N.D.[f]	N.D.[f]
9	Amano PS-IM	28	33	83	15
10	PS-Immobead 150	0	N.D.[f]	N.D.[f]	N.D.[f]
11	Amano PS	15	25	84	23
12	Immozyme ASMQ-T2-150	<2	N.D.[f]	N.D.[f]	N.D.[f]
13	Amano AK	<10	N.D.[f]	N.D.[f]	N.D.[f]
14	TL-Immobead 150	0	N.D.[f]	N.D.[f]	N.D.[f]
15	Lipozyme TL IM	0	N.D.[f]	N.D.[f]	N.D.[f]
16	Lipozyme TL 100 L	0	N.D.[f]	N.D.[f]	N.D.[f]
17	Lipozyme RM IM	0	N.D.[f]	N.D.[f]	N.D.[f]
18	Amano 10 Lipase M	0	N.D.[f]	N.D.[f]	N.D.[f]
19	Amano Lipase F-AP15	<2	N.D.[f]	N.D.[f]	N.D.[f]
20	Lipase AY Amano 30	0	N.D.[f]	N.D.[f]	N.D.[f]
21	Lipase Type VII	<10	N.D.[f]	N.D.[f]	N.D.[f]
22	Lecitase Ultra	0	N.D.[f]	N.D.[f]	N.D.[f]
23	PLE	0	N.D.[f]	N.D.[f]	N.D.[f]
24	Chirazyme E-3	>99	N.D.[f]	N.D.[f]	N.D.[f]
25	Chirazyme E-4	>99	N.D.[f]	N.D.[f]	N.D.[f]
26	α-Chymotrypsin from bovine pancreas	0	N.D.[f]	N.D.[f]	N.D.[f]
27	Everlase 6.0 T	0	N.D.[f]	N.D.[f]	N.D.[f]
28	Protease A Amano 2G	0	N.D.[f]	N.D.[f]	N.D.[f]
29	Protease P Amano 6	0	N.D.[f]	N.D.[f]	N.D.[f]
30	Protease M Amano	<2	N.D.[f]	N.D.[f]	N.D.[f]
31	Protease N Amano	0	N.D.[f]	N.D.[f]	N.D.[f]
32	Protease S Amano	<2	N.D.[f]	N.D.[f]	N.D.[f]
33	Protease Sigma Type XXIII	0	N.D.[f]	N.D.[f]	N.D.[f]
34	Subtilisin Merck 24722	<2	N.D.[f]	N.D.[f]	N.D.[f]

[a] Conditions: *rac*-**5a** 50 mg, 180 µmol, enzyme 25 mg (for solid enzymes) or 200 µL (for liquid enzymes), 0.1 M K_2HPO_4/KH_2PO_4 buffer (pH 7.5)/CH_3CN (1.1 mL; 10:1 v/v), 40 ºC, 500 rpm (magnetic stirrer). [b] Based on GC, for confirmation the % conversion was calculated from the enantiomeric excess of the unreacted alcohol (ee_s) and the product (ee_p) according to the formula conv. = $ee_s/(ee_s + ee_p)$. [c] Determined by chiral HPLC analysis by using a Chiralpak OD-H column. [d] Absolute configuration. [e] Calculated according to Chen *et al.*,[34] using the equation: $E = \{\ln[(1 - \text{conv.})(1 - ee_s)]\}/\{\ln[(1 - \text{conv.})(1 + ee_s)]\}$. [f] Not determined.

Table S2. Co-solvent screening for (NovoCor AD L)-catalyzed KR of *rac*-**5a** in KPi buffer (pH 7.5)/organic solvent at 40 °C for 48 h.

Entry	Solvent[a] (log *P*)[b]	Conv.[c] [%]	ee_s[d] [%]	ee_p[d] [%]	*E*[e]
1	1,4-Dioxane (–0.31)	<10	N.D.[f]	N.D.[f]	N.D.[f]
2	CH_3CN (0.17)	46	64	75	13
3	Acetone (0.20)	31	32	72	8
4	THF (0.40)	23	22	72	8
5	MTBE (0.96)[g]	42	45	61	6
6	CH_2Cl_2 (1.01)	<10	N.D.[f]	N.D.[f]	N.D.[f]
7	*t*-Amyl alcohol (1.09)[g]	59	38	26	2
8	$CHCl_3$ (1.67)	37	32	54	5
9	$PhCH_3$ (2.52)	30	20	47	3

[a] Conditions: *rac*-**5a** 50 mg, 180 µmmol, NovoCor AD L 200 µL, 0.1 M K_2HPO_4/KH_2PO_4 buffer (pH 7.5)/organic solvent (1.1 mL; 10:1 v/v), 40 °C, 500 rpm (magnetic stirrer). [b] Logarithm of the partition coefficient of a given solvent between *n*-octanol and water according to ChemBioDraw Ultra 13.0 software indications. [c] Based on GC, for confirmation, the % conversion was calculated from the enantiomeric excess of the unreacted alcohol (ee_s) and the product (ee_p) according to the formula conv. = $ee_s/(ee_s + ee_p)$. [d] Determined by chiral HPLC analysis. [e] Calculated using the equation: $E = \{\ln[(1 - \text{conv.})(1 - ee_s)]\}/\{\ln[(1 - \text{conv.})(1 + ee_s)]\}$[34]. [f] Not determined. [g] The volume of the solvent was increased up to 300 µL.

3.3. Screening of the reaction conditions for the synthesis of *rac*-6a

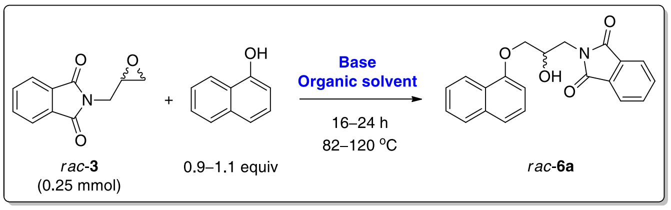

To obtain racemic 2-[2-hydroxy-3-(naphthalen-1-yloxy)propyl]-1*H*-isoindole-1,3(2*H*)-dione (*rac*-**6a**) several representative catalytic systems composed of various bases and organic solvents were tested using racemic *N*-(2,3-epoxypropyl)-phthalimide (*rac*-**3**) and 1-naphtol. The reactions conducted in neat triethylamine (Et_3N) and with K_2CO_3 or DBU in polar aprotic solvents (CH_3CN or DMF) failed to afford the desired product *rac*-**6a** in preparative yields. Only a suspension of anhydrous K_2CO_3 in $PhCH_3$ and a solution of DBU in xylene gave *rac*-**6a** in moderate to high yields (71–93%). For details, see the main manuscript.

Table S3. Conditions screening for the benchmark synthesis of *rac*-**6a**.[a]

Entry	Molar equivalent of 1-naphtol[b]	Base	Solvent[c]	t [h]	T [° C]	Yield[d] [%]
1	1.0 equiv	Et_3N (37 equiv)	–	24	90	21
2	1.0 equiv	anh. K_2CO_3 (3 equiv)	CH_3CN	16	82	<1
3	1.0 equiv	anh. K_2CO_3 (3 equiv)	DMF	16	120	6
4	0.9 equiv	anh. K_2CO_3 (2.7 equiv)	$PhCH_3$	16	111	71
5	1.1 equiv	DBU[e] (1.2 equiv)	DMF	24	120	11
6	1.0 equiv	DBU[e] (0.1 equiv)	Xylene	24	120	93

[a] An analytical scale: *rac*-**3** 50 mg, 0.25 mmol. [b] Used in ratio to *rac*-**3** (50 mg, 0.25 mmol). [c] Used in 1.5 mL/0.25 mmol of *rac*-**3**. [d] Isolated yield after column chromatography using a gradient of *n*-hexane/AcOEt (3:1, 1:1, v/v). [e] 1,8-Diazabicyclo[5.4.0]undec-7-ene.

3.4. Screening of the conditions of the S_N2 reaction of the mesylate (*R*)-(+)-9 with acetates during indirect inversion of the absolute configuration in (*R*)-(+)-4

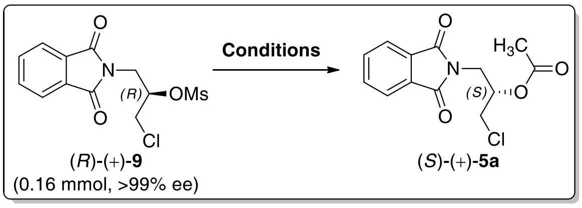

Next, we have tested various reaction conditions using cheap anhydrous sodium acetate (AcONa) as oxygen nucleophile in the presence of the catalytical amount of crown ether (18-Crown-6) or tetrabutylammonium hydrogensulfate (Bu_4NHSO_4) in dry DMF or $PhCH_3$, respectively. However, these attempts gave ester (*S*)-(+)-**5a** in poor yields (<5%). Encouraged by the results reported by Shi et al.,[35] the subsequent trials focused on applying tunable complexes formed between acetic acid (AcOH) and tertiary amines (Et_3N or DBU). Unfortunately, regardless of the complex used (Et_3N–AcOH or DBU–AcOH), the adjusted molar ratio of the tertiary amines and carboxylic acid, and the screened solvent (benzene, $PhCH_3$, DMF, or DMSO), only a trace of the desired product (*S*)-(+)-**5a** was detected, along with a highly complex mixture of by-products. Finally, the inversion of the configuration at the hydroxylated stereocenter was achieved using a slightly modified protocol reported by us previously.[36] For details, see the main manuscript.

3.5. Screening of the conditions for the hydrolysis of (*S*)-(+)-5a

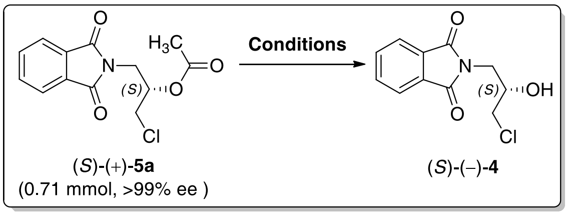

Attempts aimed at straightforward basic hydrolysis or methanolysis of the resulting acetate (*S*)-(+)-**5a** to convert it back to the free alcohol (*S*)-(–)-**4** failed. It should be pointed out that using either aqueous solutions of sodium hydroxide (NaOH) or lithium hydroxide (LiOH) as well as the suspension of potassium carbonate (K_2CO_3) in MeOH proceeded with a significant decomposition of the formed labile product. This is likely due to hydrolysis of the acetate (*S*)-(+)-**5a** to alcohol (*S*)-(–)-**4** and subsequent formation of the epoxide (*S*)-(+)-**3**, which reactive oxirane ring might be easily opened by hydroxide or methoxide ions to obtain corresponding 1,2-diol or α-methoxy alcohol, respectively. On the other hand, during hydrolytic KR of *rac*-**5a**, we found that two esterases derived from thermophilic microorganisms, namely Chirazyme E-3 and Chirazyme E-4, were potent to hydrolyze acetate *rac*-**5a** in a non-stereoselective fashion with >99% conv., and no side products were detected (for details, see Supporting Information). However, when we tried to adopt these reactions at preparative relevant conditions, the conversions reached approximately >95%. Even after increasing the enzyme loading and temperature to 50 °C, we could not achieve quantitative hydrolysis of (*S*)-(+)-**5a**, which affected the optical purity of the formed (*S*)-(–)-**4**. Finally, H_2SO_4-catalyzed hydrolysis of (*S*)-(+)-**5a** carried out in MeOH for 96 h at 35 °C afforded the desired enantiomerically pure (*S*)-(–)-**4** (>99% ee) in 86% isolated yields. For details, see the main manuscript.

Table S4. Optimal analytical separation conditions of different compounds by Chiralcel OD-H (Daicel) or Chiralpak AD-H (Daicel) chiral columns.

Compound	Ratio of *n*-hexane to 2-propanol	Elution velocity [mL/min]	Pressure [MPa]	Retention time [min]
**EPOXIDE**				
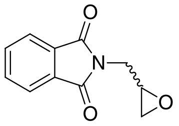 *rac*-**3**	90:10[a]	0.8	3.2	18.34 (*R*) and 19.55 (*S*)
	95:5[a]	0.8	3.0	26.89 (*R*) and 28.84 (*S*)
**ALCOHOL**				
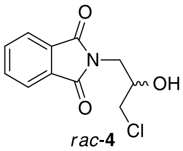 *rac*-**4**	90:10[a]	0.8	3.2	23.19 (*S*) and 31.09 (*R*)
**ACETATE**				
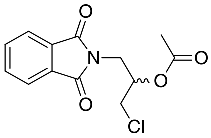 *rac*-**5a**	90:10[a]	0.8	3.2	21.26 (*S*) and 23.01 (*R*)
**MESYLATE**				
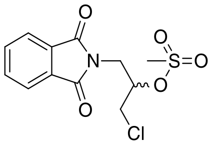 *rac*-**9**	85:15[b]	0.8	3.3	66.96 (*S*) and 77.31 (*R*)
	90:10[g]	1.0	3.6	47.82 (*S*) and 50.66 (*R*)
***para*-NO2-benzoates**				
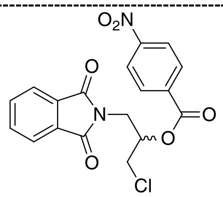 *rac*-**10**	90:10[b]	0.8	3.2	69.25 (*S*) and 74.71 (*R*)
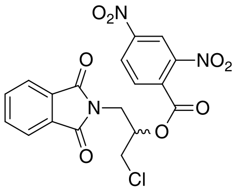 *rac*-**11**	78:22[h]	1.0	4.7	54.362 (*R*) and 60.823 (*S*)

β-blocker	Ratio of *n*-hexane to EtOH to DEA[c]	Elution velocity [mL/min]	Pressure [MPa]	Retention time [min]
		**β-BLOCKERS**		
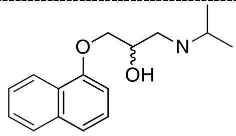 *rac*-**8a**	95:5:0.1[d]	0.5	1.7	27.58 (*R*) and 46.01 (*S*)
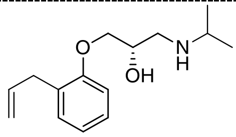 *rac*-**8b**	95:5:0.1[d]	0.5	1.7	10.83 (*R*) and 15.82 (*S*)
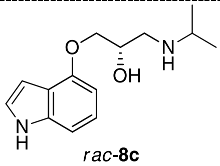 *rac*-**8c**	80:20:0.2[e]	1.0	4.3	7.080 (*R*) and 26.055 (*S*)
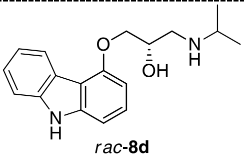 *rac*-**8d**	80:20:0.2[f]	0.5	2.1	22.211 (*S*) and 25.415 (*R*)
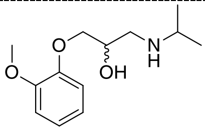 *rac*-**8e**	90:10:0.1[f]	1.2	5.4	5.020 (*S*) and 19.876 (*R*)
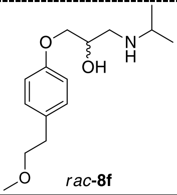 *rac*-**8f**	90:10:0.1[f]	1.2	5.4	4.366 (*R*) and 6.145 (*S*)

[a] The samples were carried out on Chiralcel OD-H at 254 nm and at 30 °C.
[b] The samples were carried out on Chiralcel OD-H at 219 nm and at 30 °C.
[c] Diethylamine (99+%, extra pure, Acros Organics; Cat. No: 149450010).
[d] The samples were carried out on Chiralcel OD-H at 232 nm and at 30 °C.
[e] The samples were carried out on Chiralcel OD-H at 264 nm and at 30 °C.
[f] The samples were carried out on Chiralcel OD-H at 220 nm and at 30 °C.
[g] The samples were carried out on Chiralpak AD-H at 216 nm and at 30 °C.
[h] The samples were carried out on Chiralpak AD-H at 217 nm and at 25 °C.

HPLC analytical separation for both enantiomers of *rac*-3

HPLC conditions: *n*-hexane-*i*-PrOH (95:5, v/v); f=0.8 mL/min; λ=254 nm; *p*=3.0 MPa (Chiralcel OD-H)

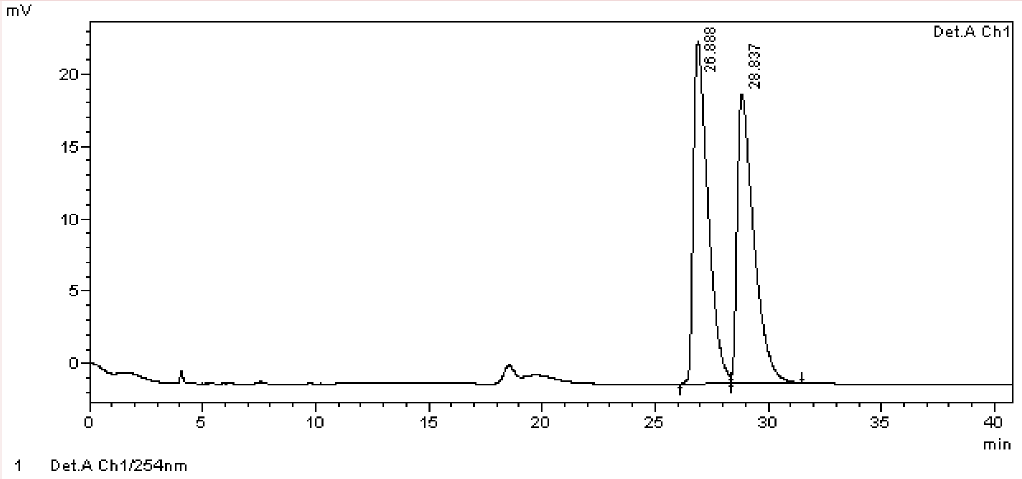

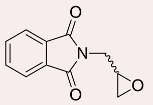

Peak Table

Detector A Ch1 254nm

Peak#	Ret. Time	Area	Height	Area %	Height %
1	26.888	1060000	23653	50.855	54.222
2	28.837	1024361	19969	49.145	45.778
Total		2084362	43622	100.000	100.000

HPLC of (*R*)-(–)-3 (>99% ee) obtained from (*R*)-(+)-4 (>99% ee) [the reaction conducted at 50 mg-scale]

HPLC conditions: *n*-hexane-*i*-PrOH (95:5, v/v); f=0.8 mL/min; λ=254 nm; *p*=3.0 MPa (Chiralcel OD-H)

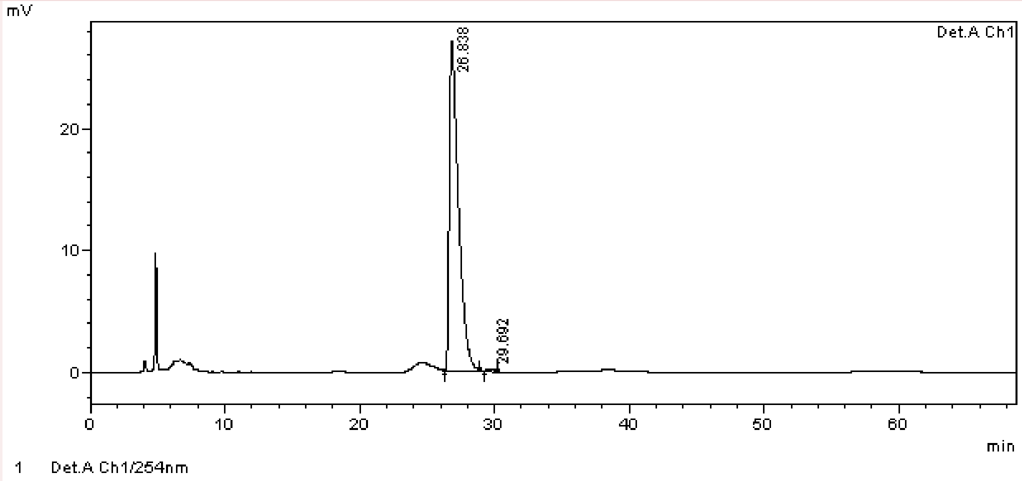

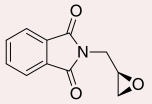

Peak Table

Detector A Ch1 254nm

Peak#	Ret. Time	Area	Height	Area %	Height %
1	26.838	1278231	26950	99.560	99.461
2	29.692	5655	146	0.440	0.539
Total		1283886	27096	100.000	100.000

HPLC of (*R*)-(–)-3 (>99% ee) obtained from (*R*)-(+)-4 (>99% ee) [the reaction conducted at 2 g-scale from EKR conducted at 2.5 g-scale]

HPLC conditions: *n*-hexane-*i*-PrOH (95:5, v/v); f=0.8 mL/min; λ=254 nm; *p*=3.0 MPa (Chiralcel OD-H)

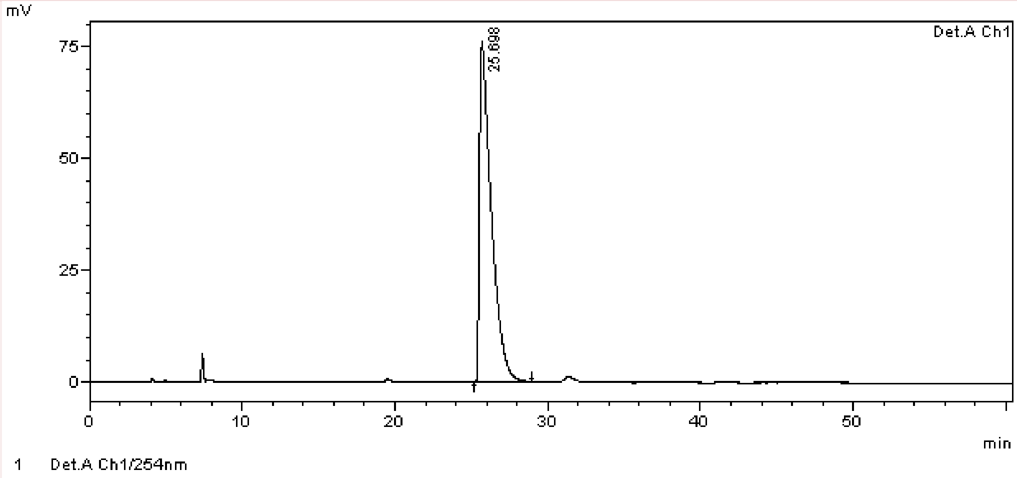

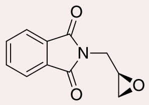

Peak Table

Detector A Ch1 254nm

Peak#	Ret. Time	Area	Height	Area %	Height %
1	25.698	4098497	76125	100.000	100.000
Total		4098497	76125	100.000	100.000

HPLC analytical separation for both enantiomers of *rac*-3 performed on Shimadzu LC-40 Nexera equipped with a photodiode array detector (PAD)

HPLC conditions: *n*-hexane-*i*-PrOH (95:5, v/v); f=0.8 mL/min; λ=254 nm; *p*=3.0 MPa (Chiralcel OD-H)

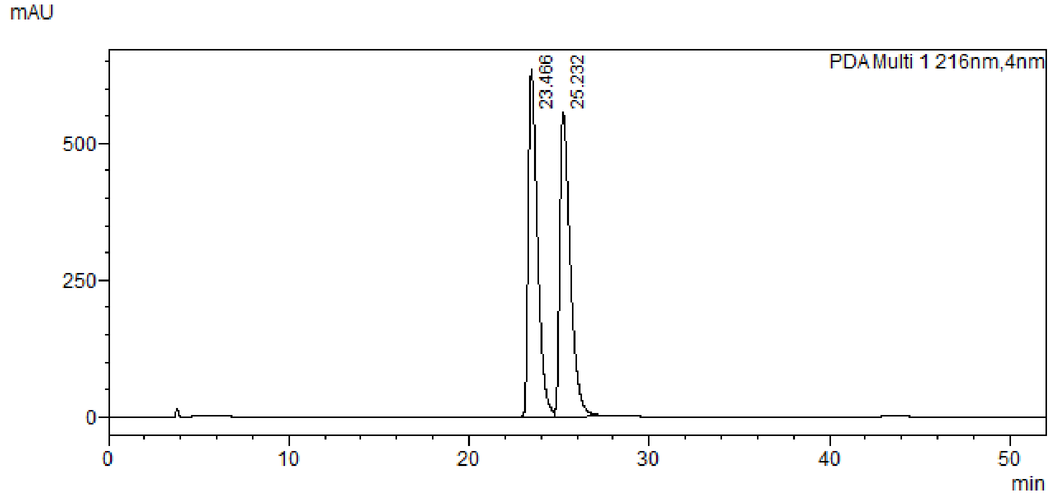

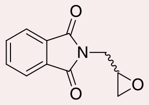

Peak Table

PDA Ch1 216nm

Peak#	Ret. Time	Area	Height	Height%	Area%
1	23.466	22447650	633802	53.295	49.934
2	25.232	22506638	555428	46.705	50.066
Total		44954288	1189230	100.000	100.000

HPLC of (*R*)-(–)-3 (>99% ee) obtained from (*R*)-(+)-4 (>99% ee) [the reaction conducted at 2 g-scale from EKR conducted at 5 g-scale] performed on Shimadzu LC-40 Nexera equipped with a photodiode array detector (PAD)

HPLC conditions: *n*-hexane-*i*-PrOH (95:5, v/v); f=0.8 mL/min; λ=254 nm; *p*=3.0 MPa (Chiralcel OD-H)

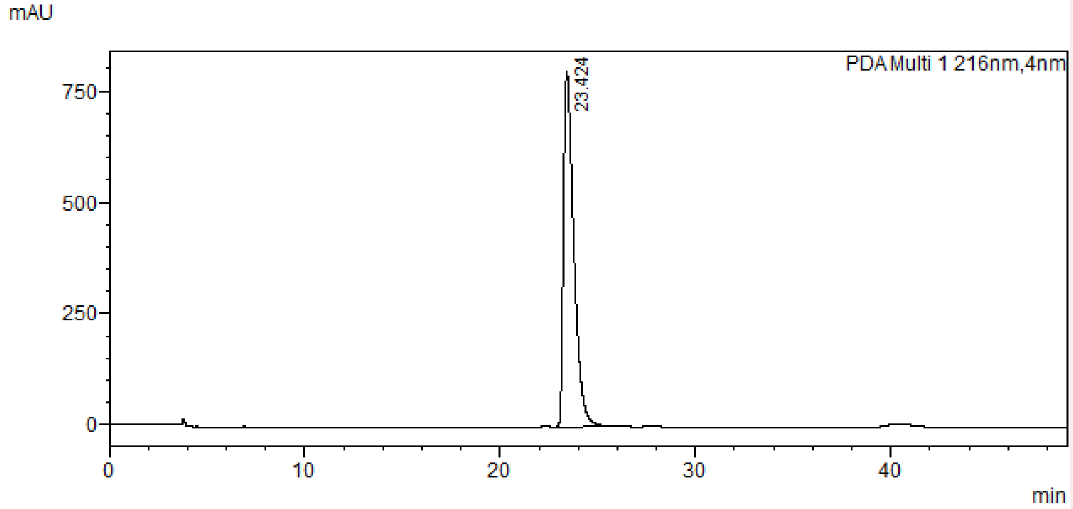

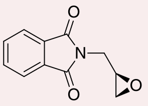

Peak Table

PDA Ch1 216nm

Peak#	Ret. Time	Area	Height	Height%	Area%
1	23.424	29345028	798282	100.000	100.000
Total		29345028	798282	100.000	100.000

HPLC of (*S*)-(+)-3 (>99% ee) obtained from (*S*)-(–)-4 (>99% ee) [after stereoinversion] performed on Shimadzu Nexera-*i* (LC-2040C 3D) equipped with a photodiode array detector (PAD)

HPLC conditions: *n*-hexane-*i*-PrOH (95:5, v/v); f=0.8 mL/min; λ=254 nm; *p*=3.0 MPa (Chiralcel OD-H)

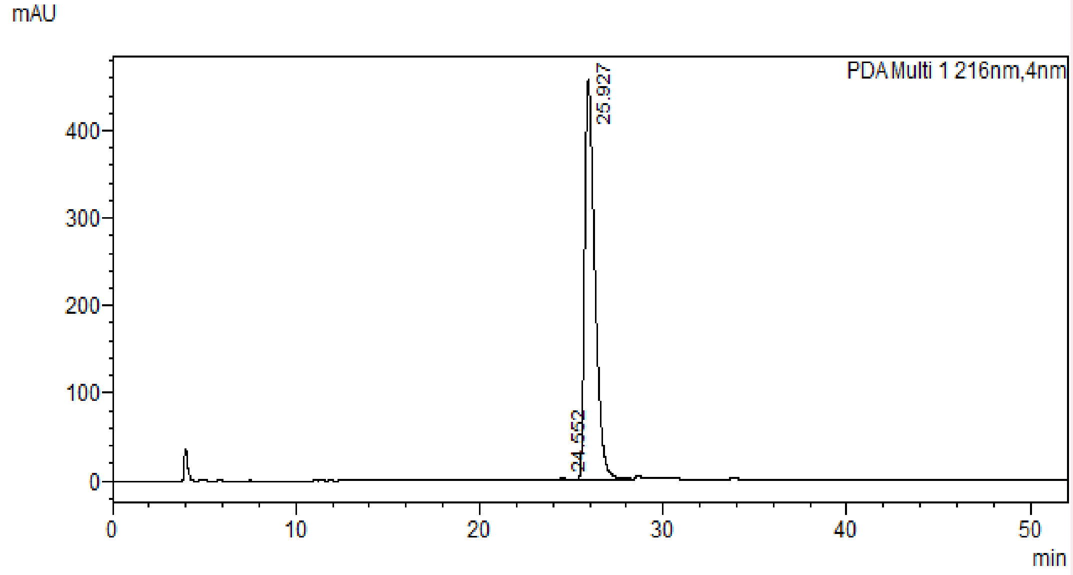

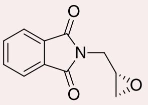

Peak Table

PDA Ch1 216nm

Peak#	Ret. Time	Area	Height	Height%	Area%
1	24.552	24158	1184	0.258	0.137
2	25.927	17661505	456932	99.742	99.863
Total		17685662	458116	100.000	100.000

HPLC analytical separation for both enantiomers of *rac*-3 performed on Shimadzu LC-40 Nexera equipped with a photodiode array detector (PAD) with older Chiralcel OD-H

HPLC conditions: *n*-hexane-*i*-PrOH (95:5, v/v); f=0.8 mL/min; λ=254 nm; *p*=3.0 MPa (Chiralcel OD-H)

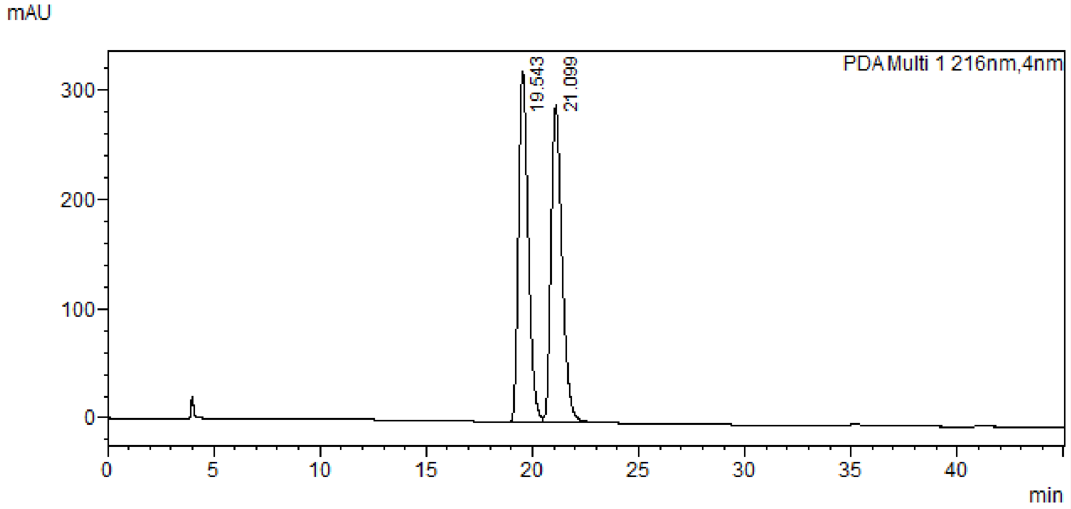

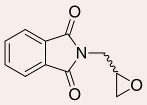

Peak Table

PDA Ch1 216nm

Peak#	Ret. Time	Area	Height	Height%	Area%
1	19.543	10299885	319968	52.543	49.674
2	21.099	10435023	288990	47.457	50.326
Total		20734907	608958	100.000	100.000

HPLC of (*S*)-(+)-3 (>99% ee) obtained from (*S*)-(–)-4 (99% ee) (yielded after inversion of stereochemistry) performed on Shimadzu LC-40 Nexera equipped with a photodiode array detector (PAD) with older Chiralcel OD-H

HPLC conditions: *n*-hexane-*i*-PrOH (95:5, v/v); f=0.8 mL/min; λ=254 nm; *p*=3.0 MPa (Chiralcel OD-H)

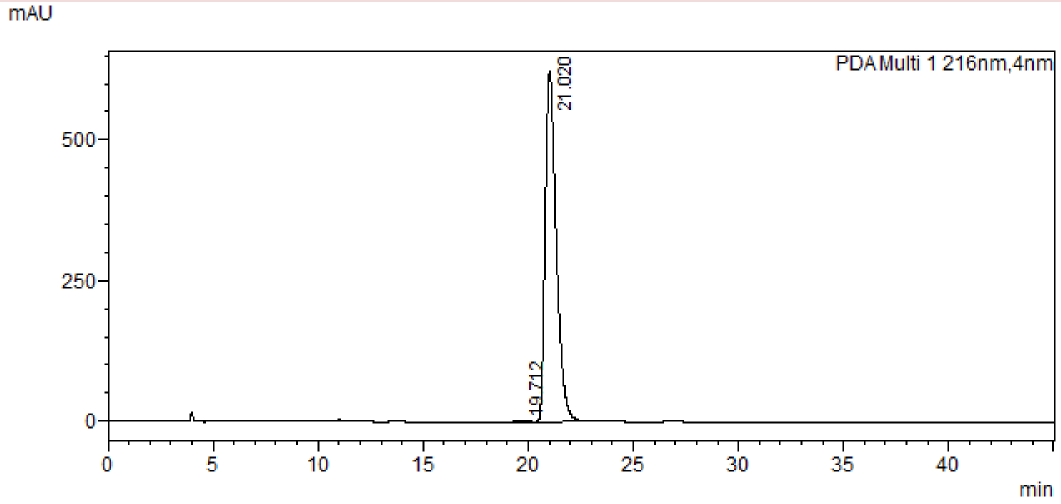

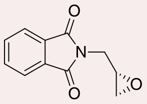

Peak Table

PDA Ch1 216nm

Peak#	Ret. Time	Area	Height	Height%	Area%
1	19.712	77761	2624	0.420	0.342
2	21.020	22675269	622265	99.580	99.658
Total		22753030	624889	100.000	100.000

HPLC analytical separation for both enantiomers of *rac*-4

HPLC conditions: *n*-hexane-*i*-PrOH (90:10, v/v); f=0.8 mL/min; λ=254 nm; *p*=3.2 MPa (Chiralcel OD-H)

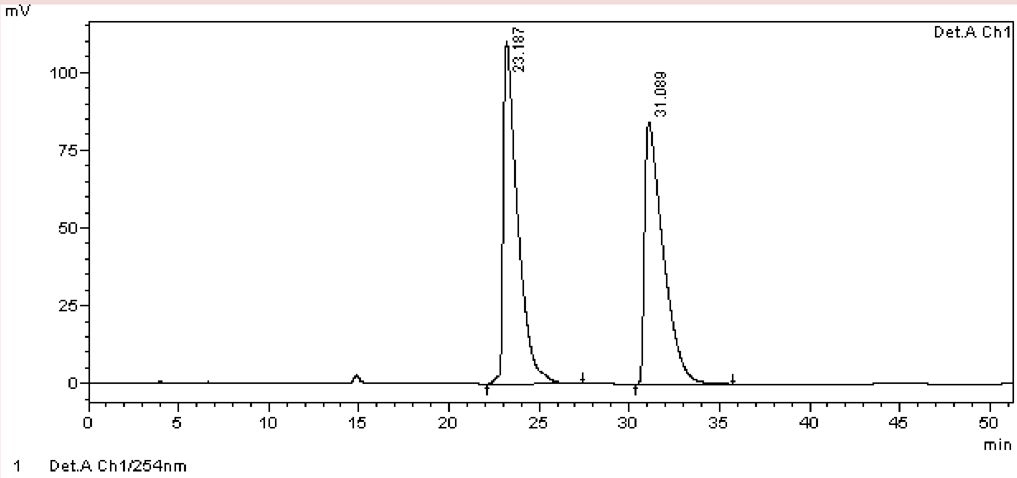

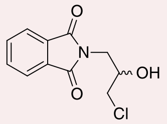

Peak Table

Detector A Ch1 254nm

Peak#	Ret. Time	Area	Height	Area %	Height %
1	23.187	6109284	110019	50.329	56.654
2	31.089	6029529	84177	49.671	43.346
Total		12138813	194196	100.000	100.000

HPLC analytical separation for both enantiomers of *rac*-5a

HPLC conditions: *n*-hexane-*i*-PrOH (90:10, v/v); f=0.8 mL/min; λ=254 nm; *p*=3.2 MPa (Chiralcel OD-H)

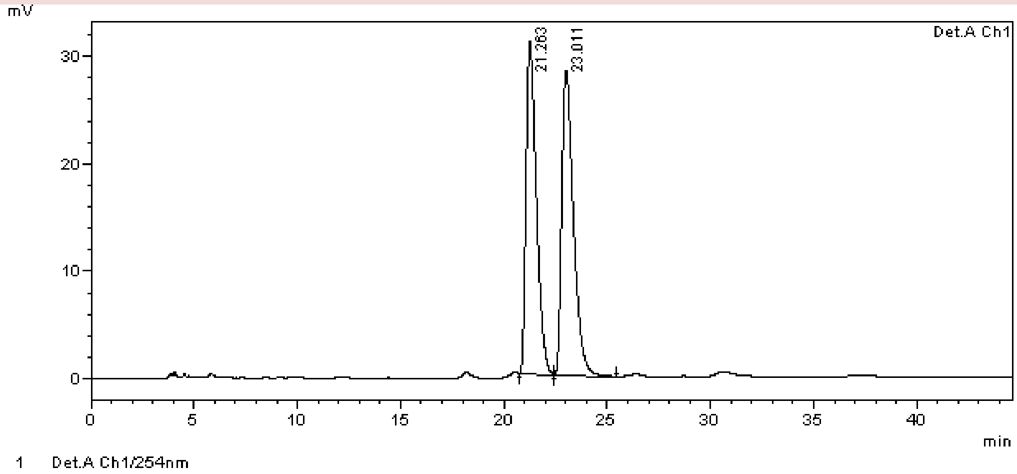

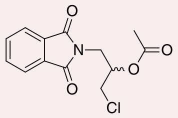

Peak Table

Detector A Ch1 254nm

Peak#	Ret. Time	Area	Height	Area %	Height %
1	21.263	1074832	30961	48.983	52.196
2	23.011	1119449	28356	51.017	47.804
Total		2194281	59317	100.000	100.000

HPLC results from Table 1. Lipase screening for the enantioselective transesterification of *rac*-4 with vinyl acetate under kinetically controlled conditions in TBME.

Lipase	HPLC of (*R*)-(+)-4	HPLC of (*S*)-(+)-5a
Novozym 435	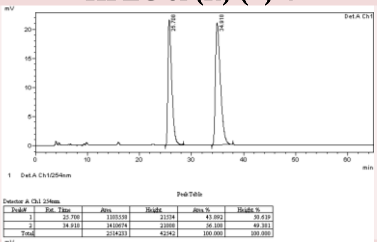	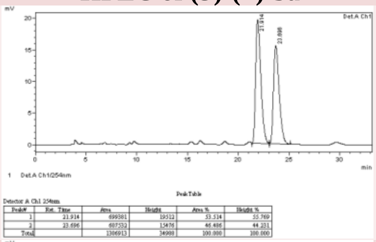
Lipozyme 435	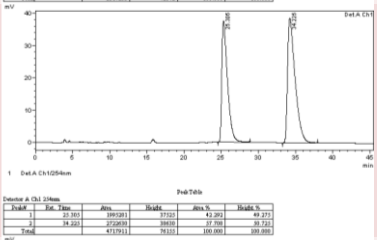	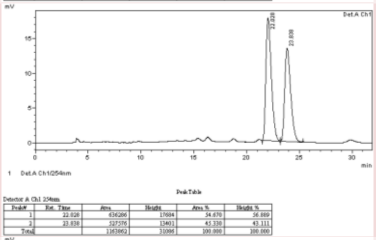
Chirazyme L-2, C-2	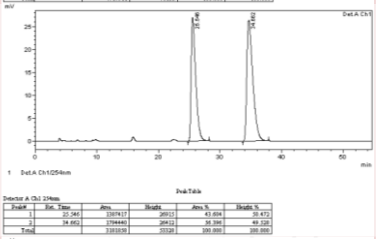	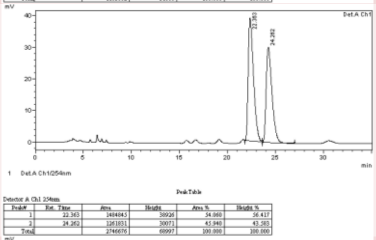
Chirazyme L-10	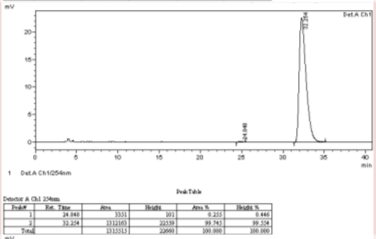	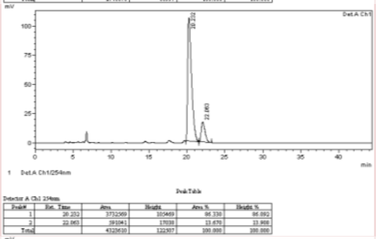
Lipozyme TL IM	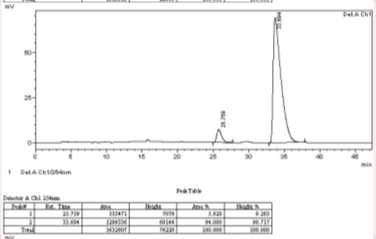	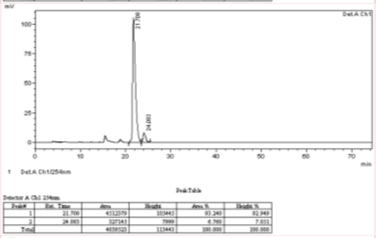
Amano PS-IM	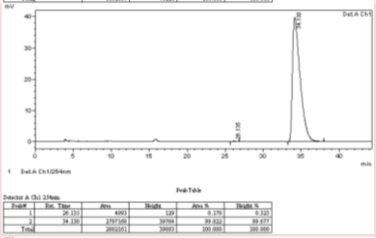	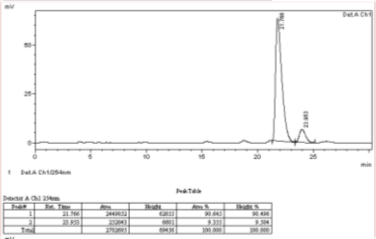
Amano PS-C II	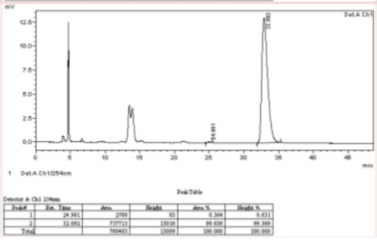	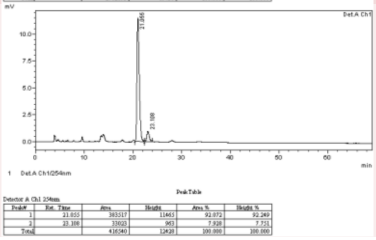

Amano AK	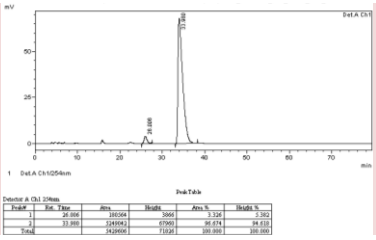	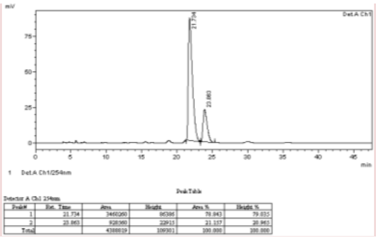

HPLC results from Table 2. Co-solvent screening for (Amano PS-IM)-catalyzed enantioselective transesterification of *rac*-4 with vinyl acetate under kinetically controlled conditions.

Co-solvent	HPLC of (*R*)-(+)-4	HPLC of (*S*)-(+)-5a
Acetone (0.20)	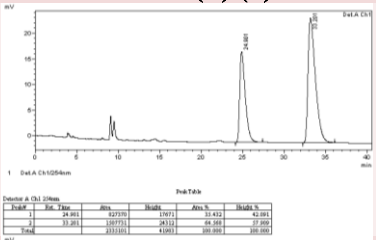	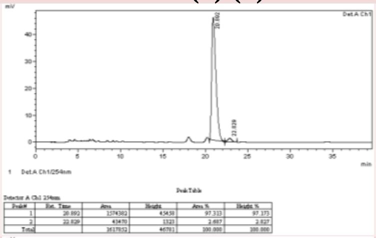
THF (0.40)	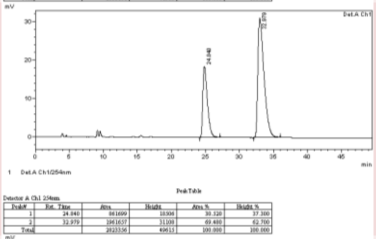	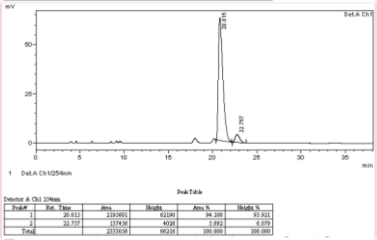
Vinyl Acetate (0.54)	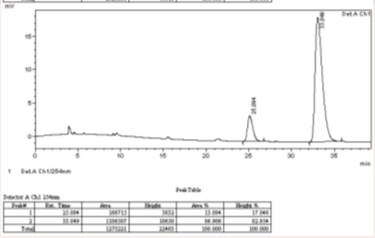	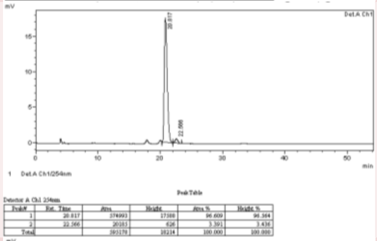
TBME (0.96)	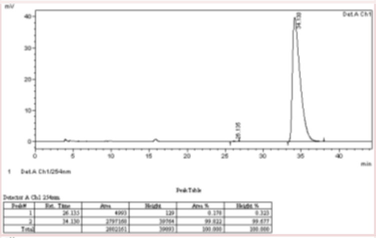	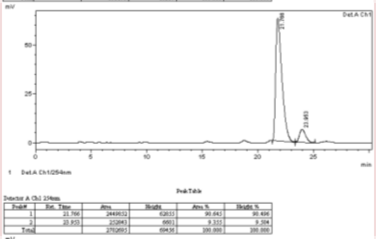
t-Amyl alcohol (1.09)	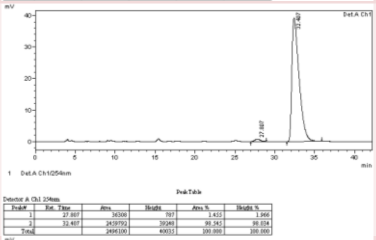	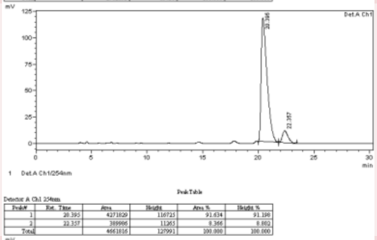
$PhCH_3$ (2.52)	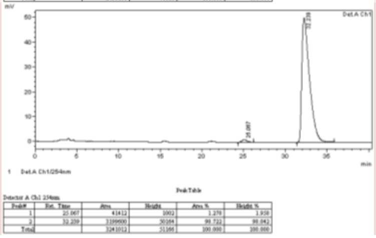	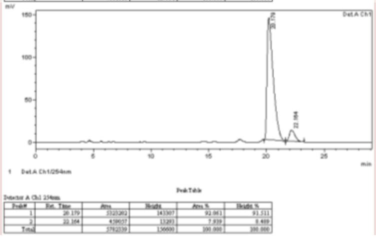

HPLC results from Table 3. Temperature effect on (Amano PS-IM)-catalyzed enantioselective transesterification of *rac*-4 with vinyl acetate under kinetically controlled conditions.

Temperature [°C]	HPLC of (*R*)-(+)-4	HPLC of (*S*)-(+)-5a
30	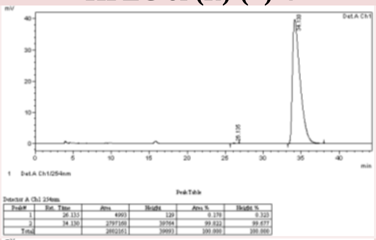	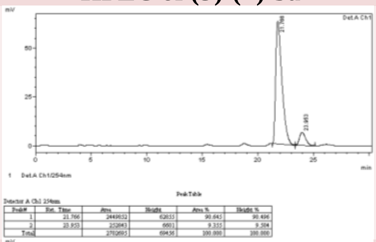
40	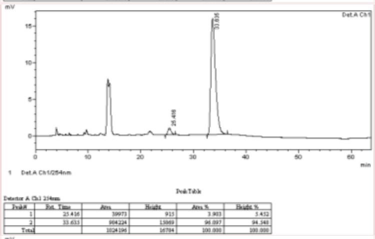	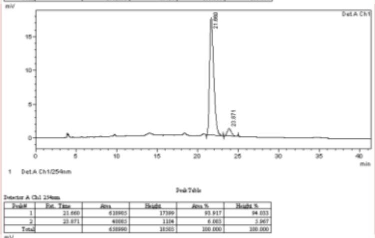
50	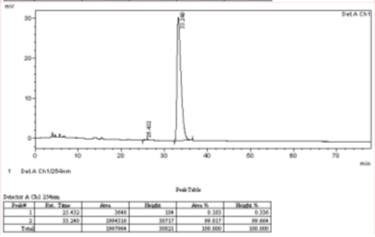	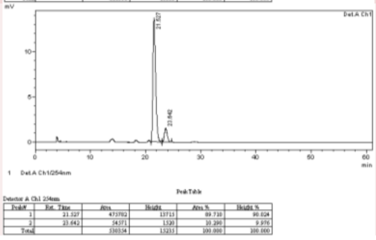

HPLC results from Fig 4. Dependence of optical purities (% ee) of KR products on the conversion degree of *rac*-4 during (Amano PS-IM)-catalyzed acetylation with vinyl acetate in a TBME solution at 50 °C.

Time [h]	HPLC of (*R*)-(+)-4	HPLC of (*S*)-(+)-5a
2	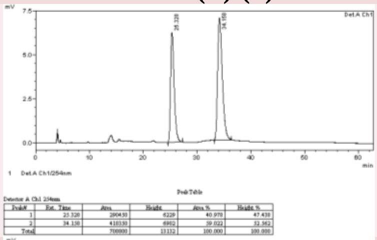	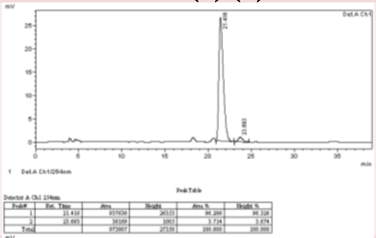
4	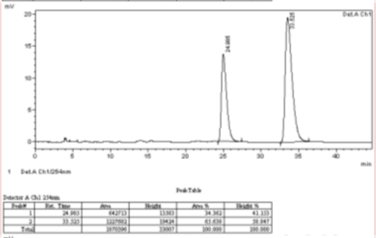	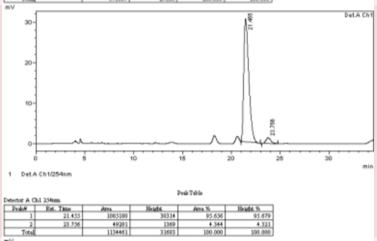
6	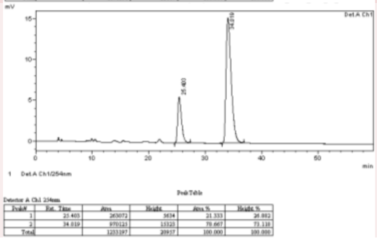	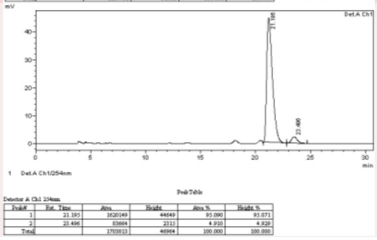

8	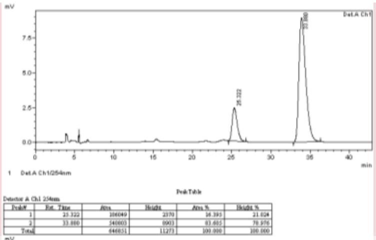	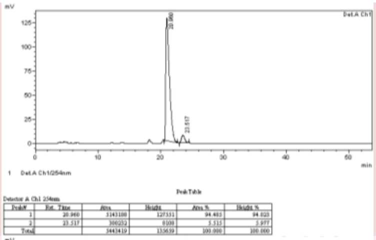
16	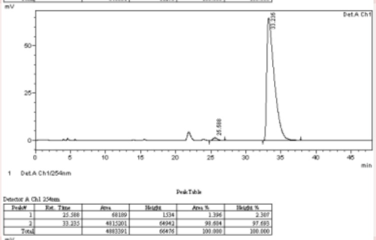	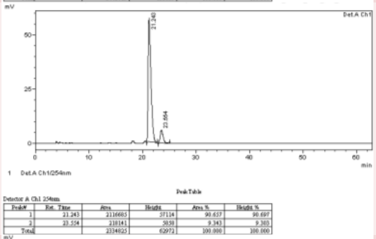
20	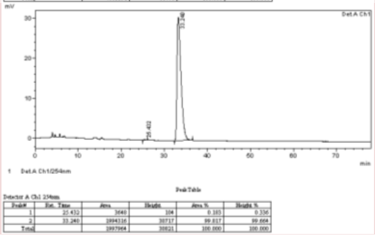	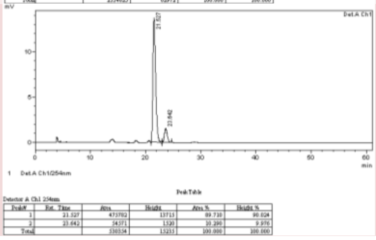

HPLC results from Table 4. Up-scaling of the KR of *rac*-4 using Amano PS-IM (25%, w/w) suspended in vinyl acetate (3 equiv) and TBME solution at 50 °C.

Scale [g] / Lipase loading [w/w] / Time [h]	HPLC of (*R*)-(+)-4	HPLC of (*S*)-(+)-5a
2.5 / 50% / 21	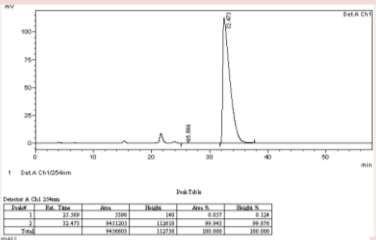	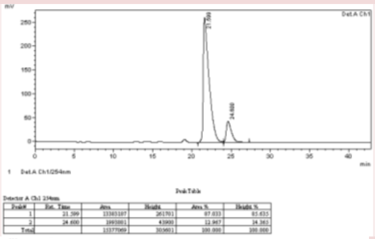
5 / 25% / 24	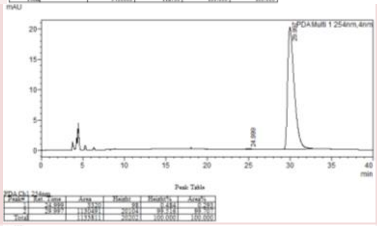	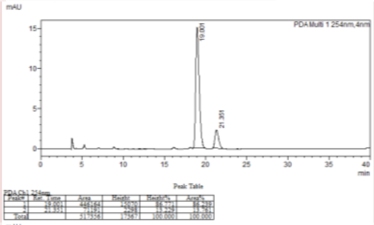
10 / 50% / 96	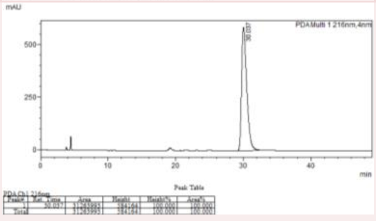	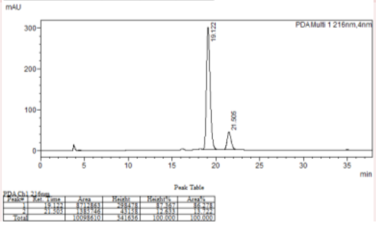

HPLC results from Table 7. An analytical-scale studies on stereoselective reduction of 2-(3-chloro-2-oxopropyl)-1*H*-isoindole-1,3(2*H*)-dione (12, 10 mM) with different biocatalysts after 48 h.

Biocatalyst	Compound	HPLC using *n*-hexane-*i*-PrOH (90:10, v/v); f=1.0 mL/min; λ=216 nm (Chiralcel OD-H)
-	*rac*-**4**	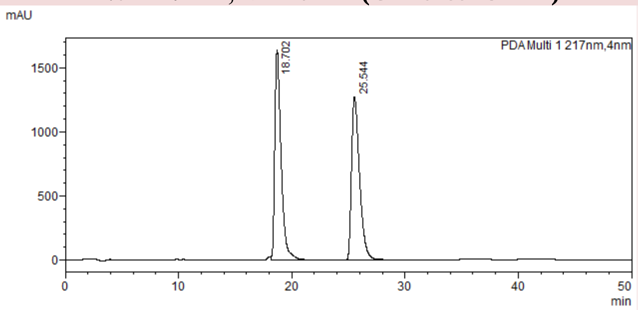
-	**12**	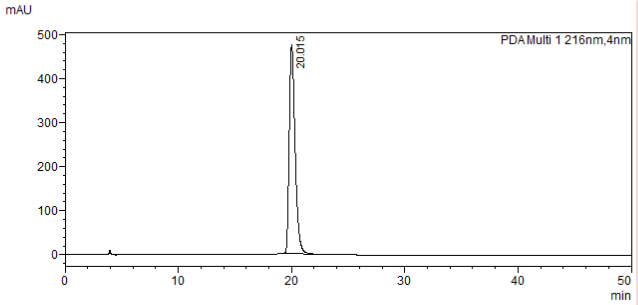
-	*rac*-**4** + **12**	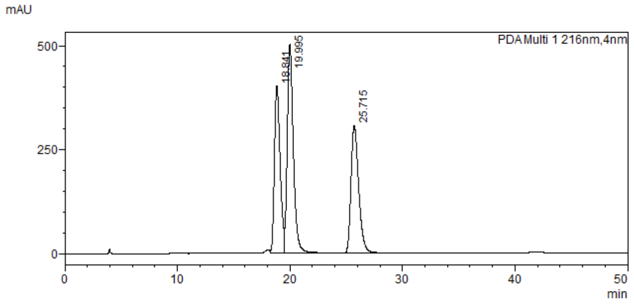
Arthrobacter sp.	(*R*)-(+)-**4**	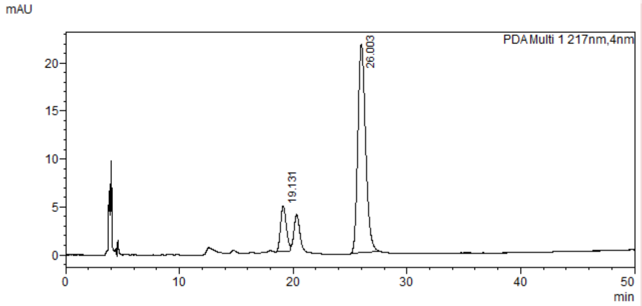

Peak Table

PDA Ch1 217nm

Peak#	Ret. Time	Area	Height	Height%	Area%
1	18.702	63504448	1636836	56.230	50.209
2	25.544	62974796	1274148	43.770	49.791
Total		126479245	2910984	100.000	100.000

Peak Table

PDA Ch1 216nm

Peak#	Ret. Time	Area	Height	Height%	Area%
1	20.015	17205806	474731	100.000	100.000
Total		17205806	474731	100.000	100.000

Peak Table

PDA Ch1 216nm

Peak#	Ret. Time	Area	Height	Height%	Area%
1	18.841	14128026	401043	33.166	29.674
2	19.995	18841570	501513	41.475	39.574
3	25.715	14641066	306641	25.359	30.752
Total		47610663	1209196	100.000	100.000

Peak Table

PDA Ch1 217nm

Peak#	Ret. Time	Area	Height	Height%	Area%
1	19.131	171190	4771	18.033	14.274
2	26.003	1028146	21686	81.967	85.726
Total		1199336	26457	100.000	100.000

isolate *Actinomyces* sp. SRB-AN040

(*R*)-(+)-**4**

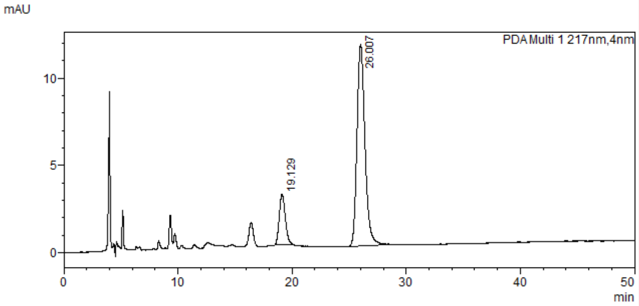

Peak Table

PDA Ch1 217nm

Peak#	Ret. Time	Area	Height	Height%	Area%
1	19.129	104671	2872	19.957	15.809
2	26.007	557423	11518	80.043	84.191
Total		662095	14390	100.000	100.000

isolate *Actinomyces* sp. ARG-AN024

(*R*)-(+)-**4**

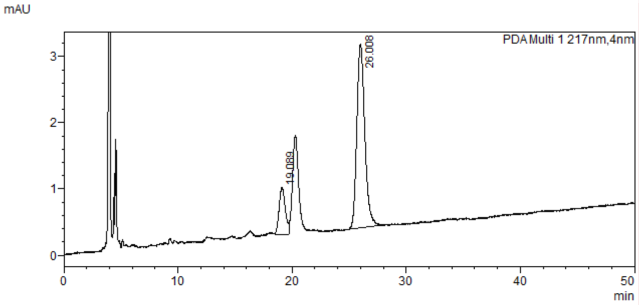

Peak Table

PDA Ch1 217nm

Peak#	Ret. Time	Area	Height	Height%	Area%
1	19.089	27928	719	20.630	17.241
2	26.008	134059	2766	79.370	82.759
Total		161987	3485	100.000	100.000

isolate ARG-AN025

(*R*)-(+)-**4**

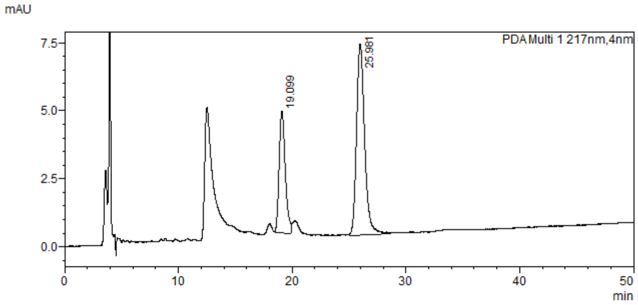

Peak Table

PDA Ch1 217nm

Peak#	Ret. Time	Area	Height	Height%	Area%
1	19.099	169072	4479	38.954	32.558
2	25.981	350230	7020	61.046	67.442
Total		519302	11499	100.000	100.000

isolate USA-AN012

(*R*)-(+)-**4**

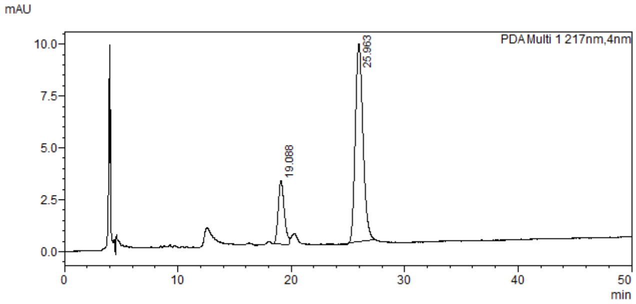

Peak Table

PDA Ch1 217nm

Peak#	Ret. Time	Area	Height	Height%	Area%
1	19.088	114119	3046	24.288	20.376
2	25.963	445938	9496	75.712	79.624
Total		560057	12542	100.000	100.000

E. coli/Lk-ADH Prince	(S)-(–)-**4**	

E. coli/Lk-ADH Prince (*S*)-(–)-**4**

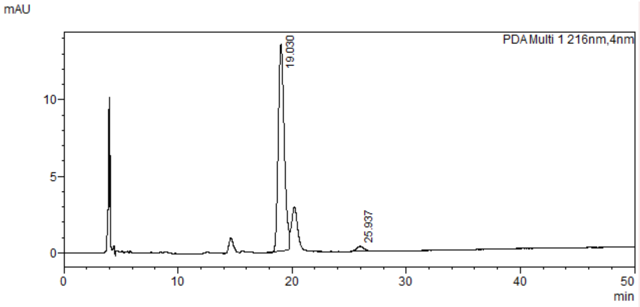

Peak Table

PDA Ch1 216nm

Peak#	Ret. Time	Area	Height	Height%	Area%
1	19.030	496082	13513	97.758	97.406
2	25.937	13213	310	2.242	2.594
Total		509295	13823	100.000	100.000

E. coli/Lk-ADH-Lica (*S*)-(–)-**4**

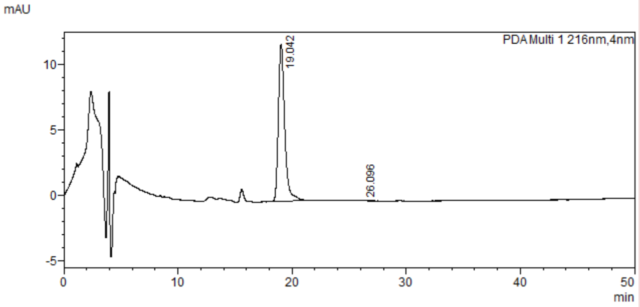

Peak Table

PDA Ch1 216nm

Peak#	Ret. Time	Area	Height	Height%	Area%
1	19.042	466842	11977	99.607	99.693
2	26.096	1437	47	0.393	0.307
Total		468279	12024	100.000	100.000

The HPLC analysis of preparative-scale *E. coli*/Lk-ADH-Lica–catalyzed bioreduction of 2-(3-chloro-2-oxopropyl)-1*H*-isoindole-1,3(2*H*)-dione (12)

HPLC analytical separation for both enantiomers of *rac*-4

HPLC conditions: *n*-hexane-*i*-PrOH (90:10, v/v); f=1.0 mL/min; λ=217 nm; *p*=3.7 MPa (Chiralcel OD-H)

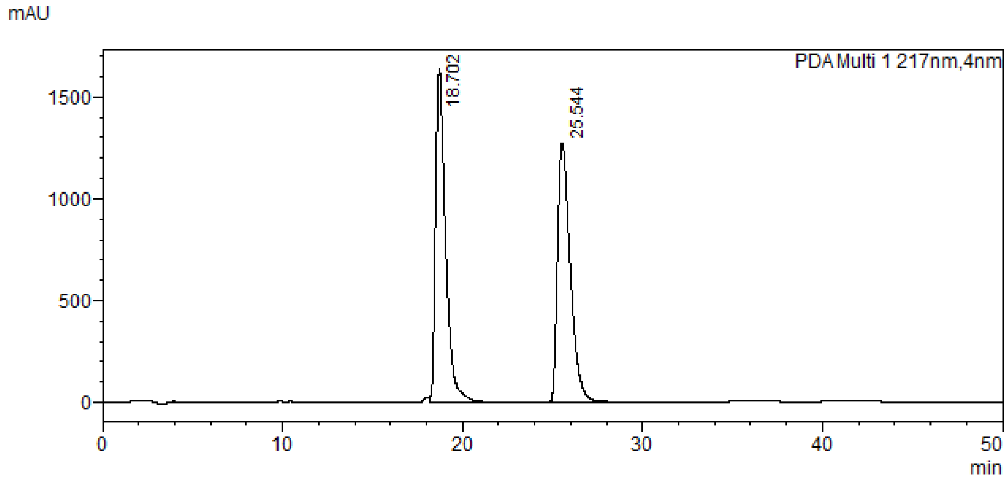

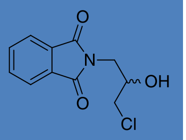

Peak Table

PDA Ch1 217nm

Peak#	Ret. Time	Area	Height	Height%	Area%
1	18.702	63504448	1636836	56.230	50.209
2	25.544	62974796	1274148	43.770	49.791
Total		126479245	2910984	100.000	100.000

HPLC analysis for the subsequent biocatalytic reaction:

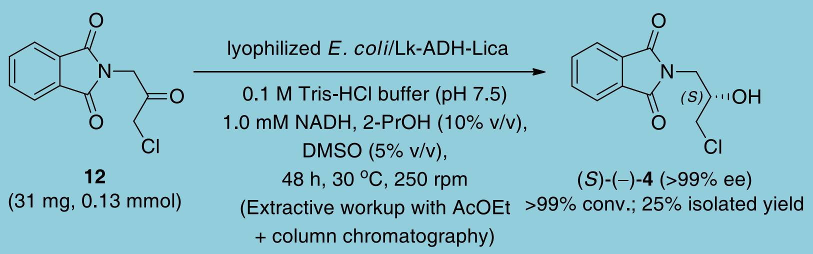

HPLC conditions: *n*-hexane-*i*-PrOH (90:10, v/v); f=1.0 mL/min; λ=217 nm; *p*=3.7 MPa (Chiralcel OD-H)

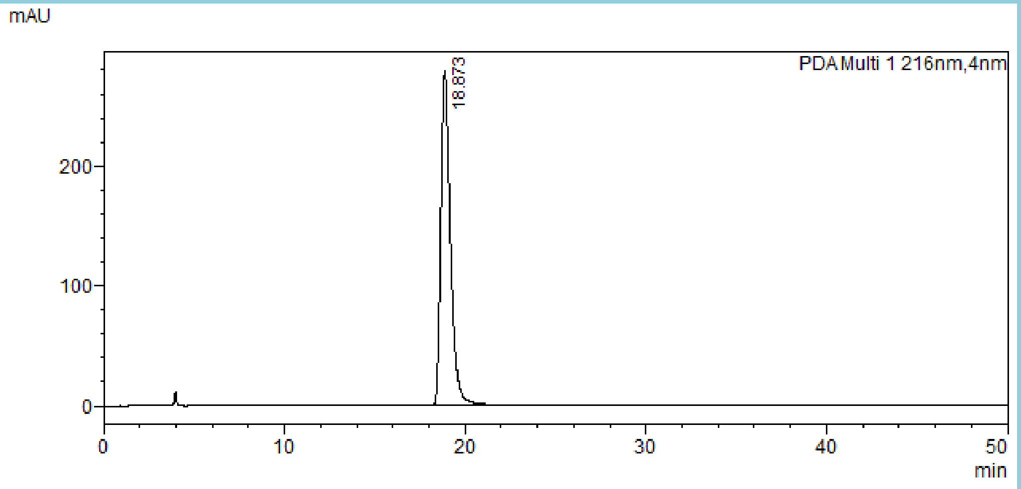

Peak Table

PDA Ch1 216nm

Peak#	Ret. Time	Area	Height	Height%	Area%
1	18.873	10285850	278570	100.000	100.000
Total		10285850	278570	100.000	100.000

HPLC analytical separation for both enantiomers of *rac*-4

HPLC conditions: *n*-hexane-*i*-PrOH (90:10, v/v); f=1.0 mL/min; λ=217 nm; *p*=3.7 MPa (Chiralcel OD-H)

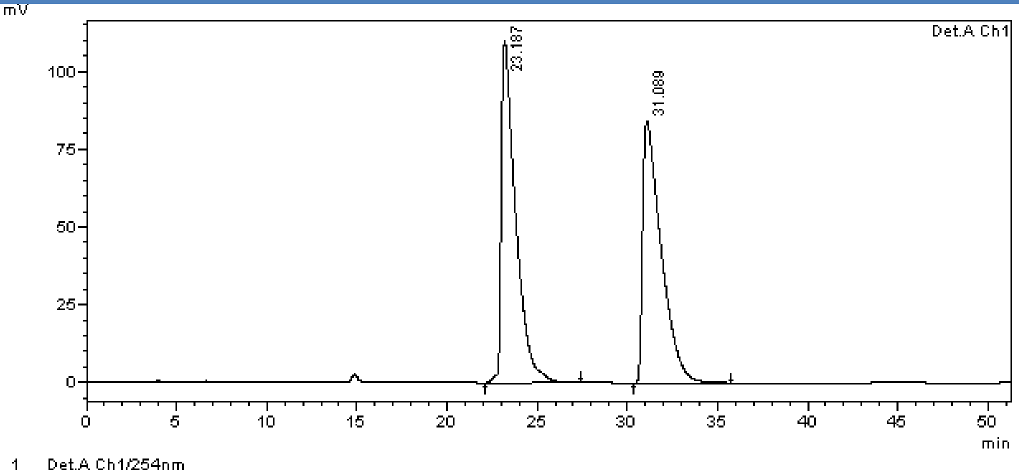

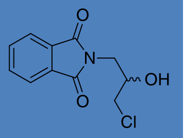

Peak Table

Detector A Ch1 254nm

Peak#	Ret. Time	Area	Height	Area %	Height %
1	23.187	6109284	110019	50.329	56.654
2	31.089	6029529	84177	49.671	43.346
Total		12138813	194196	100.000	100.000

HPLC analysis for the subsequent biocatalytic reaction:

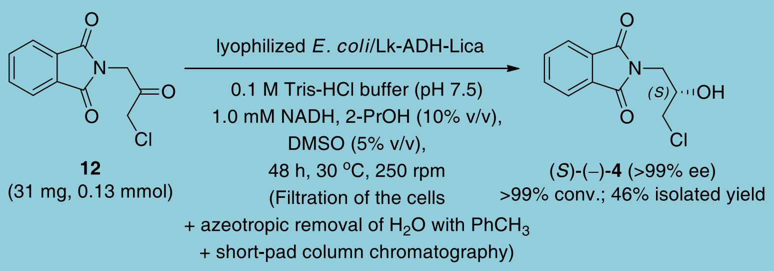

12
(31 mg, 0.13 mmol)

(*S*)-(–)-**4** (>99% ee)
>99% conv.; 46% isolated yield

HPLC conditions: *n*-hexane-*i*-PrOH (90:10, v/v); f=1.0 mL/min; λ=217 nm; *p*=3.7 MPa (Chiralcel OD-H)

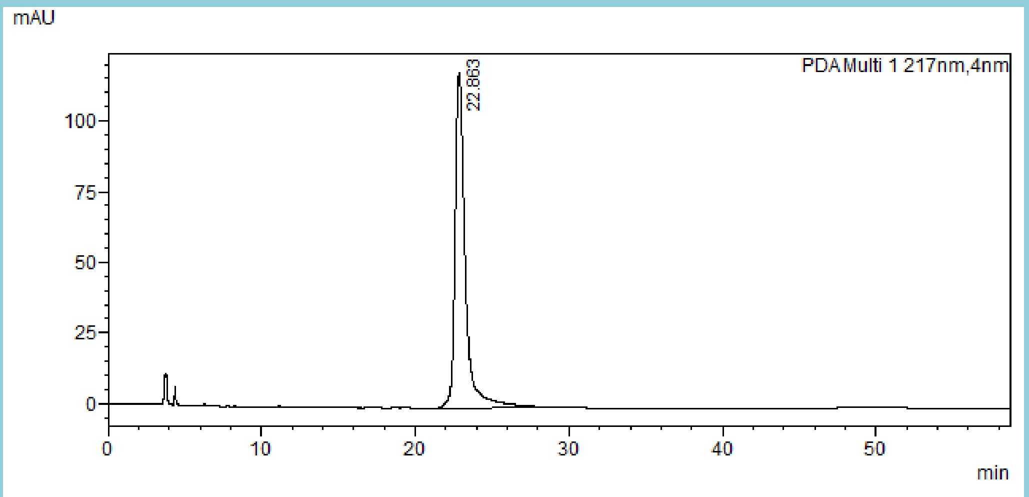

Peak Table

PDA Ch1 217nm

Peak#	Ret. Time	Area	Height	Height%	Area%
1	22.863	5517582	118411	100.000	100.000
Total		5517582	118411	100.000	100.000

HPLC analytical separation for both enantiomers of *rac*-8a (*Propranolol*)

HPLC conditions: *n*-hexane-EtOH-DEA (95:5:0.1, v/v/v); f=0.5 mL/min; λ=232 nm; *p*=1.7 MPa (Chiralcel OD-H)

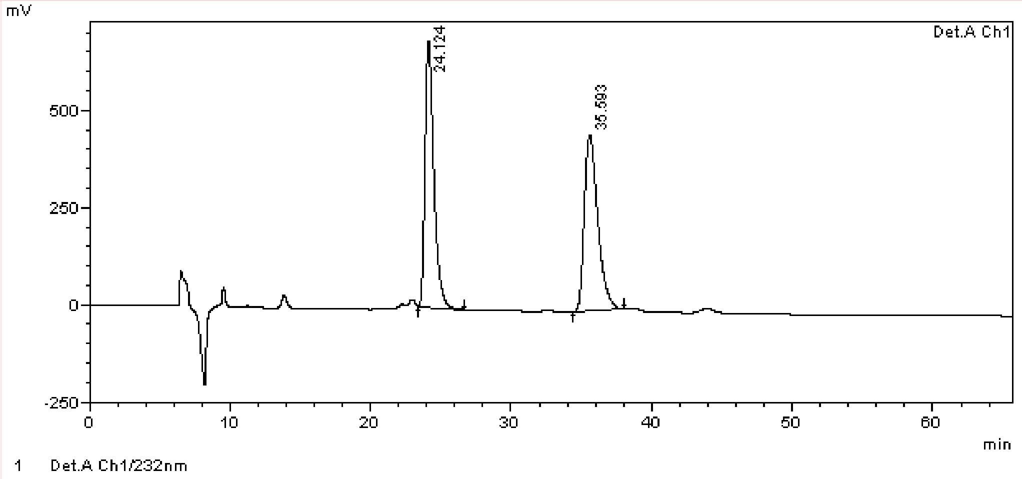

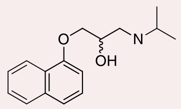

Peak Table

Detector A Ch1 232nm

Peak#	Ret. Time	Area	Height	Area %	Height %
1	24.124	29995563	682555	49.802	60.125
2	35.593	30233904	452673	50.198	39.875
Total		60229467	1135228	100.000	100.000

HPLC analytical separation for both enantiomers of (*R*)-(+)-8b (99% ee)

HPLC conditions: *n*-hexane-EtOH-DEA (95:5:0.1, v/v/v); f=0.5 mL/min; λ=232 nm; *p*=1.7 MPa (Chiralcel OD-H)

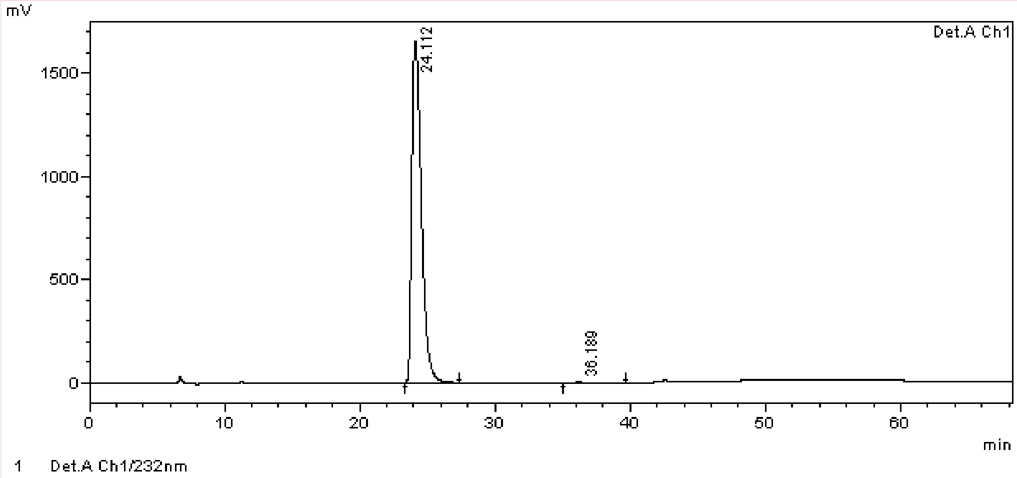

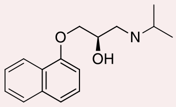

Peak Table

Detector A Ch1 232nm

Peak#	Ret. Time	Area	Height	Area %	Height %
1	24.112	81836014	1653567	99.400	99.633
2	36.189	494347	6098	0.600	0.367
Total		82330361	1659665	100.000	100.000

HPLC analytical separation for both enantiomers of *rac*-8a (*Propranolol*)

HPLC conditions: *n*-hexane-EtOH-DEA (95:5:0.1, v/v/v); f=0.5 mL/min; λ=232 nm; *p*=1.7 MPa (Chiralcel OD-H)

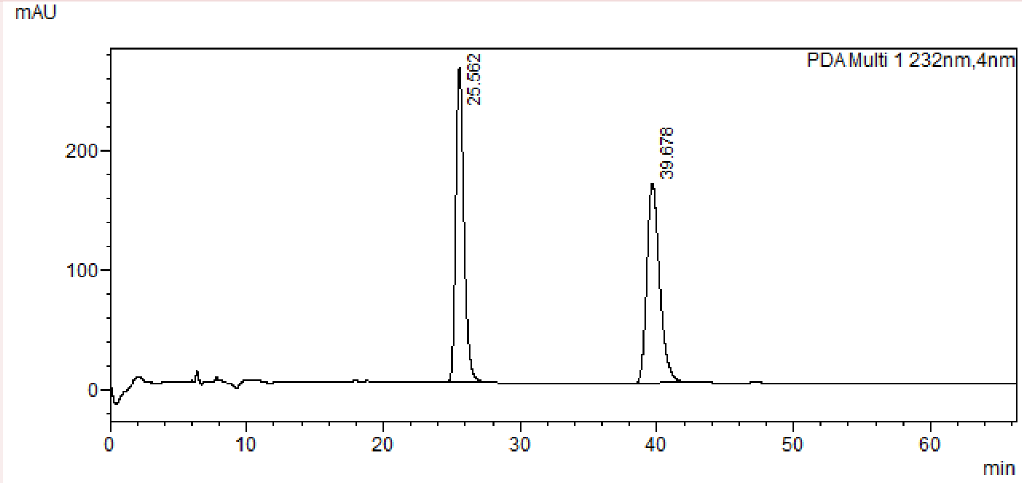

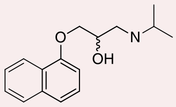

Peak Table

PDA Ch1 232nm

Peak#	Ret. Time	Area	Height	Height%	Area%
1	25.562	10933421	263037	61.177	50.136
2	39.678	10874181	166923	38.823	49.864
Total		21807602	429960	100.000	100.000

HPLC analytical separation for both enantiomers of (*S*)-(–)-8b (99% ee)

HPLC conditions: *n*-hexane-EtOH-DEA (95:5:0.1, v/v/v); f=0.5 mL/min; λ=232 nm; *p*=1.7 MPa (Chiralcel OD-H)

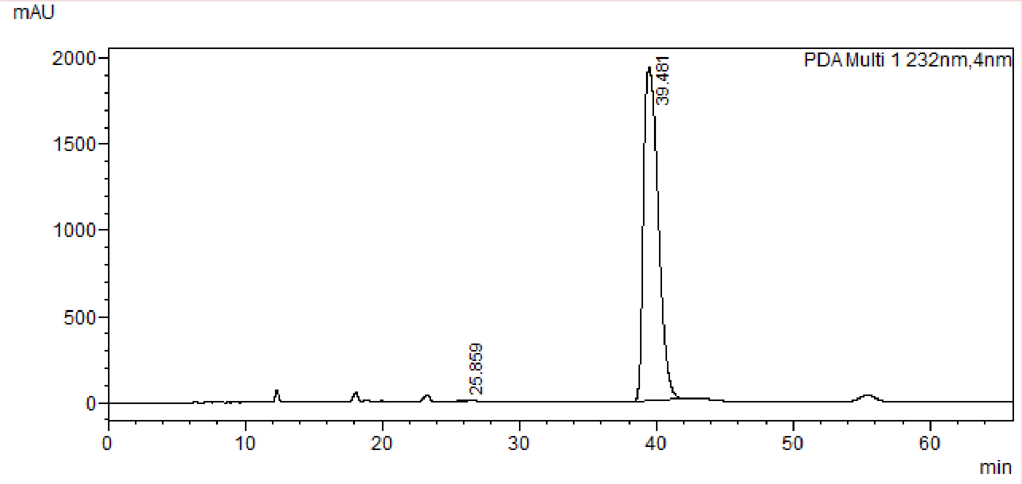

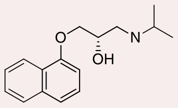

Peak Table

PDA Ch1 232nm

Peak#	Ret. Time	Area	Height	Height%	Area%
1	25.859	762859	17083	0.875	0.525
2	39.481	144641975	1935429	99.125	99.475
Total		145404833	1952511	100.000	100.000

HPLC analytical separation for both enantiomers of *rac*-8b (*Alprenolol*)

HPLC conditions: *n*-hexane-EtOH-DEA (95:5:0.1, v/v/v); f=0.5 mL/min; λ=232 nm; *p*=1.7 MPa (Chiralcel OD-H)

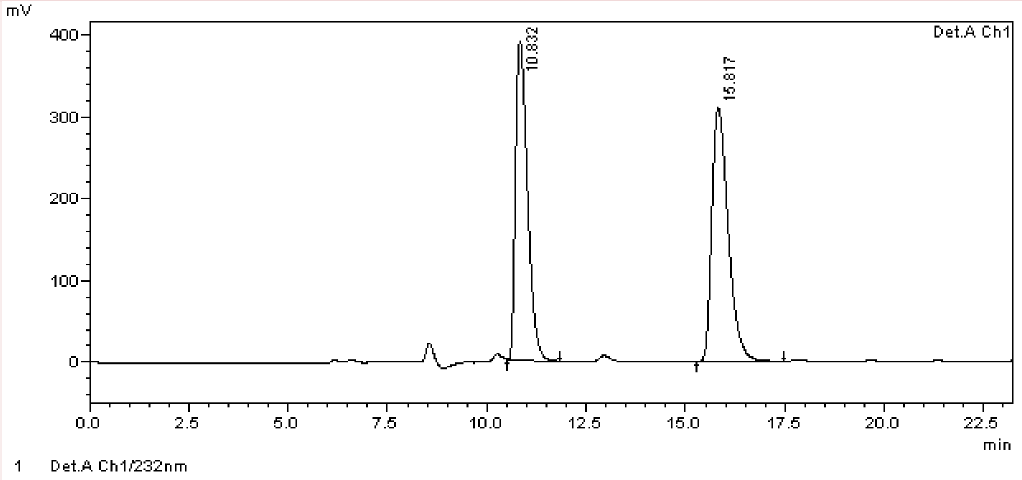

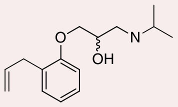

Peak Table

Detector A Ch1 232nm

Peak#	Ret. Time	Area	Height	Area %	Height %
1	10.832	7982888	388829	47.119	55.632
2	15.817	8959179	310099	52.881	44.368
Total		16942067	698929	100.000	100.000

HPLC analytical separation for both enantiomers of (*R*)-(+)-8b (96% ee)

HPLC conditions: *n*-hexane-EtOH-DEA (95:5:0.1, v/v/v); f=0.5 mL/min; λ=232 nm; *p*=1.7 MPa (Chiralcel OD-H)

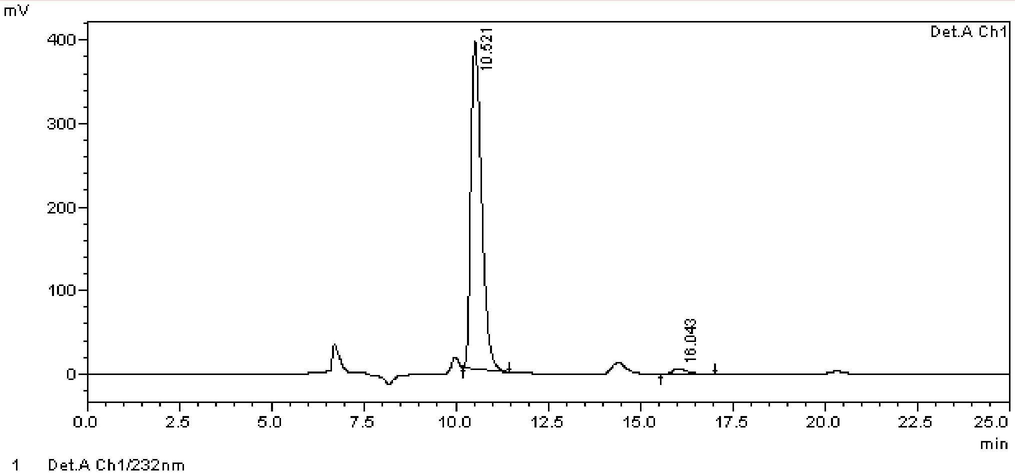

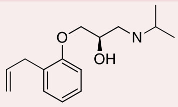

Peak Table

Detector A Ch1 232nm

Peak#	Ret. Time	Area	Height	Area %	Height %
1	10.521	7996119	391806	97.801	98.351
2	16.043	179790	6568	2.199	1.649
Total		8175909	398374	100.000	100.000

HPLC analytical separation for both enantiomers of *rac*-8c (*Pindolol*)

HPLC conditions: *n*-hexane-EtOH-DEA (80:20:0.2, v/v/v); f=1.0 mL/min; λ=264 nm; *p*=4.3 MPa (Chiralcel OD-H)

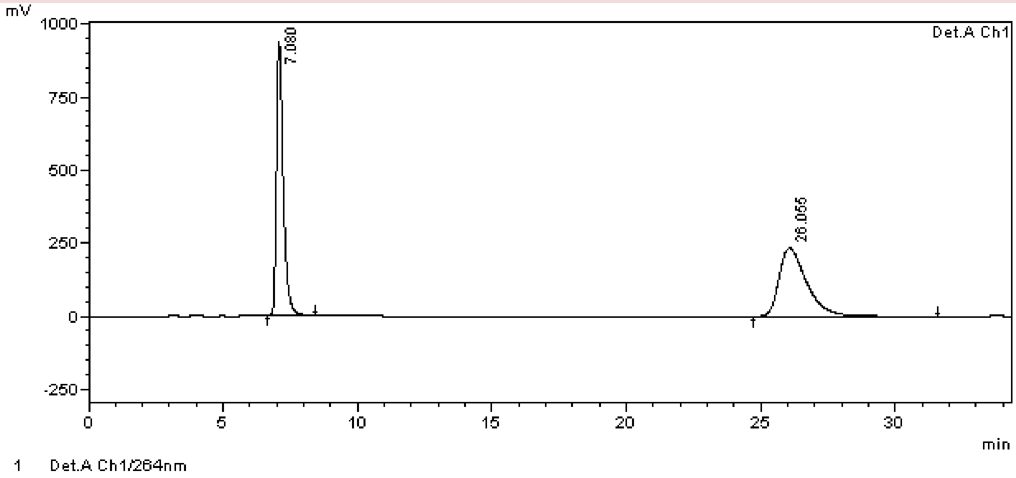

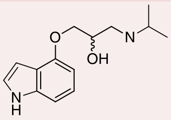

Peak Table

Detector A Ch1 264nm

Peak#	Ret. Time	Area	Height	Area %	Height %
1	7.080	16516493	937480	49.961	80.092
2	26.055	16542112	233031	50.039	19.908
Total		33058605	1170510	100.000	100.000

HPLC analytical separation for both enantiomers of (*R*)-(+)-8c (99% ee)

HPLC conditions: *n*-hexane-EtOH-DEA (95:5:0.1, v/v/v); f=0.5 mL/min; λ=264 nm; *p*=1.7 MPa (Chiralcel OD-H)

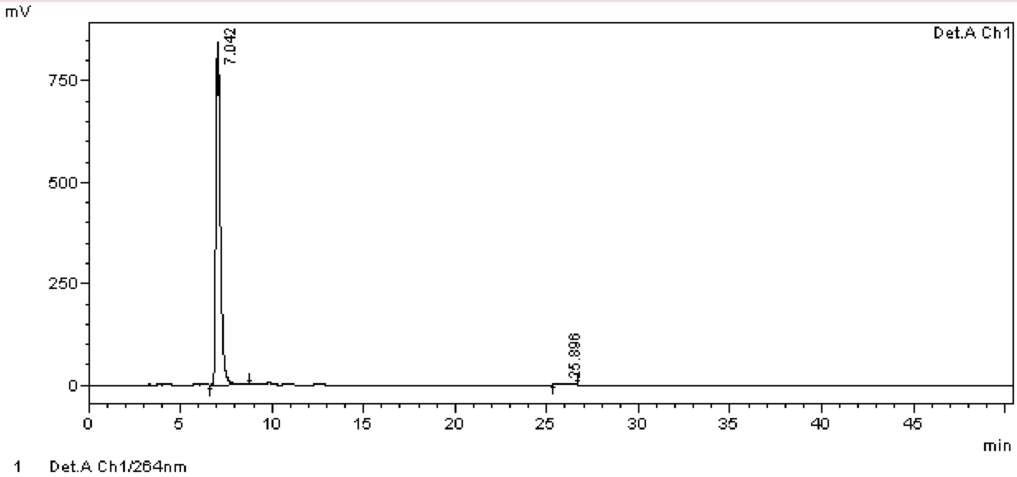

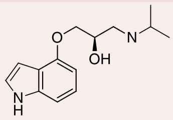

Peak Table

Detector A Ch1 264nm

Peak#	Ret. Time	Area	Height	Area %	Height %
1	7.042	14042257	844770	99.394	99.786
2	25.896	85590	1811	0.606	0.214
Total		14127848	846582	100.000	100.000

HPLC analytical separation for both enantiomers of *rac*-8d (*Carazolol*)

HPLC conditions: *n*-hexane-EtOH-DEA (80:20:0.2, v/v/v); f=0.5 mL/min; λ=220 nm; *p*=2.1 MPa (Chiralcel OD-H)

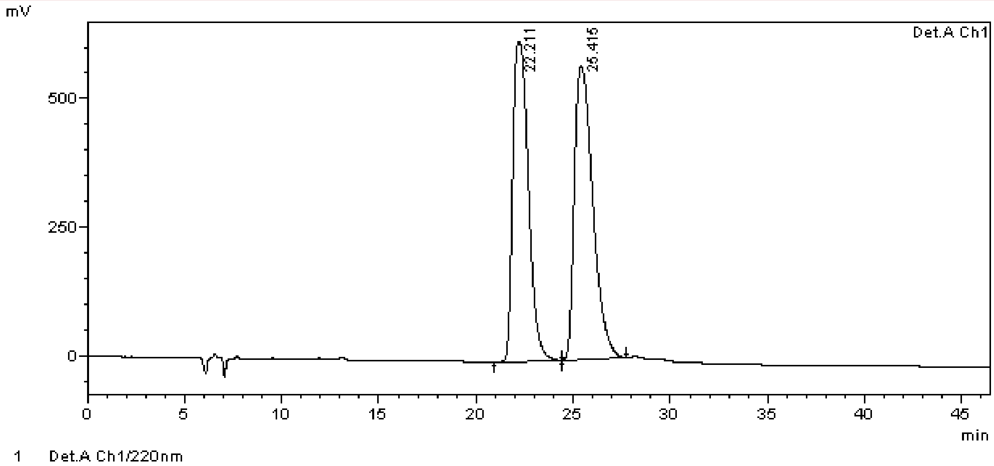

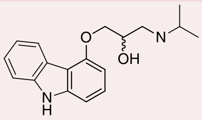

Peak Table

Detector A Ch1 220nm

Peak#	Ret. Time	Area	Height	Area %	Height %
1	22.211	34910875	621504	47.993	52.152
2	25.415	37830611	570223	52.007	47.848
Total		72741486	1191728	100.000	100.000

HPLC analytical separation for both enantiomers of (*R*)-(+)-8d (97% ee)

HPLC conditions: *n*-hexane-EtOH-DEA (80:20:0.2, v/v/v); f=0.5 mL/min; λ=220 nm; *p*=2.1 MPa (Chiralcel OD-H)

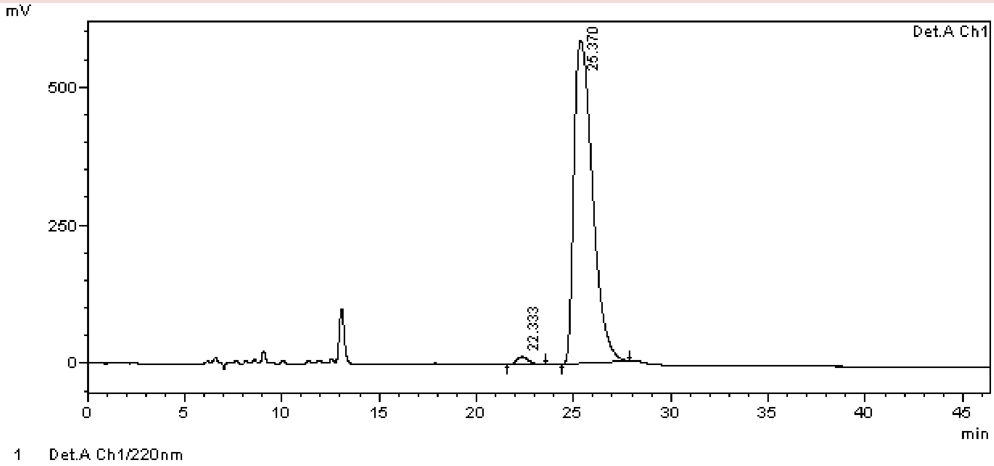

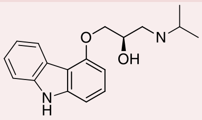

Peak Table

Detector A Ch1 220nm

Peak#	Ret. Time	Area	Height	Area %	Height %
1	22.333	639010	14605	1.611	2.437
2	25.370	39032092	584669	98.389	97.563
Total		39671102	599274	100.000	100.000

HPLC analytical separation for both enantiomers of *rac*-8e (*Moprolol*)

HPLC conditions: *n*-hexane-EtOH-DEA (90:10:0.1, v/v/v); f=1.2 mL/min; λ=220 nm; *p*=5.4 MPa (Chiralcel OD-H)

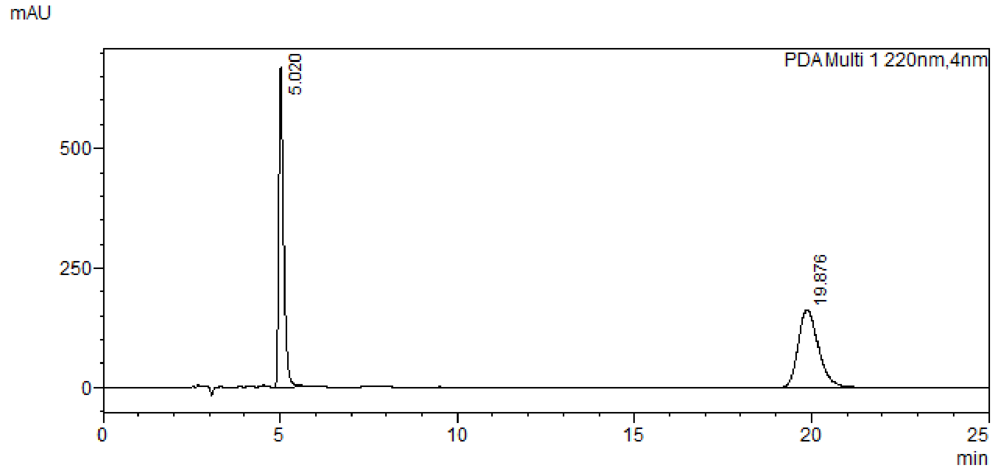

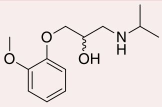

Peak Table

PDA Ch1 220nm

Peak#	Ret. Time	Area	Height	Height%	Area%
1	5.020	6321149	668295	80.445	49.438
2	19.876	6464783	162457	19.555	50.562
Total		12785932	830752	100.000	100.000

HPLC analytical separation for both enantiomers of (*R*)-(+)-8e (>99% ee)

HPLC conditions: *n*-hexane-EtOH-DEA (90:10:0.1, v/v/v); f=1.2 mL/min; λ=220 nm; *p*=5.4 MPa (Chiralcel OD-H)

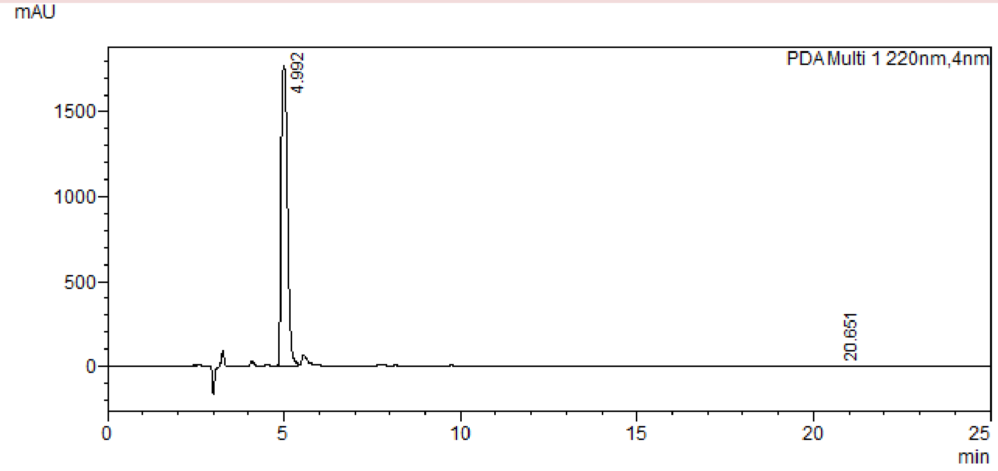

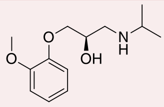

Peak Table

PDA Ch1 220nm

Peak#	Ret. Time	Area	Height	Height%	Area%
1	4.992	22053385	1766019	99.877	99.589
2	20.651	91040	2181	0.123	0.411
Total		22144425	1768201	100.000	100.000

HPLC analytical separation for both enantiomers of *rac*-8f (*Metoprolol*)

HPLC conditions: *n*-hexane-EtOH-DEA (90:10:0.1, v/v/v); f=1.2 mL/min; λ=220 nm; *p*=5.4 MPa (Chiralcel OD-H)

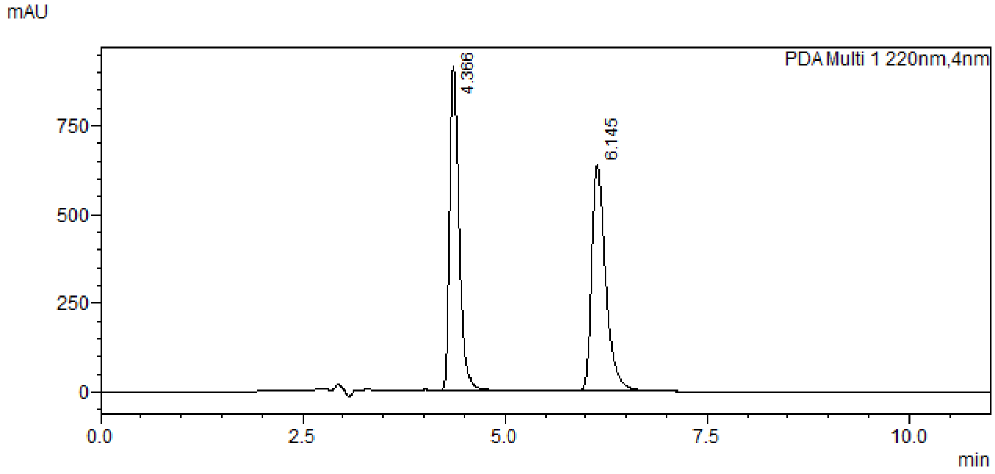

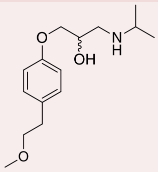

Peak Table

PDA Ch1 220nm

Peak#	Ret. Time	Area	Height	Height%	Area%
1	4.366	7379904	910613	58.931	49.023
2	6.145	7673922	634605	41.069	50.977
Total		15053827	1545218	100.000	100.000

HPLC analytical separation for both enantiomers of (*R*)-(+)-8f (94% ee)

HPLC conditions: *n*-hexane-EtOH-DEA (90:10:0.1, v/v/v); f=1.2 mL/min; λ=220 nm; *p*=5.4 MPa (Chiralcel OD-H)

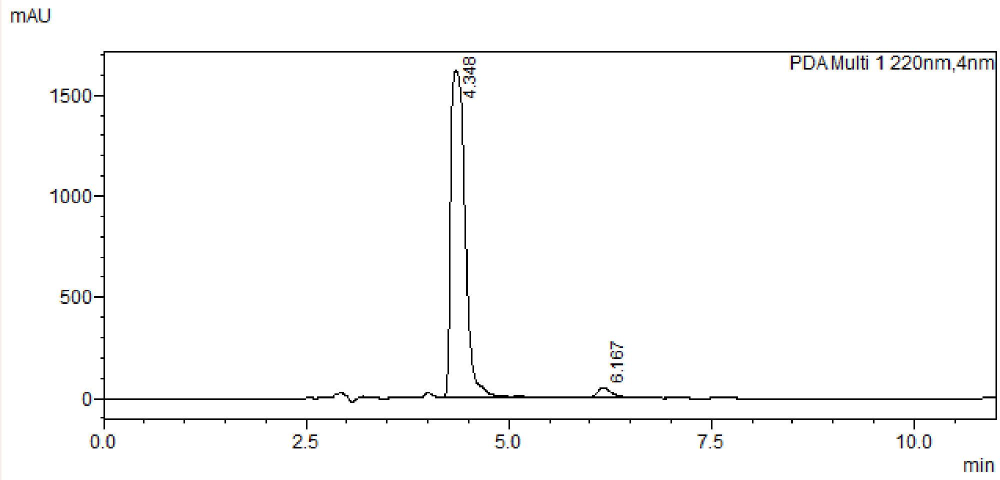

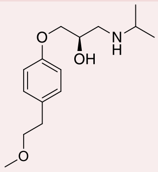

Peak Table

PDA Ch1 220nm

Peak#	Ret. Time	Area	Height	Height%	Area%
1	4.348	20080709	1616812	96.888	96.930
2	6.167	635926	51936	3.112	3.070
Total		20716635	1668748	100.000	100.000

HPLC analytical separation for both enantiomers of *rac*-10

HPLC conditions: *n*-hexane-*i*-PrOH (90:10, v/v); f=0.8 mL/min; λ=219 nm; *p*=3.2 MPa (Chiralcel OD-H)

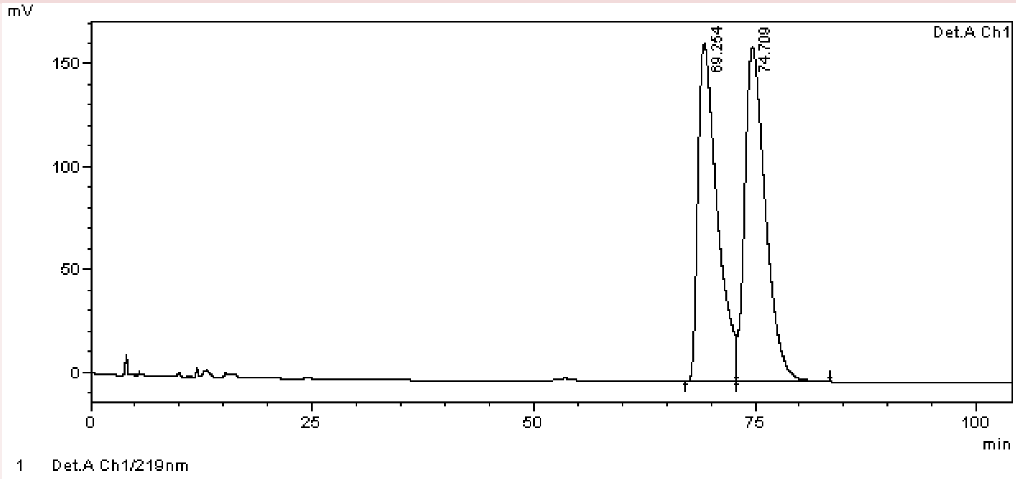

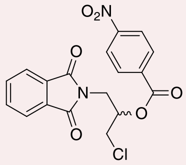

Peak Table

Detector A Ch1 219nm

Peak#	Ret. Time	Area	Height	Area %	Height %
1	69.254	24762184	164104	48.320	50.186
2	74.709	26484321	162888	51.680	49.814
Total		51246506	326992	100.000	100.000

HPLC of (*S*)-10 (18% ee) obtained from (*R*)-(+)-4 (>99% ee) [the reaction conducted at 100 mg-scale]

HPLC conditions: *n*-hexane-*i*-PrOH (90:10, v/v); f=0.8 mL/min; λ=219 nm; *p*=3.2 MPa (Chiralcel OD-H)

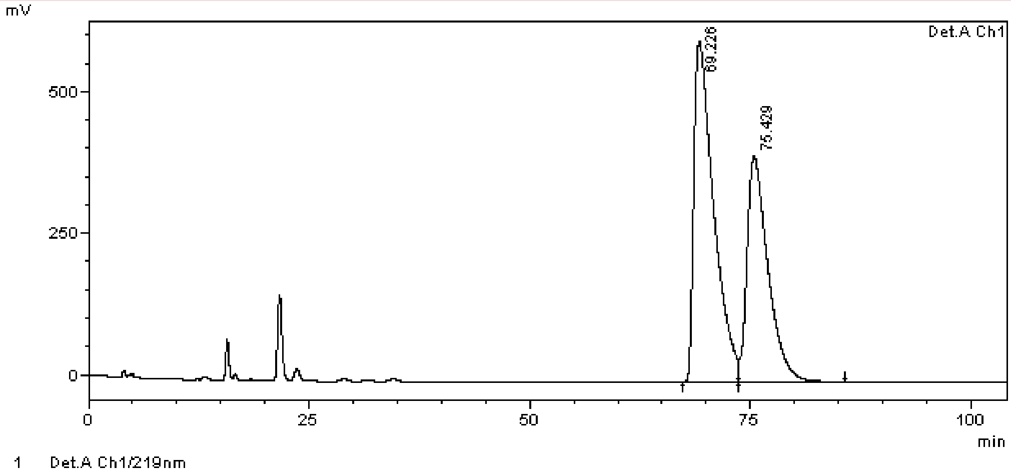

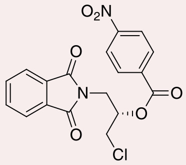

Peak Table

Detector A Ch1 219nm

Peak#	Ret. Time	Area	Height	Area %	Height %
1	69.226	94075899	601427	58.873	60.159
2	75.429	65718708	398299	41.127	39.841
Total		159794606	999726	100.000	100.000

HPLC analytical separation for both enantiomers of *rac*-11

HPLC conditions: *n*-hexane-*i*-PrOH (78:22, v/v); f=1.0 mL/min; λ=217 nm; *p*=4.7 MPa (Chiralpak AD-H)

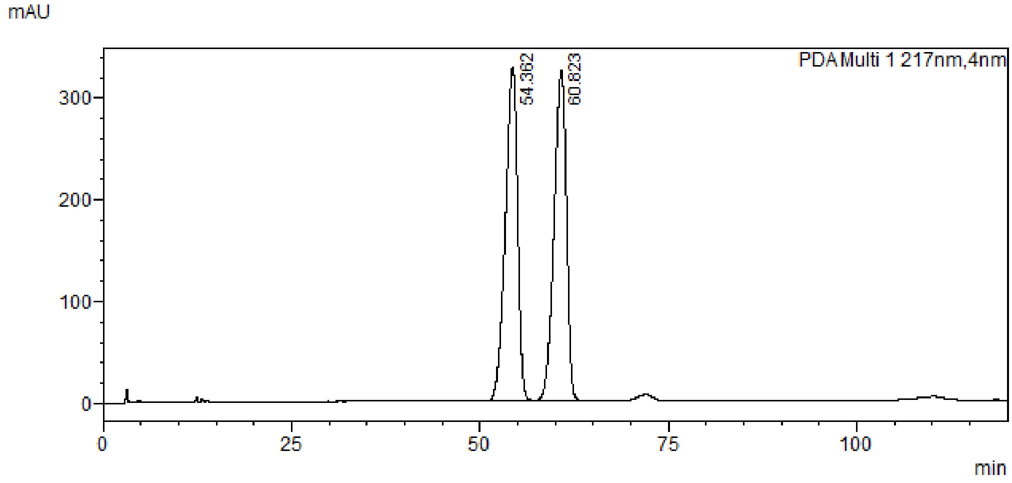

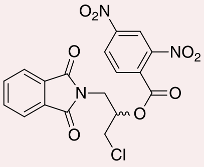

Peak Table

PDA Ch1 217nm

Peak#	Ret. Time	Area	Height	Height%	Area%
1	54.362	36960884	327205	50.204	49.995
2	60.823	36968800	324550	49.796	50.005
Total		73929684	651755	100.000	100.000

HPLC of (*S*)-11 (81% ee) obtained from (*R*)-(+)-4 (>99% ee) [the reaction conducted at 239 mg-scale (1.0 mmol)]

HPLC conditions: *n*-hexane-*i*-PrOH (78:22, v/v); f=1.0 mL/min; λ=217 nm; *p*=4.7 MPa (Chiralpak AD-H)

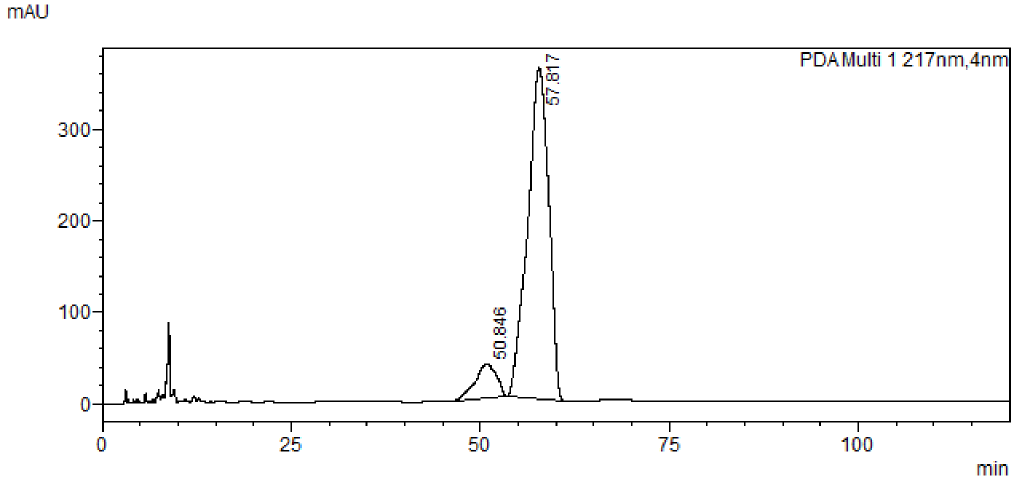

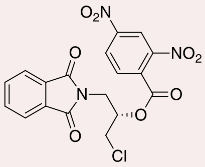

Peak Table

PDA Ch1 217nm

Peak#	Ret. Time	Area	Height	Height%	Area%
1	50.846	7267415	36918	9.257	9.330
2	57.817	70625769	361894	90.743	90.670
Total		77893183	398812	100.000	100.000

HPLC analytical separation for both enantiomers of *rac*-9

HPLC conditions: *n*-hexane-*i*-PrOH (85:15, v/v); f=0.8 mL/min; λ=219 nm; *p*=3.3 MPa (Chiralcel OD-H)

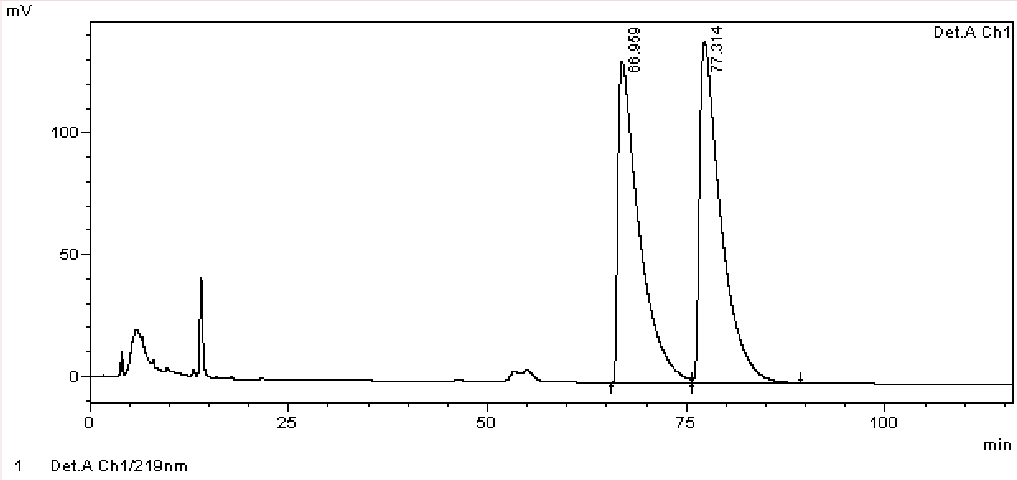

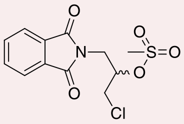

Peak Table

Detector A Ch1 219nm

Peak#	Ret. Time	Area	Height	Area %	Height %
1	66.959	24340658	132061	48.294	48.537
2	77.314	26060048	140020	51.706	51.463
Total		50400706	272082	100.000	100.000

HPLC of (*R*)-(+)-9 (>99% ee) obtained from (*R*)-(+)-4 (>99% ee) [the reaction conducted at 100 mg-scale]

HPLC conditions: *n*-hexane-*i*-PrOH (85:15, v/v); f=0.8 mL/min; λ=219 nm; *p*=3.3 MPa (Chiralcel OD-H)

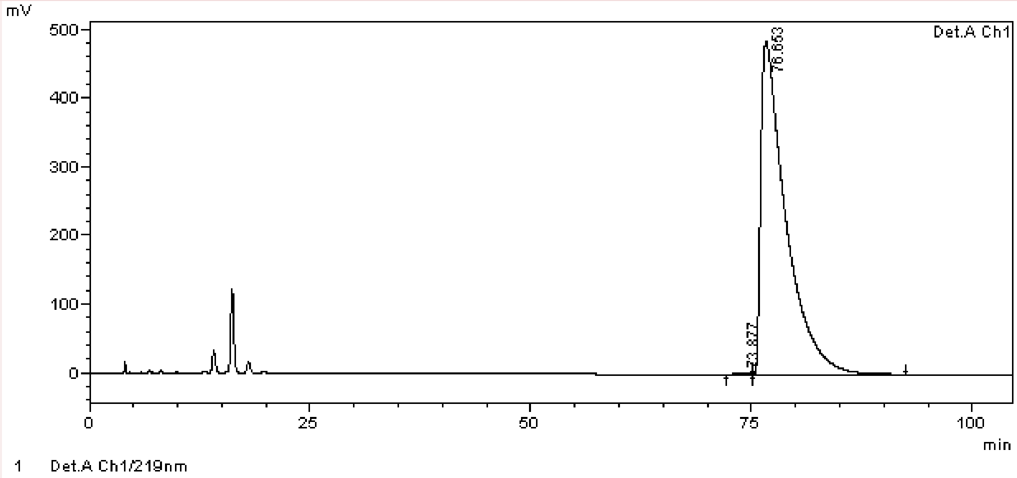

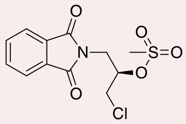

Peak Table

Detector A Ch1 219nm

Peak#	Ret. Time	Area	Height	Area %	Height %
1	73.877	85873	760	0.090	0.156
2	76.653	95149483	485285	99.910	99.844
Total		95235356	486045	100.000	100.000

HPLC analytical separation for both enantiomers of *rac*-9

HPLC conditions: *n*-hexane-*i*-PrOH (90:10, v/v); f=1.0 mL/min; λ=216 nm; *p*=3.6 MPa (Chiralcpak AD-H)

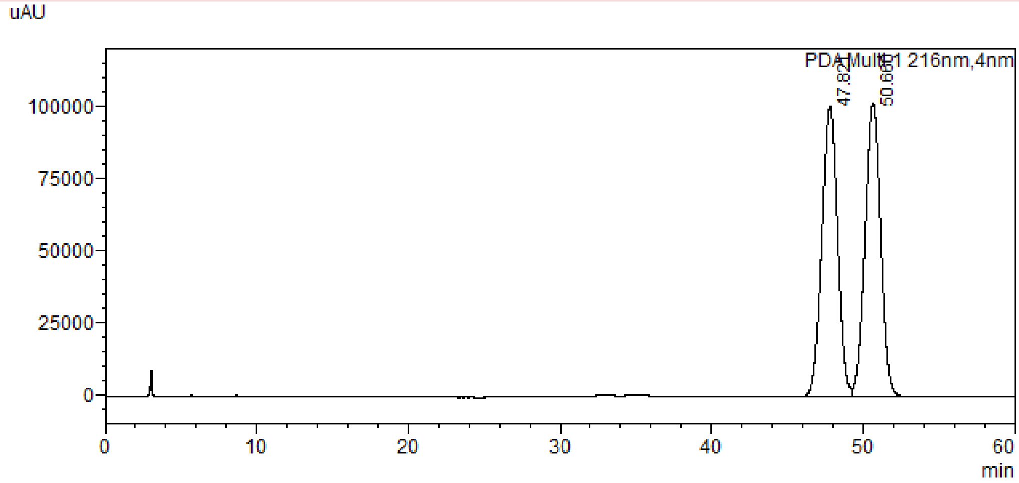

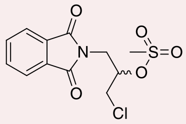

<Peak Table>

PDA Ch1 216nm

Peak#	Ret. Time	Area	Height	Conc.	Area%
1	47.821	7247893	100704	0.000	49.920
2	50.660	7271162	101670	0.000	50.080
Total		14519055	202373		100.000

HPLC of (*R*)-(+)-9 (>99% ee) obtained from (*R*)-(+)-4 (>99% ee) [the reaction conducted at 1 g-scale]

HPLC conditions: *n*-hexane-*i*-PrOH (90:10, v/v); f=1.0 mL/min; λ=216 nm; *p*=3.6 MPa (Chiralpak AD-H)

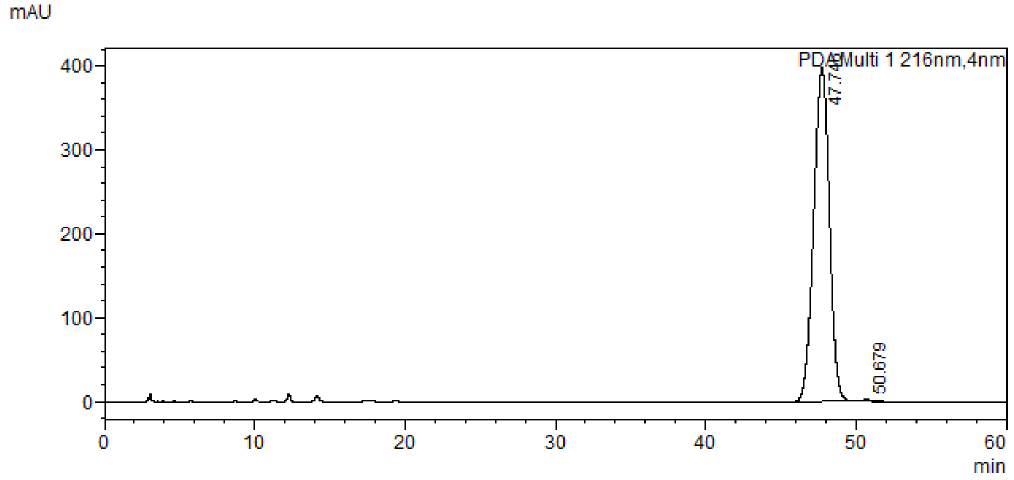

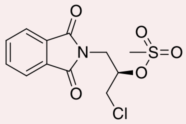

<Peak Table>

PDA Ch1 216nm

Peak#	Ret. Time	Area	Height	Conc.	Area%
1	47.746	28851572	397601	0.000	99.738
2	50.679	75808	1252	0.000	0.262
Total		28927380	398853		100.000

HPLC analytical separation for both enantiomers of *rac*-5a

HPLC conditions: *n*-hexane-*i*-PrOH (90:10, v/v); f=0.8 mL/min; λ=254 nm; *p*=3.2 MPa (Chiralcel OD-H)

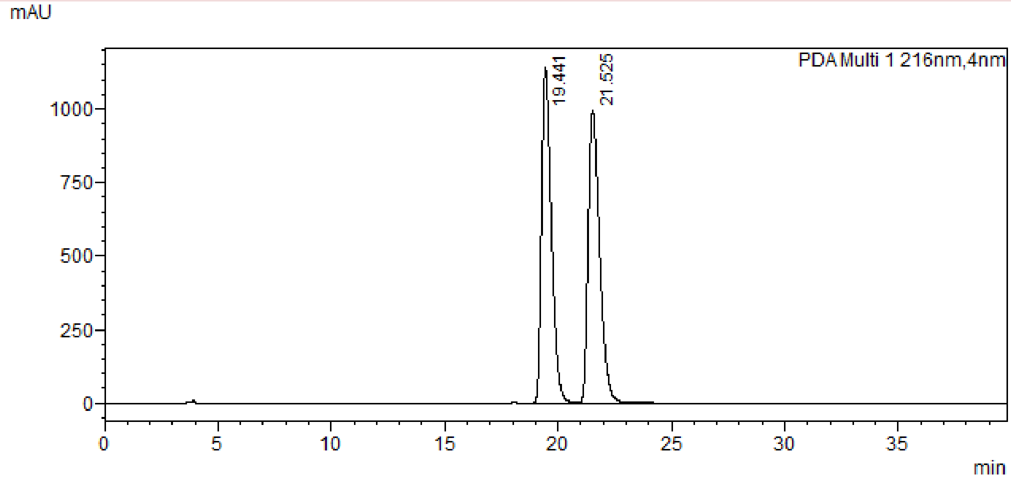

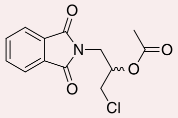

<Peak Table>

PDA Ch1 216nm

Peak#	Ret. Time	Area	Height	Conc.	Area%
1	19.441	34678792	1140514	0.000	49.808
2	21.525	34945984	997817	0.000	50.192
Total		69624776	2138332		100.000

HPLC of (*S*)-(+)-5a (>99% ee) obtained from (*R*)-(+)-9 (>99% ee) (yielded after inversion of stereochemistry) after treatment with AcOCs

HPLC conditions: *n*-hexane-*i*-PrOH (90:10, v/v); f=0.8 mL/min; λ=254 nm; *p*=3.2 MPa (Chiralcel OD-H)

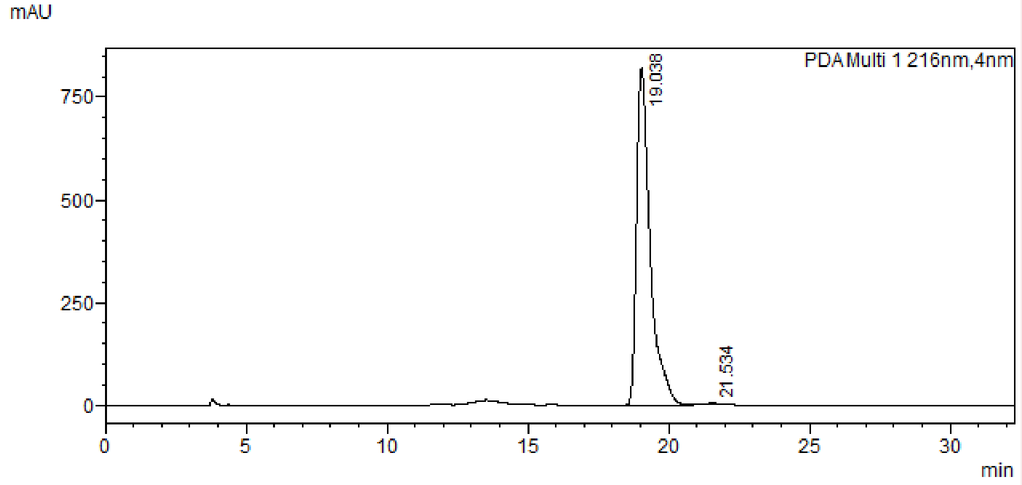

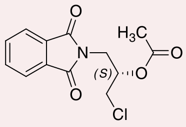

Peak Table

PDA Ch1 216nm

Peak#	Ret. Time	Area	Height	Height%	Area%
1	19.038	27574504	820463	99.694	99.788
2	21.534	58654	2518	0.306	0.212
Total		27633158	822981	100.000	100.000

HPLC analytical separation for both enantiomers of *rac*-4

HPLC conditions: *n*-hexane-*i*-PrOH (90:10, v/v); f=0.8 mL/min; λ=254 nm; *p*=3.2 MPa (Chiralcel OD-H)

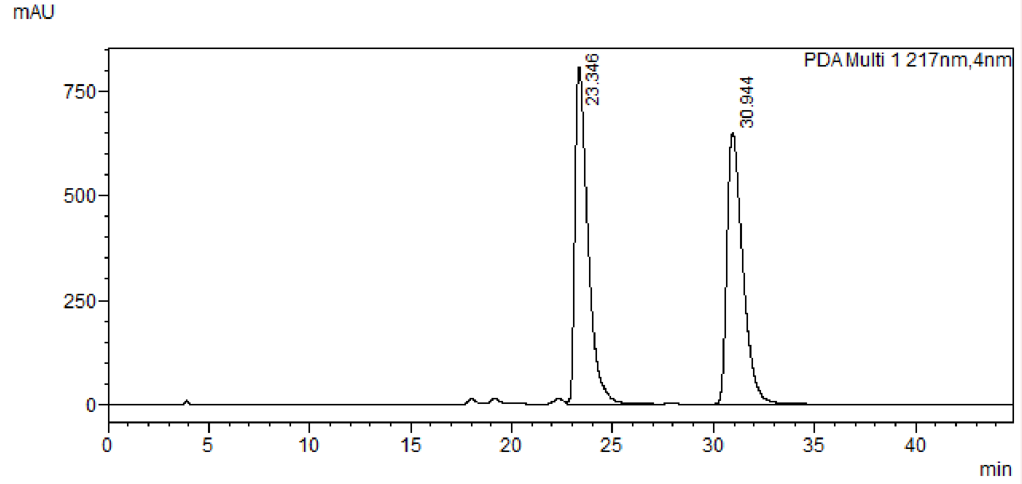

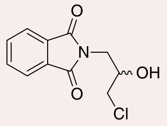

<Peak Table>

PDA Ch1 217nm

Peak#	Ret. Time	Area	Height	Conc.	Area%
1	23.346	35990049	806744	0.000	50.231
2	30.944	35659588	650080	0.000	49.769
Total		71649638	1456824		100.000

HPLC of (*S*)-(+)-4 (>99% ee) obtained from (*S*)-(+)-5a (>99% ee) (yielded after inversion of stereochemistry) after H_2SO_4-catalyzed hydrolysis of the acetate

HPLC conditions: *n*-hexane-*i*-PrOH (90:10, v/v); f=0.8 mL/min; λ=254 nm; *p*=3.2 MPa (Chiralcel OD-H)

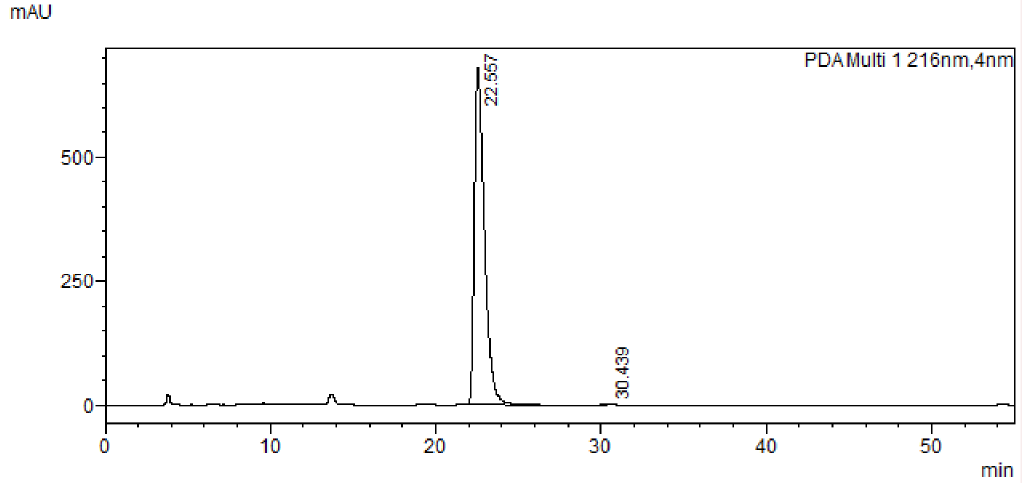

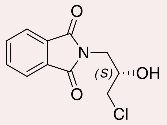

Peak Table

PDA Ch1 216nm

Peak#	Ret. Time	Area	Height	Height%	Area%
1	22.557	28963857	679137	99.797	99.823
2	30.439	51389	1384	0.203	0.177
Total		29015246	680520	100.000	100.000

2-(Oxiran-2-ylmethyl)-1H-isoindole-1,3(2H)-dione (rac-3)

^{1}H NMR spectrum of *rac*-**3** (500 MHz, $CDCl_3$)

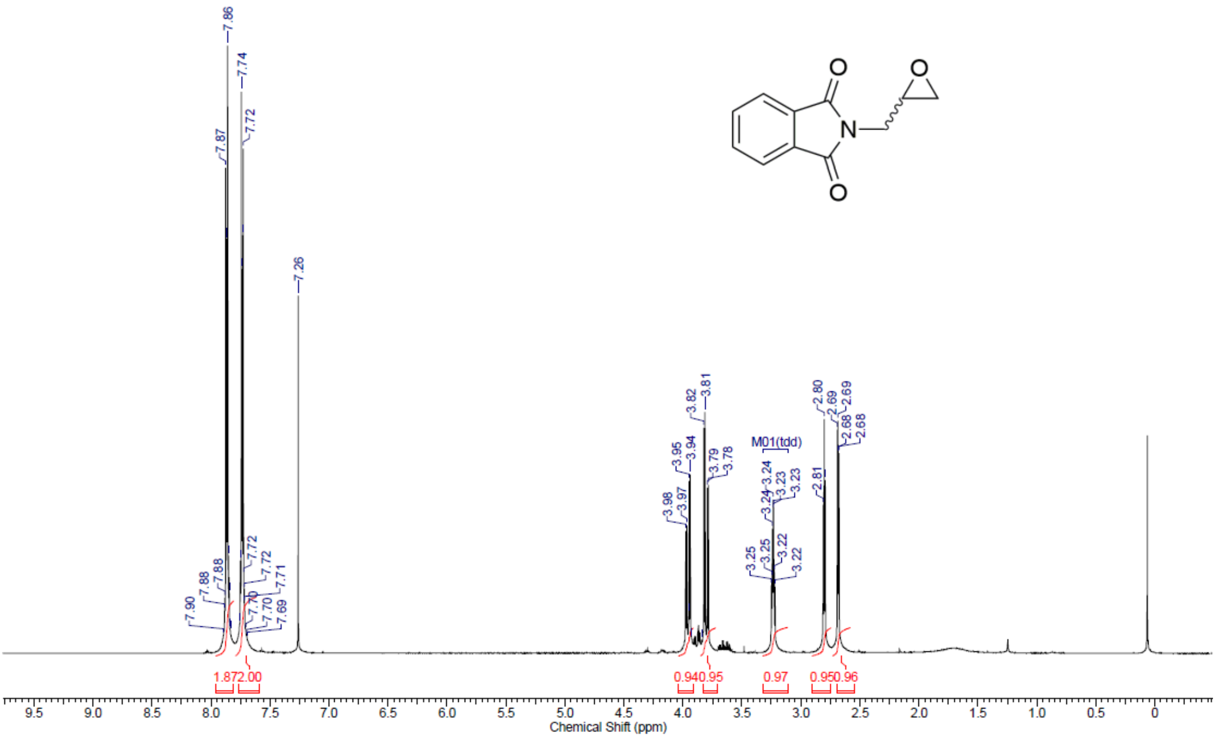

^{13}C NMR spectrum of *rac*-**3** (126 MHz, $CDCl_3$)

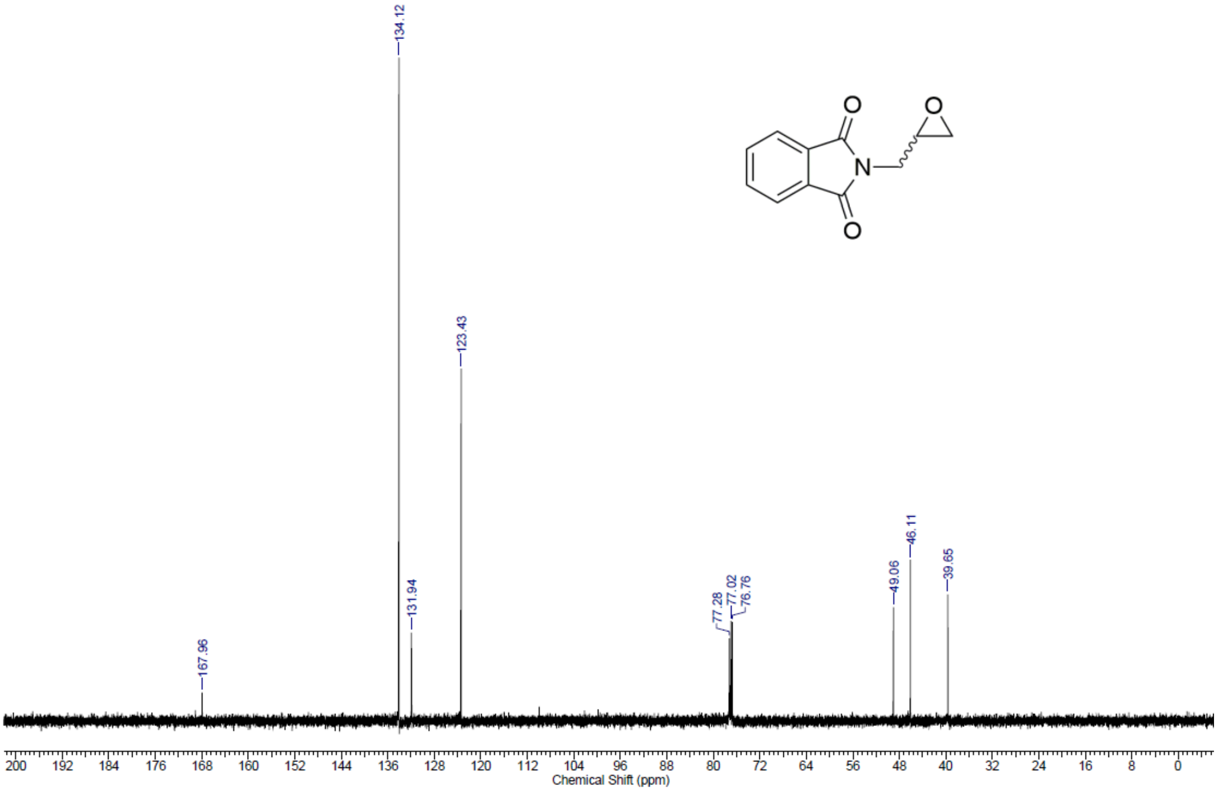

FTMS spectrum of *rac*-**3** (ESI-TOF)

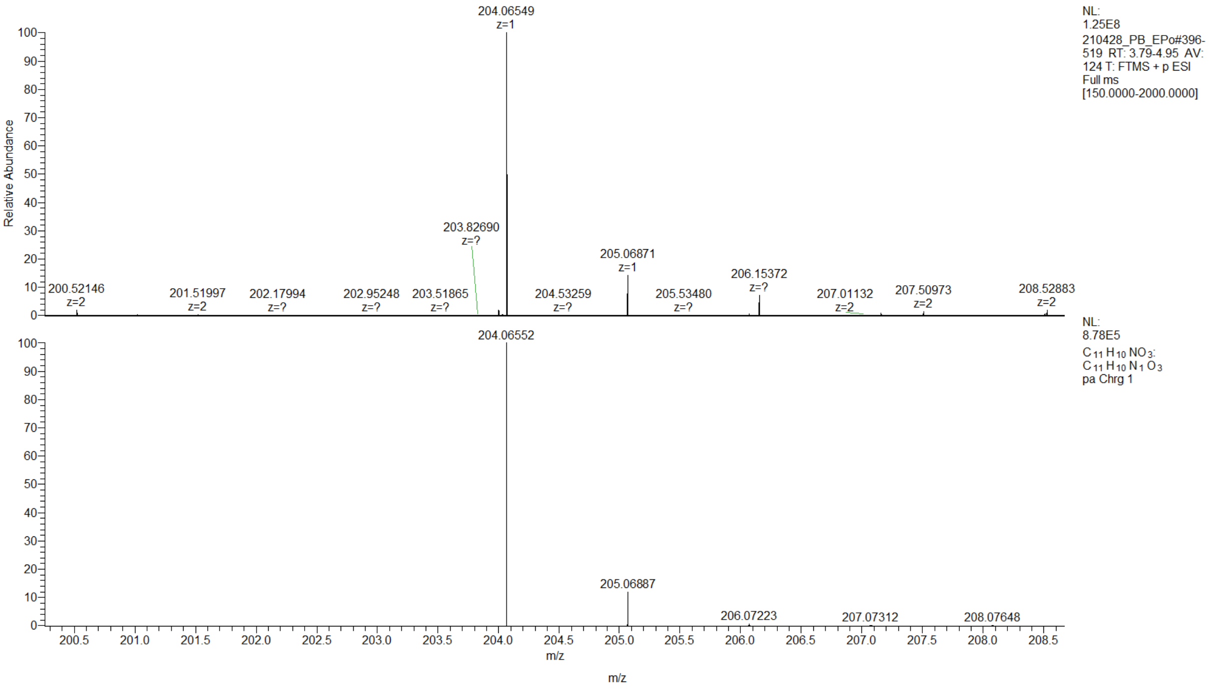

IR spectrum of *rac*-**3** (Mineral oil, Nujol)

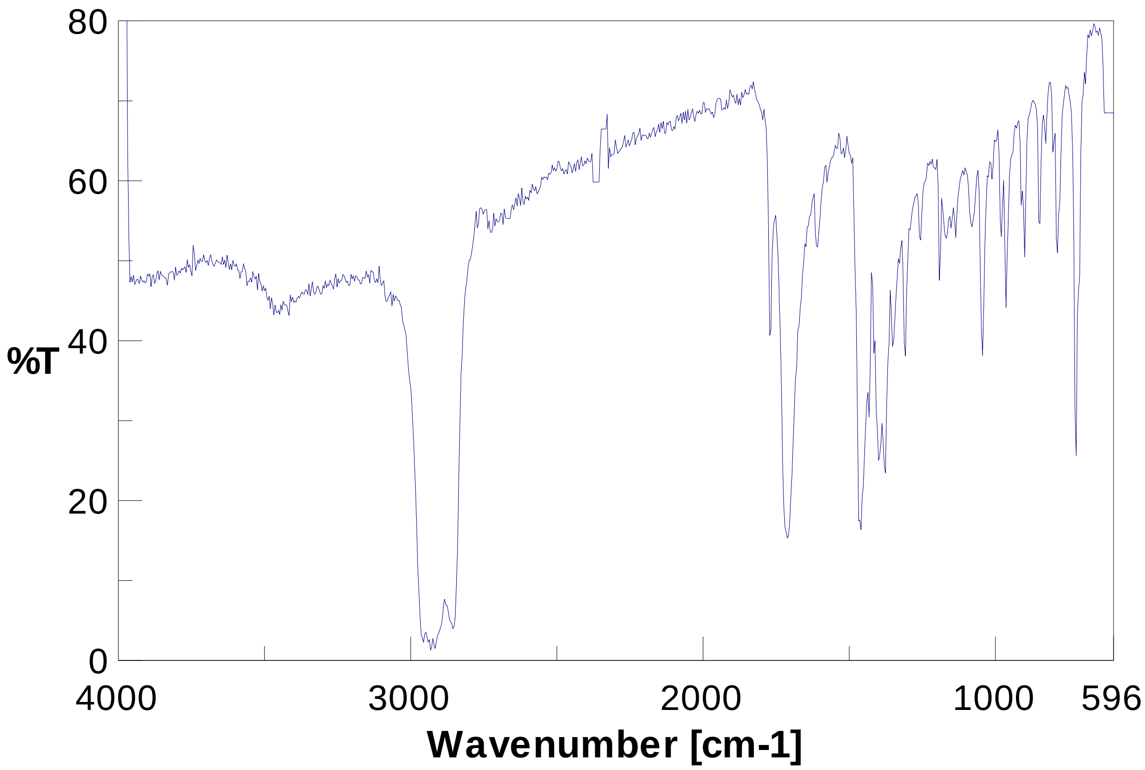

2-(3-Chloro-2-hydroxypropyl)-1H-isoindole-1,3(2H)-dione (rac-4)

^{1}H NMR spectrum of *rac*-**4** (500 MHz, $CDCl_3$)

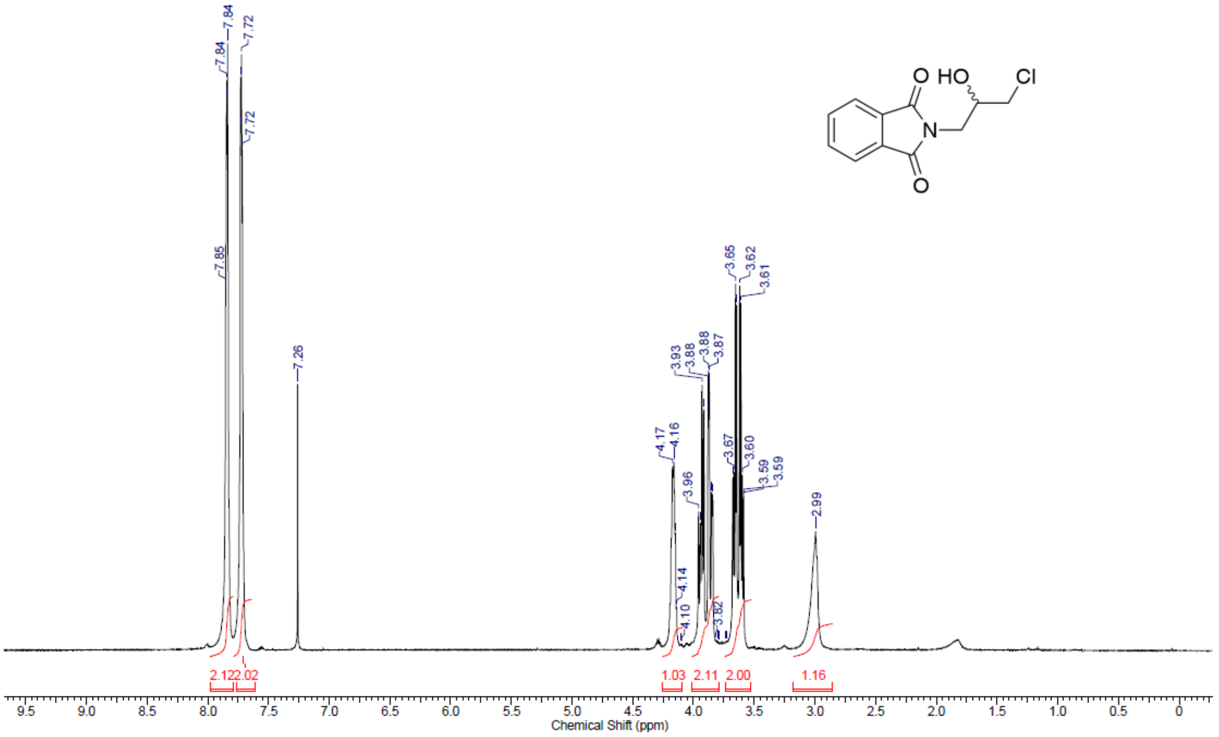

^{13}C NMR spectrum of *rac*-**4** (126 MHz, $CDCl_3$)

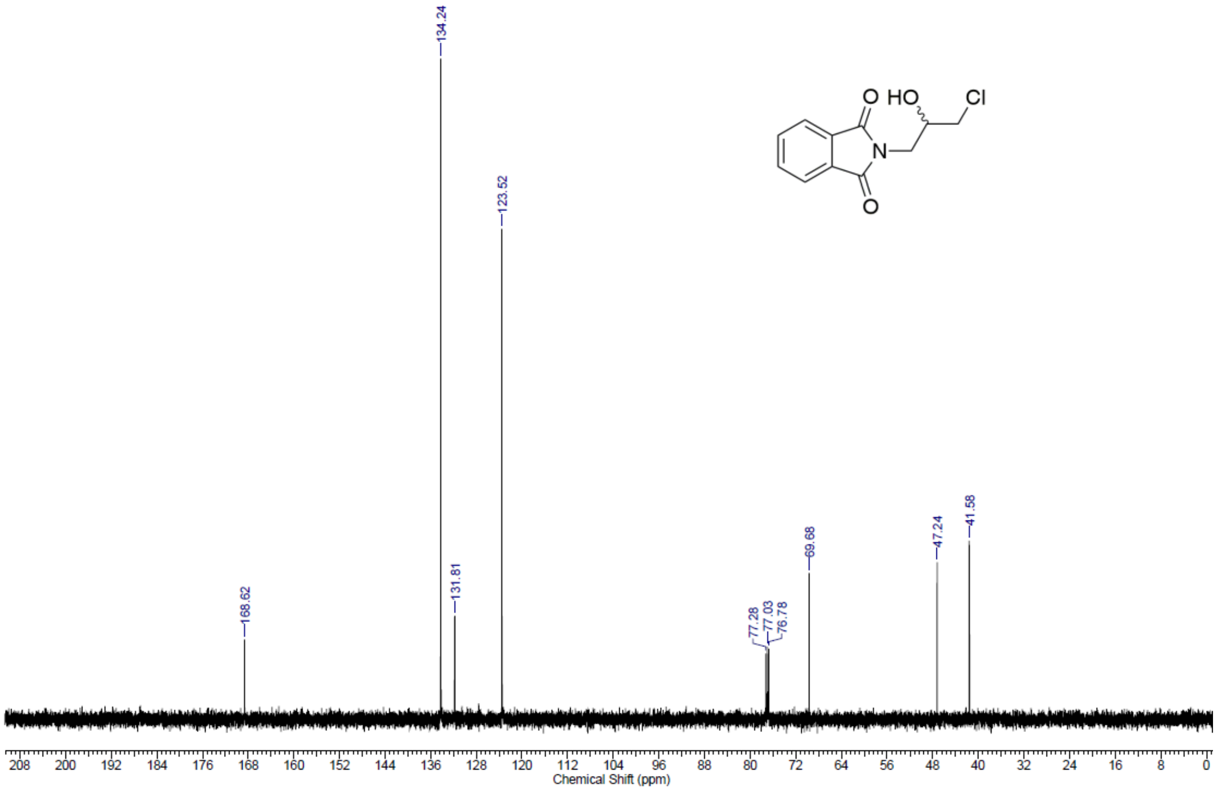

FTMS spectrum of *rac*-**4** (ESI-TOF)

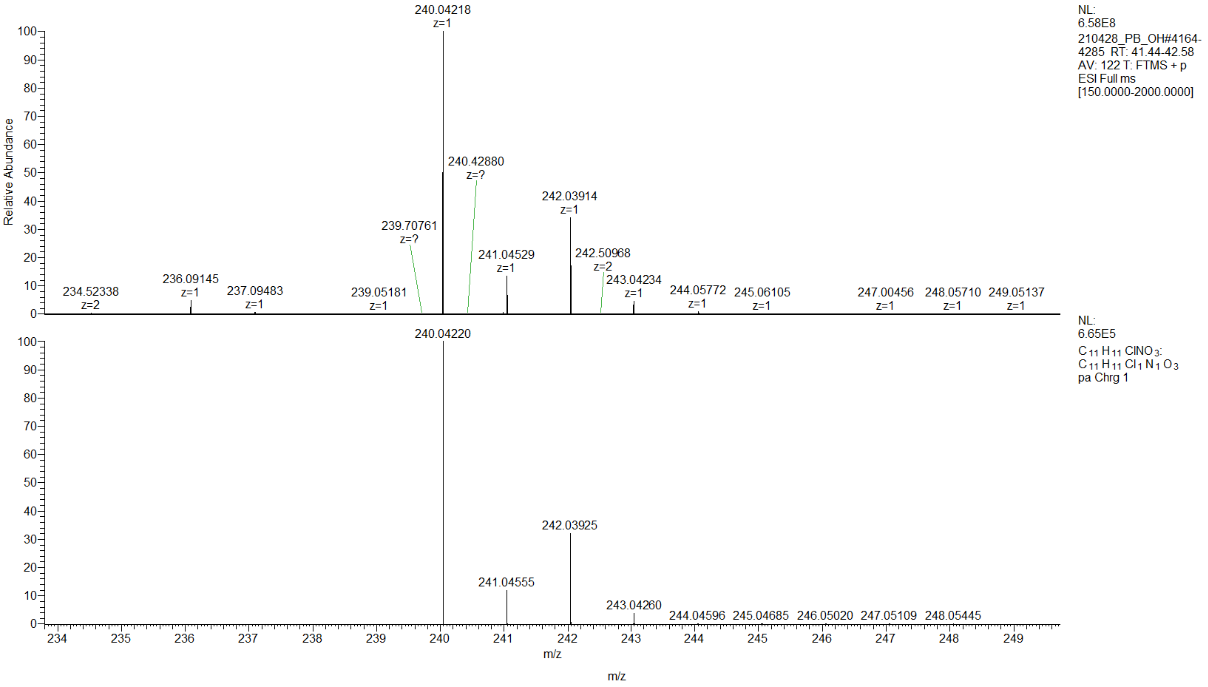

IR spectrum of *rac*-**4** (Mineral oil, Nujol)

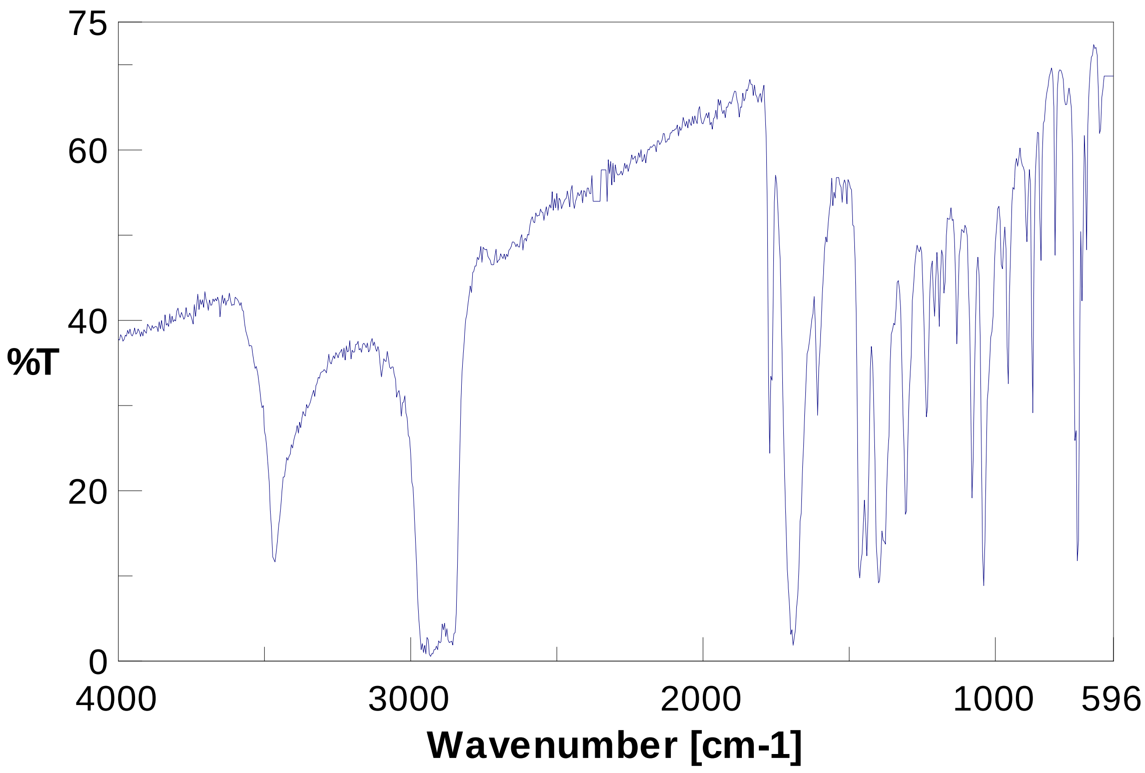

1-Chloro-3-(1,3-dioxo-1,3-dihydro-2H-isoindol-2-yl)propan-2-yl acetate (rac-5a)

^{1}H NMR spectrum of *rac*-**5a** (500 MHz, $CDCl_3$)

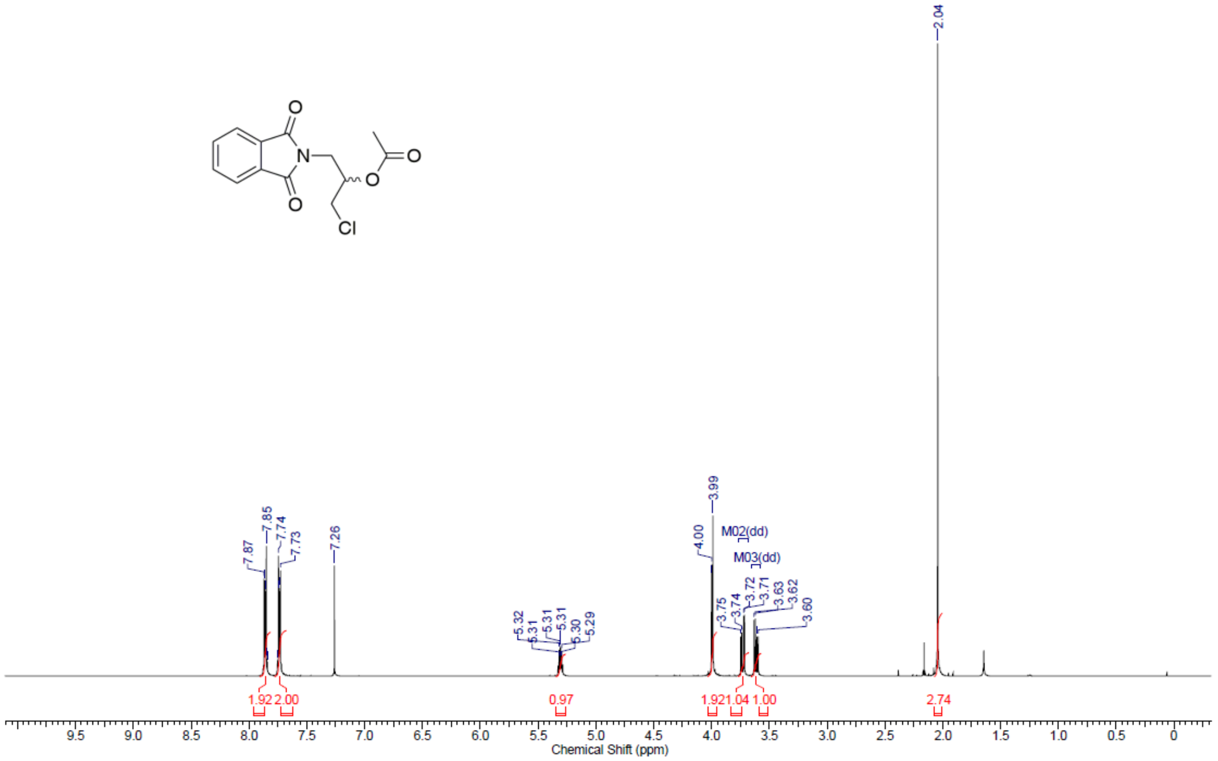

^{13}C NMR spectrum of *rac*-**5a** (126 MHz, $CDCl_3$)

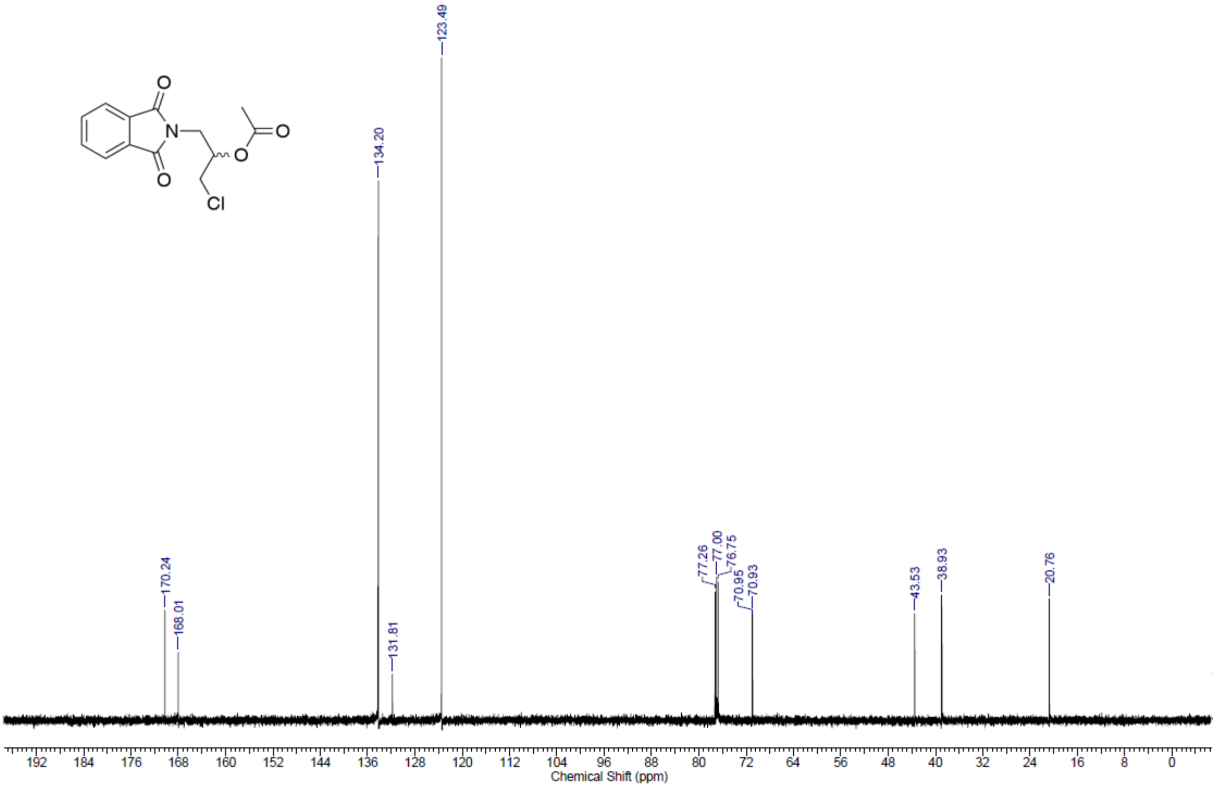

FTMS spectrum of *rac*-**5a** (ESI-TOF)

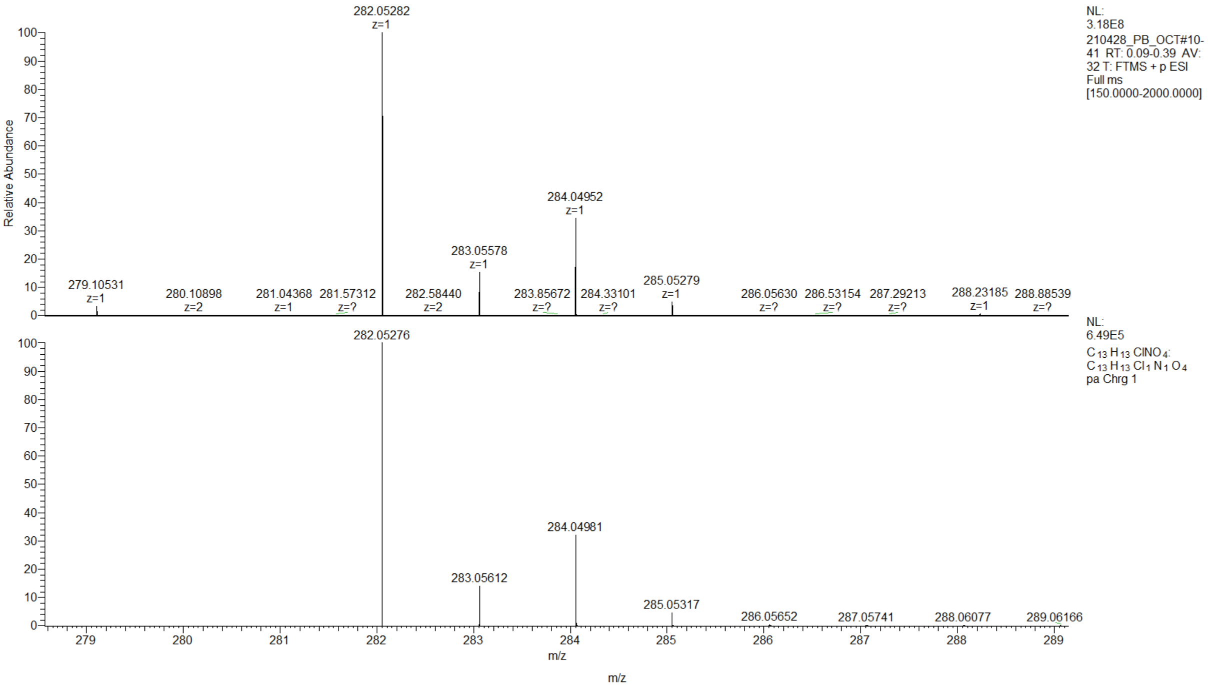

IR spectrum of *rac*-**5a** (Mineral oil, Nujol)

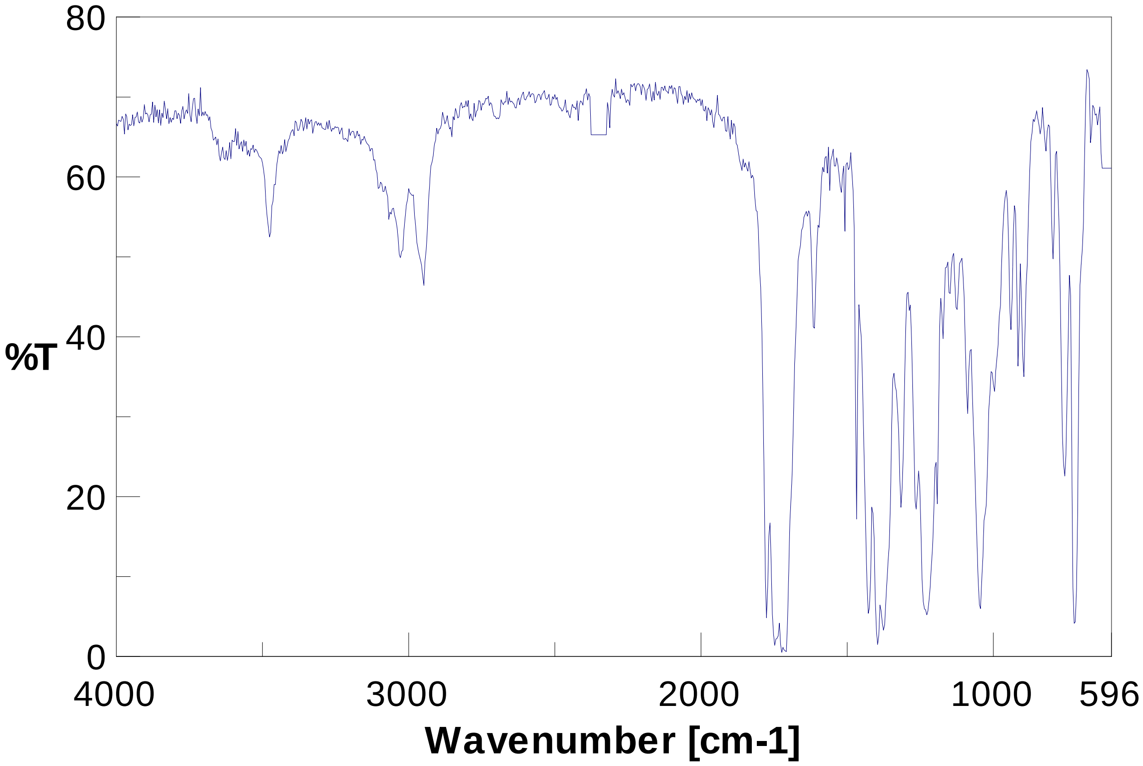

1-Chloro-3-(1,3-dioxo-1,3-dihydro-2H-isoindol-2-yl)propan-2-yl butanoate (rac-5b)

^{1}H NMR spectrum of *rac*-**5b** (500 MHz, $CDCl_3$)

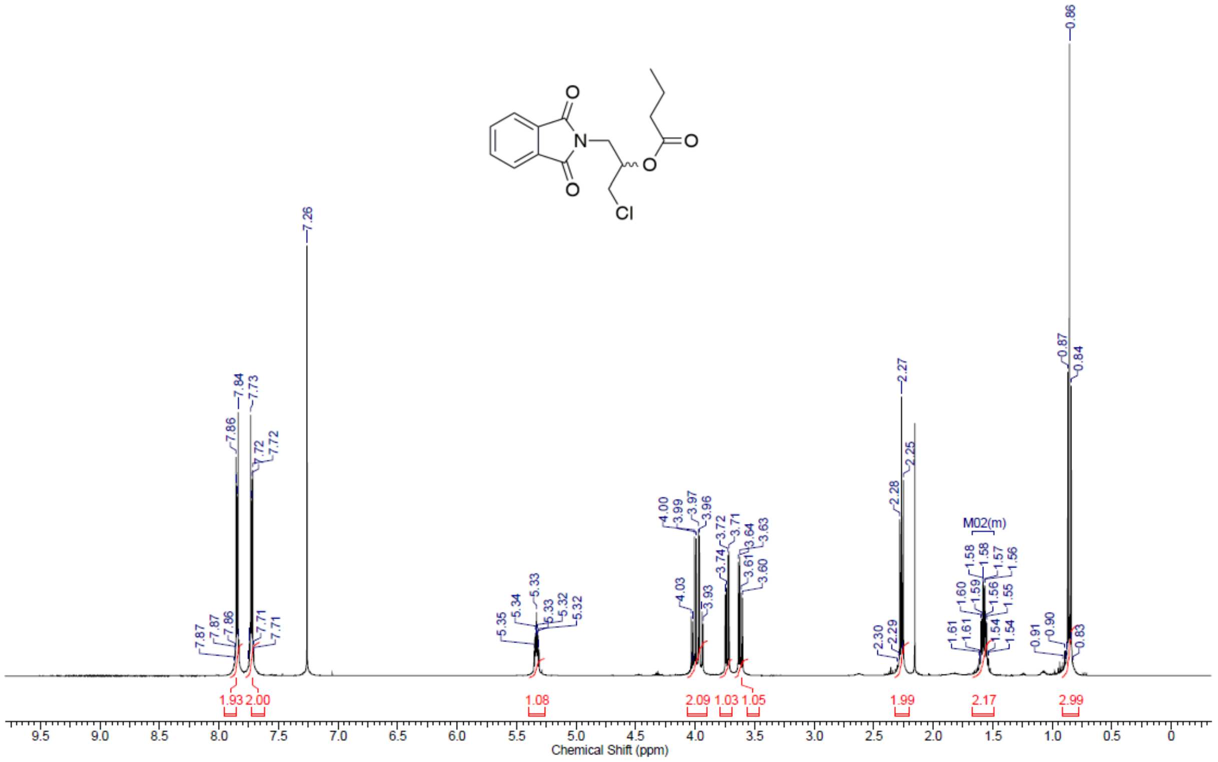

^{13}C NMR spectrum of *rac*-**5b** (126 MHz, $CDCl_3$)

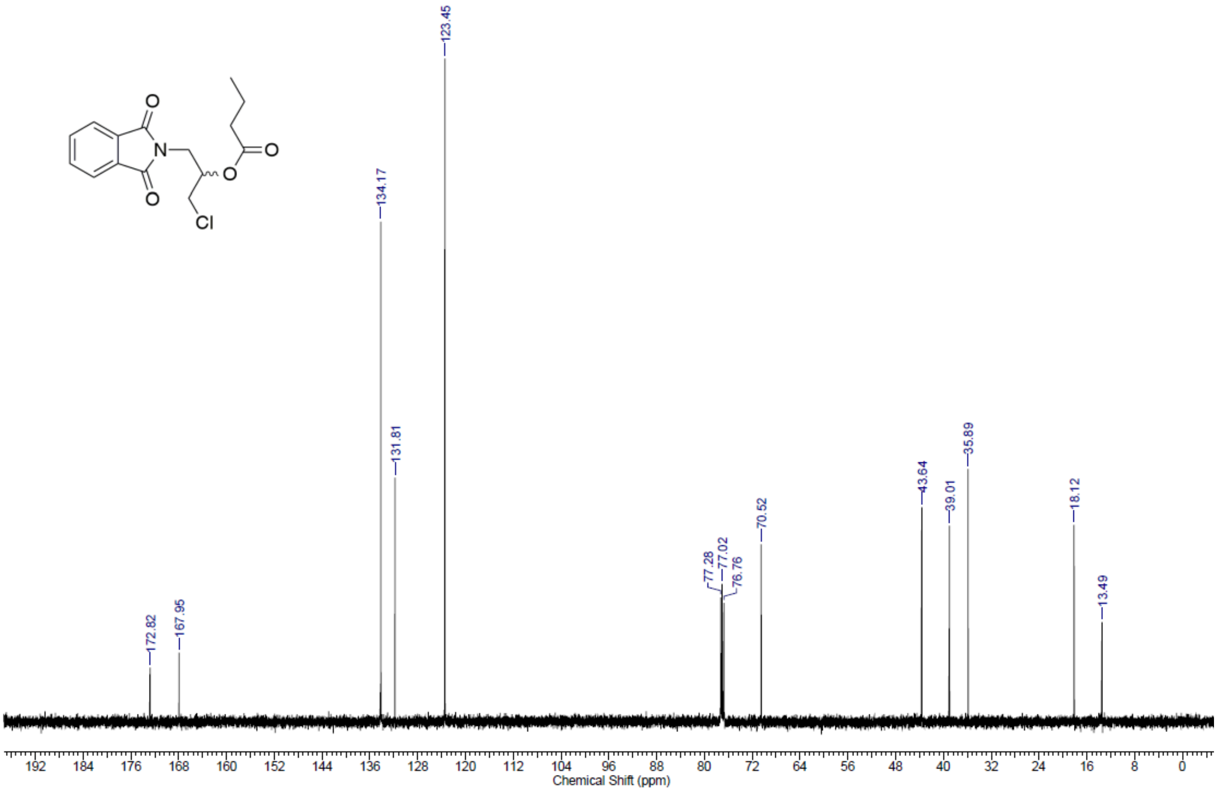

HRMS spectrum of *rac*-**5b** (ESI-TOF)

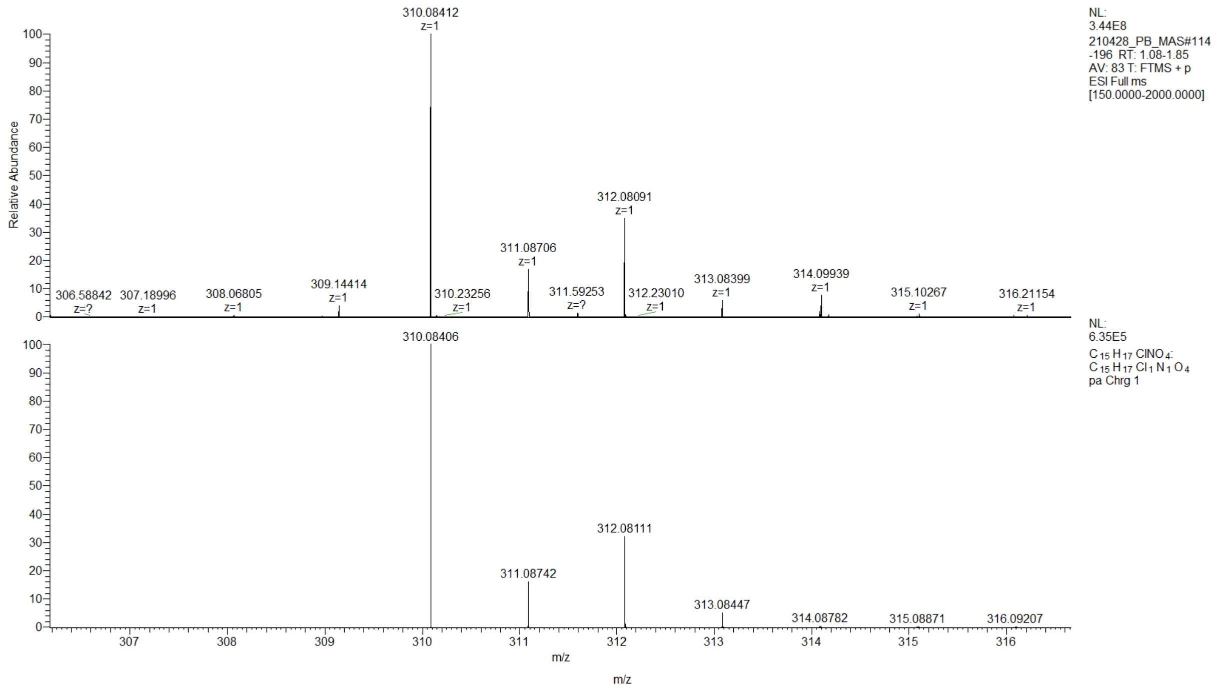

IR spectrum of *rac*-**5b** (Mineral oil, Nujol)

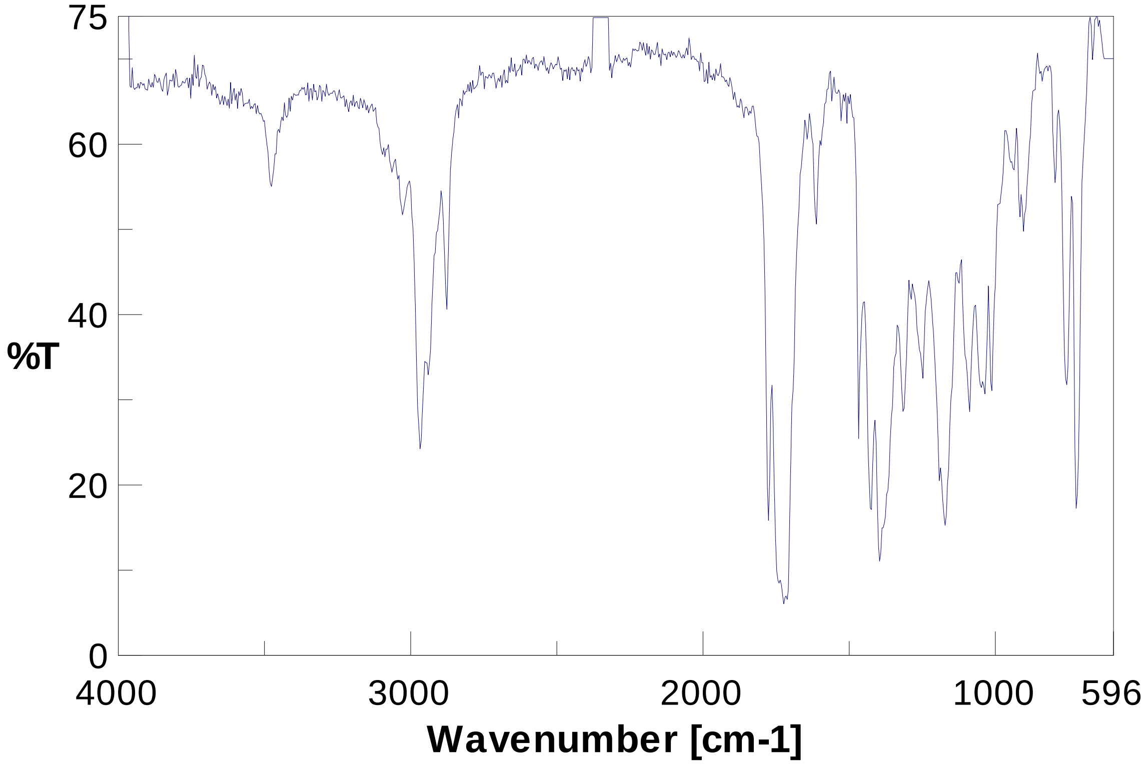

2-[2-Hydroxy-3-(naphthalen-1-yloxy)propyl]-1H-isoindole-1,3(2H)-dione (rac-6a)

^{1}H NMR spectrum of *rac*-**6a** (500 MHz, $CDCl_3$)

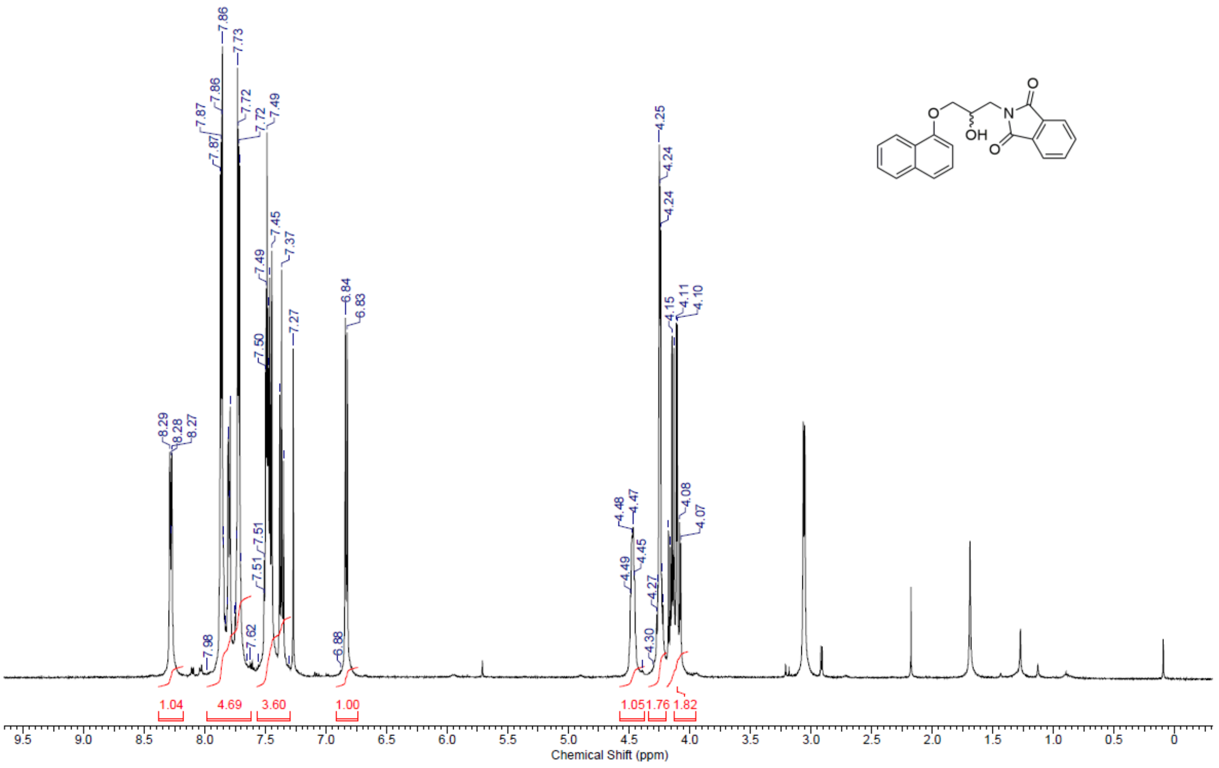

^{13}C NMR spectrum of *rac*-**6a** (126 MHz, $CDCl_3$)

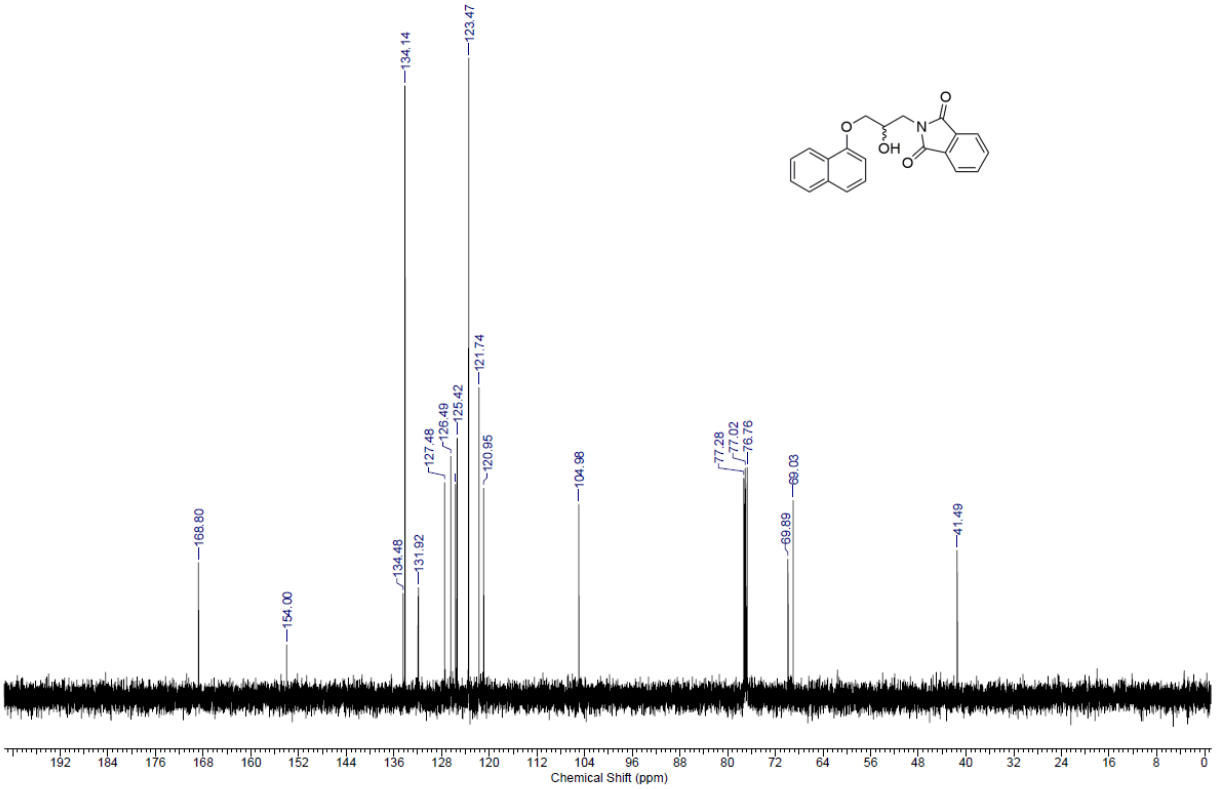

HRMS spectrum of *rac*-**6a** (ESI-TOF)

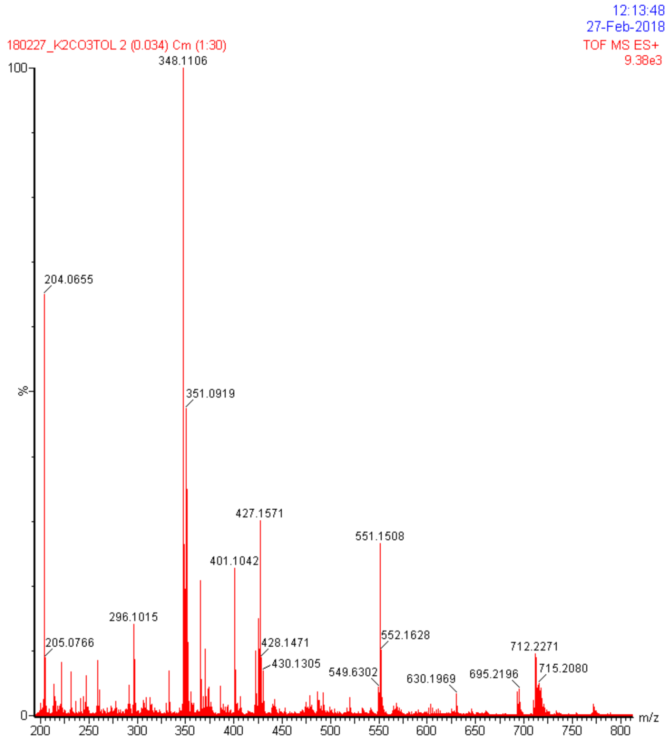

IR spectrum of *rac*-**6a** (Mineral oil, Nujol)

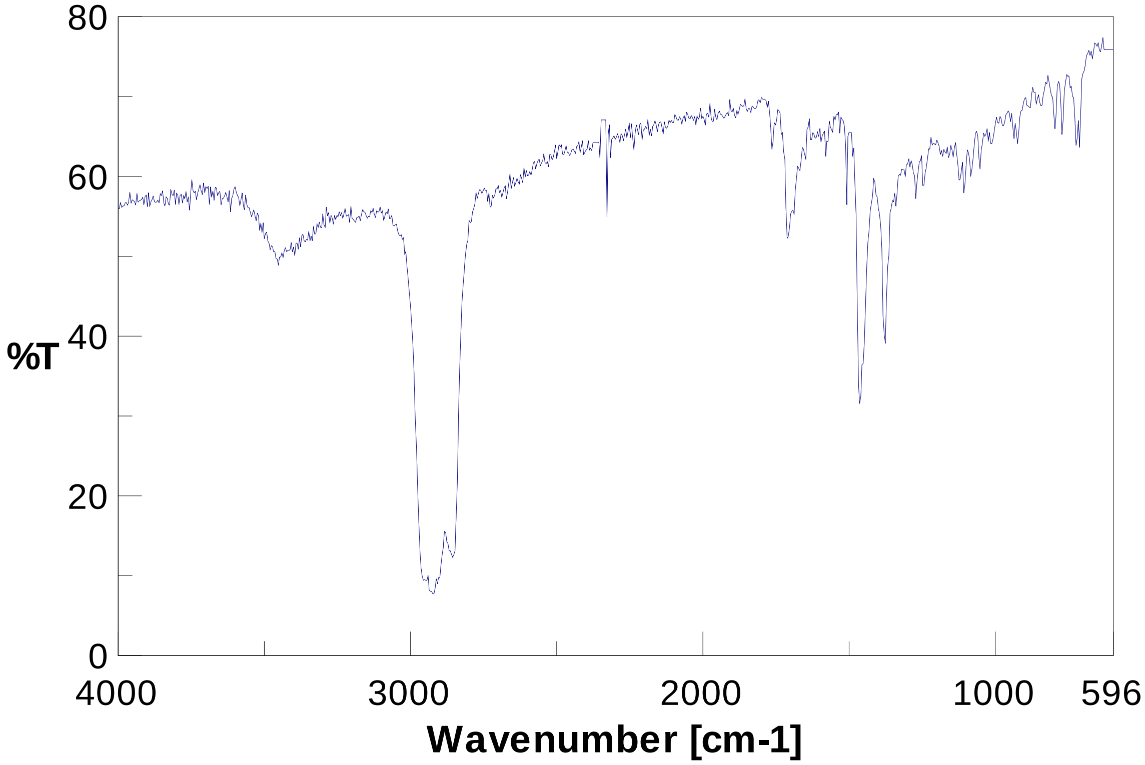

2-{(2-Hydroxy-3-[2-(prop-2-en-1-yl)phenoxy]propyl}-1H-isoindole-1,3(2H)-dione (rac-6b)

^{1}H NMR spectrum of *rac*-**6b** (500 MHz, $CDCl_3$)

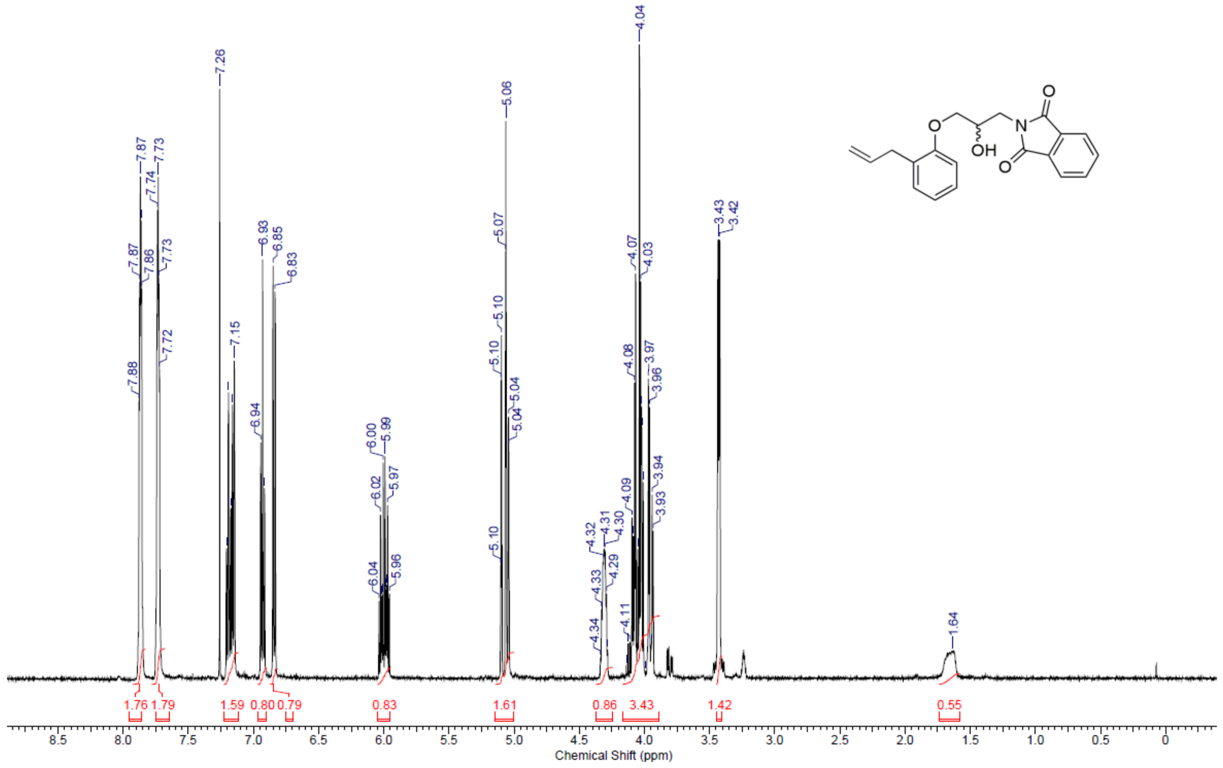

^{13}C NMR spectrum of *rac*-**6b** (126 MHz, $CDCl_3$)

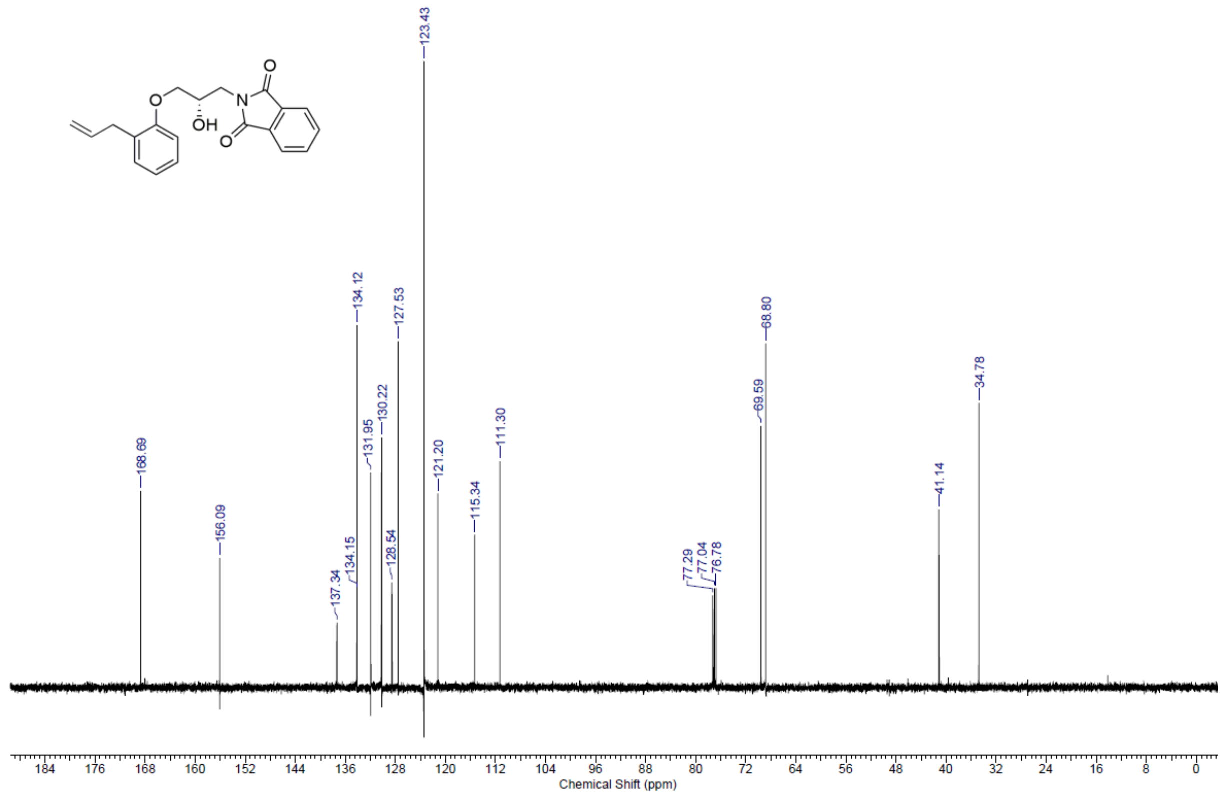

HRMS spectrum of *rac*-**6b** (ESI-TOF)

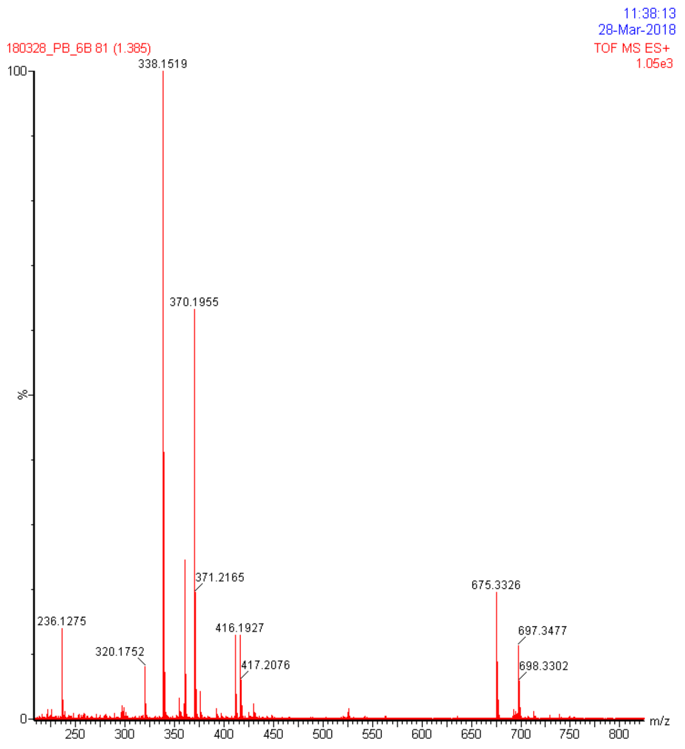

IR spectrum of *rac*-**6b** (Mineral oil, Nujol)

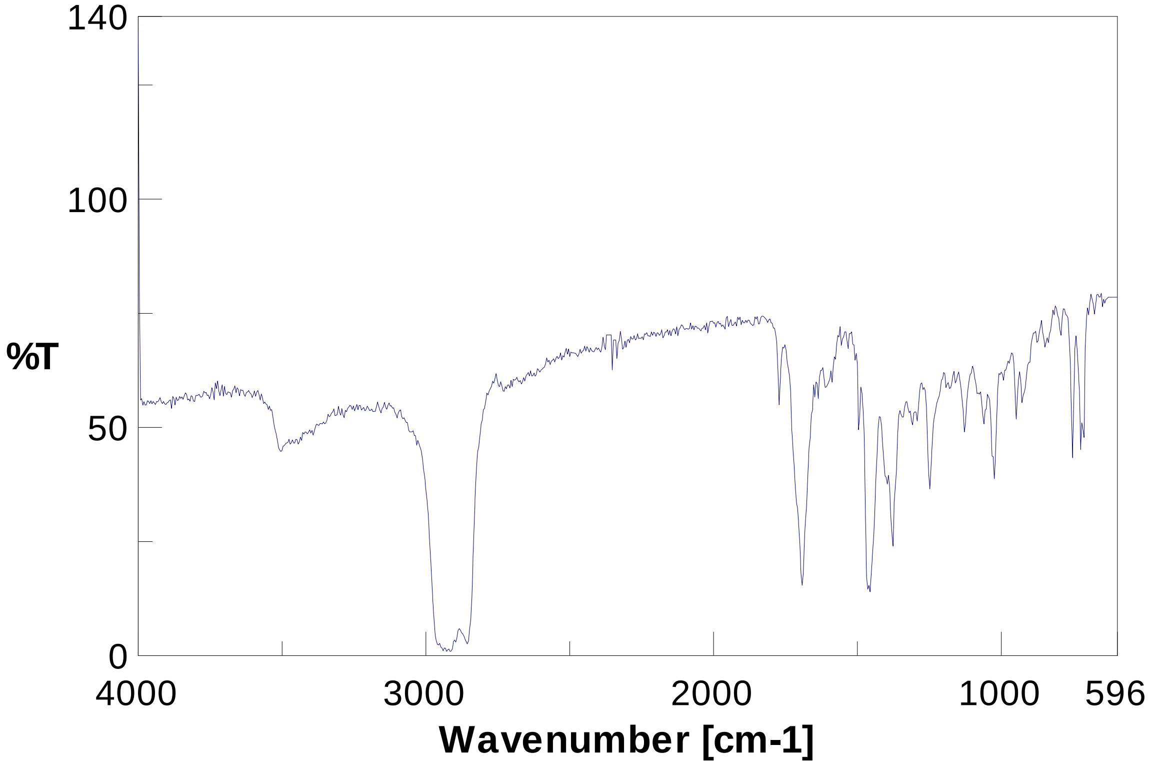

2-(2-Hydroxy-3-(naphthalen-1-yloxy)propyl)-1H-isoindole-1,3(2H)-dione (rac-7a)

^{1}H NMR spectrum of *rac*-**7a** (500 MHz, DMSO-d_6)

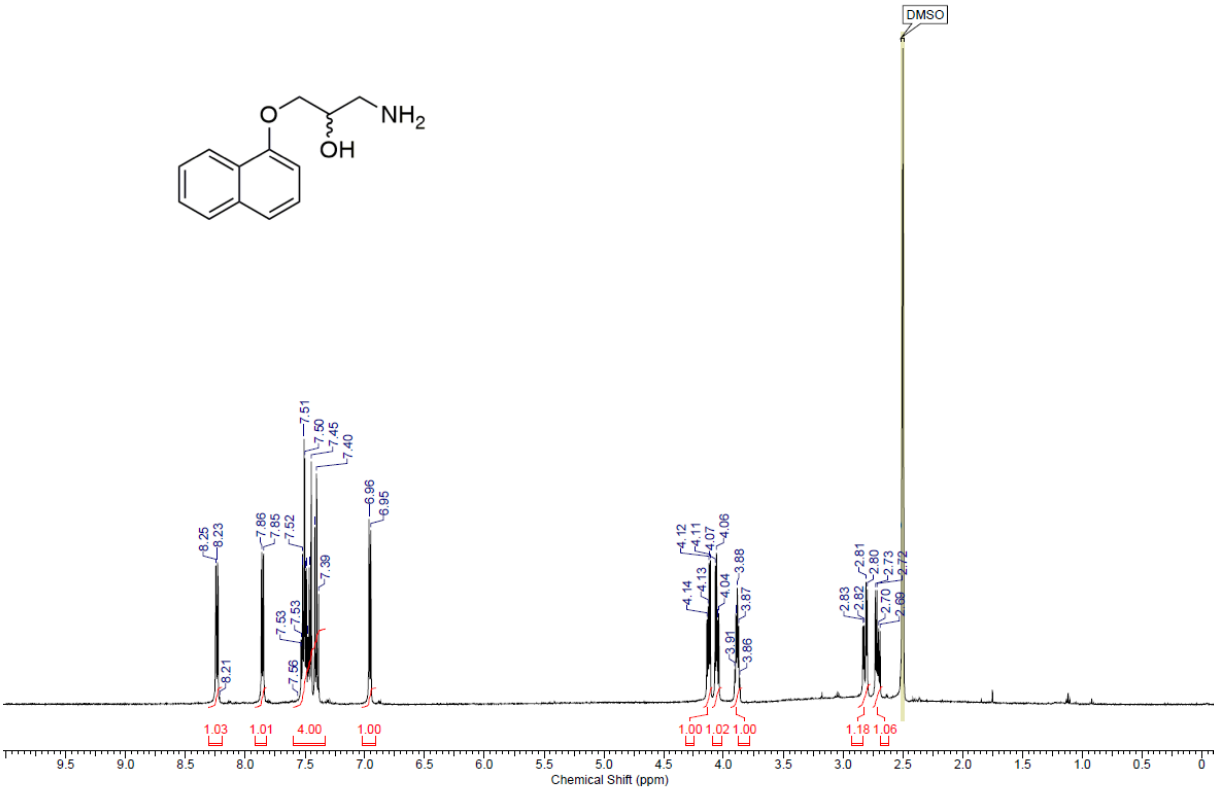

^{13}C NMR spectrum of *rac*-**7a** (126 MHz, DMSO-d_6)

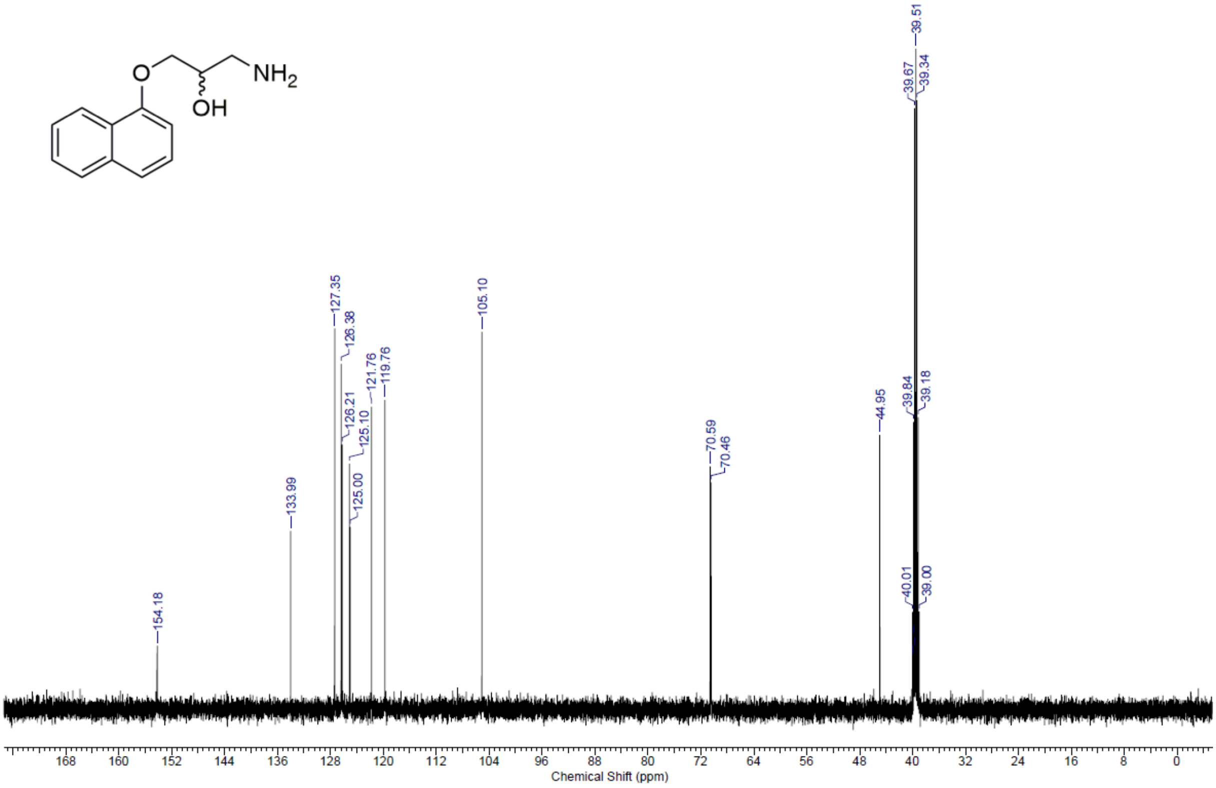

HRMS spectrum of *rac*-**7a** (ESI-TOF)

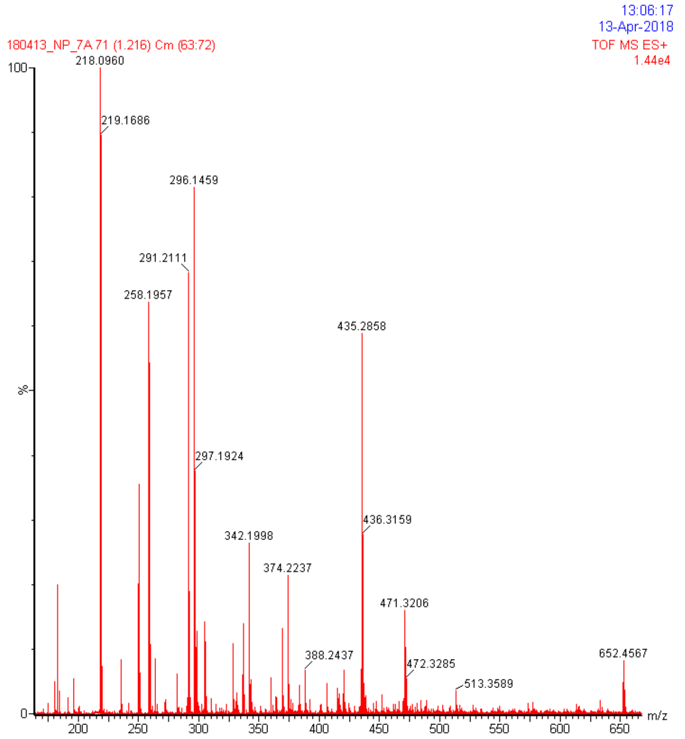

FTMS spectrum of *rac*-**7a** (ESI-TOF)

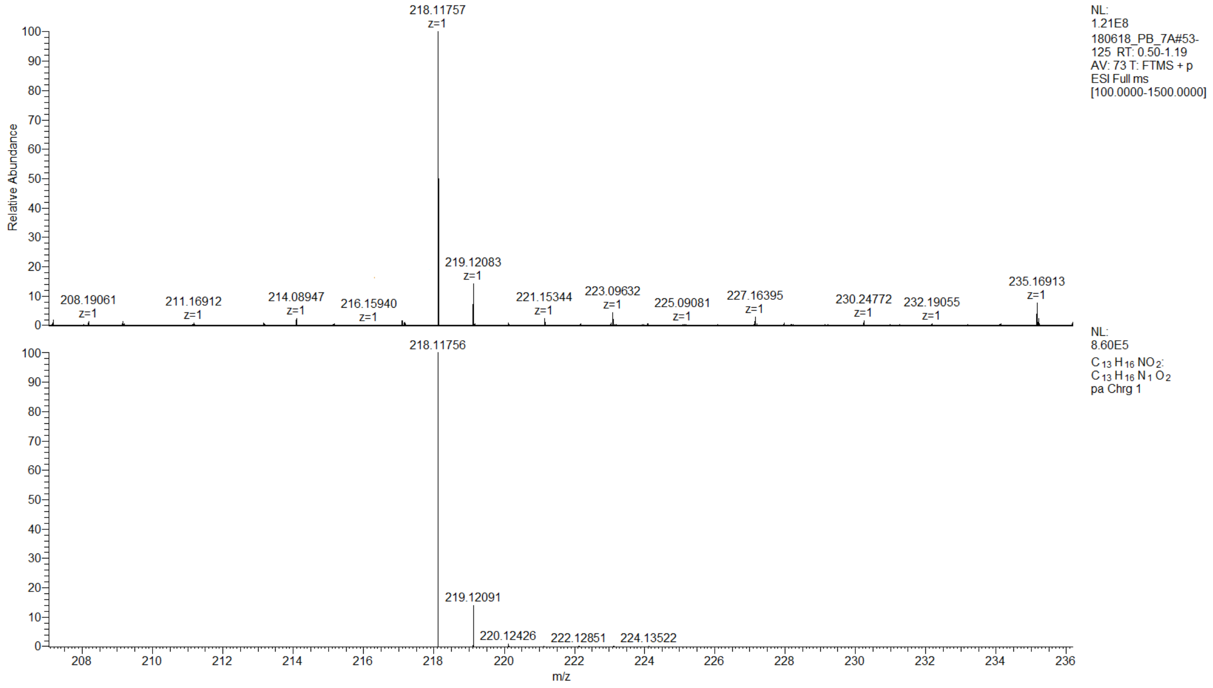

IR spectrum of *rac*-**7a** (Mineral oil, Nujol)

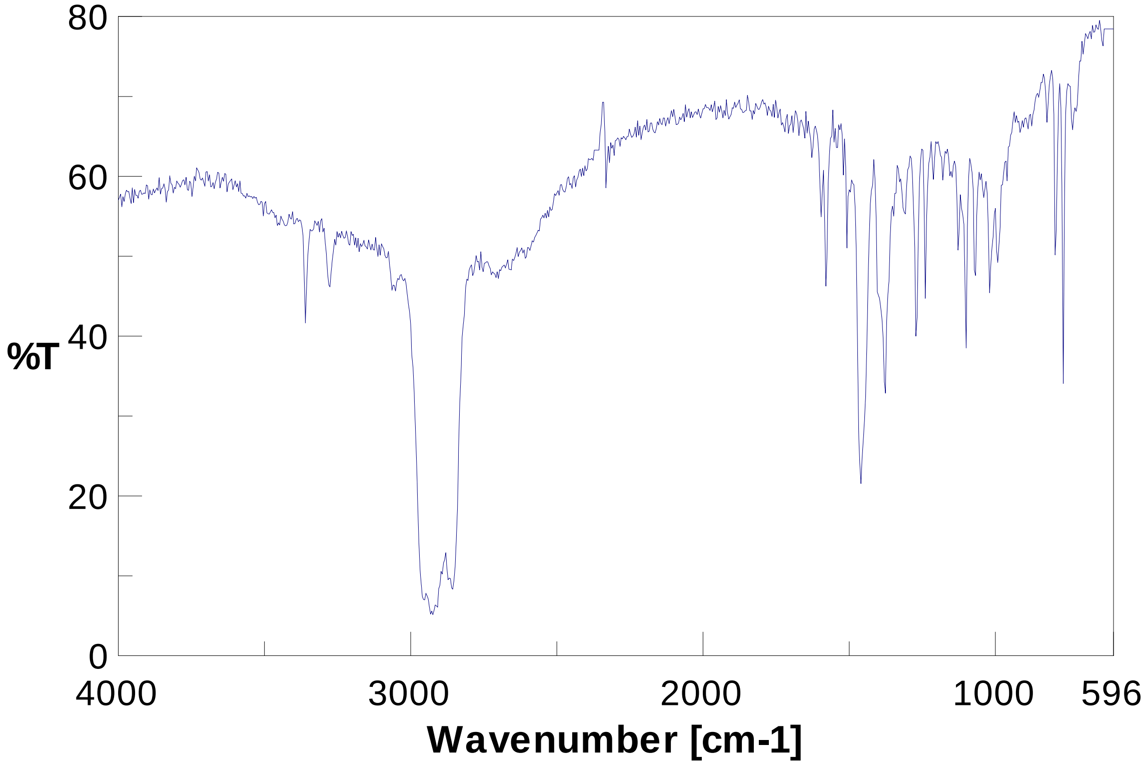

2-[2-Hydroxy-3-(2-(prop-2-en-1-yl)phenoxy)propyl]-1H-isoindole-1,3(2H)-dione (rac-7b)

^{1}H NMR spectrum of *rac*-**7b** (500 MHz, DMSO-d_6)

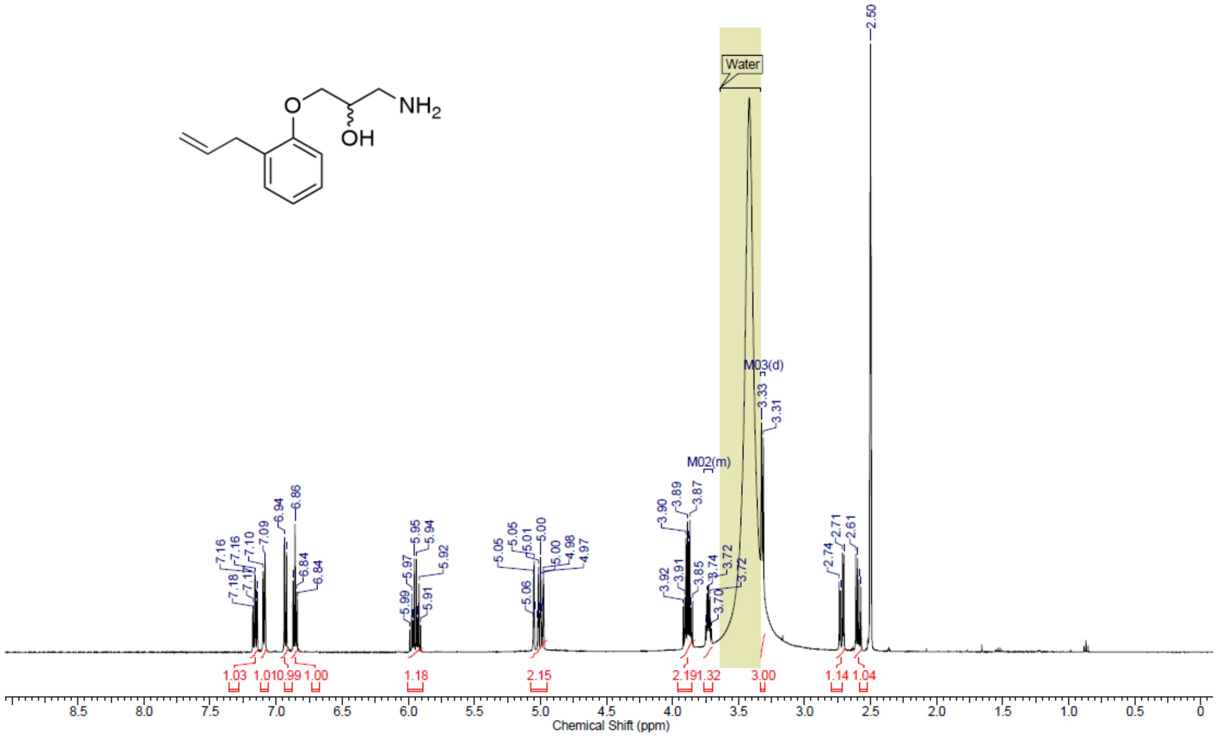

^{13}C NMR spectrum of *rac*-**7b** (126 MHz, DMSO-d_6)

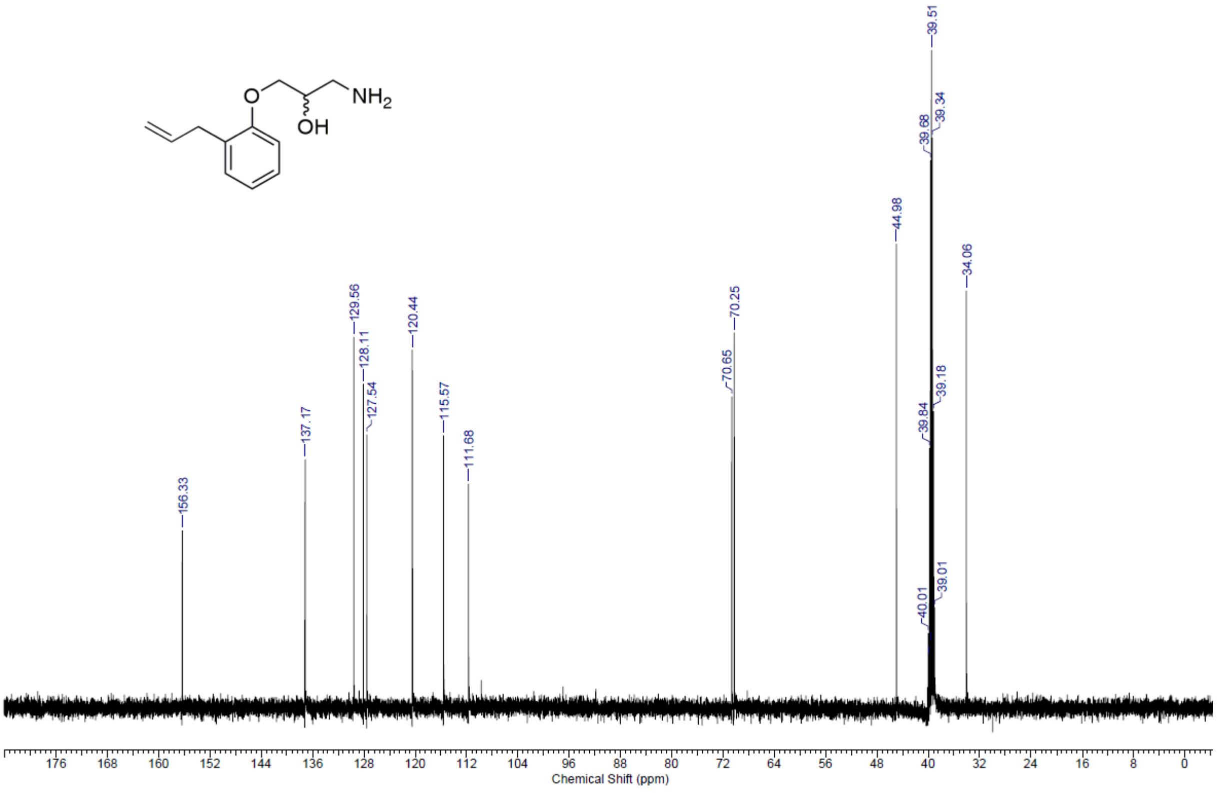

HRMS spectrum of *rac*-**7b** (ESI-TOF)

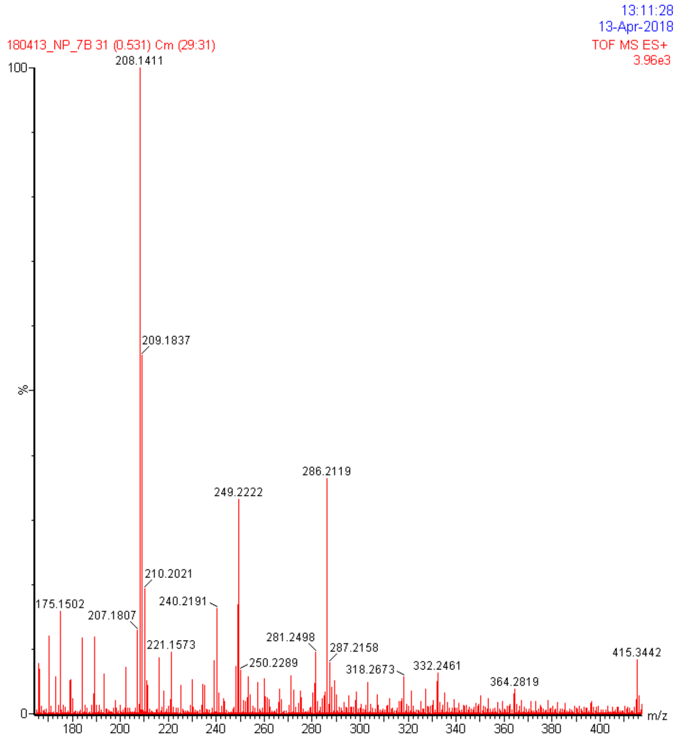

FTMS spectrum of *rac*-**7b** (ESI-TOF)

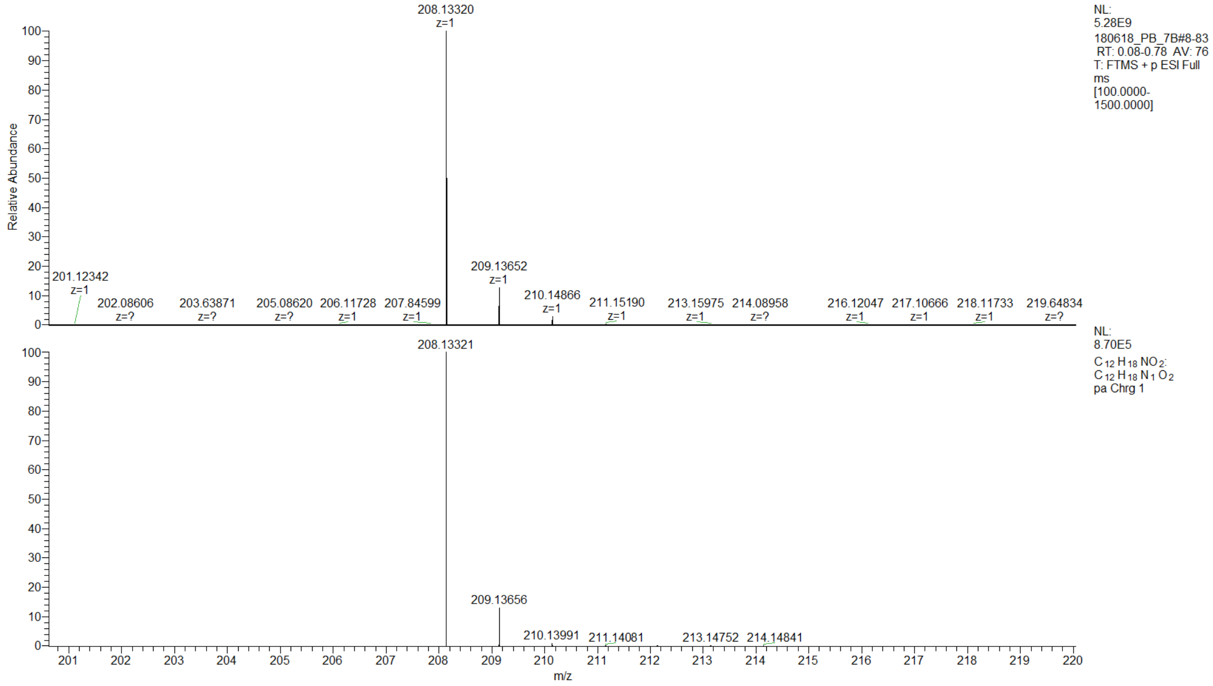

IR spectrum of *rac*-**7b** (Mineral oil, Nujol)

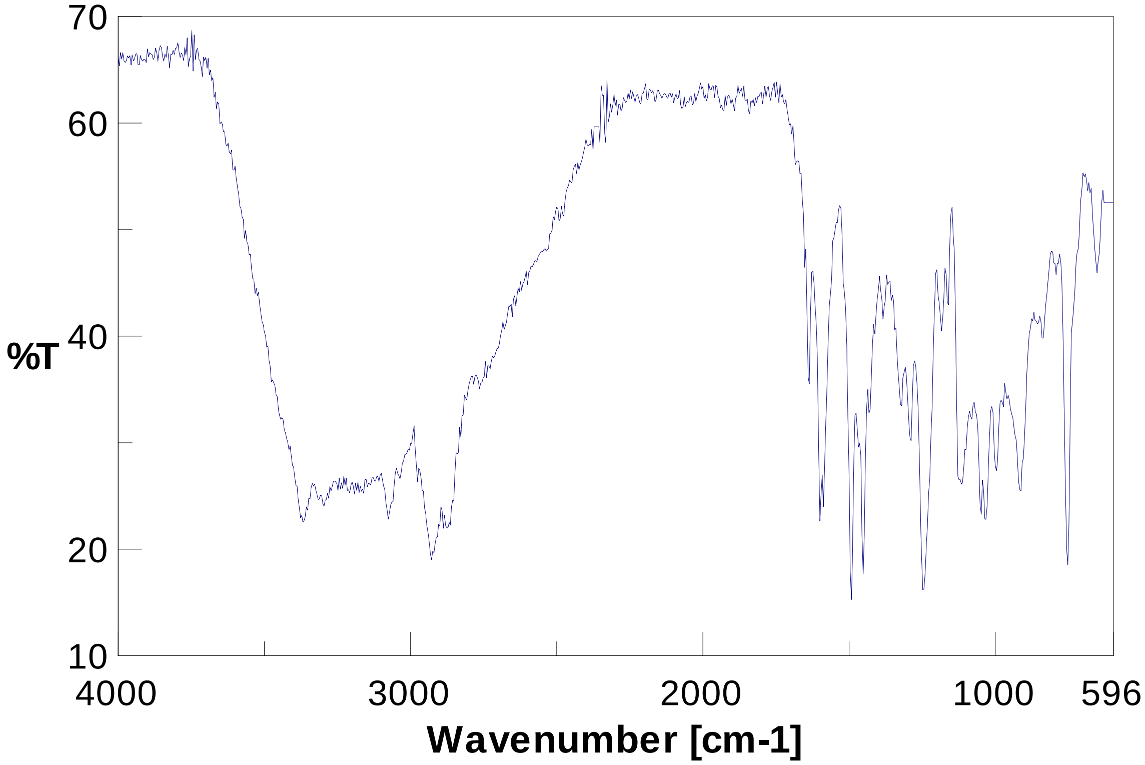

2-[2-Hydroxy-3-(1H-indol-4-yloxy)propyl]-1H-isoindole-1,3(2H)-dione (rac-7c)

^{1}H NMR spectrum of *rac*-**7c** (500 MHz, CD_3OD)

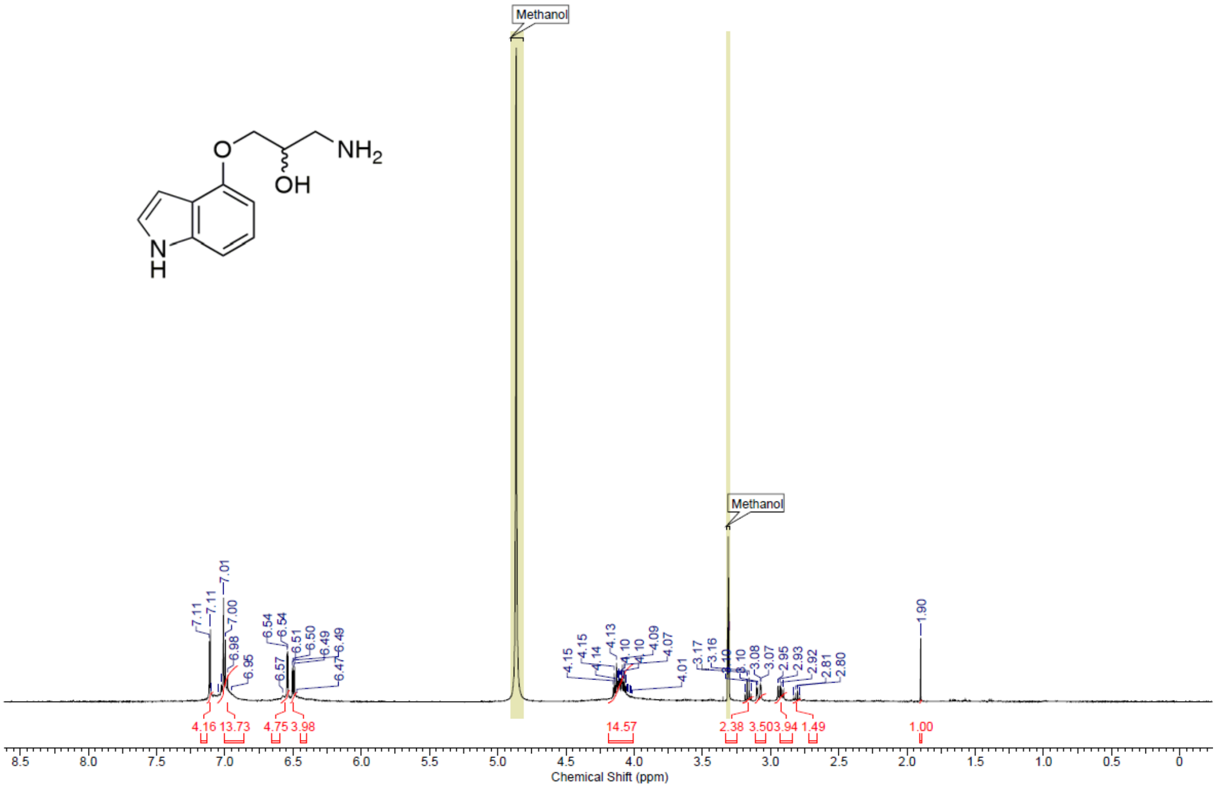

^{13}C NMR spectrum of *rac*-**7c** (126 MHz, CD_3OD)

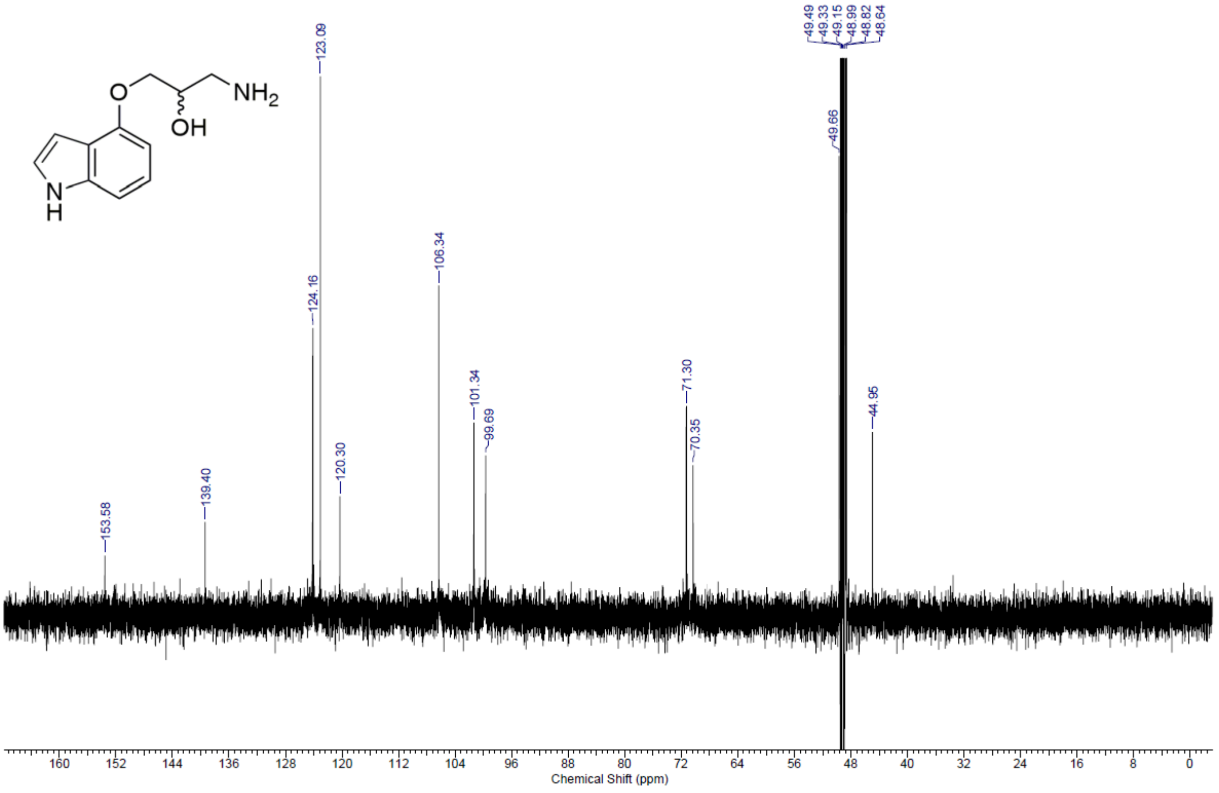

HRMS spectrum of *rac*-**7c** (ESI-TOF)

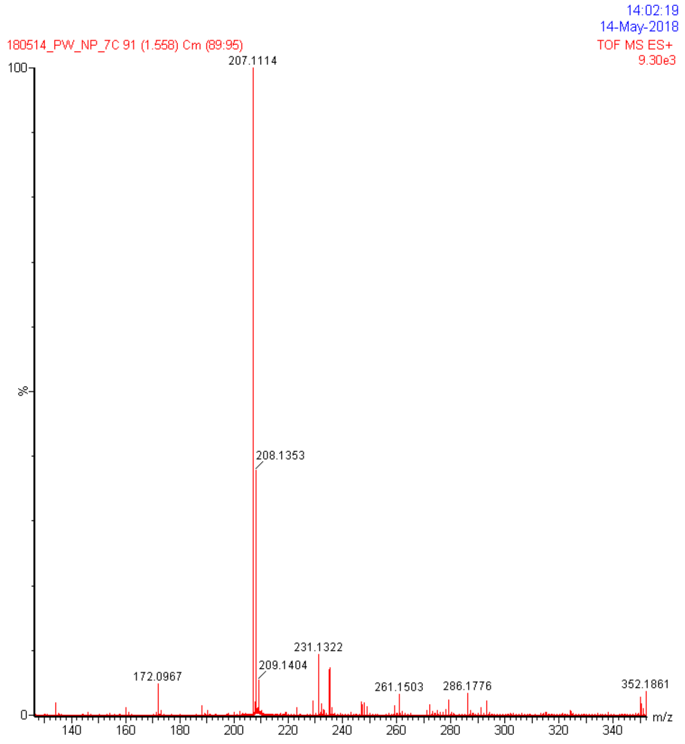

FTMS spectrum of *rac*-**7c** (ESI-TOF)

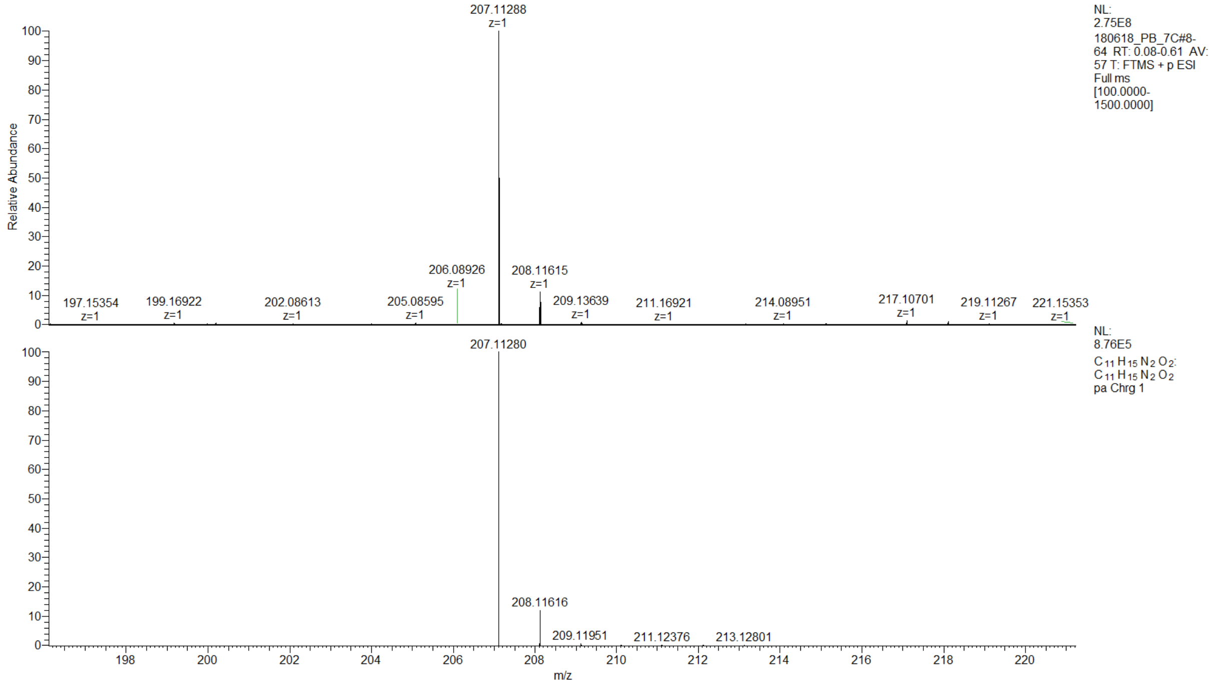

IR spectrum of *rac*-**7c** (Mineral oil, Nujol)

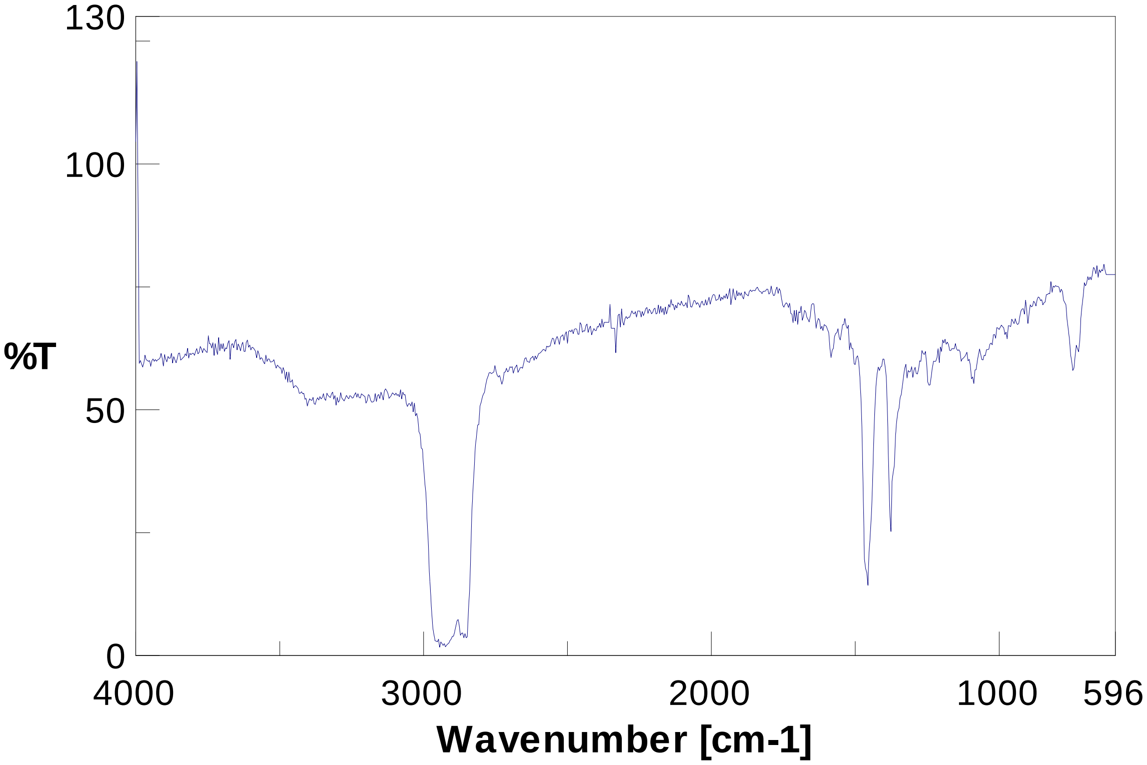

2-[3-(9H-Carbazol-4-yloxy)-2-hydroxypropyl]-1H-isoindole-1,3(2H)-dione (rac-7d)

^{1}H NMR spectrum of *rac*-**7d** (500 MHz, CD_3OD)

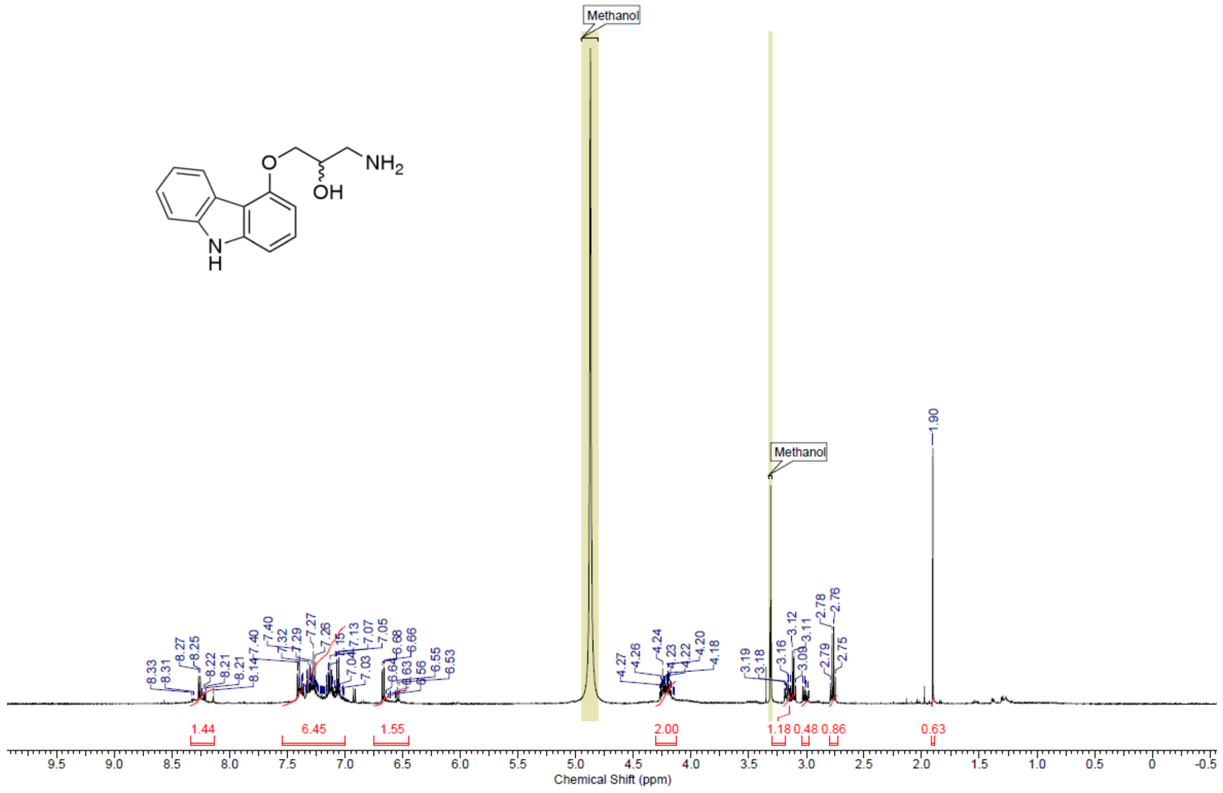

^{13}C NMR spectrum of *rac*-**7d** (126 MHz, CD_3OD)

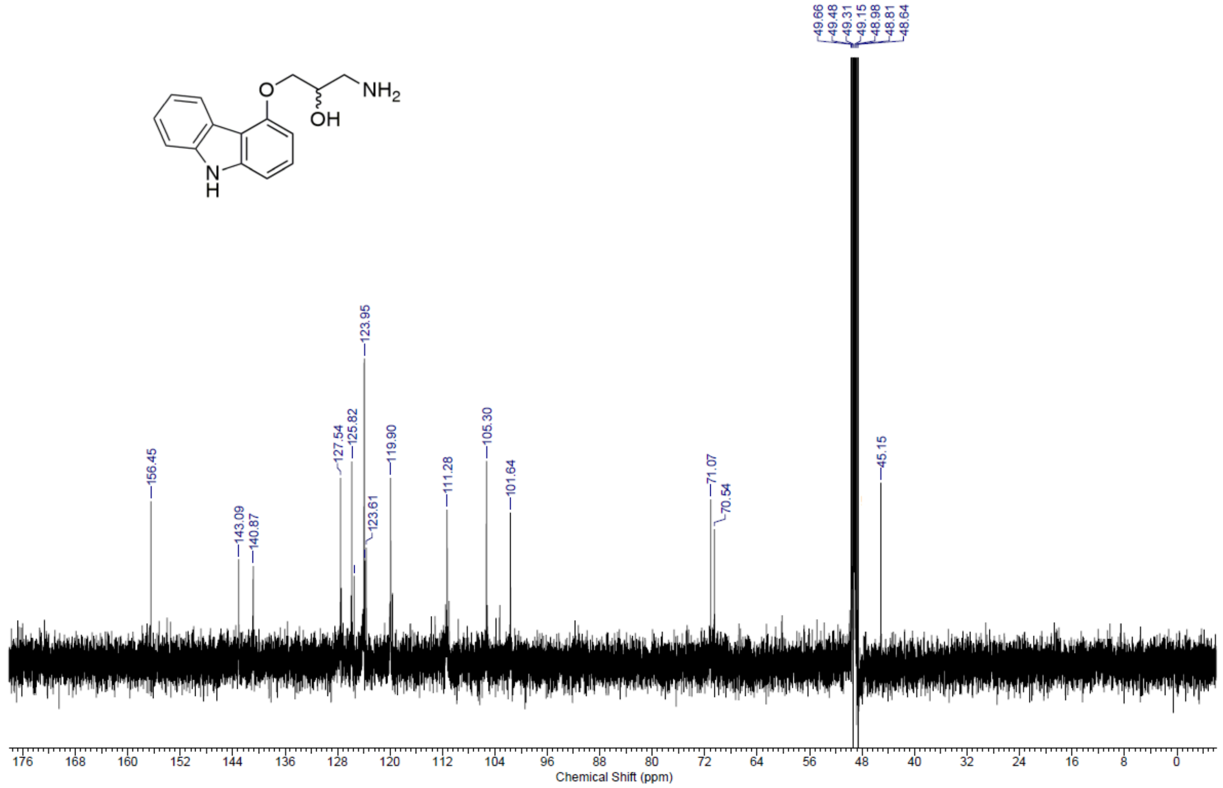

HRMS spectrum of *rac*-**7d** (ESI-TOF)

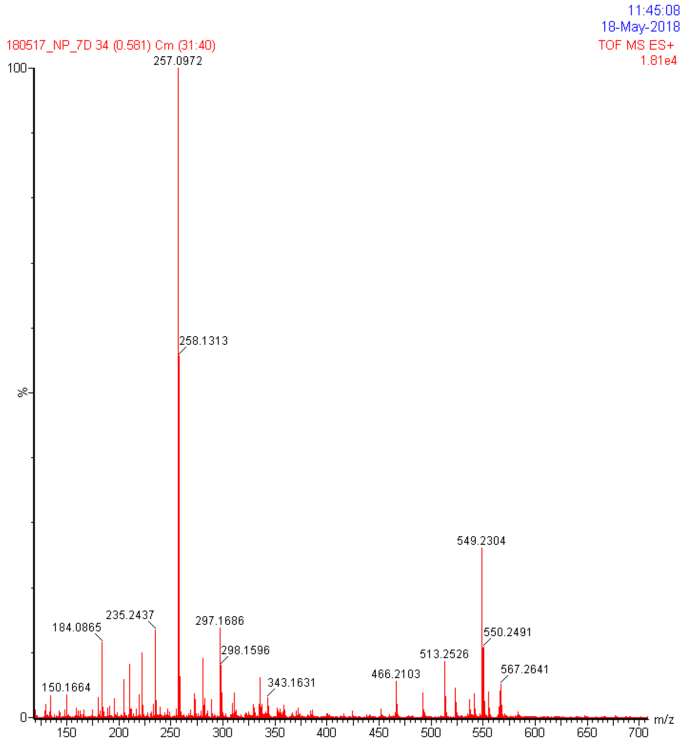

FTMS spectrum of *rac*-**7d** (ESI-TOF)

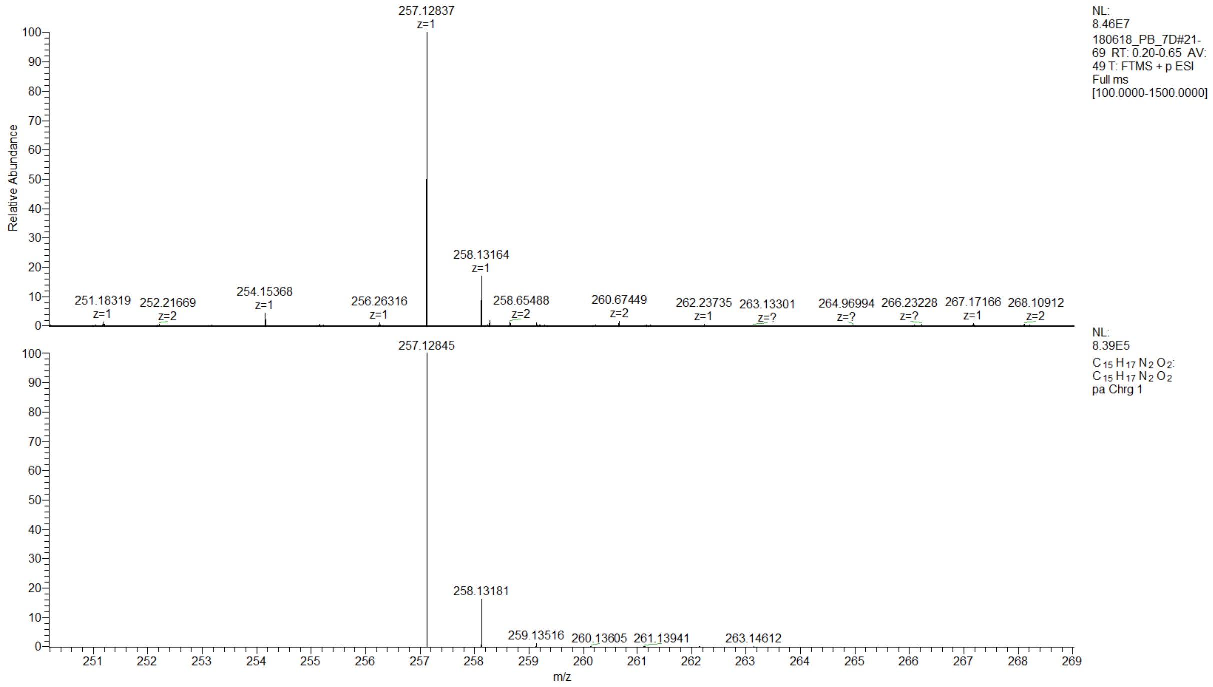

IR spectrum of *rac*-**7d** (Mineral oil, Nujol)

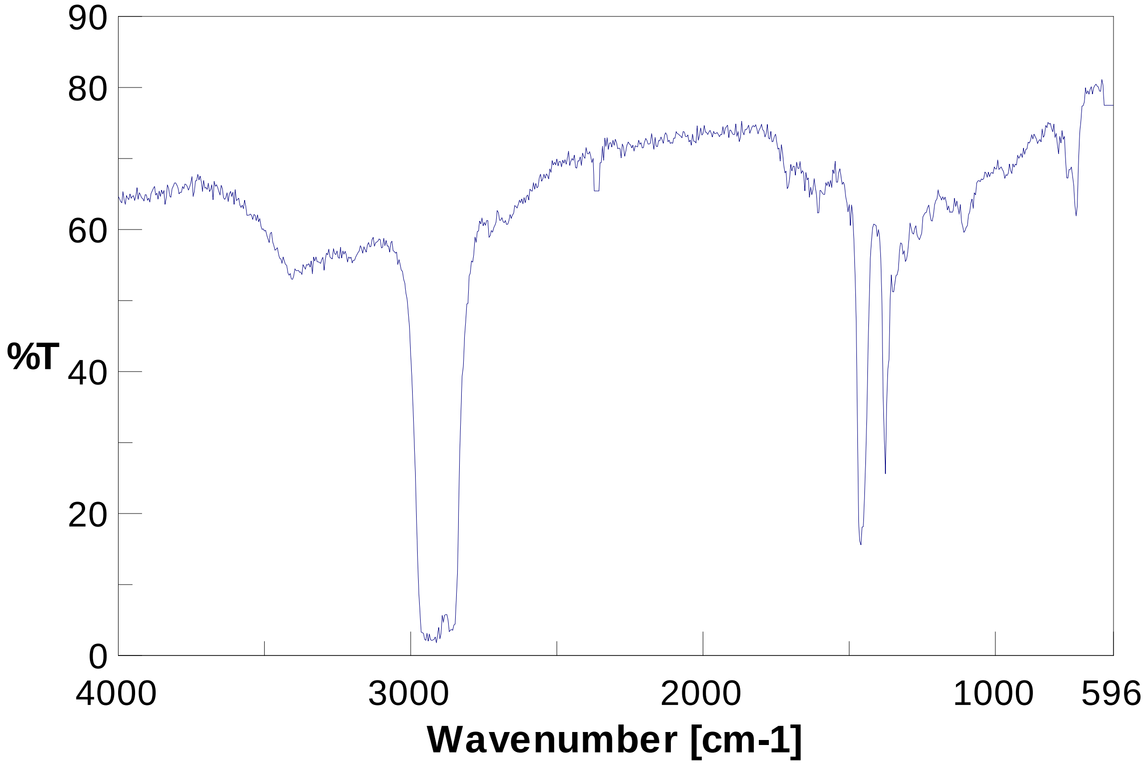

1-Amino-3-(2-methoxyphenoxy)propan-2-ol (rac-7e)

^{1}H NMR spectrum of *rac*-**7e** (500 MHz, $CDCl_3$)

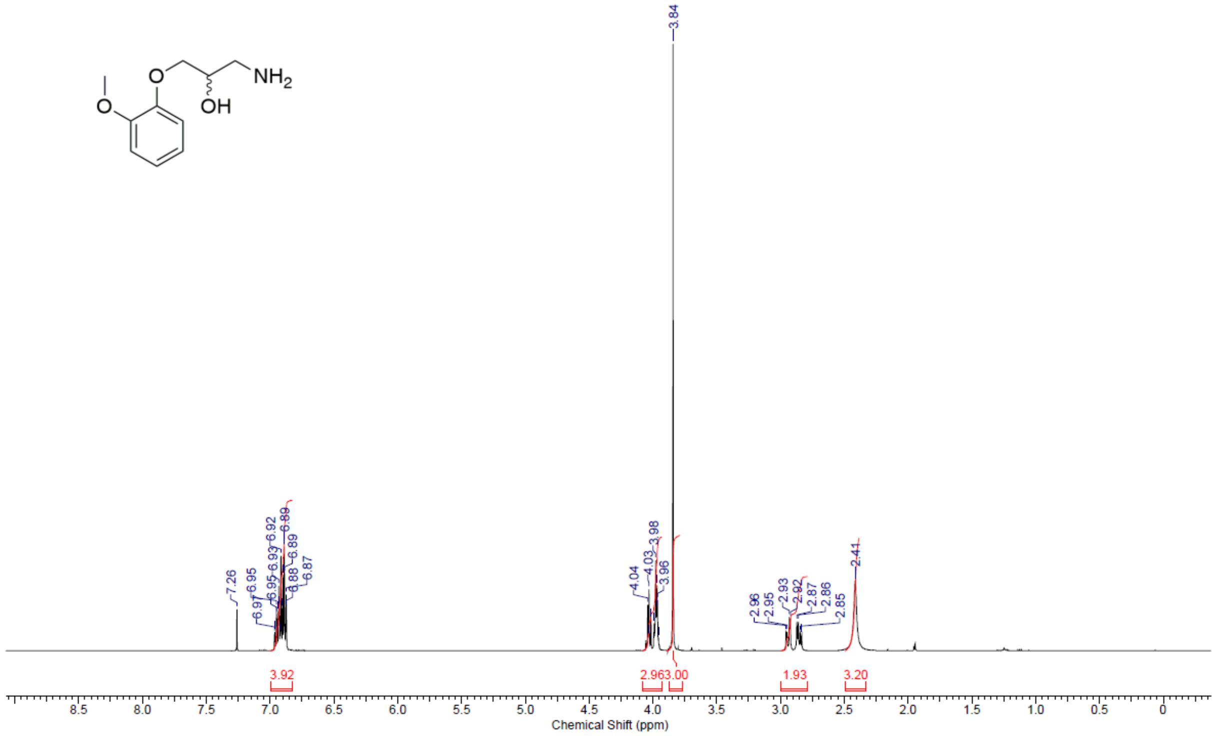

^{13}C NMR spectrum of *rac*-**7e** (126 MHz, $CDCl_3$)

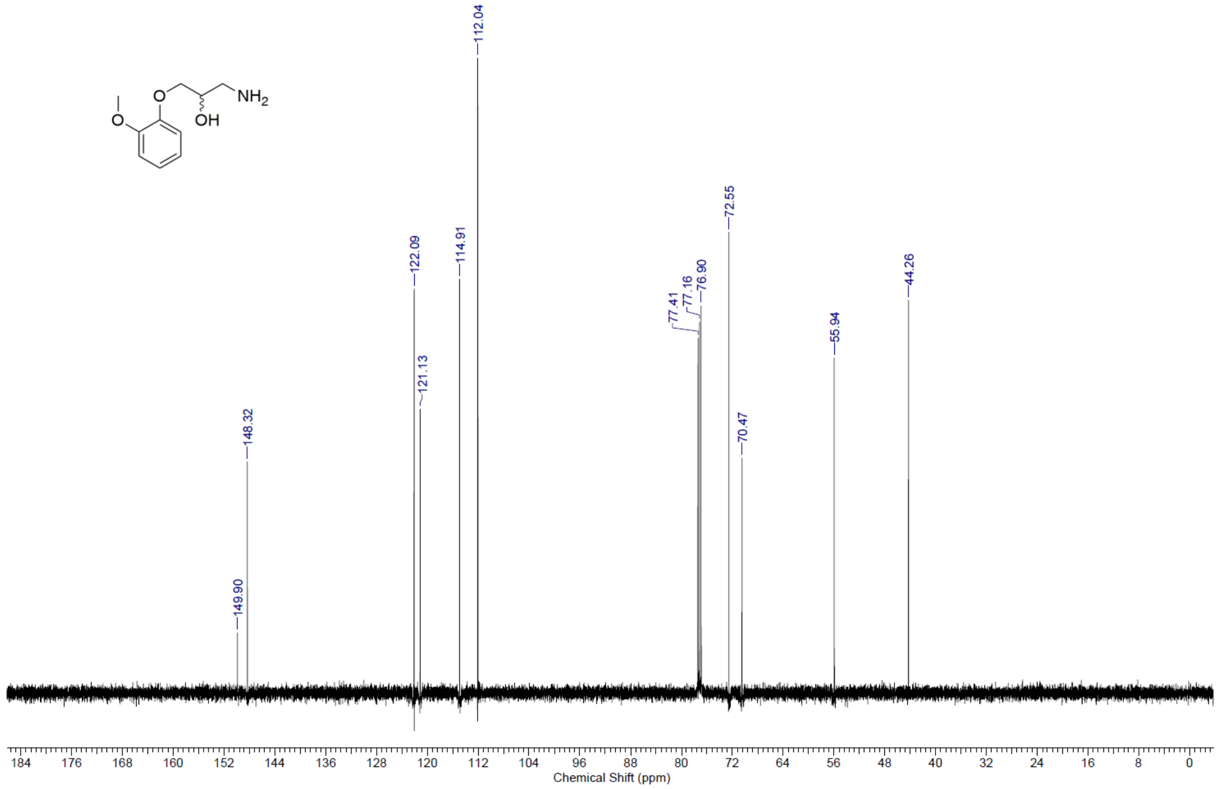

FTMS spectrum of *rac*-**7e** (ESI-TOF)

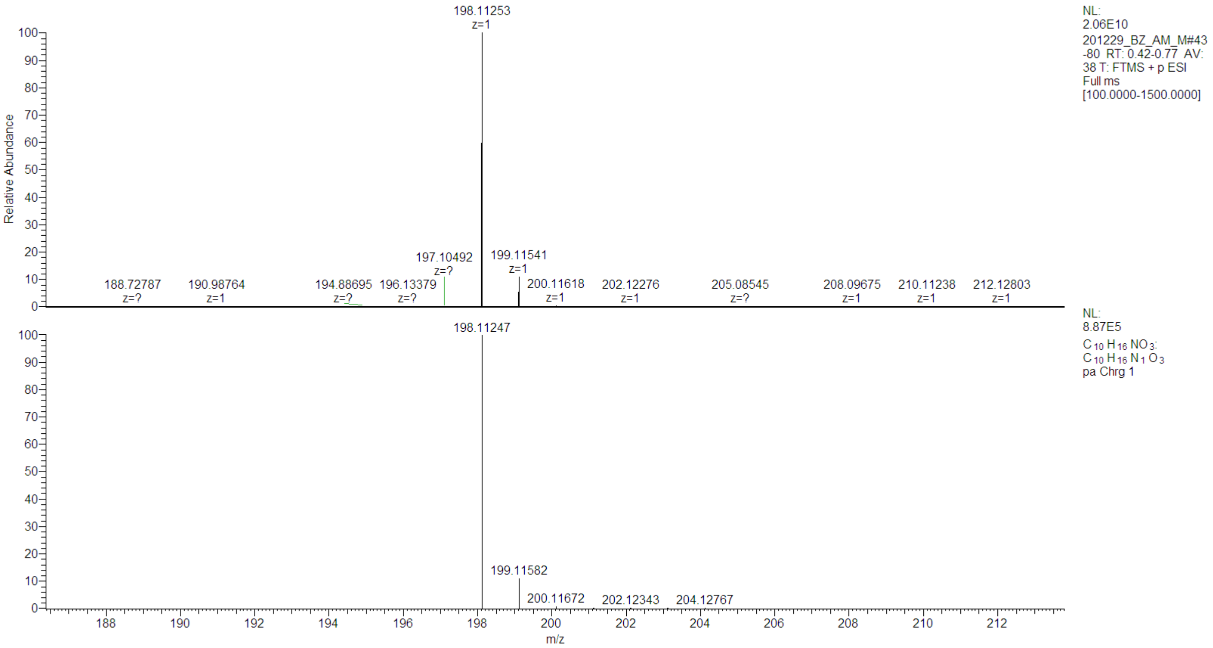

IR spectrum of *rac*-**7e** (Mineral oil, Nujol)

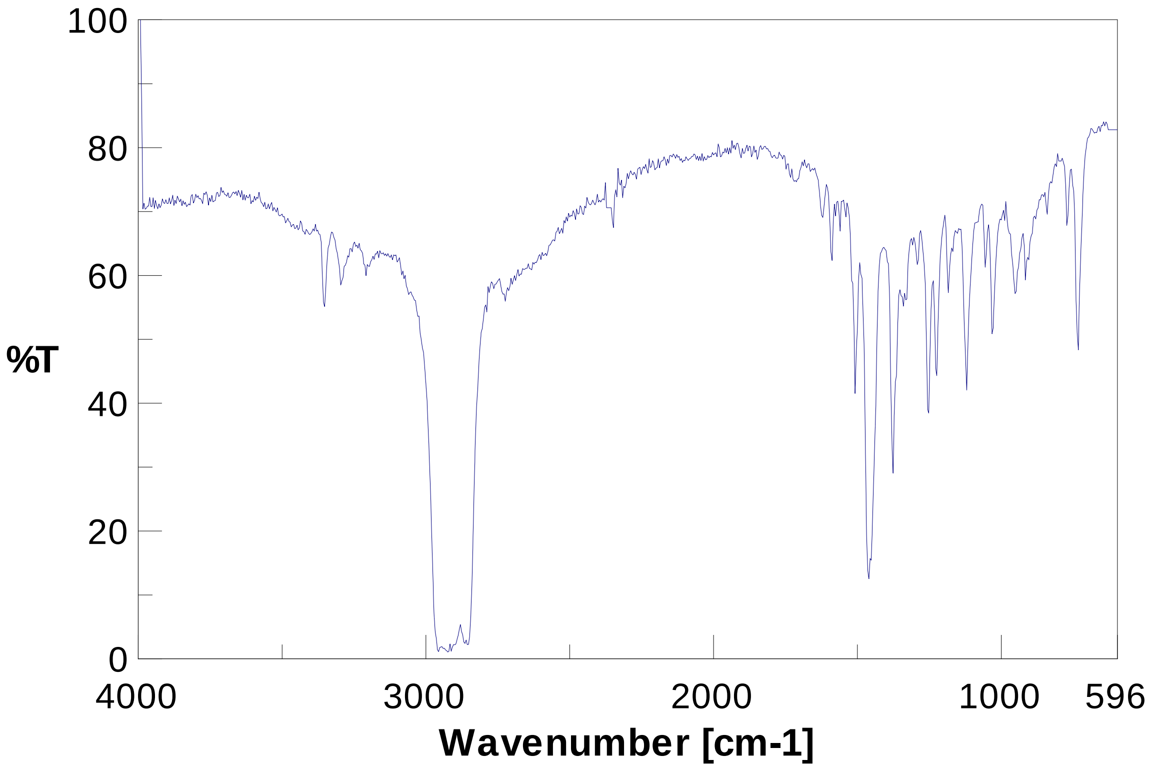

1-Amino-3-[4-(2-methoxyethyl)phenoxy]propan-2-ol (rac-7f)

^{1}H NMR spectrum of *rac*-**7f** (500 MHz, DMSO-d_6)

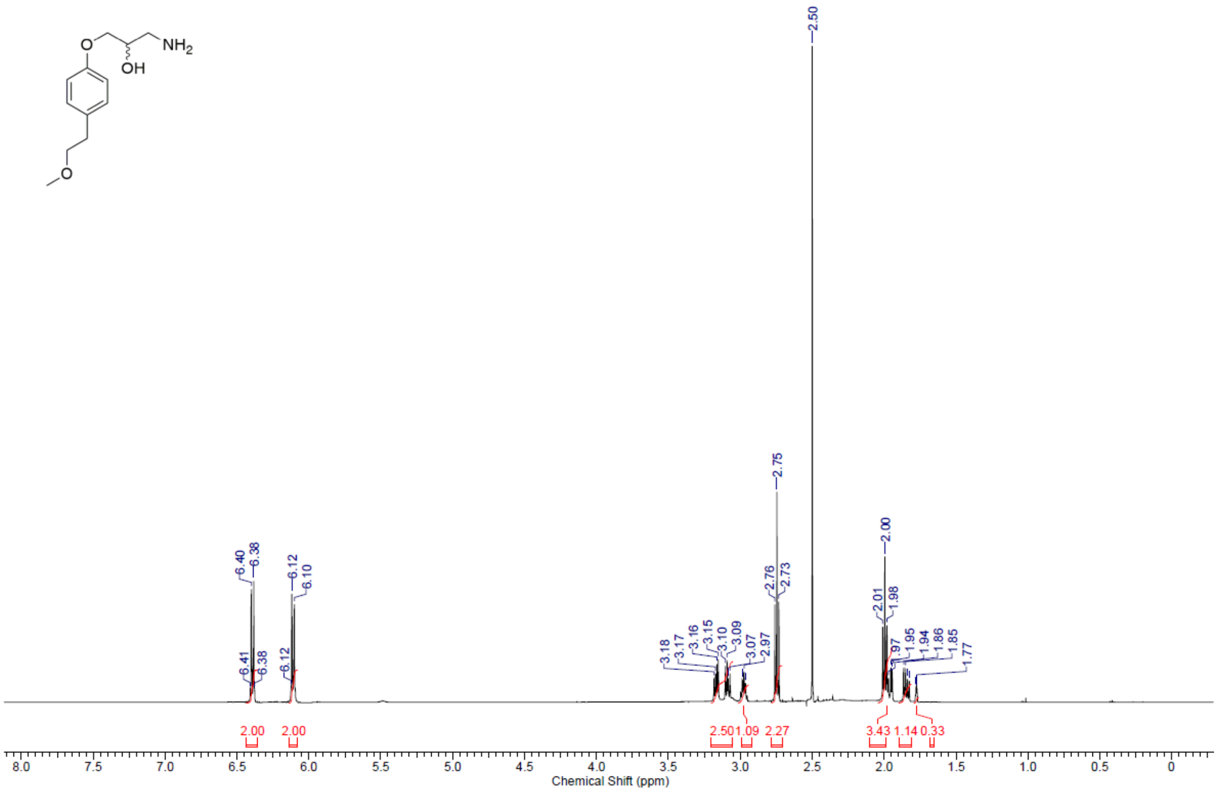

^{13}C NMR spectrum of *rac*-**7f** (126 MHz, DMSO-d_6)

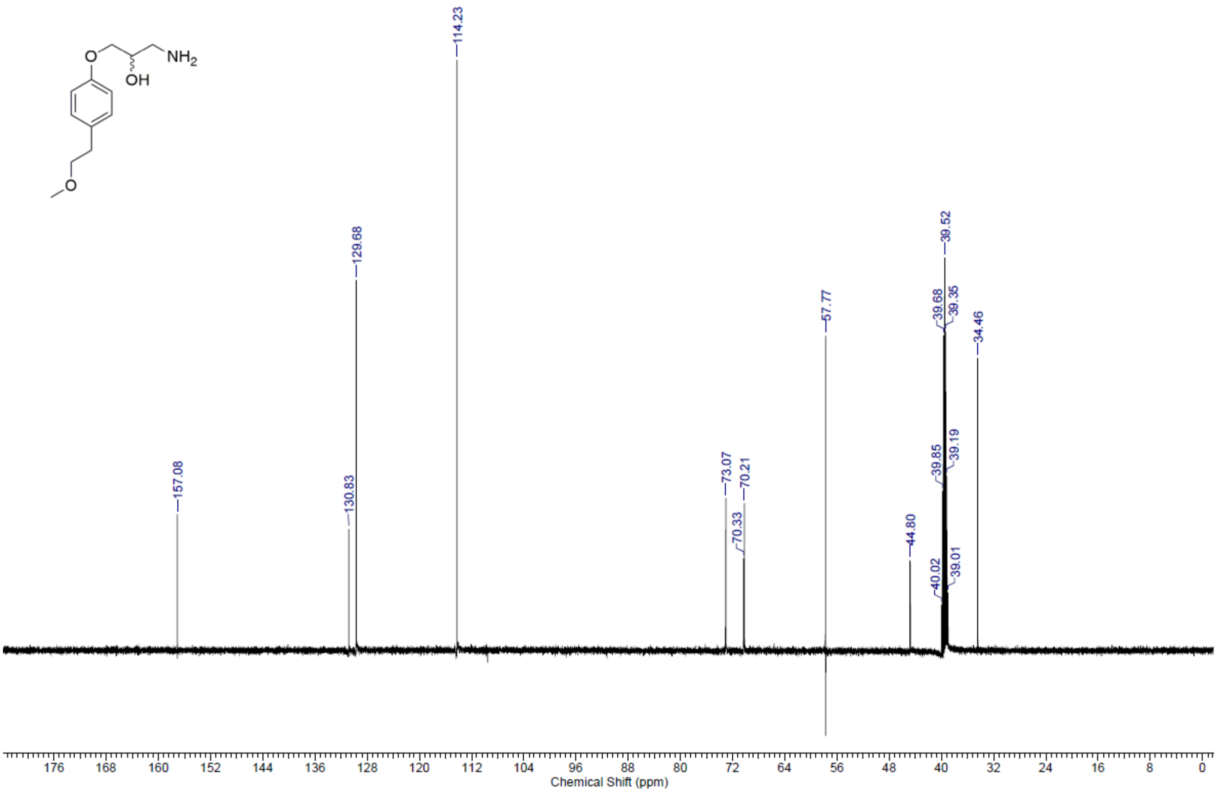

FTMS spectrum of *rac*-**7f** (ESI-TOF)

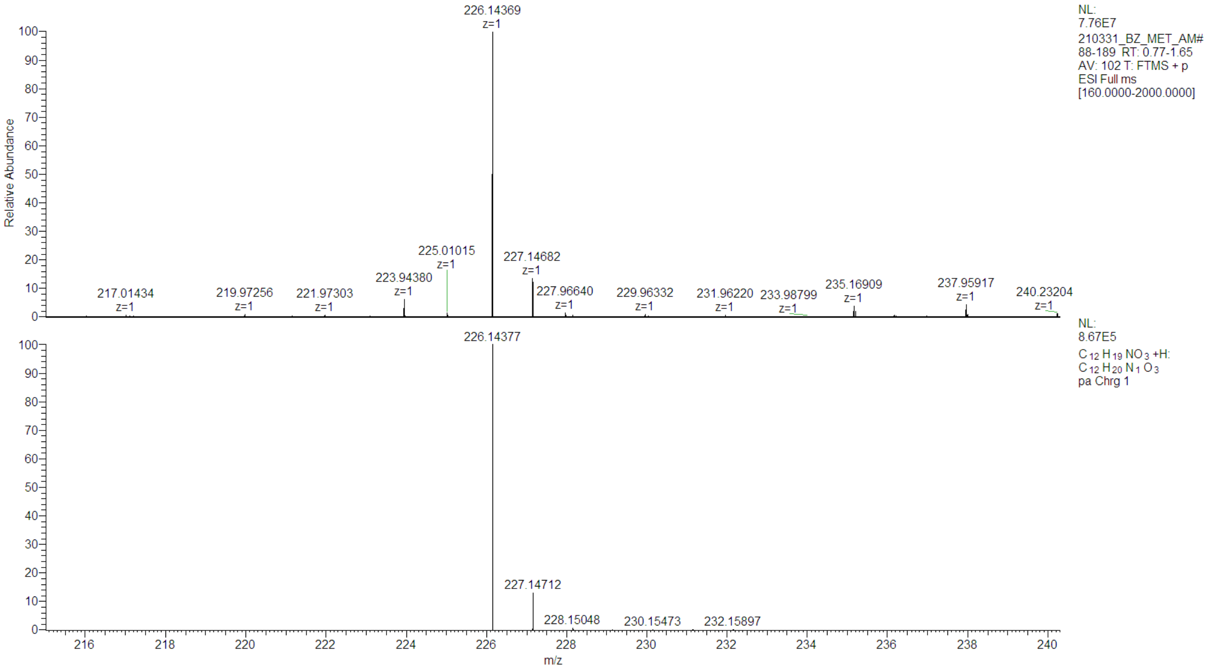

IR spectrum of *rac*-**7f** (Mineral oil, Nujol)

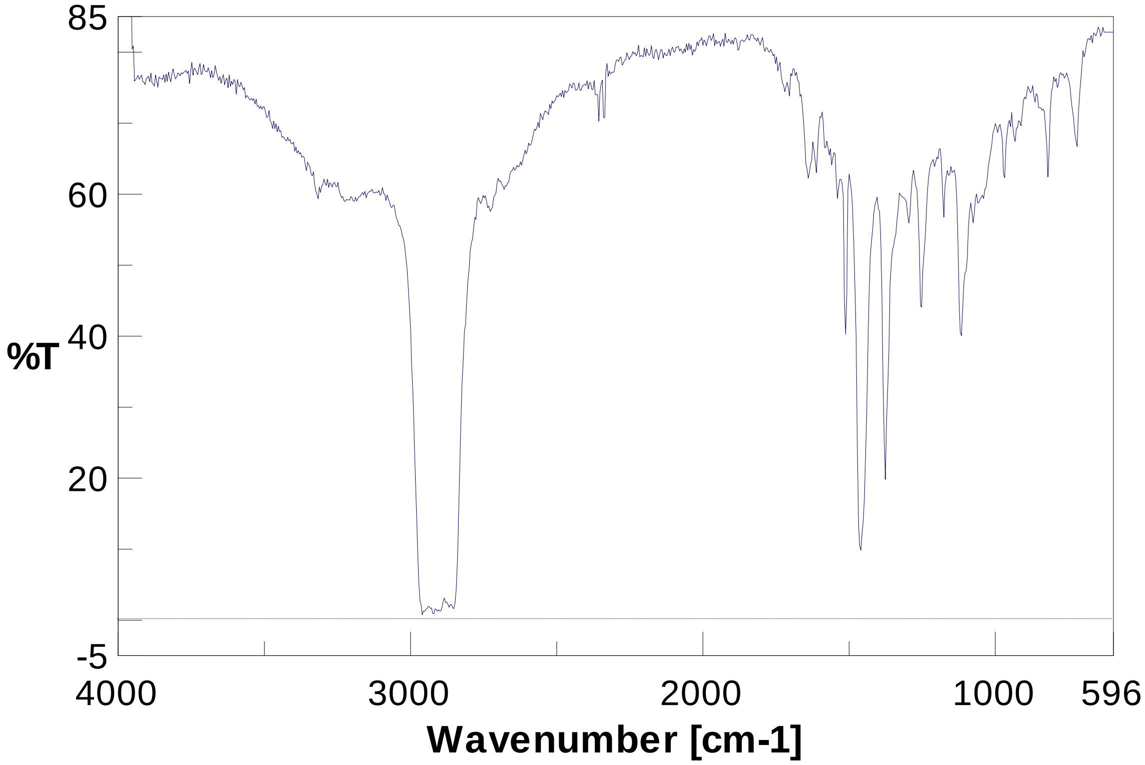

1-[(Naphthalen-1-yl)oxy]-3-[(propan-2-yl)amino]propan-2-ol (Propranolol, rac-8a)

^{1}H NMR spectrum of *rac*-**8a** (500 MHz, $CDCl_3$)

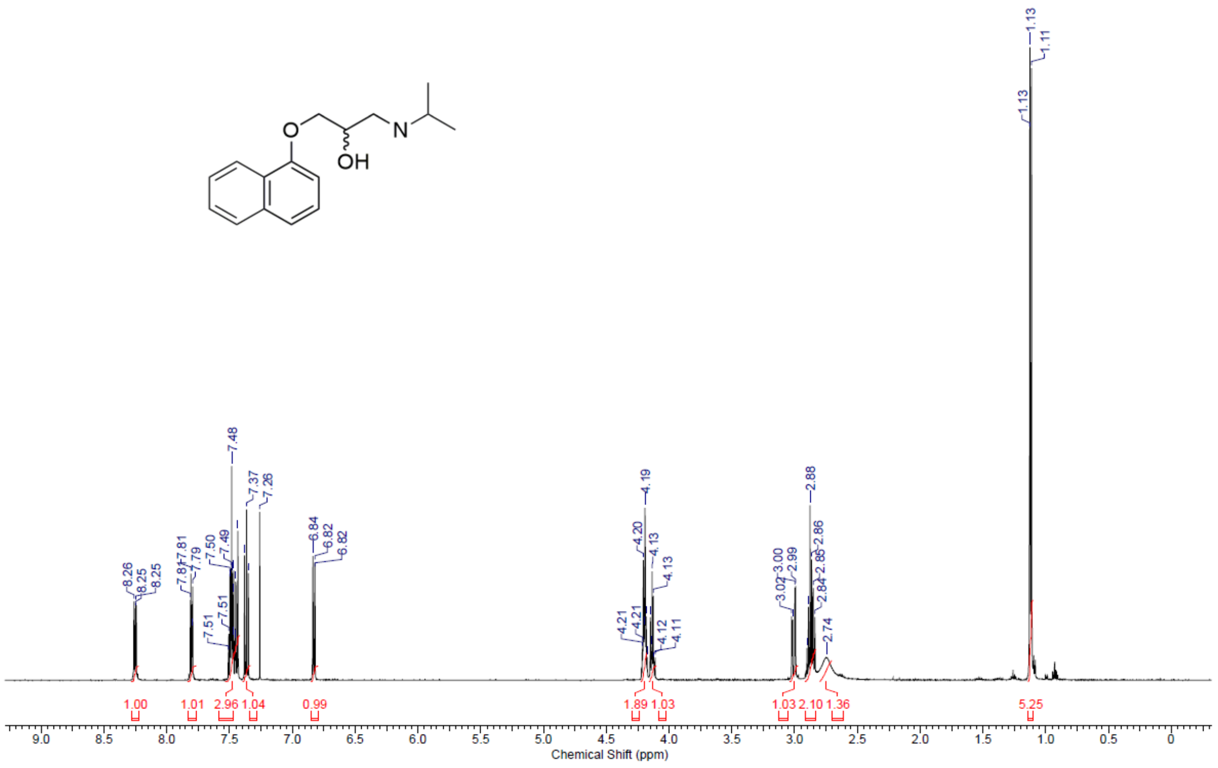

^{13}C NMR spectrum of *rac*-**8a** (126 MHz, $CDCl_3$)

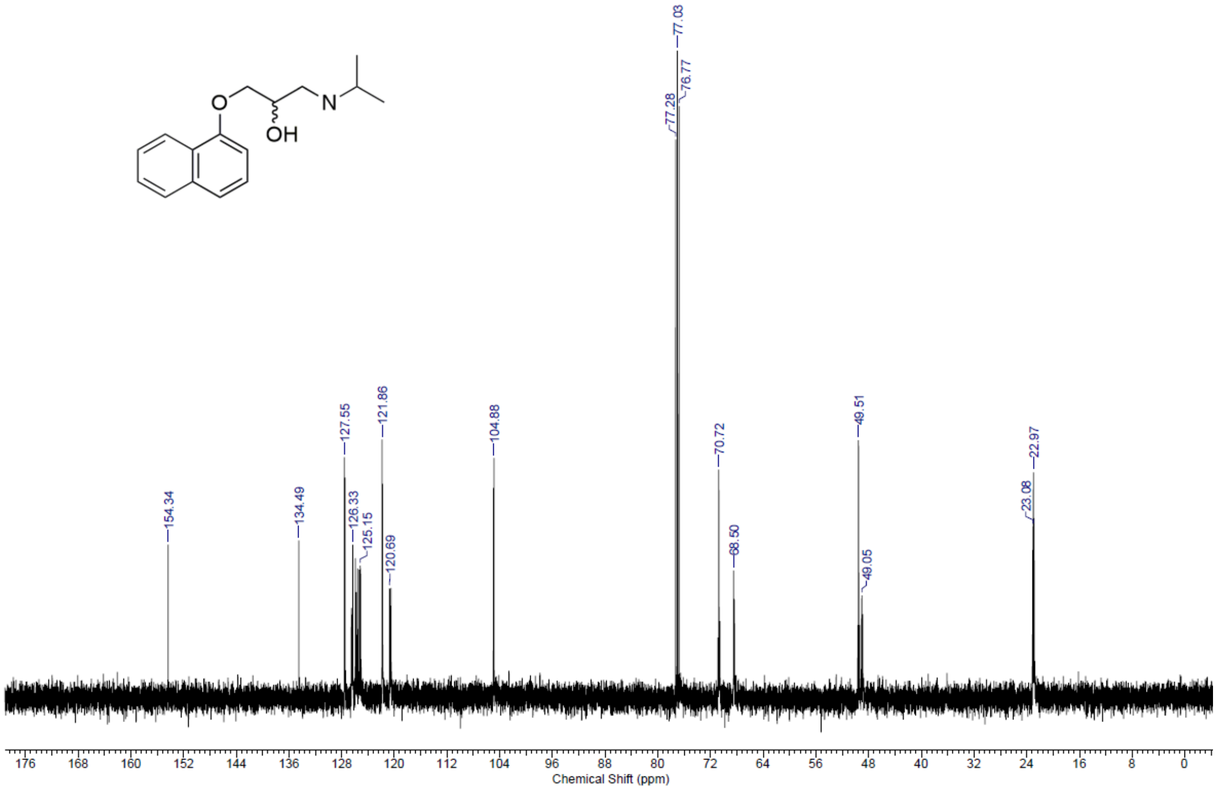

HRMS spectrum of *rac*-**8a** (ESI-TOF)

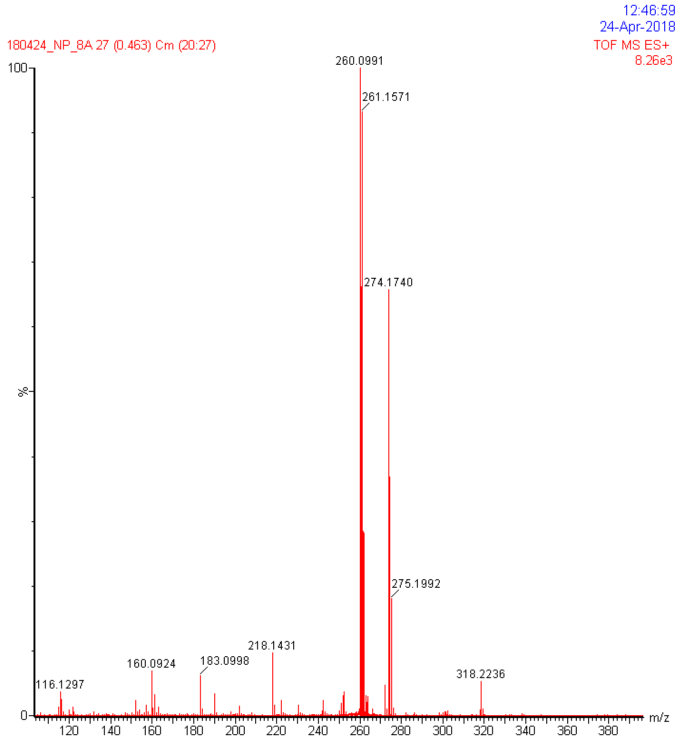

FTMS spectrum of *rac*-**8a** (ESI-TOF)

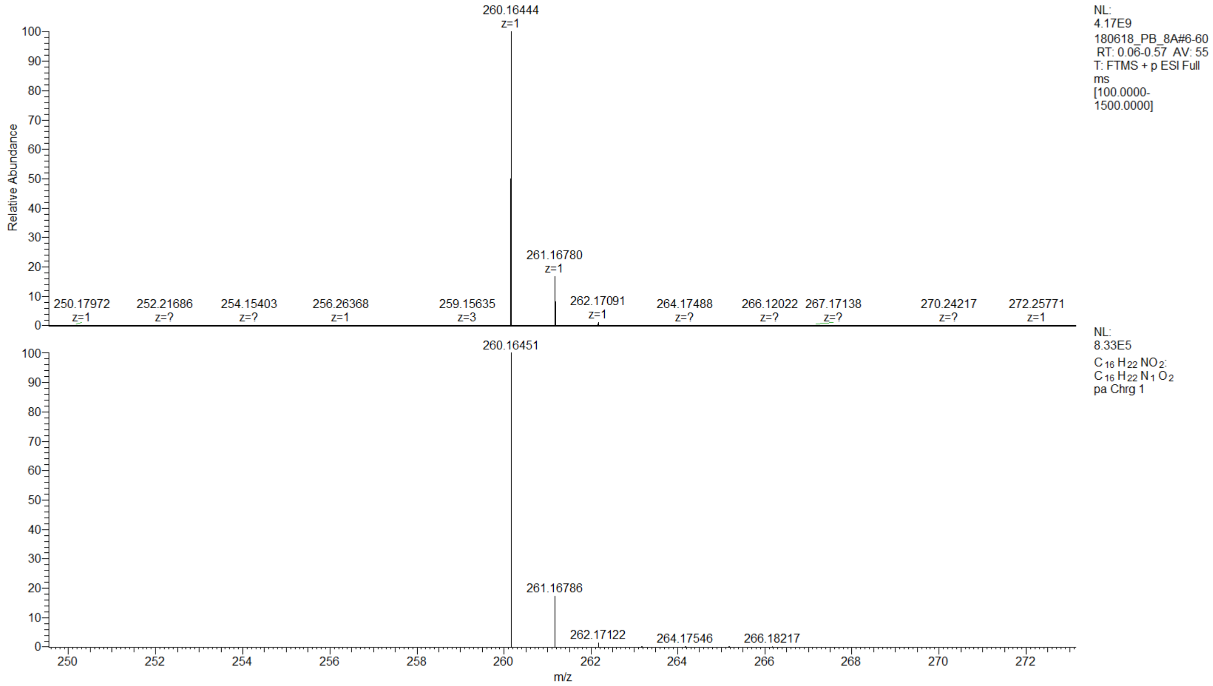

IR spectrum of *rac*-**8a** (Mineral oil, Nujol)

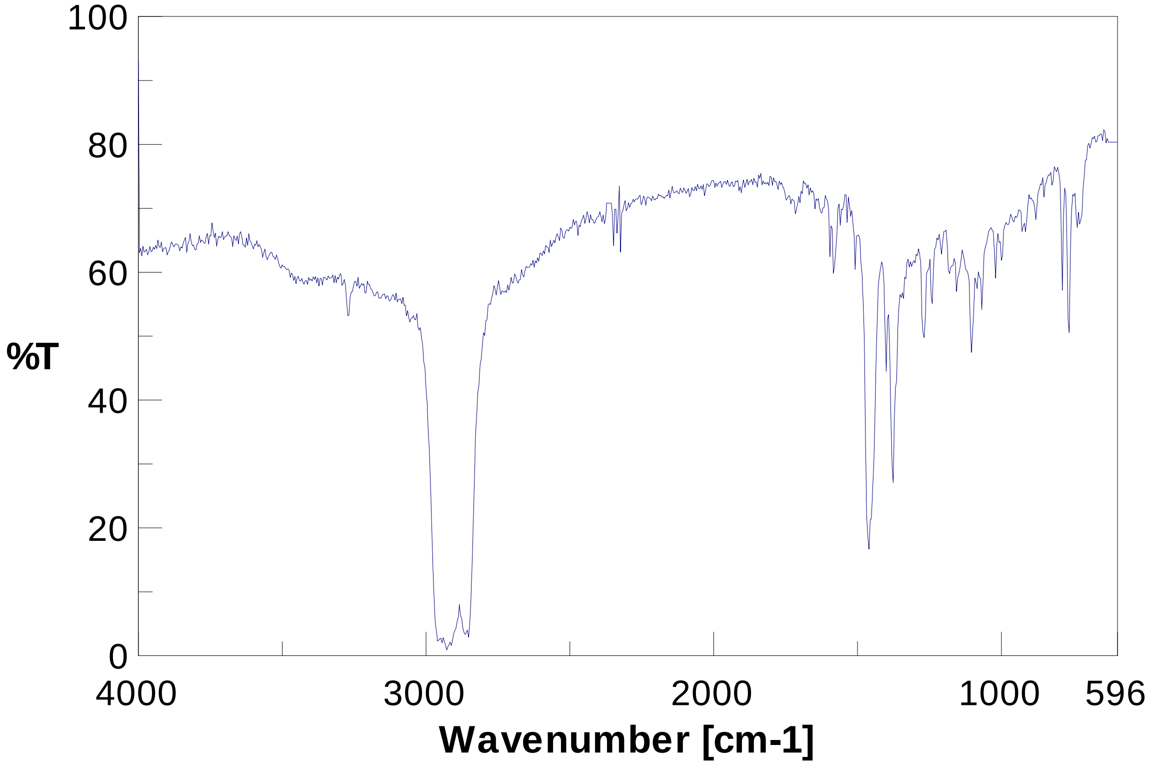

1-[(Propan-2-yl)amino]-3-[2-(prop-2-en-1-yl)phenoxy]propan-2-ol (Alprenolol, rac-8b)

^{1}H NMR spectrum of *rac*-**8b** (500 MHz, $CDCl_3$)

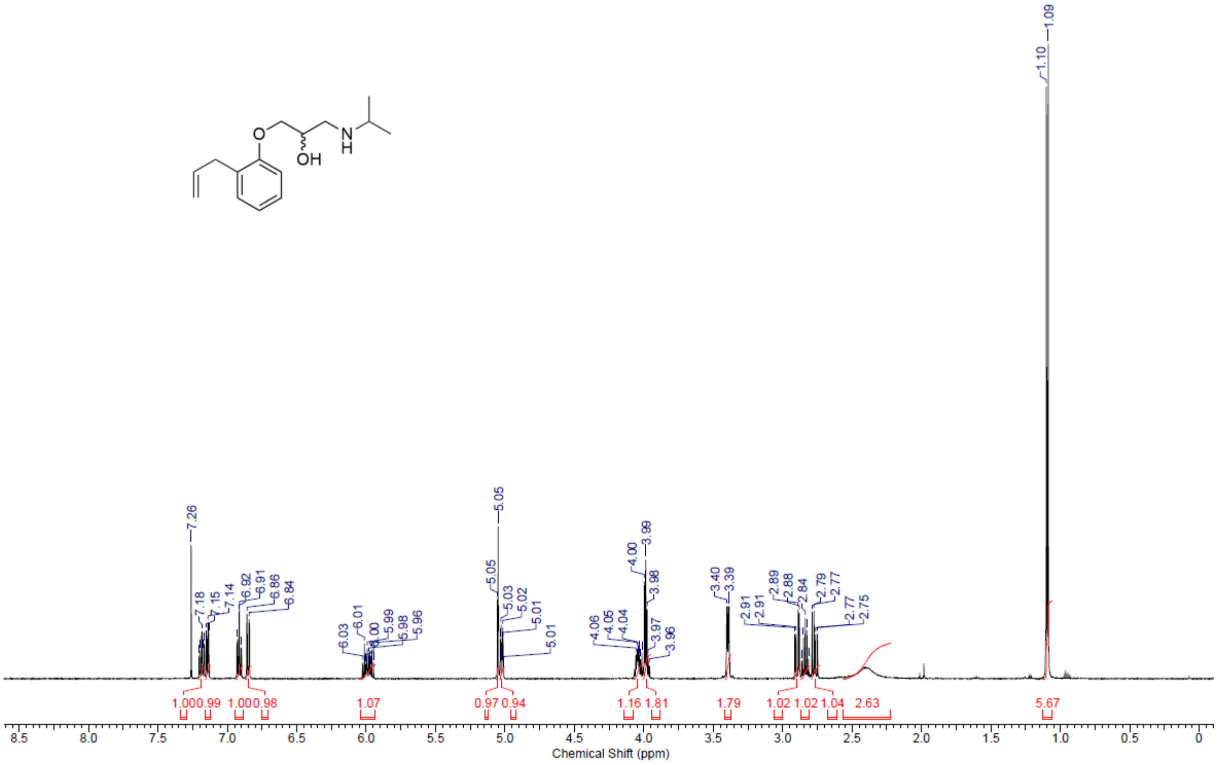

^{13}C NMR spectrum of *rac*-**8b** (126 MHz, $CDCl_3$)

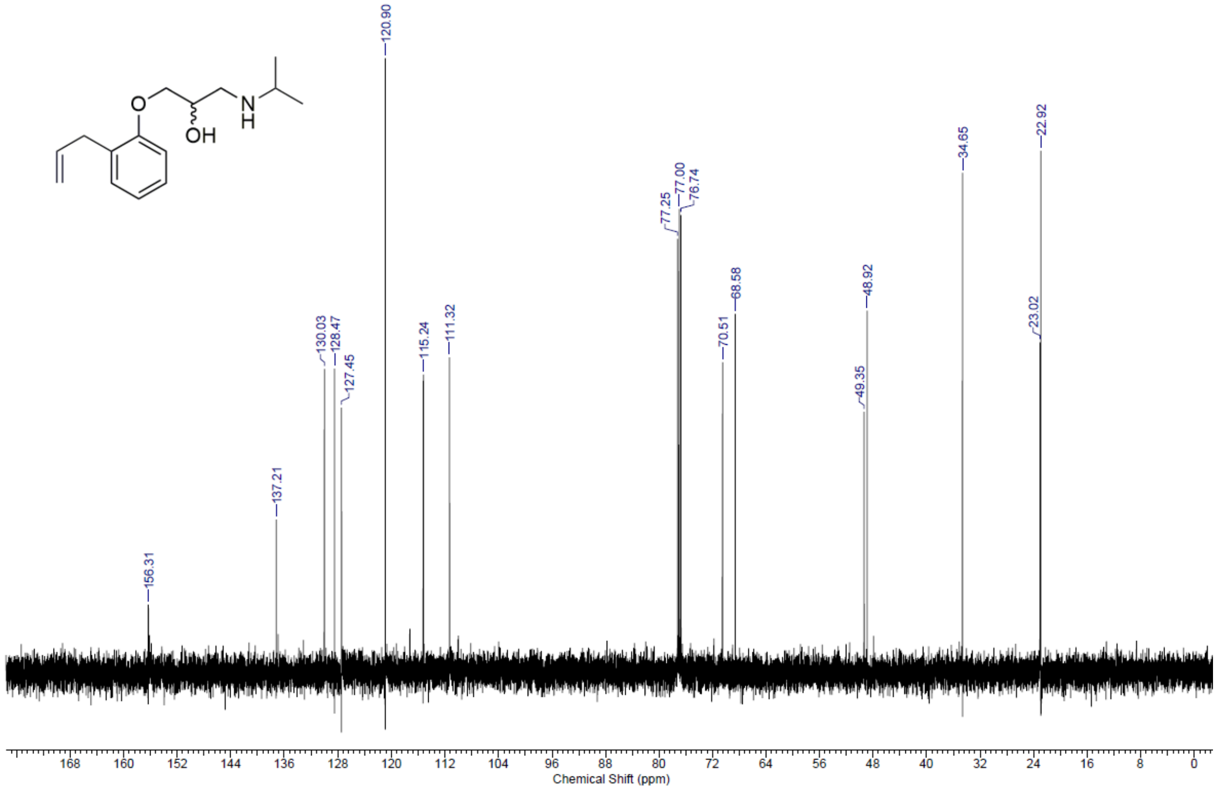

HRMS spectrum of *rac*-**8b** (ESI-TOF)

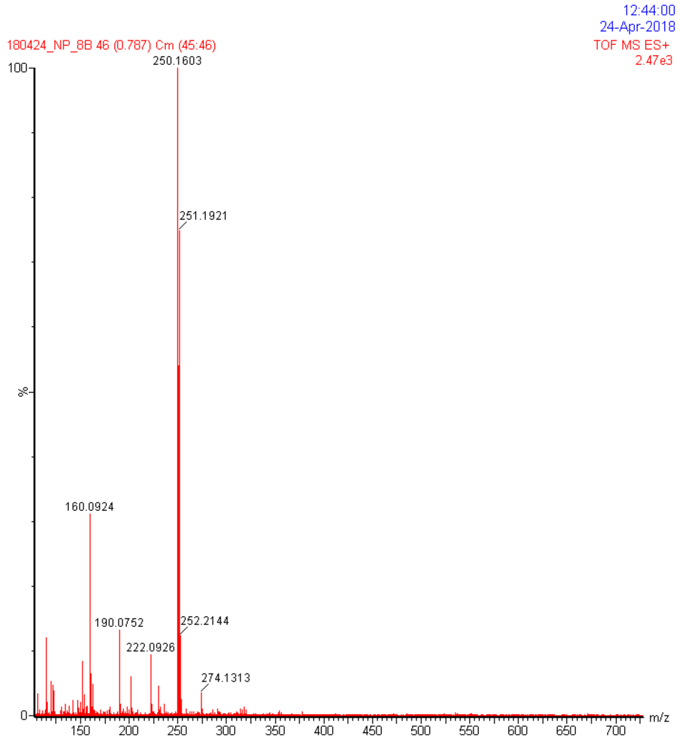

FTMS spectrum of *rac*-**8b** (ESI-TOF)

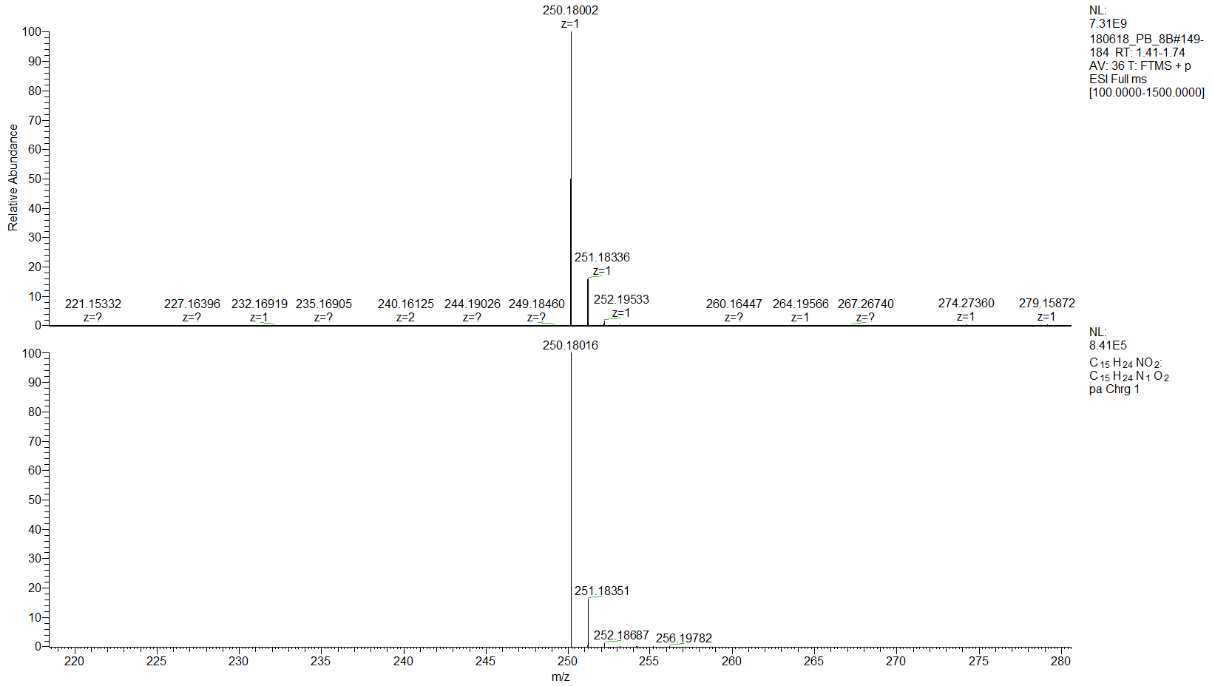

IR spectrum of *rac*-**8b** (Mineral oil, Nujol)

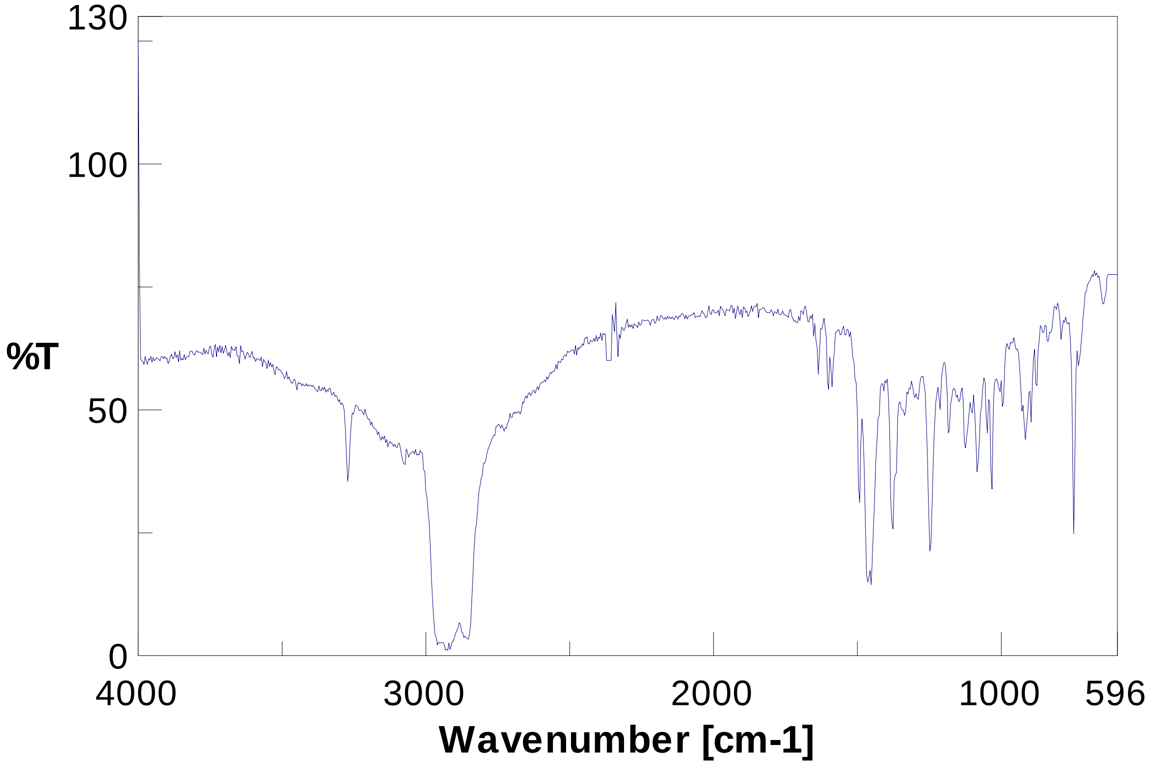

1-[(1H-Indol-4-yl)oxy]-3-[(propan-2-yl)amino]propan-2-ol (Pindolol, rac-8c)

^{1}H NMR spectrum of *rac*-**8c** (500 MHz, CD_3OD)

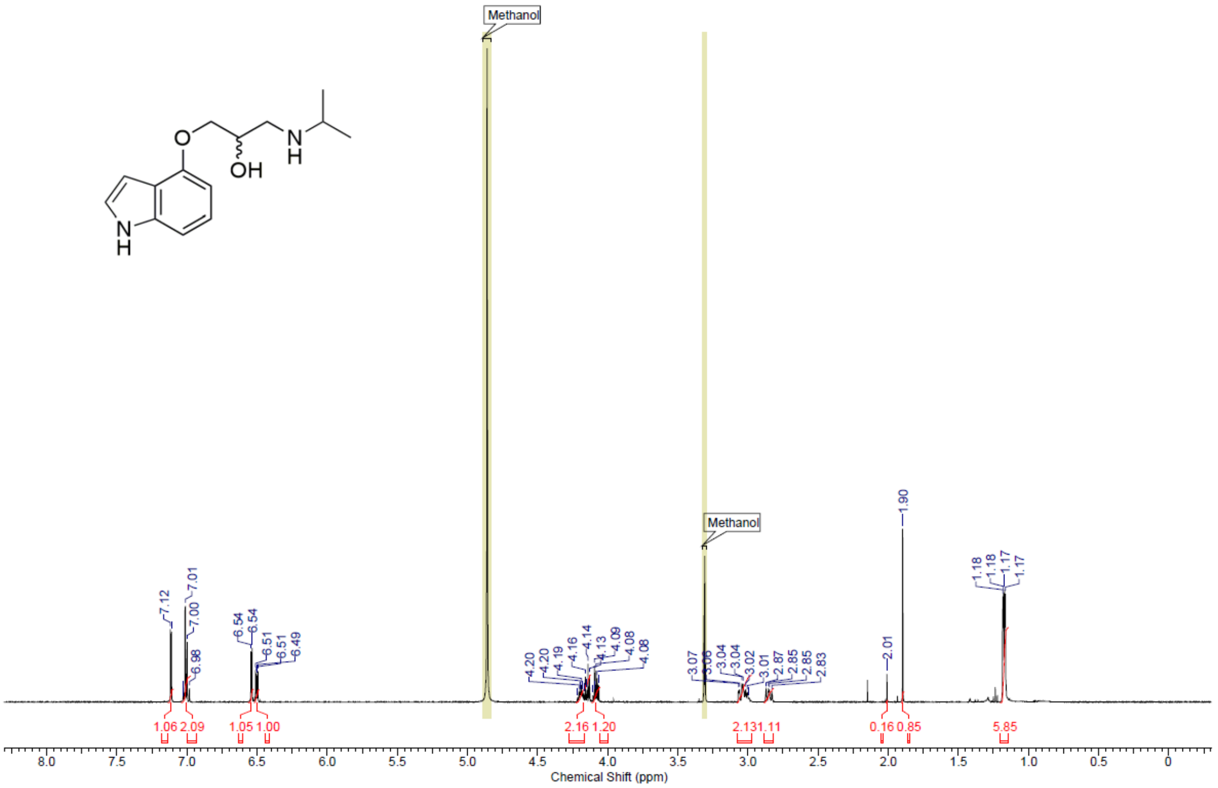

^{13}C NMR spectrum of *rac*-**8c** (126 MHz, CD_3OD)

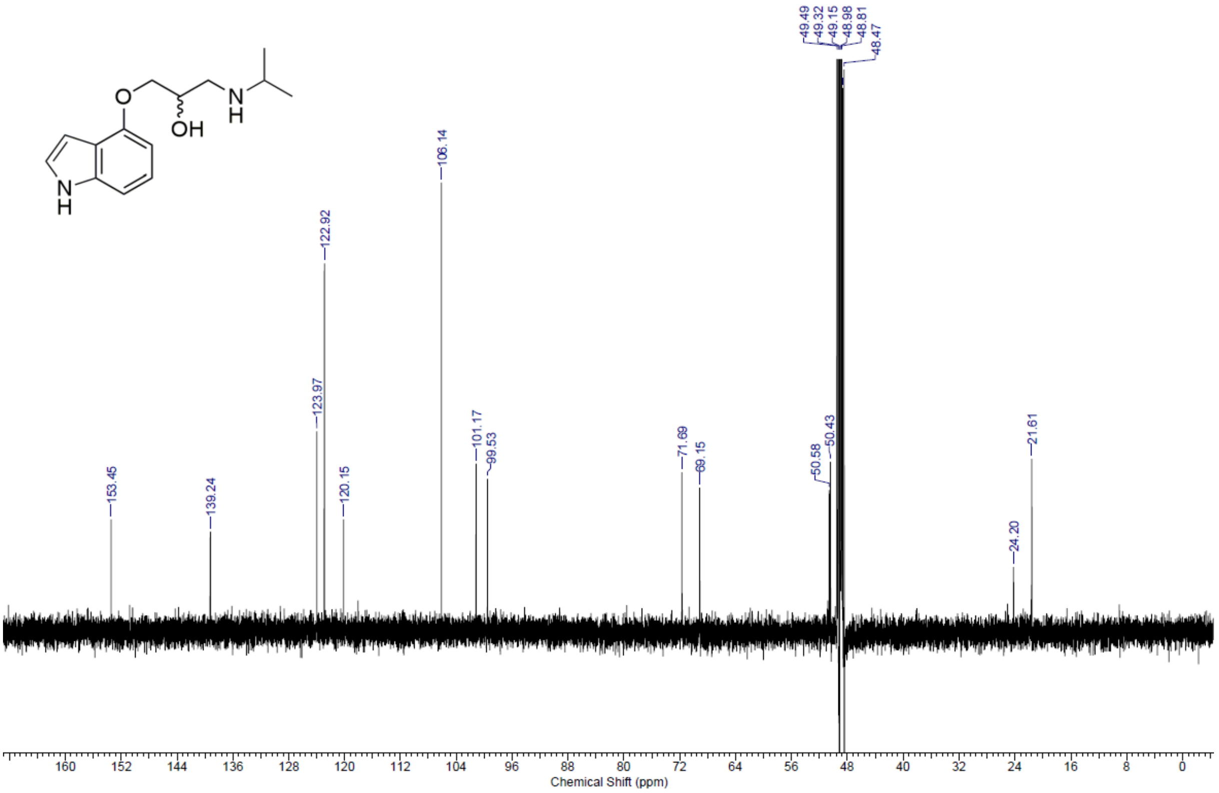

HRMS spectrum of *rac*-**8c** (ESI-TOF)

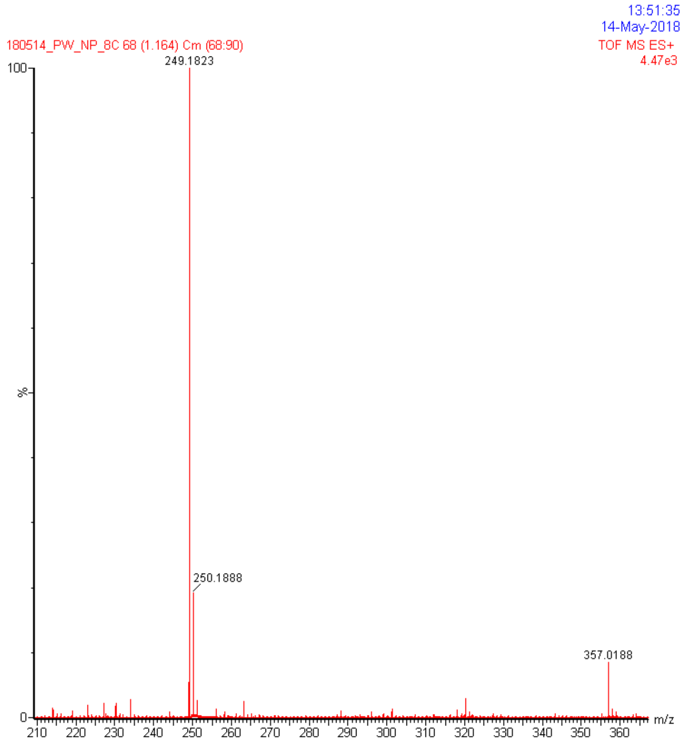

FTMS spectrum of *rac*-**8c** (ESI-TOF)

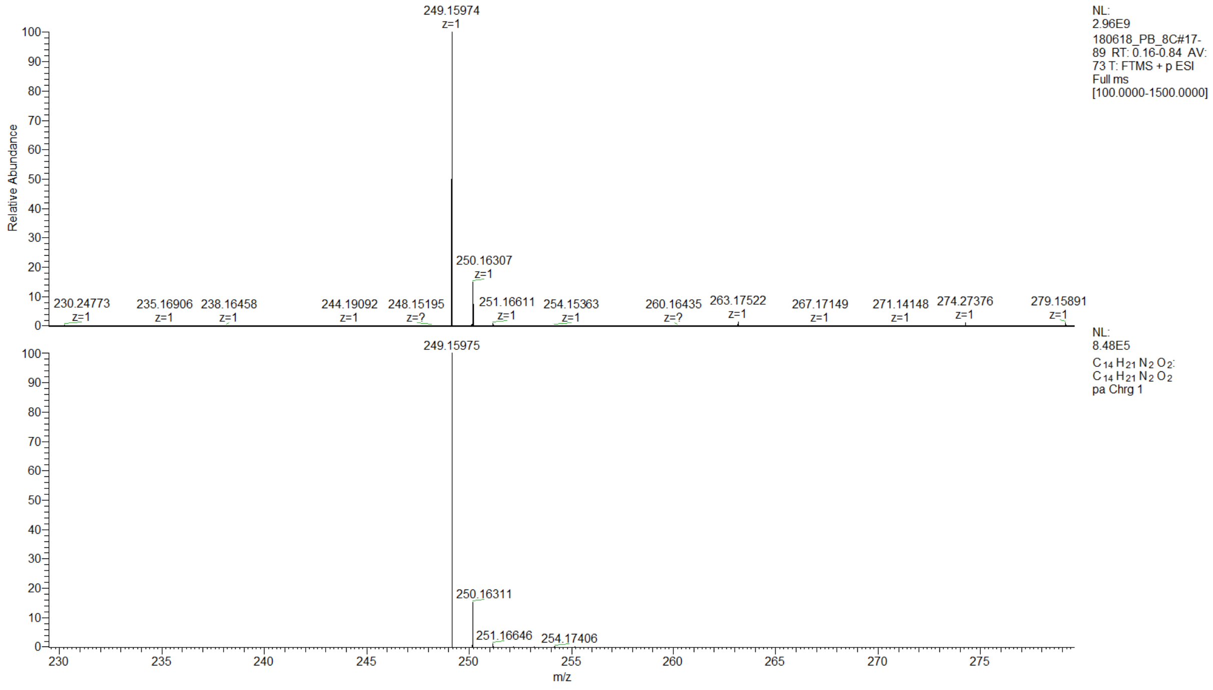

IR spectrum of *rac*-**8c** (Mineral oil, Nujol)

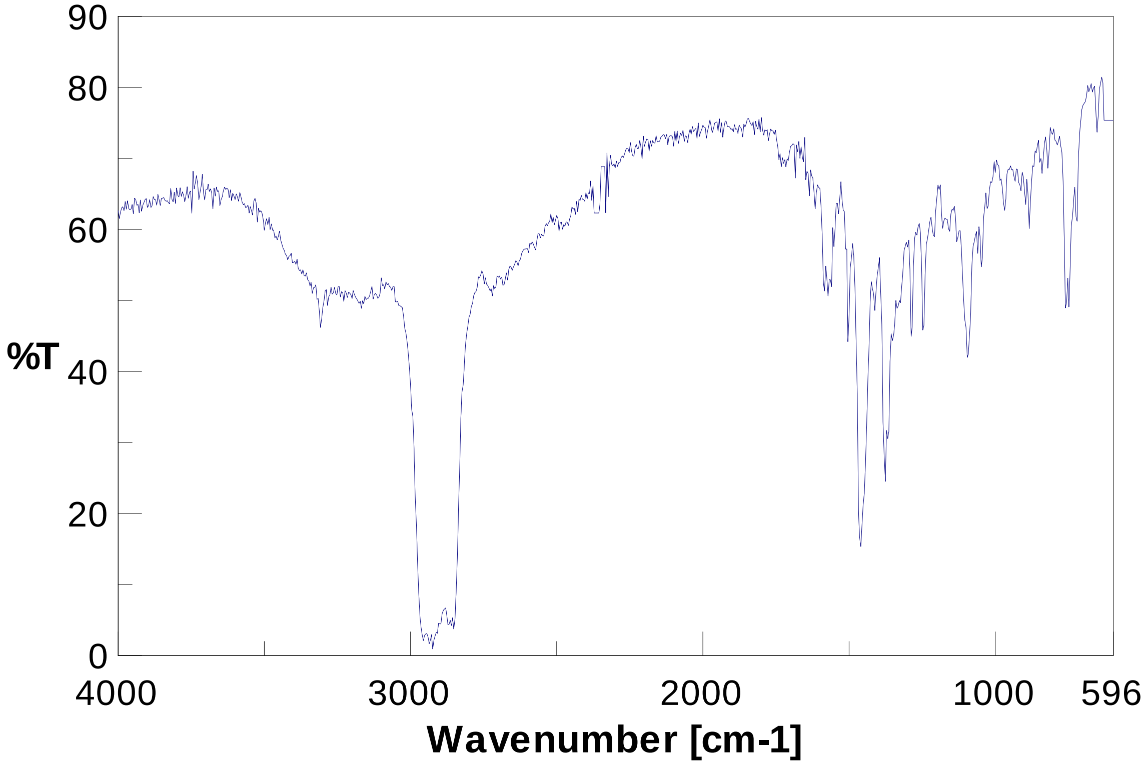

1-[(9H-Carbazol-4-yl)oxy]-3-[(propan-2-yl)amino]propan-2-ol (Carazolol, rac-8d)

^{1}H NMR spectrum of *rac*-**8d** (500 MHz, CD_3OD)

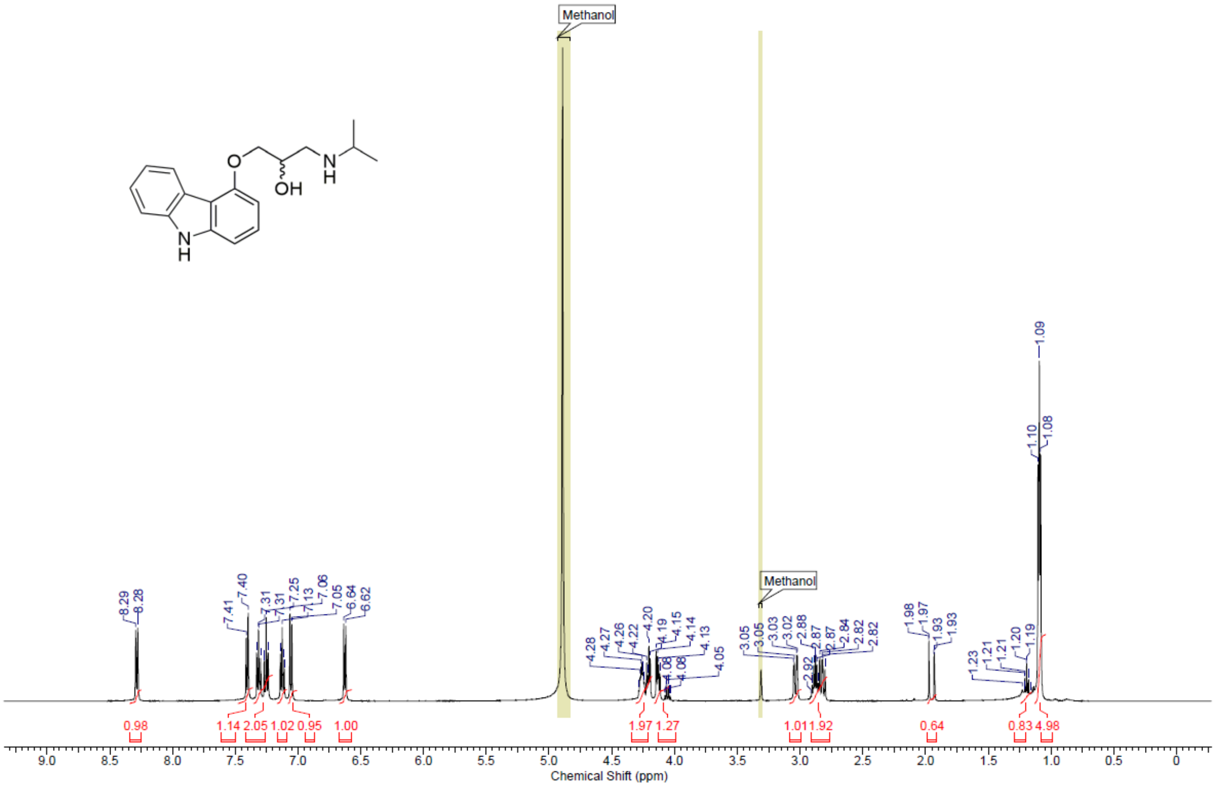

^{13}C NMR spectrum of *rac*-**8d** (126 MHz, CD_3OD)

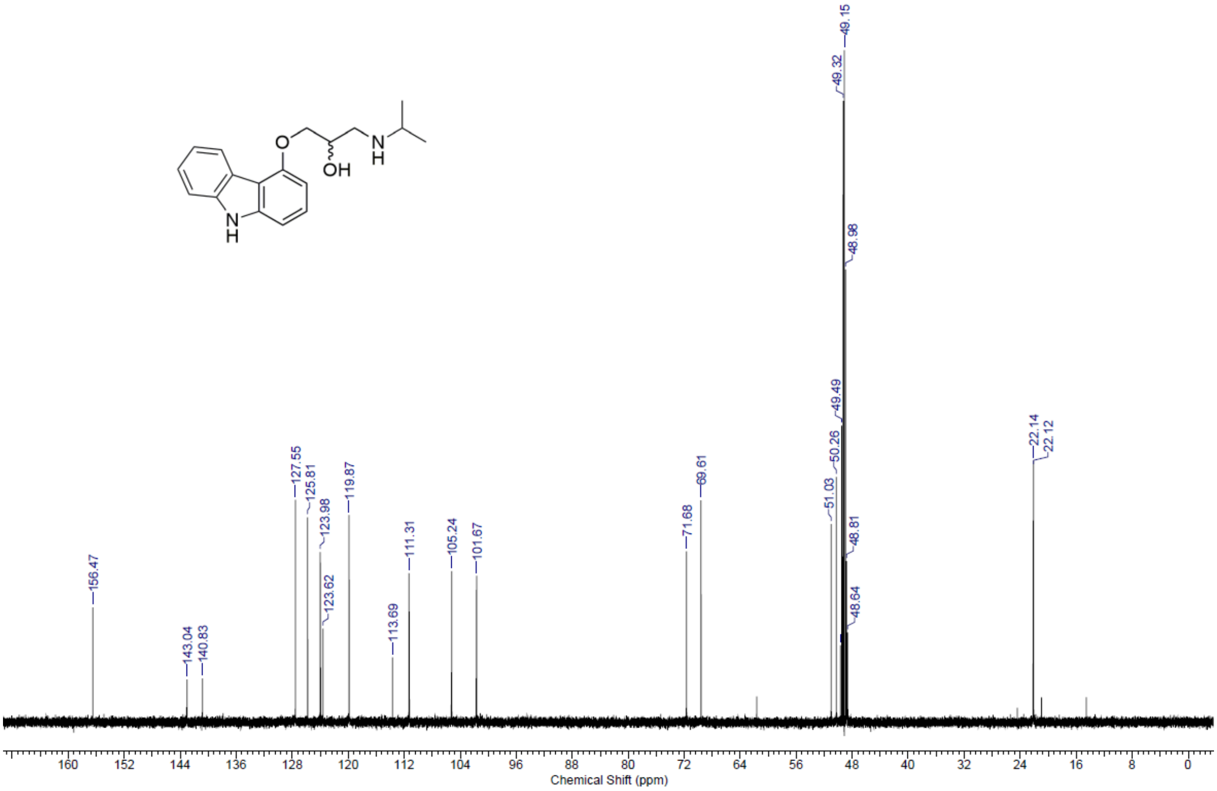

HRMS spectrum of *rac*-**8d** (ESI-TOF)

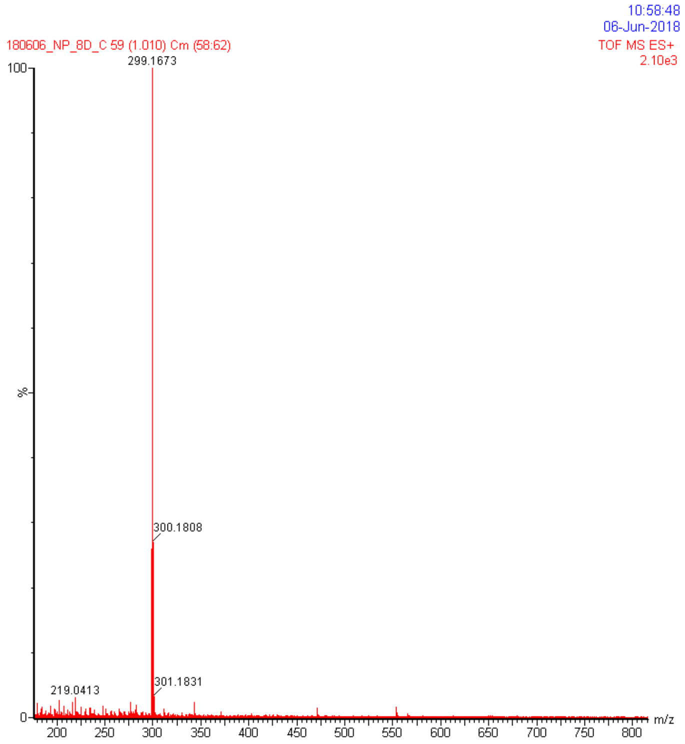

FTMS spectrum of *rac*-**8d** (ESI-TOF)

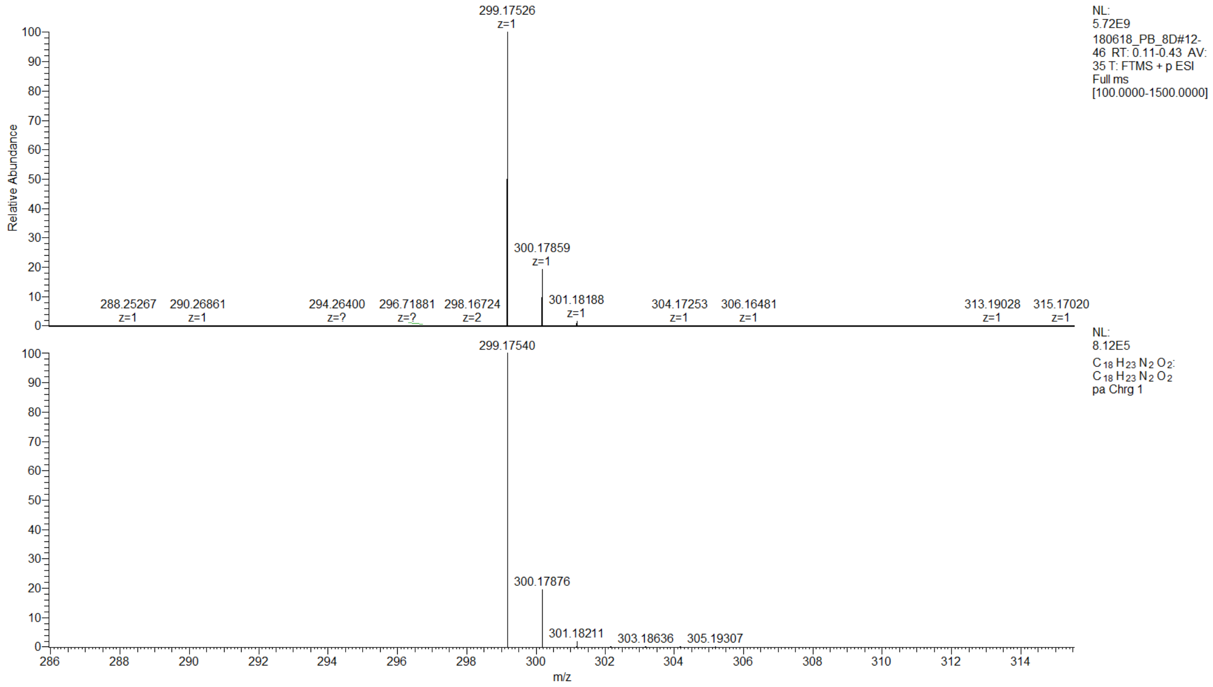

IR spectrum of *rac*-**8d** (Mineral oil, Nujol)

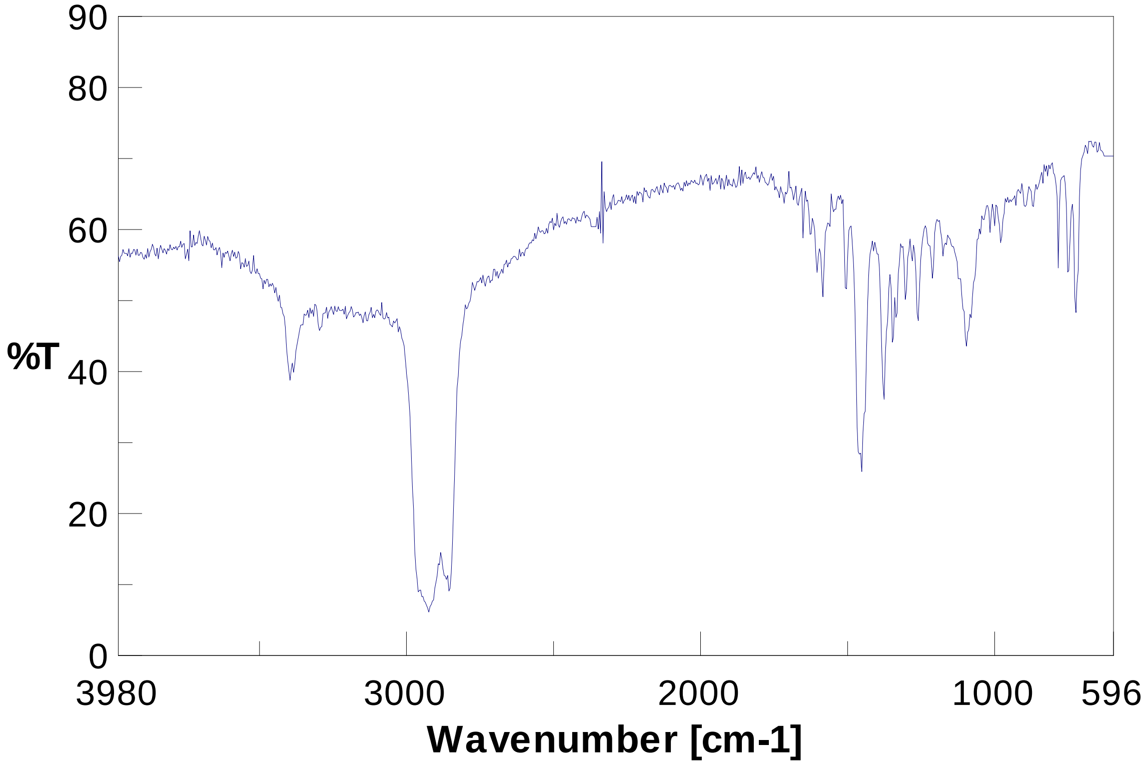

1-(2-Methoxyphenoxy)-3-[(propan-2-yl)amino]propan-2-ol (Moprolol, rac-8e)

^{1}H NMR spectrum of *rac*-**8e** (500 MHz, $CDCl_3$)

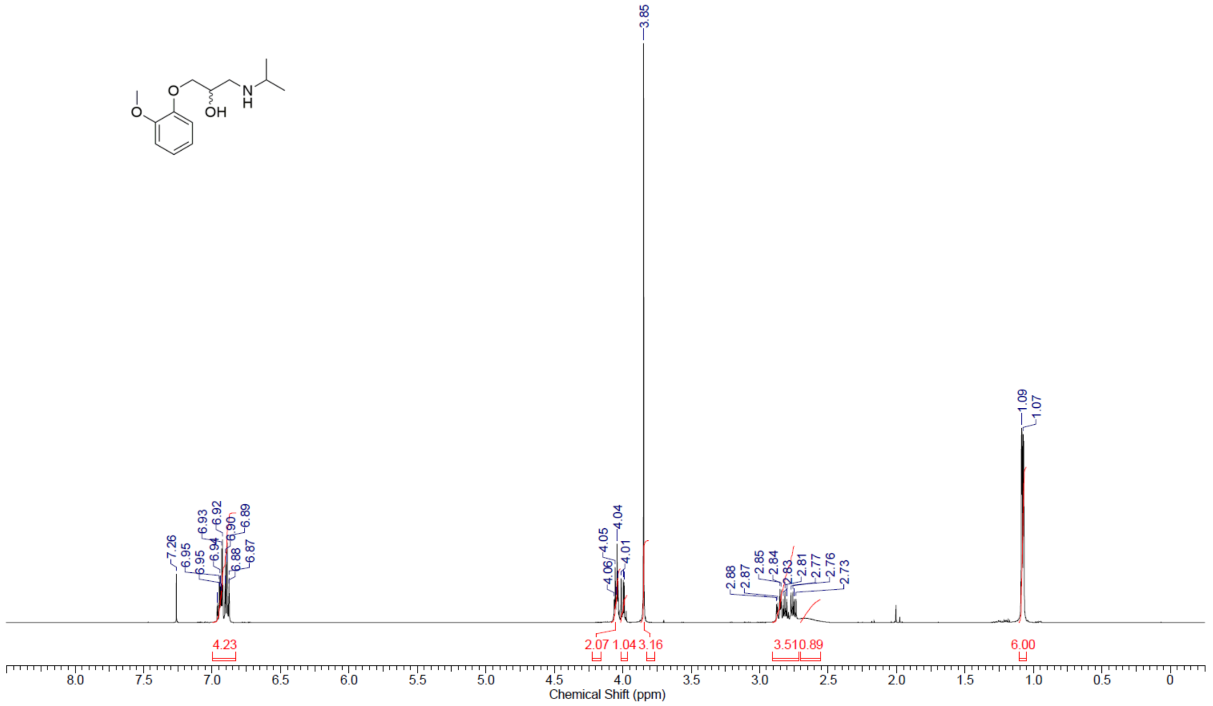

^{13}C NMR spectrum of *rac*-**8e** (126 MHz, $CDCl_3$)

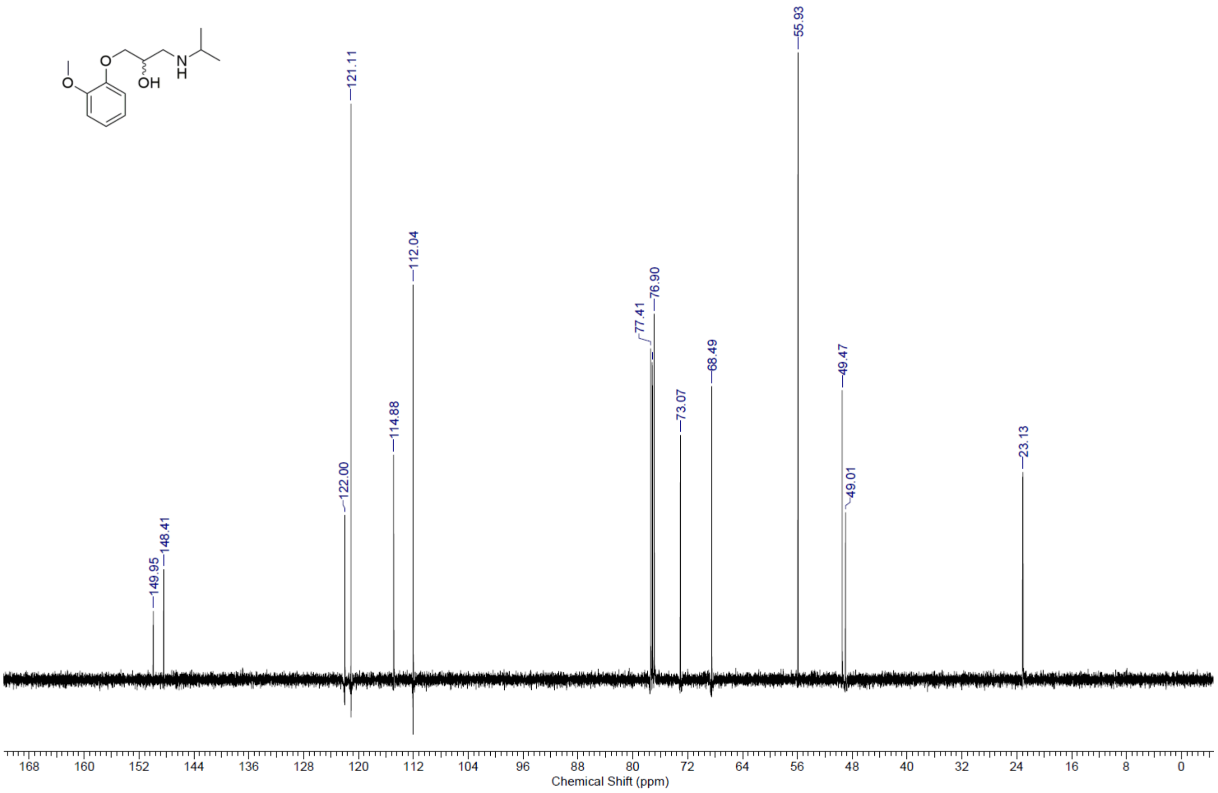

FTMS spectrum of *rac*-**8e** (ESI-TOF)

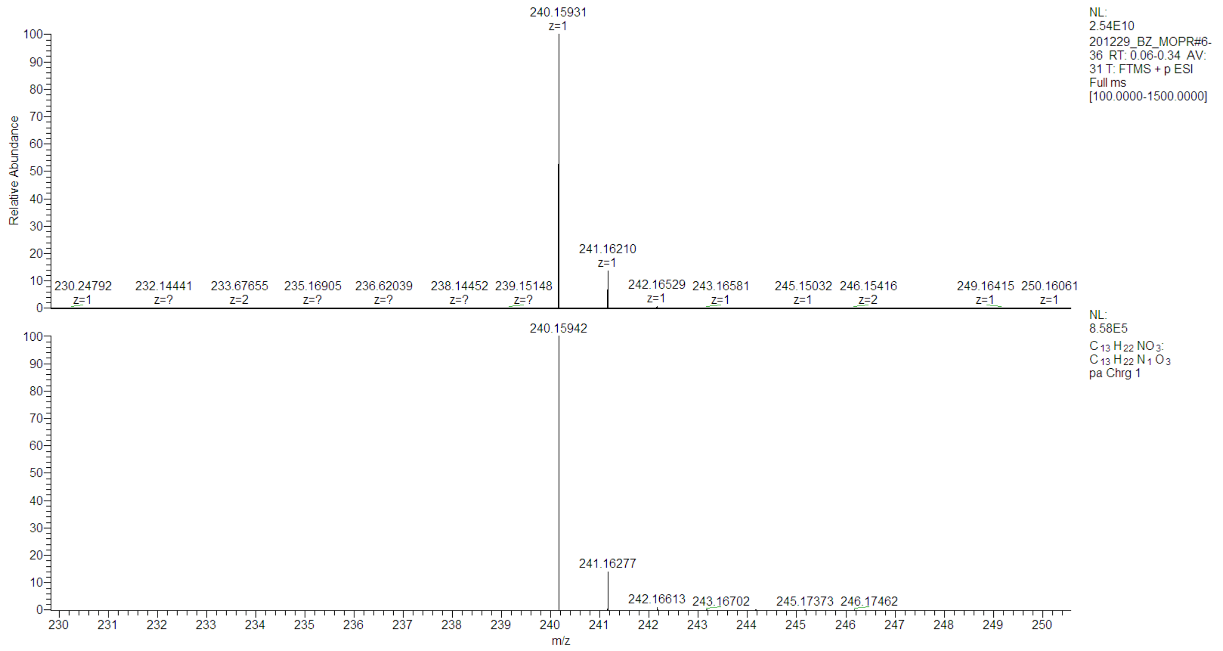

IR spectrum of *rac*-**8e** (Mineral oil, Nujol)

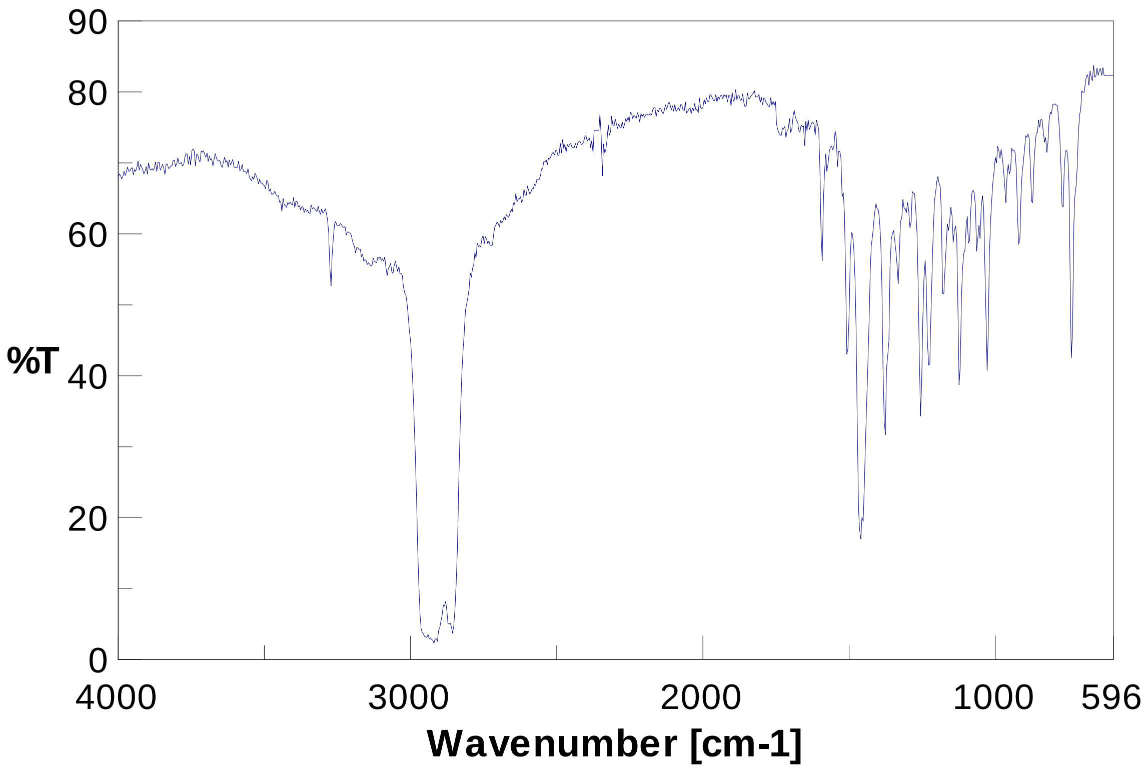

1-[4-(2-Methoxyethyl)phenoxy]-3-(propan-2-ylamino)propan-2-ol (Metoprolol, rac-8f)

^{1}H NMR spectrum of *rac*-**8f** (500 MHz, $CDCl_3$)

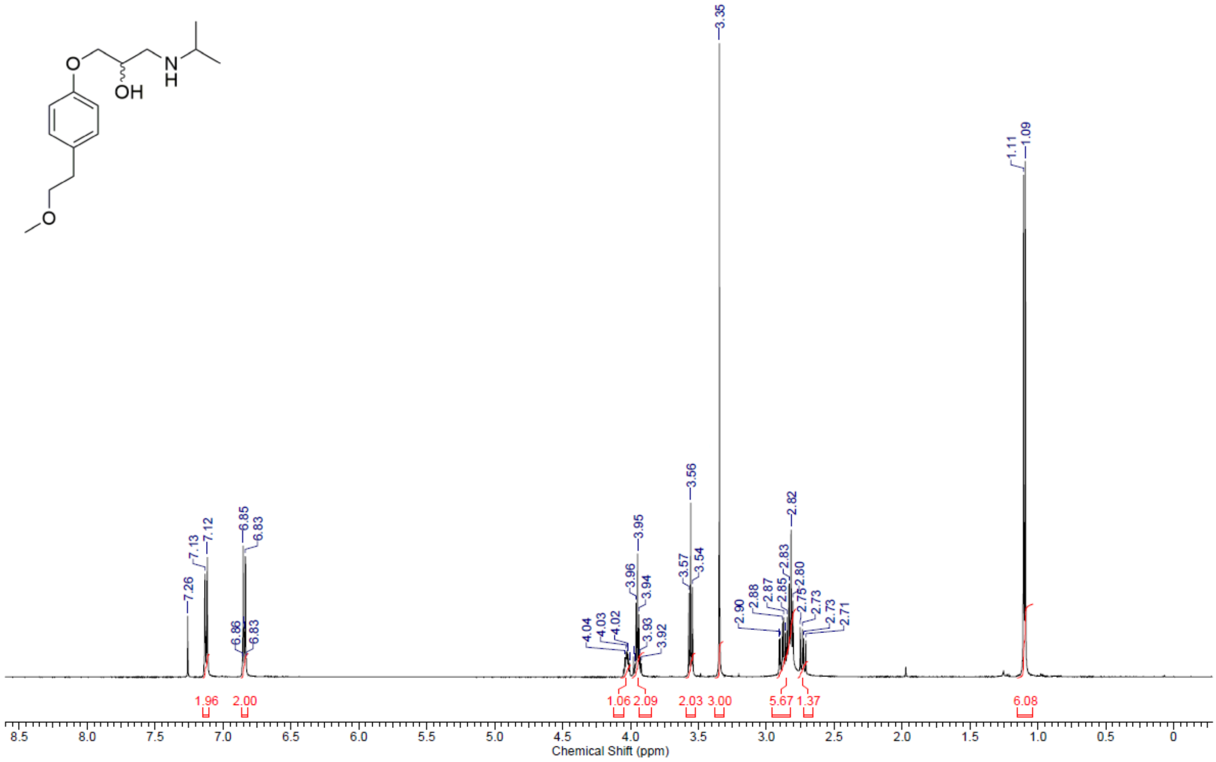

^{13}C NMR spectrum of *rac*-**8f** (126 MHz, $CDCl_3$)

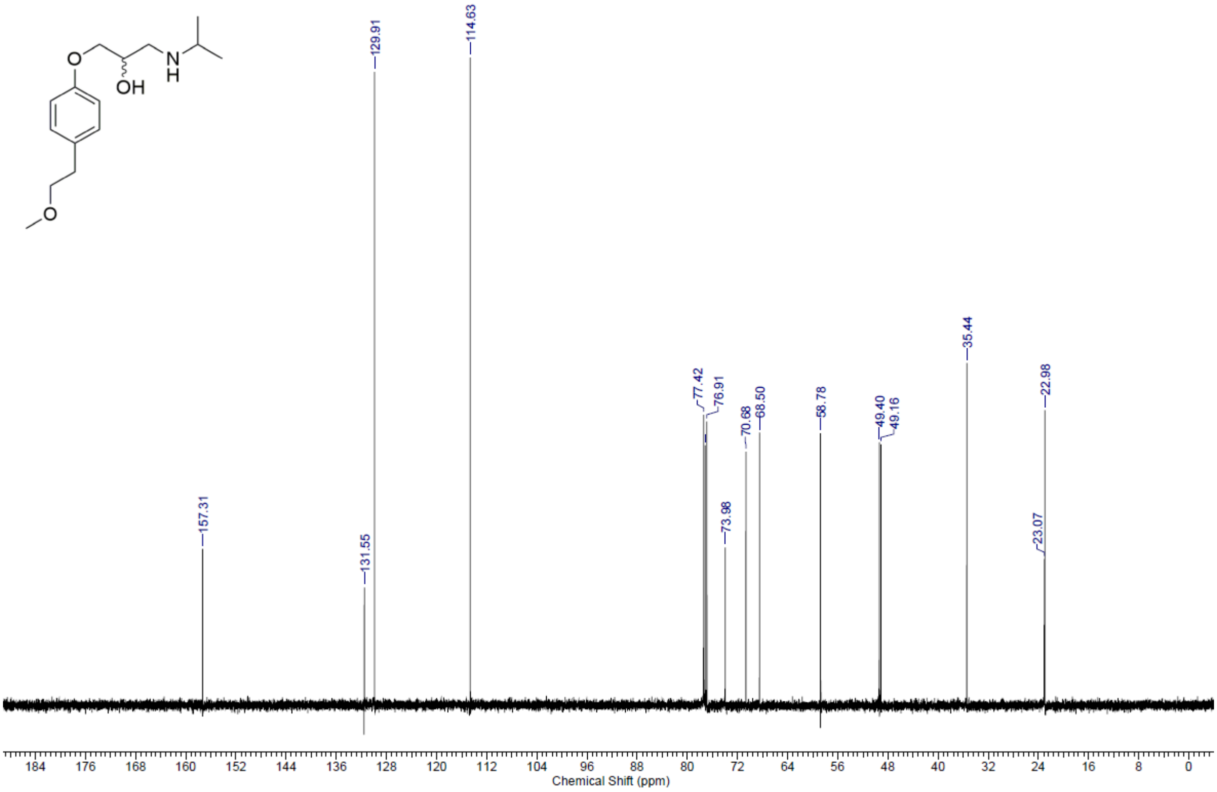

FTMS spectrum of *rac*-**8f** (ESI-TOF)

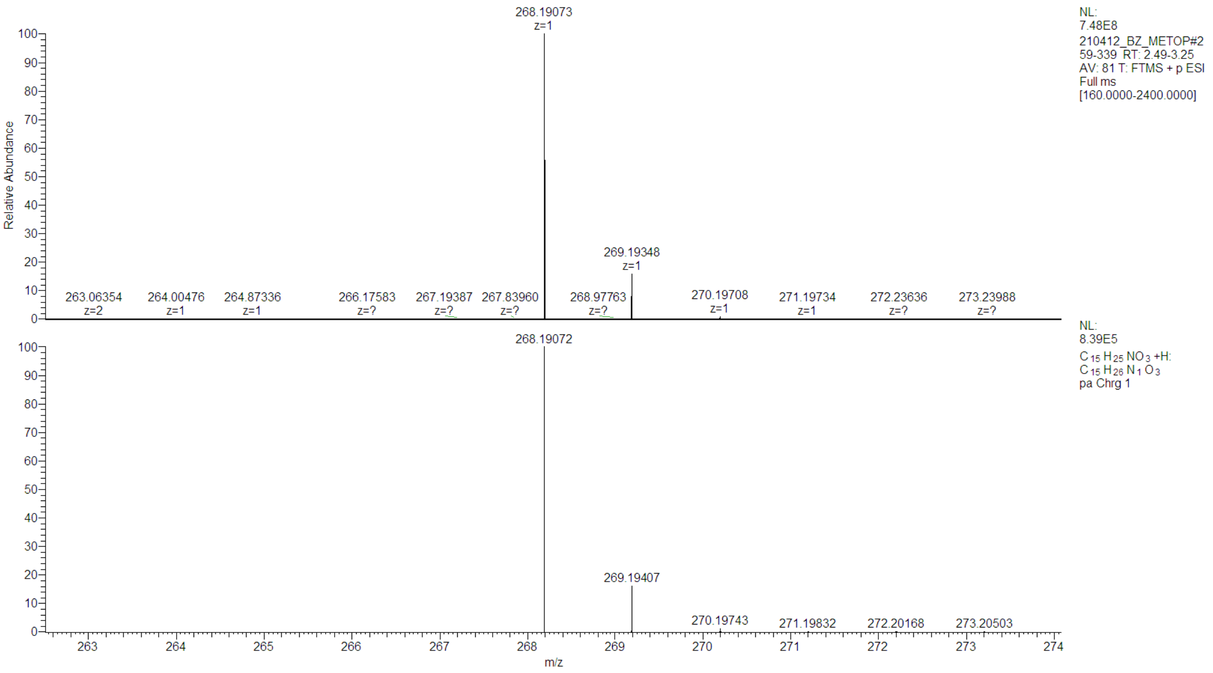

IR spectrum of *rac*-**8f** (Mineral oil, Nujol)

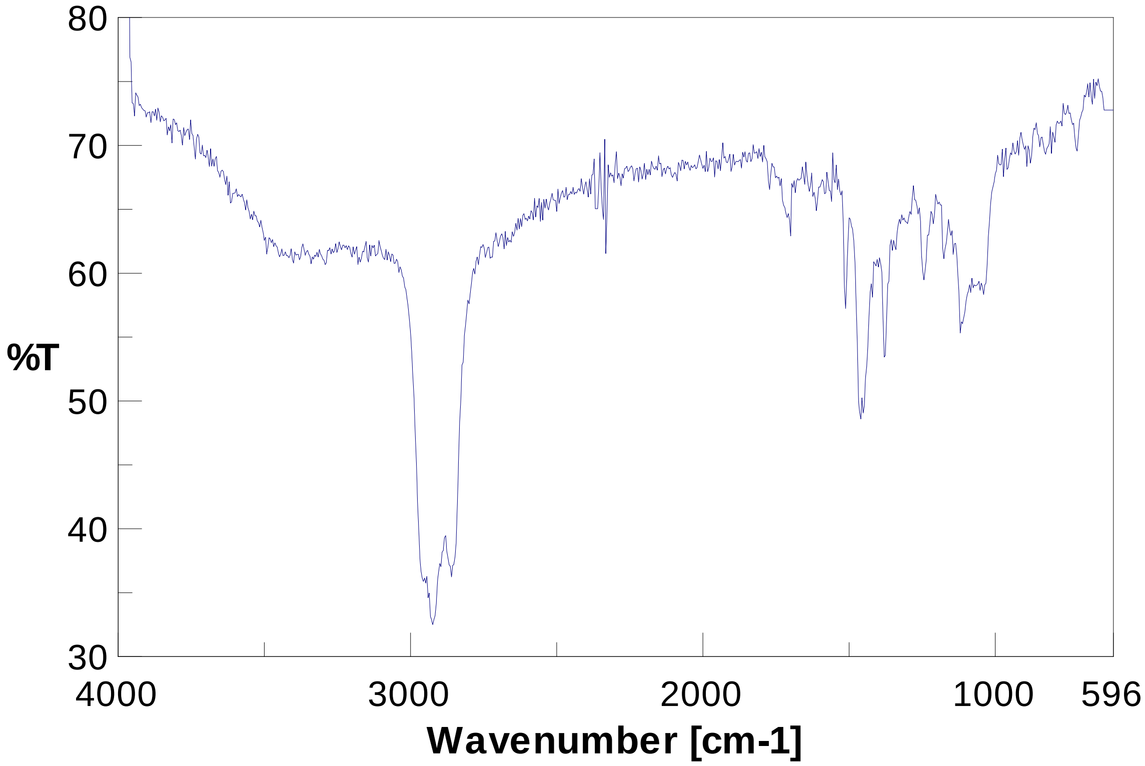

1-Chloro-3-(1,3-dioxo-1,3-dihydro-2H-isoindol-2-yl)propan-2-yl methanesulfonate (rac-9)

^{1}H NMR spectrum of *rac*-**9** (500 MHz, $CDCl_3$)

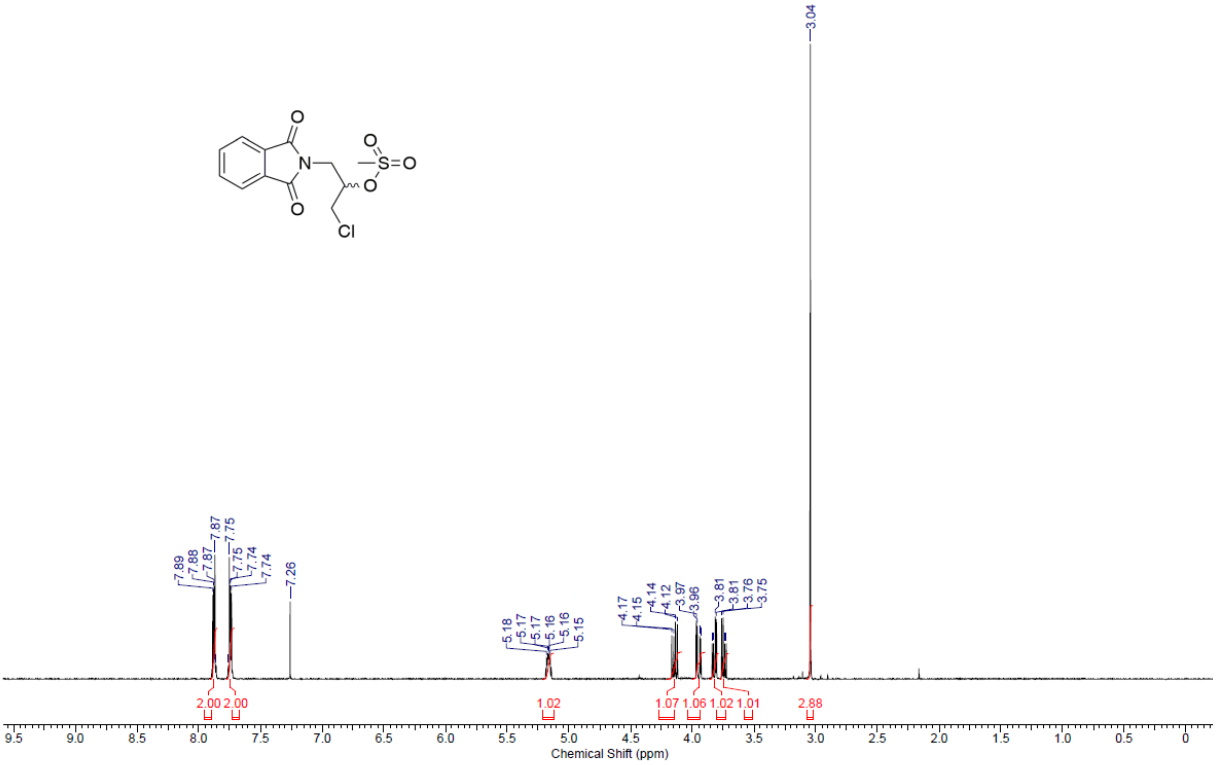

^{13}C NMR spectrum of *rac*-**9** (126 MHz, $CDCl_3$)

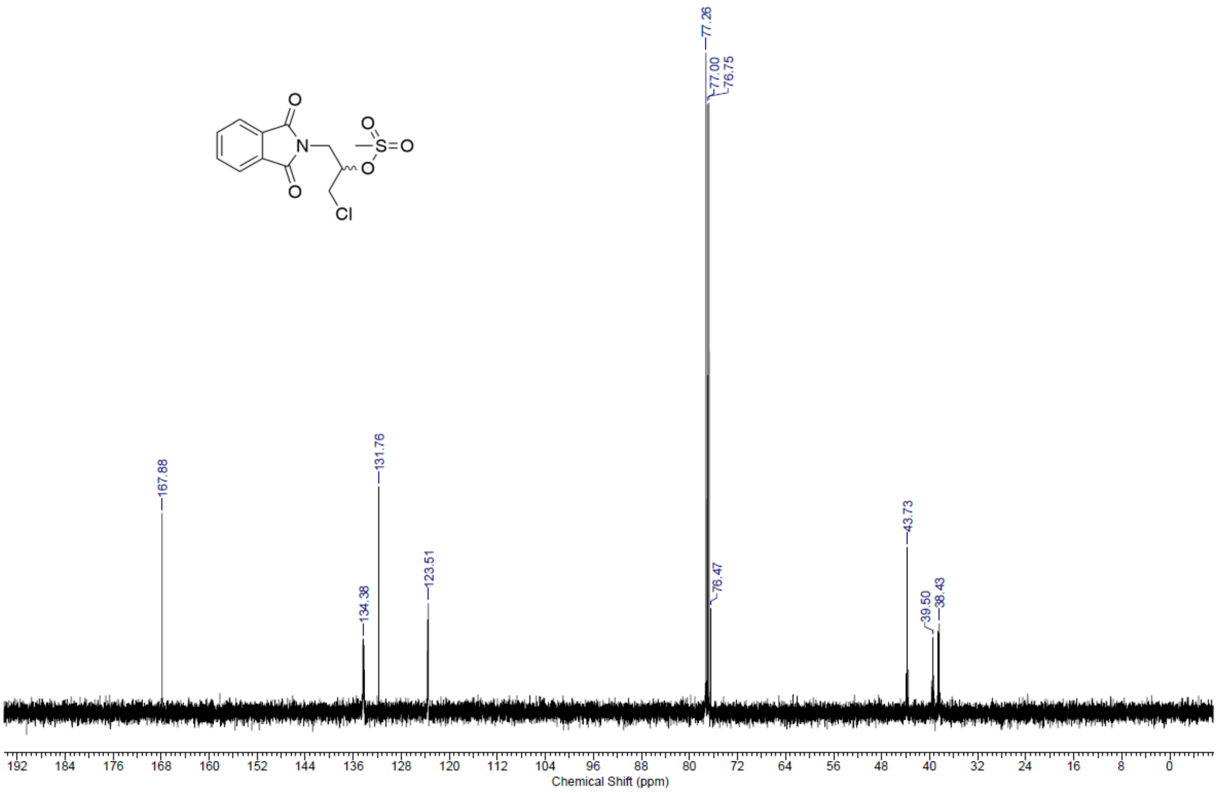

HRMS spectrum of *rac*-**9** (ESI-TOF)

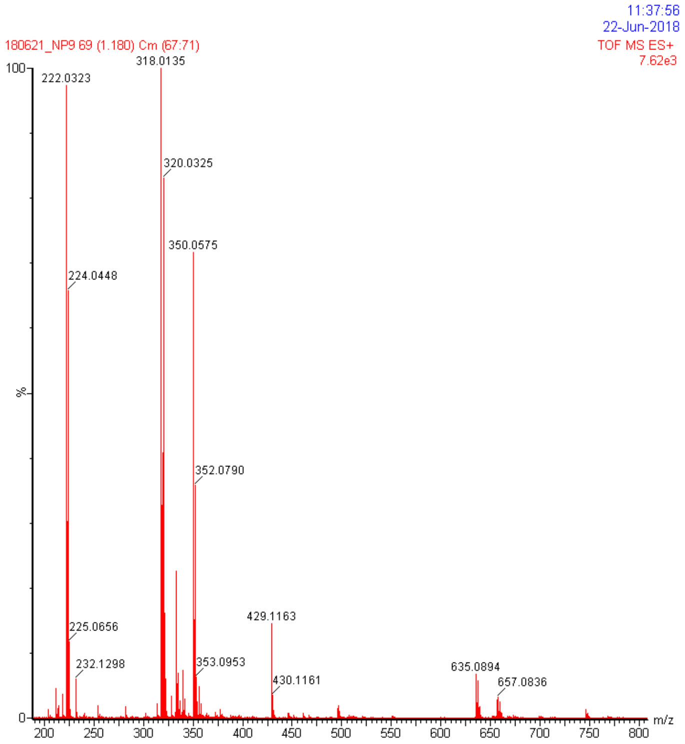

FTMS spectrum of *rac*-**9** (ESI-TOF)

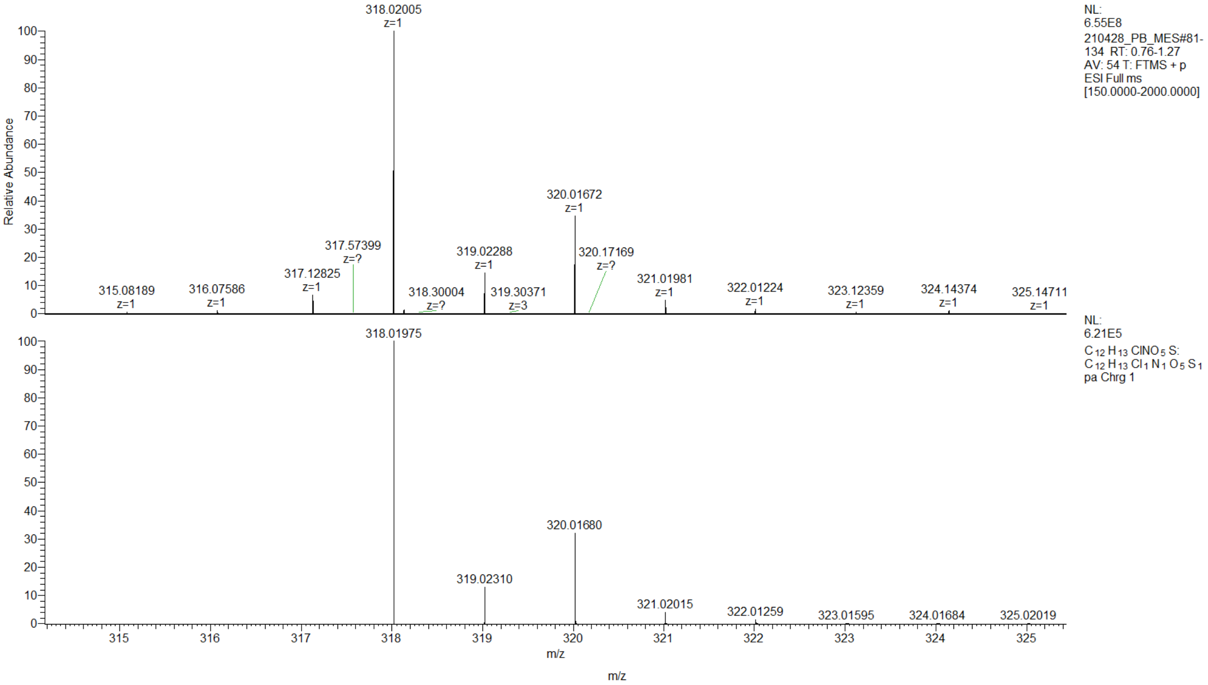

IR spectrum of *rac*-**9** (Mineral oil, Nujol)

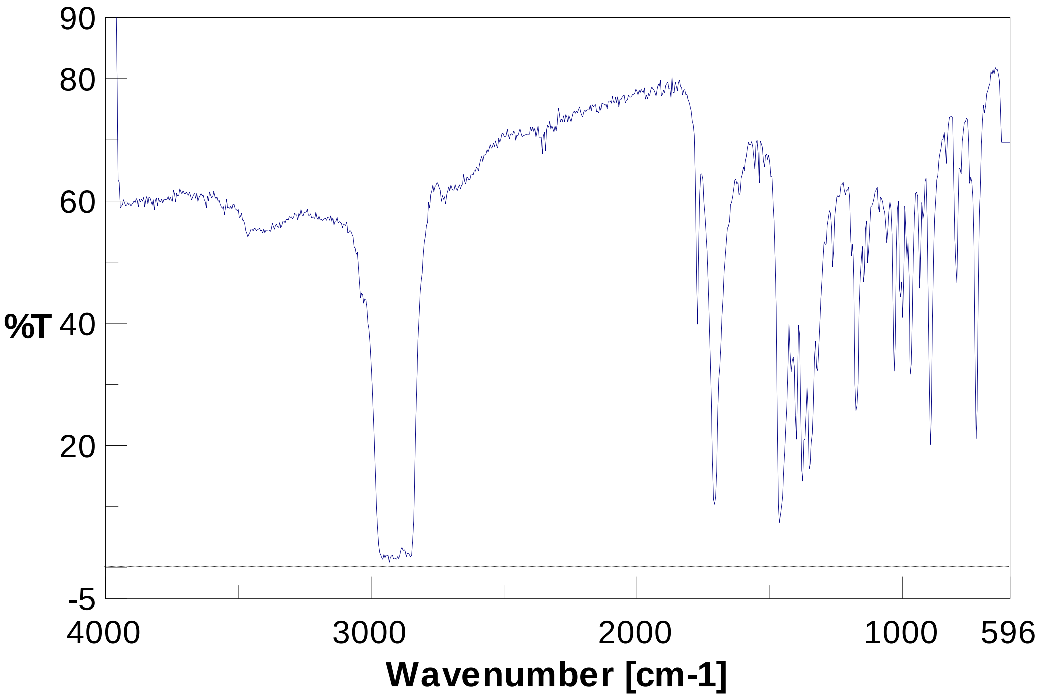

UV-VIS spectrum of *rac*-**9** (EtOH)

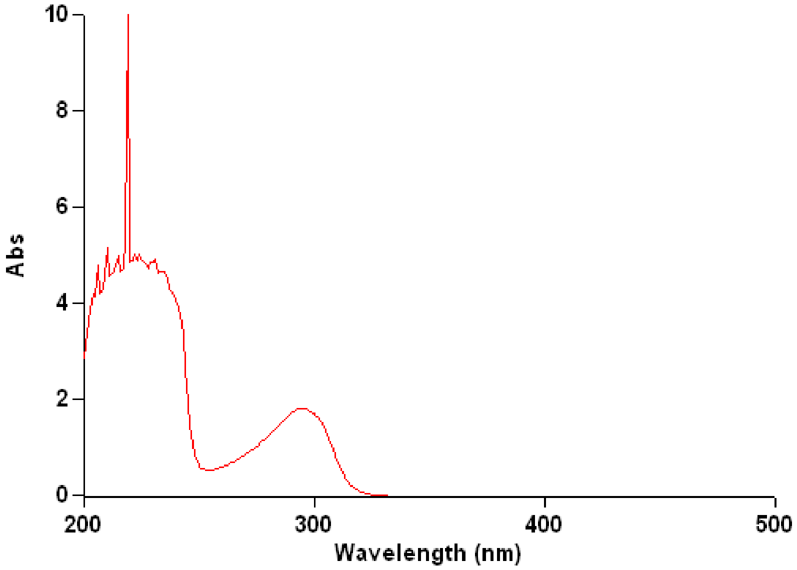

Wavelength (nm)	Abs
294.00	1.802
235.00	4.665
233.00	4.661
231.00	4.918
224.00	5.022
222.00	5.004
219.00	10.000
215.00	4.966
210.00	5.129
206.00	4.786
204.00	4.194

1-Chloro-3-(1,3-dioxo-1,3-dihydro-2H-isoindol-2-yl)propan-2-yl 4-nitrobenzoate (rac-10)

^{1}H NMR spectrum of *rac*-**10** (500 MHz, $CDCl_3$)

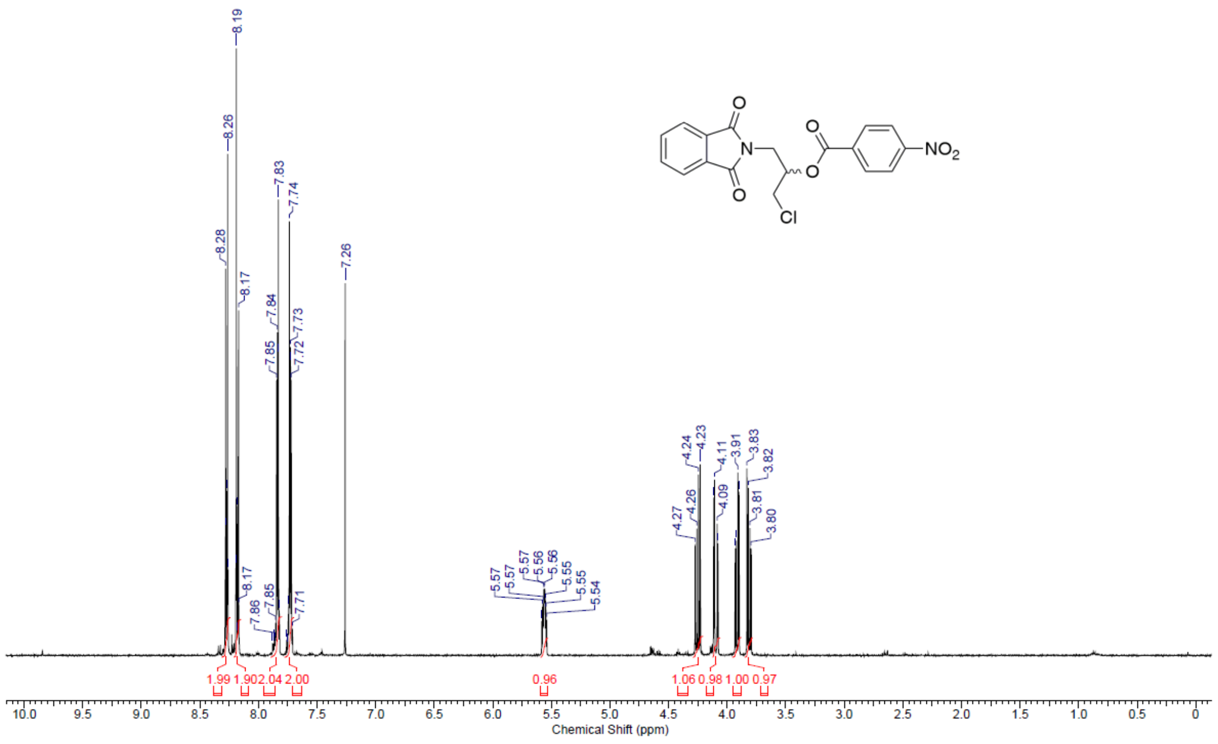

^{13}C NMR spectrum of *rac*-**10** (126 MHz, $CDCl_3$)

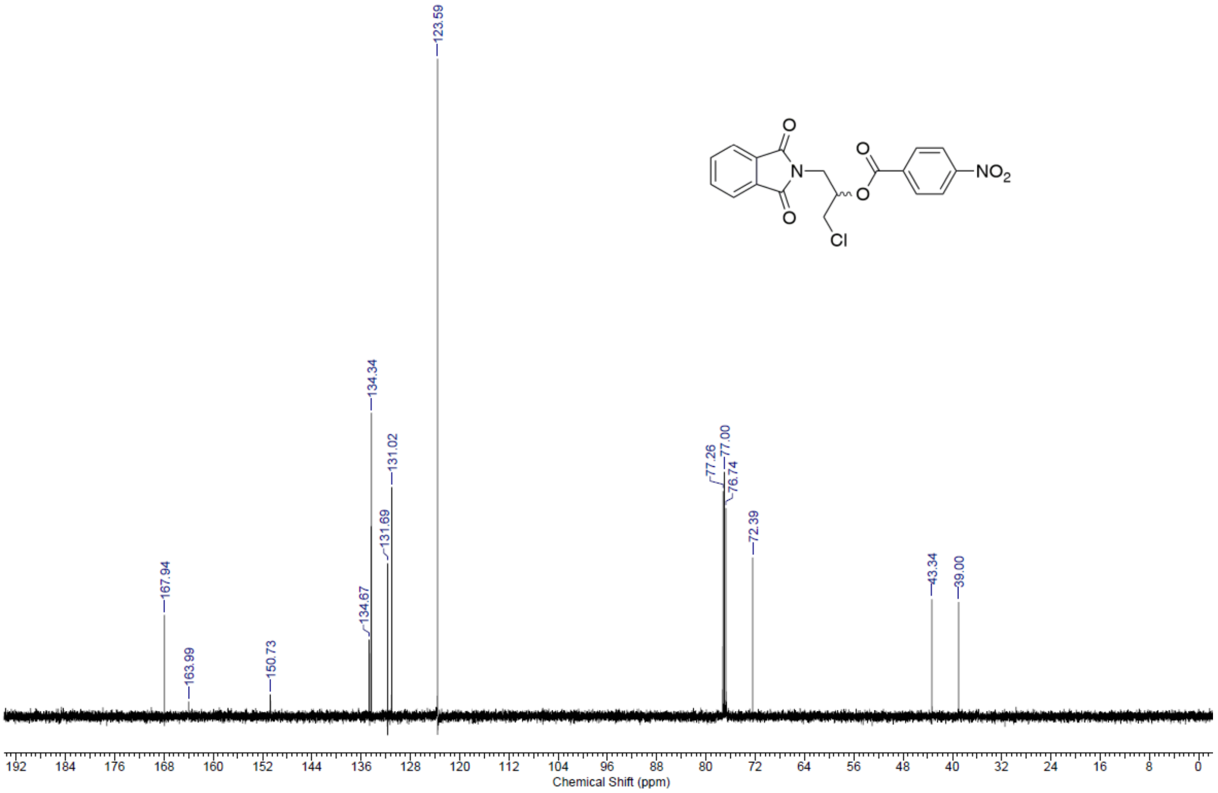

FTMS spectrum of *rac*-**10** (ESI-TOF)

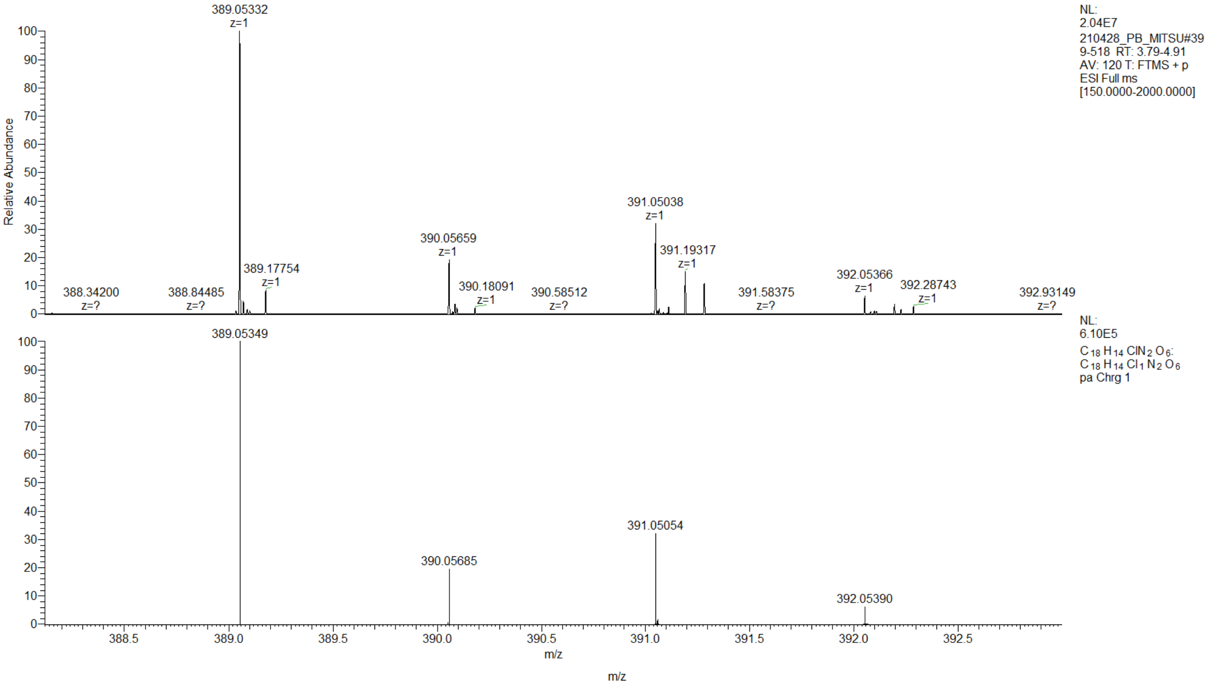

IR spectrum of *rac*-**10** (Mineral oil, Nujol)

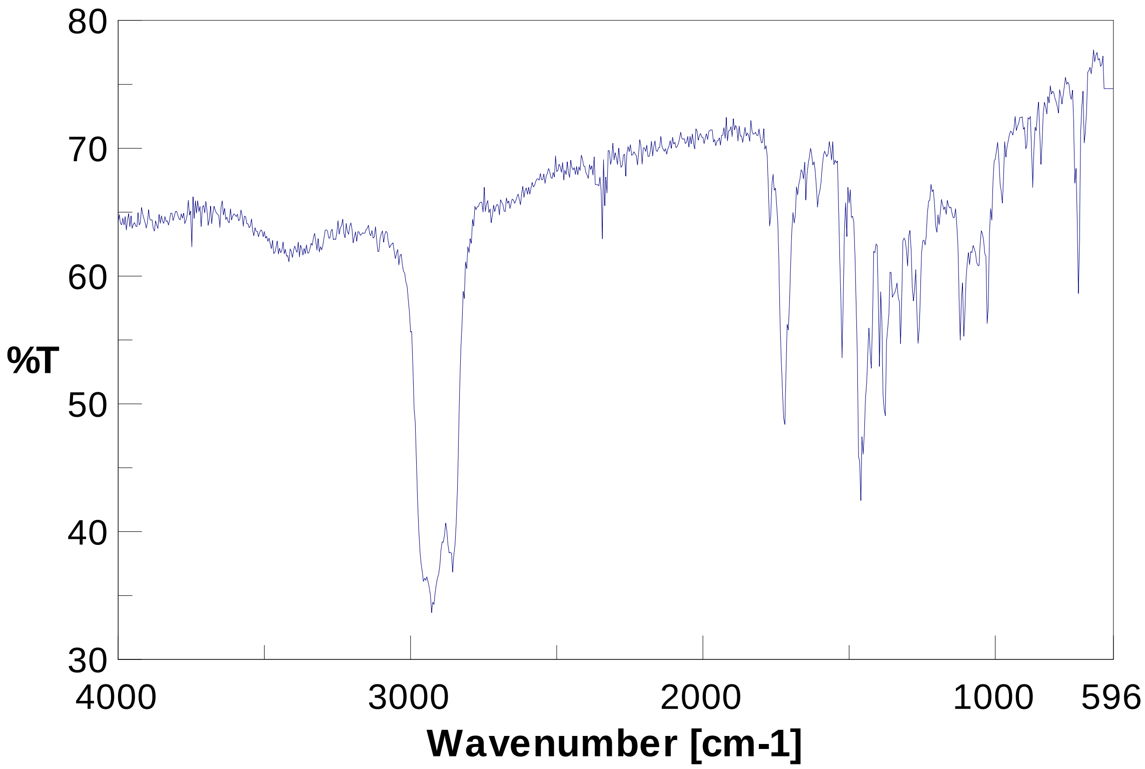

UV-VIS spectrum of *rac*-**10** (EtOH)

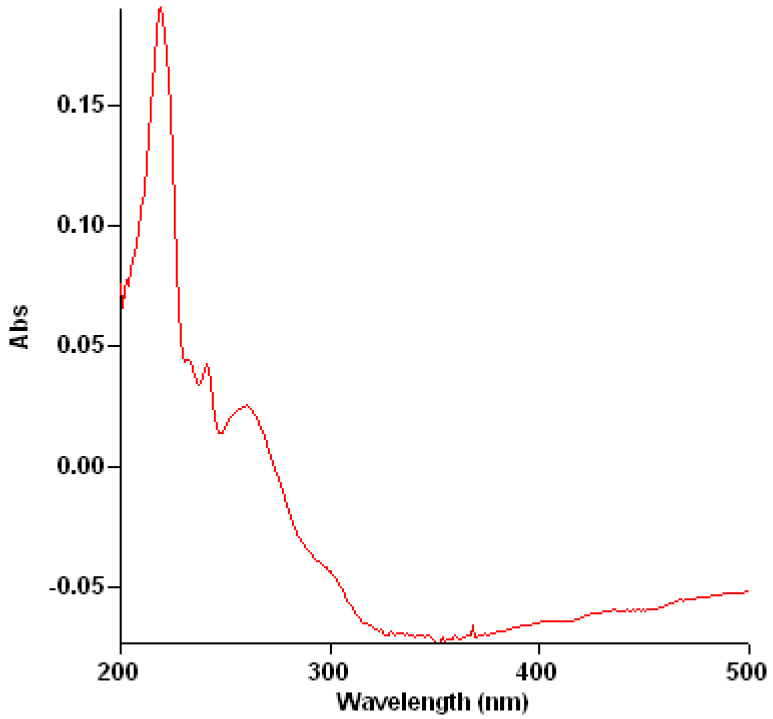

Wavelength (nm)	Abs
260.00	0.025
219.00	0.191

1-Chloro-3-(1,3-dioxo-1,3-dihydro-2H-isoindol-2-yl)propan-2-yl 2,4-dinitrobenzoate (rac-11)

^{1}H NMR spectrum of *rac*-**11** (500 MHz, $CDCl_3$)

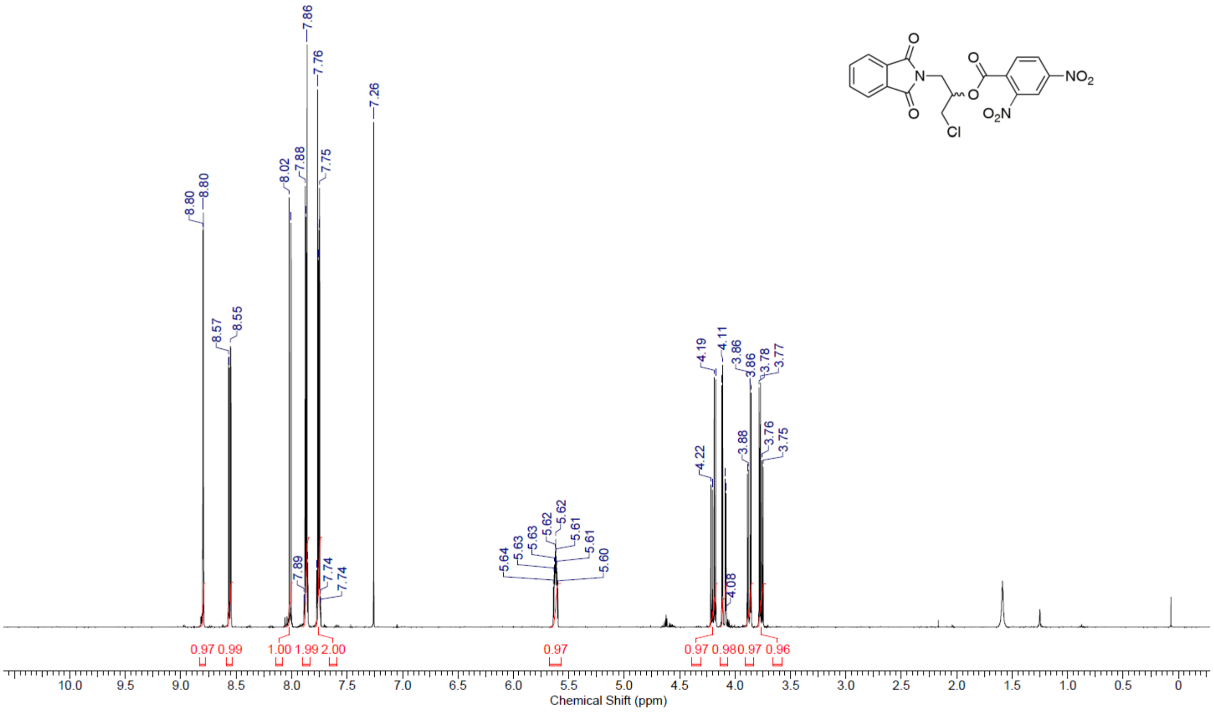

^{13}C NMR spectrum of *rac*-**11** (126 MHz, $CDCl_3$)

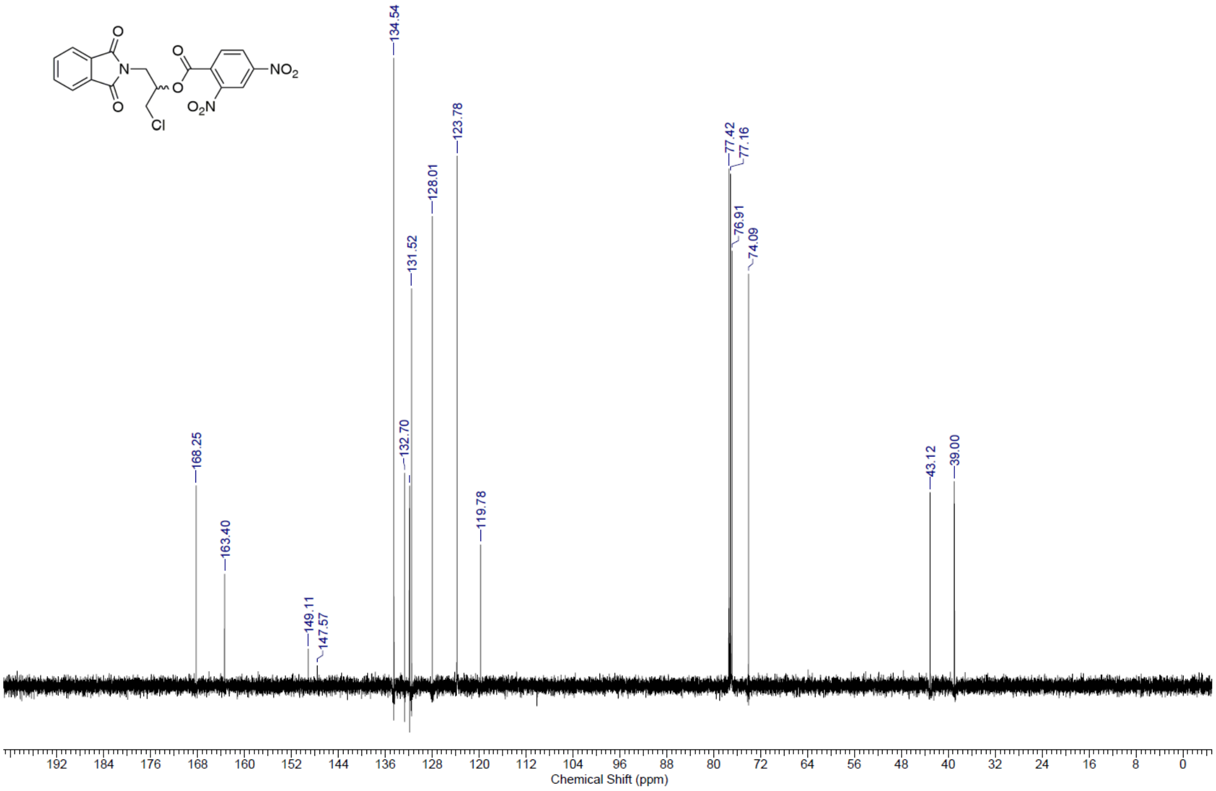

FTMS spectrum of *rac*-**11** (ESI-TOF)

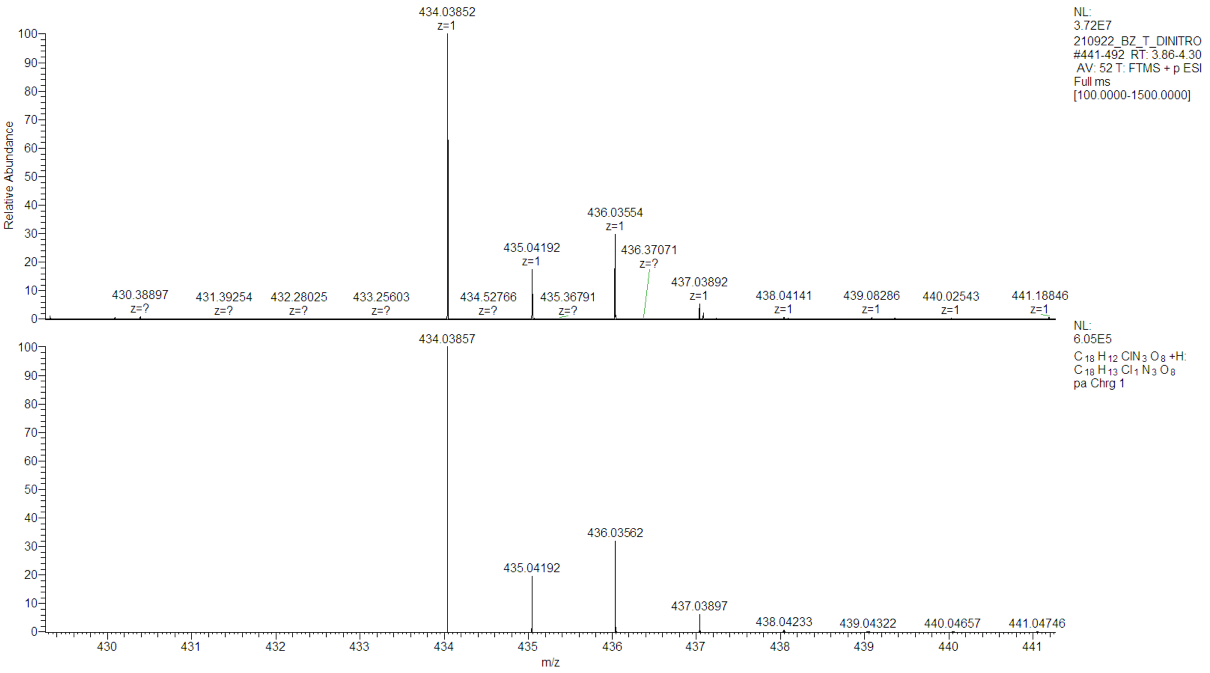

IR spectrum of *rac*-**11** (Mineral oil, Nujol)

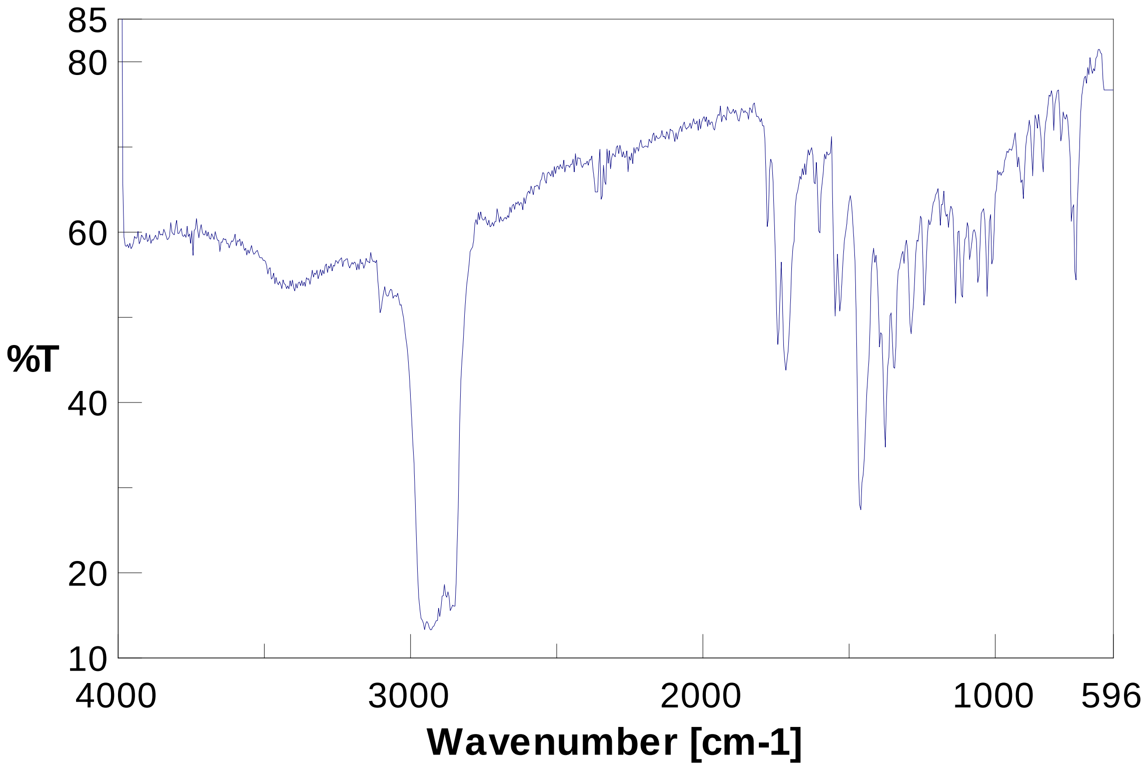

2-(3-Chloro-2-oxopropyl)-1H-isoindole-1,3(2H)-dione (12)

^{1}H NMR spectrum of **12** (500 MHz, $CDCl_3$)

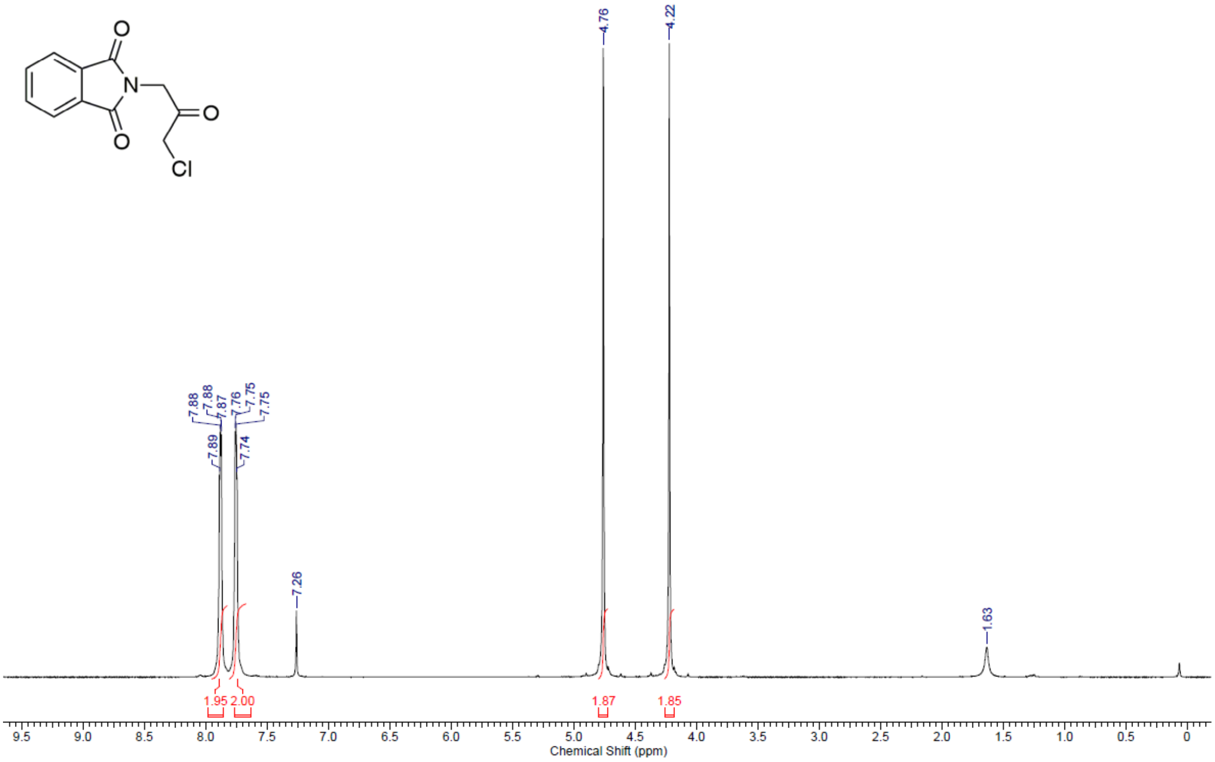

^{13}C NMR spectrum of **12** (126 MHz, $CDCl_3$)

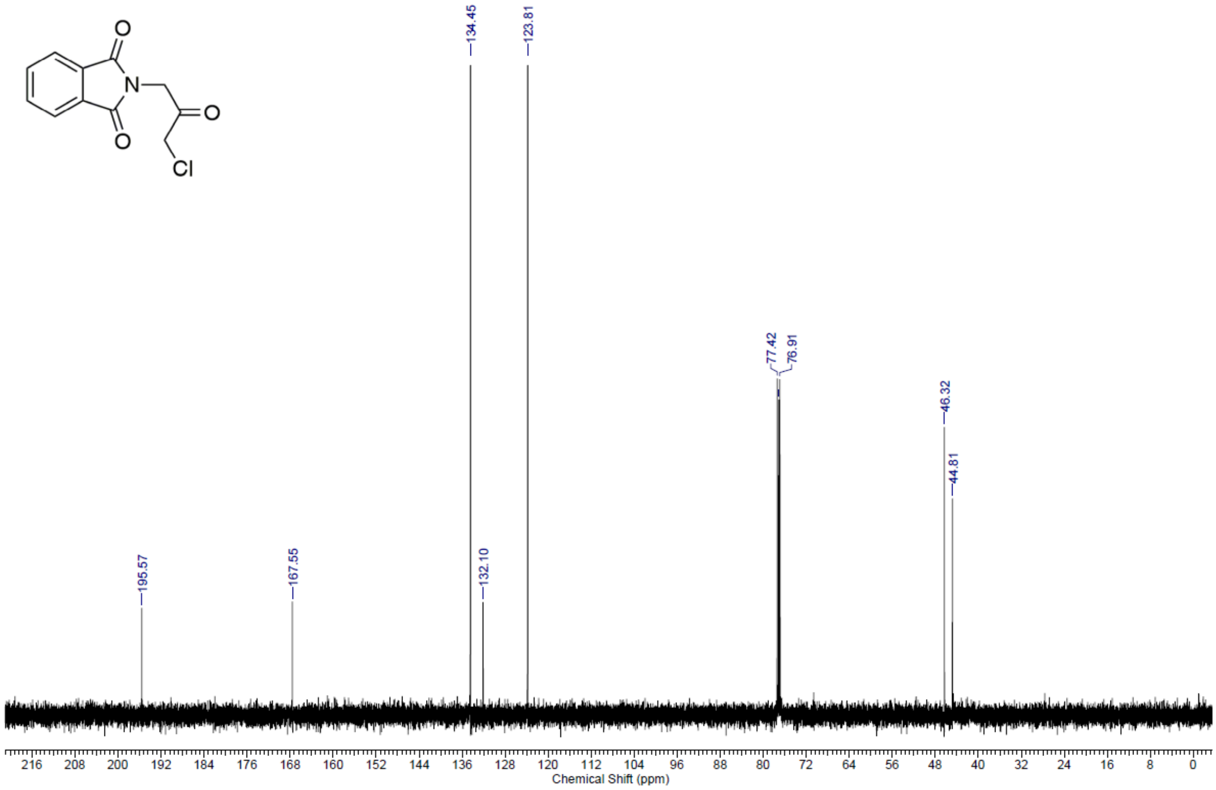

FTMS spectrum of **12** (ESI-TOF)

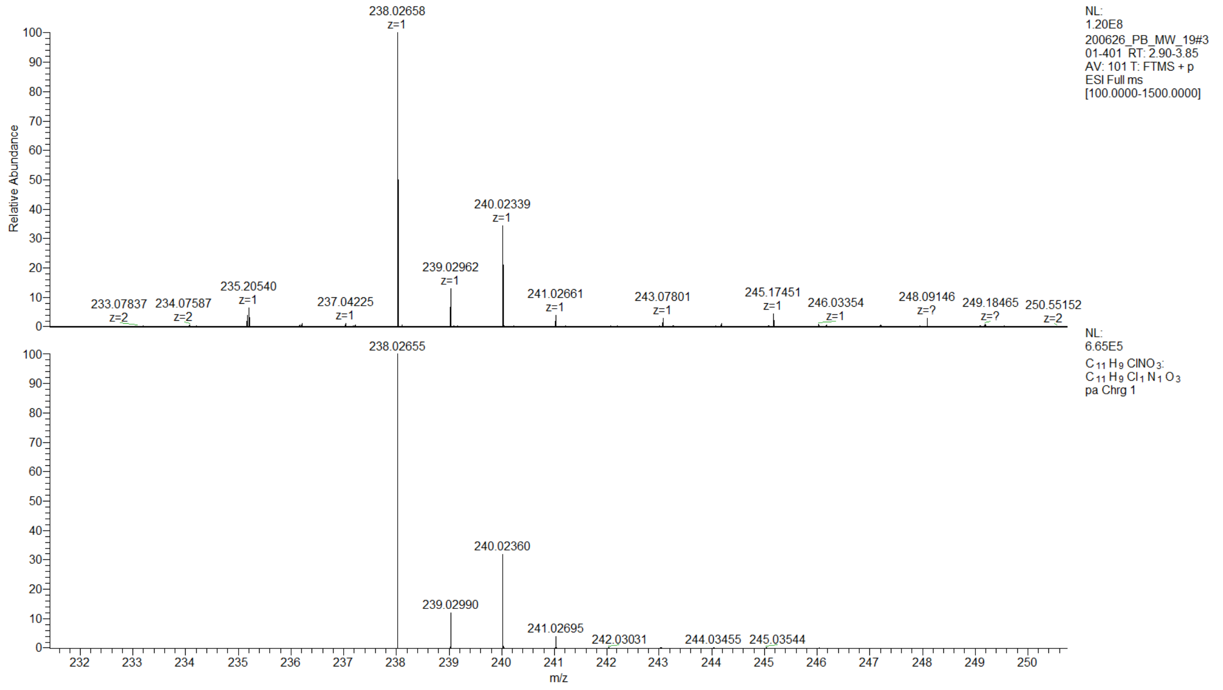

IR spectrum of **12** (Mineral oil, Nujol)

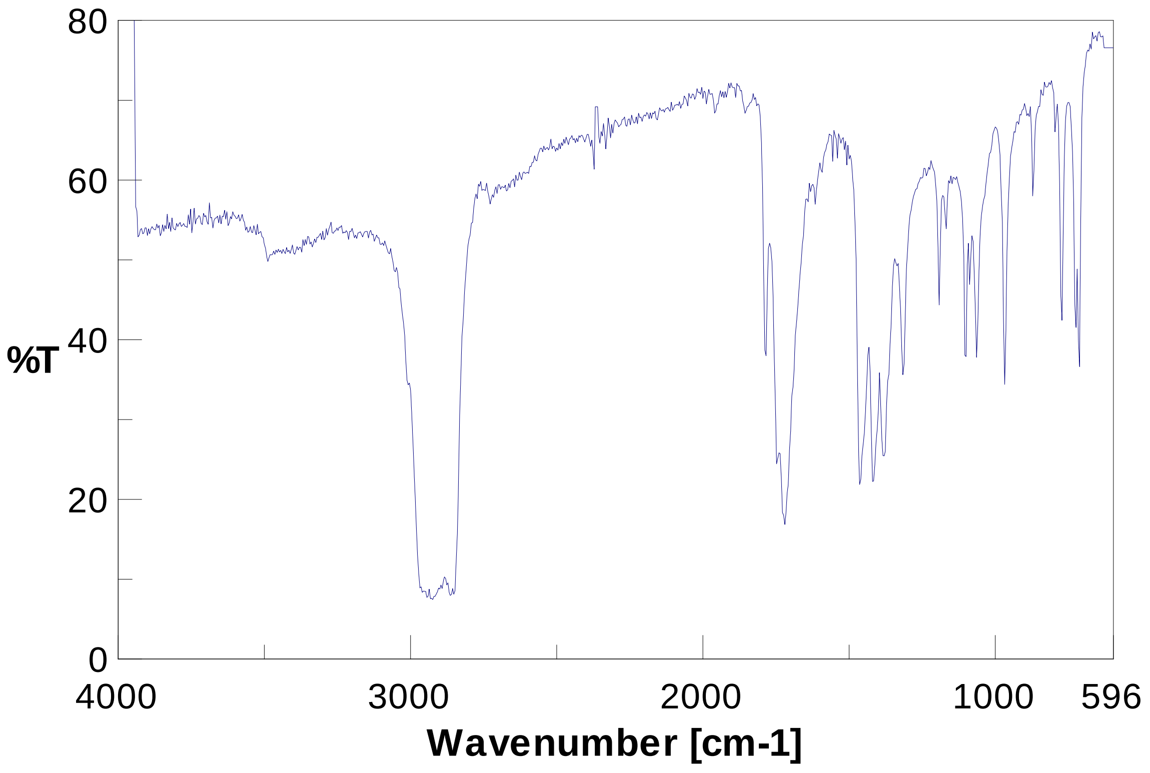

2-{3-chloro-2-[(trimethylsilyl)oxy]propyl}-2,3-dihydro-1H-isoindole-1,3-dione (rac-12)

^{1}H NMR spectrum of *rac*-**13** (500 MHz, $CDCl_3$)

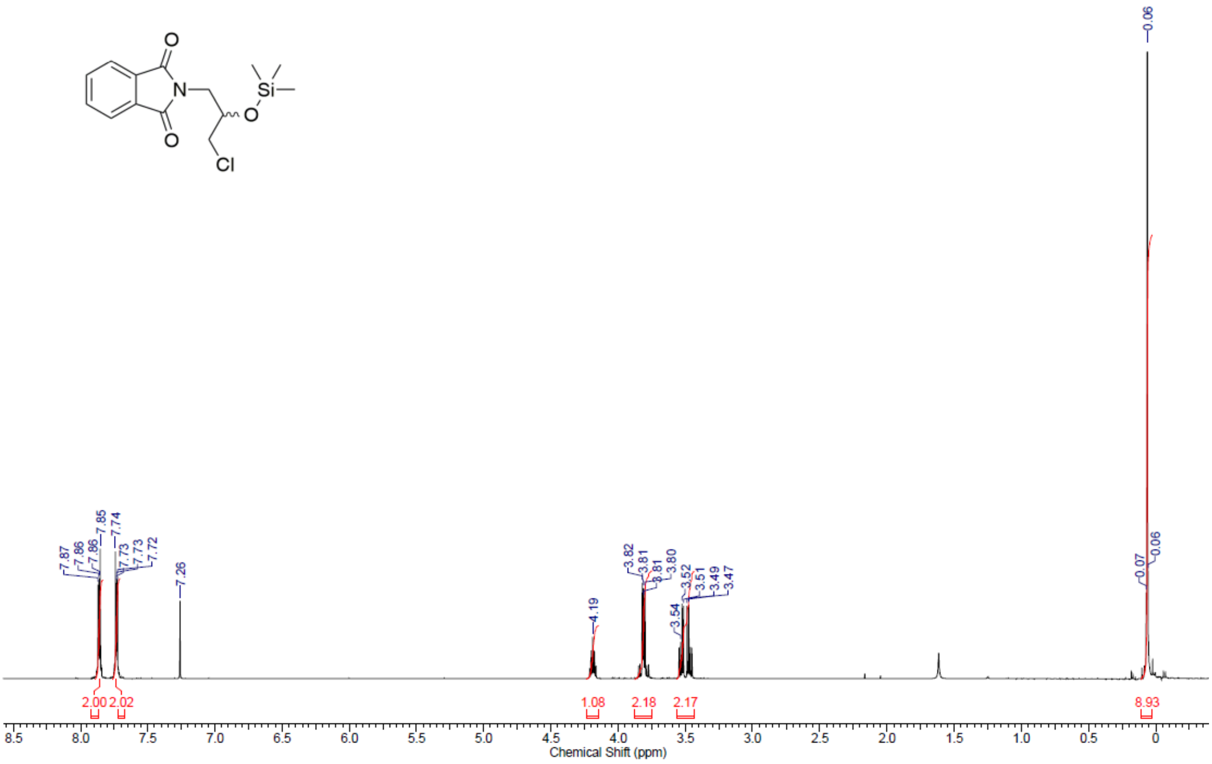

^{13}C NMR spectrum of *rac*-**13** (126 MHz, $CDCl_3$)

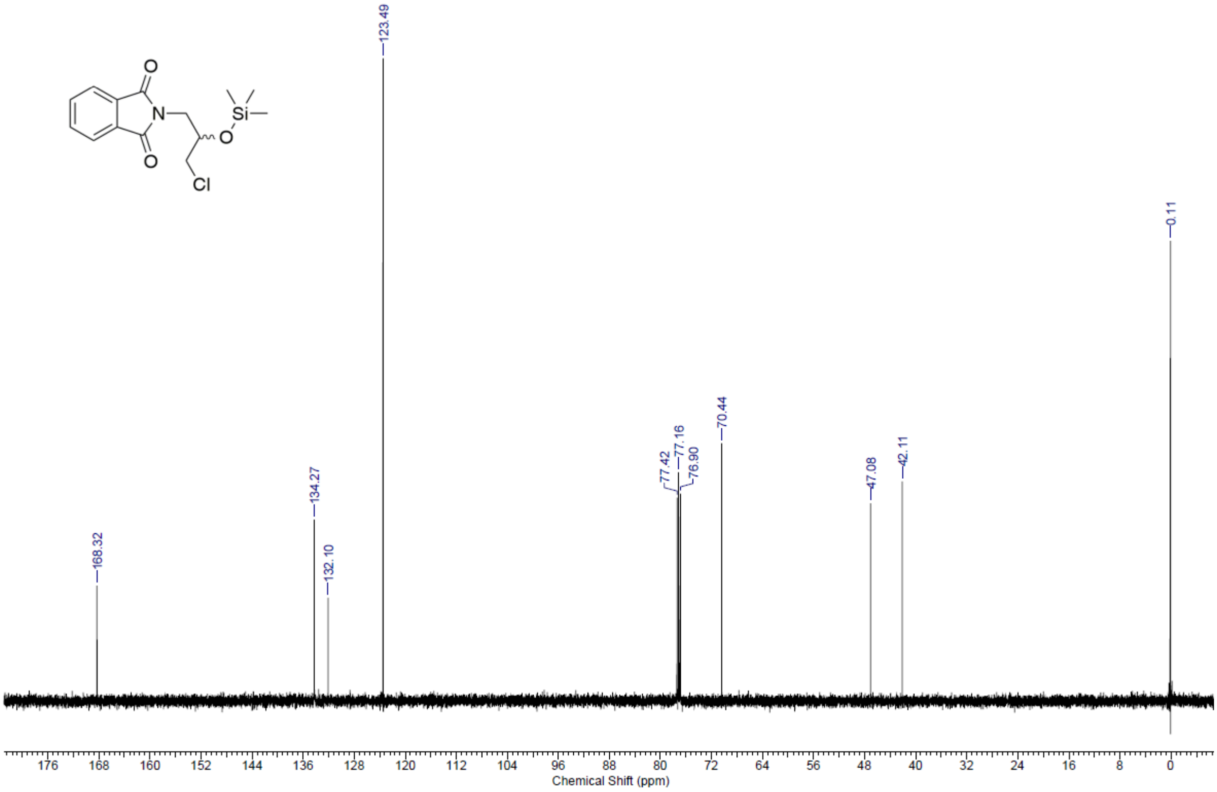

FTMS spectrum of *rac*-**13** (ESI-TOF)

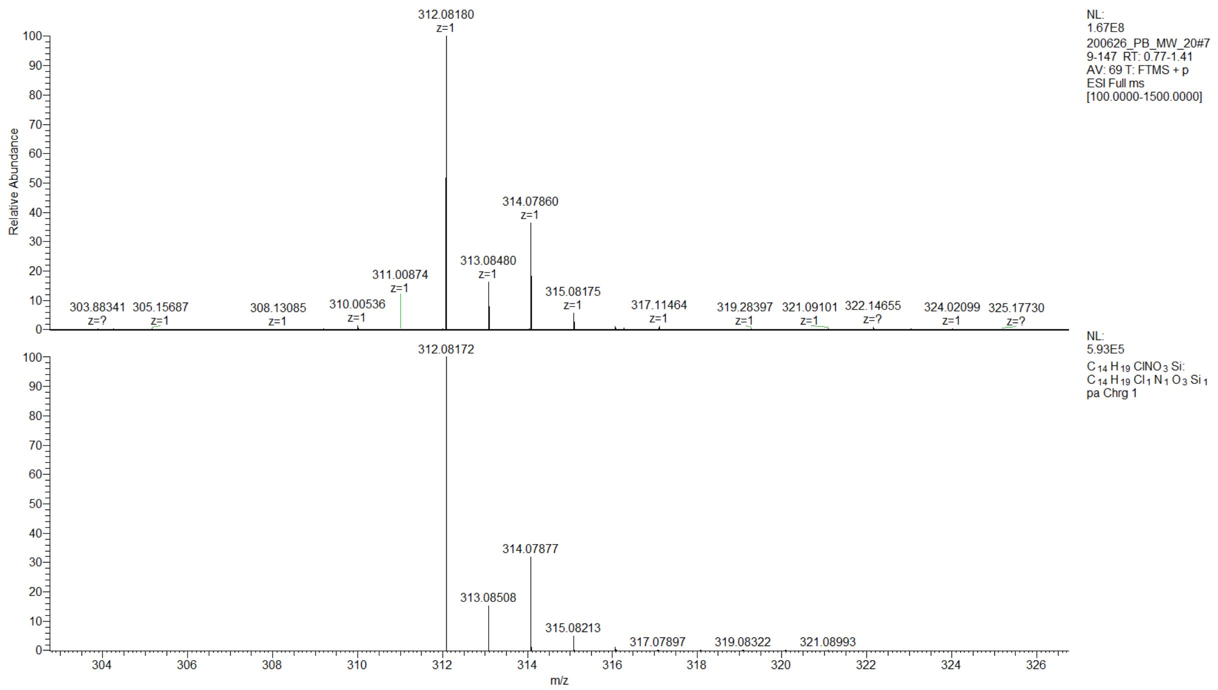

IR spectrum of *rac*-**13** (Mineral oil, Nujol)

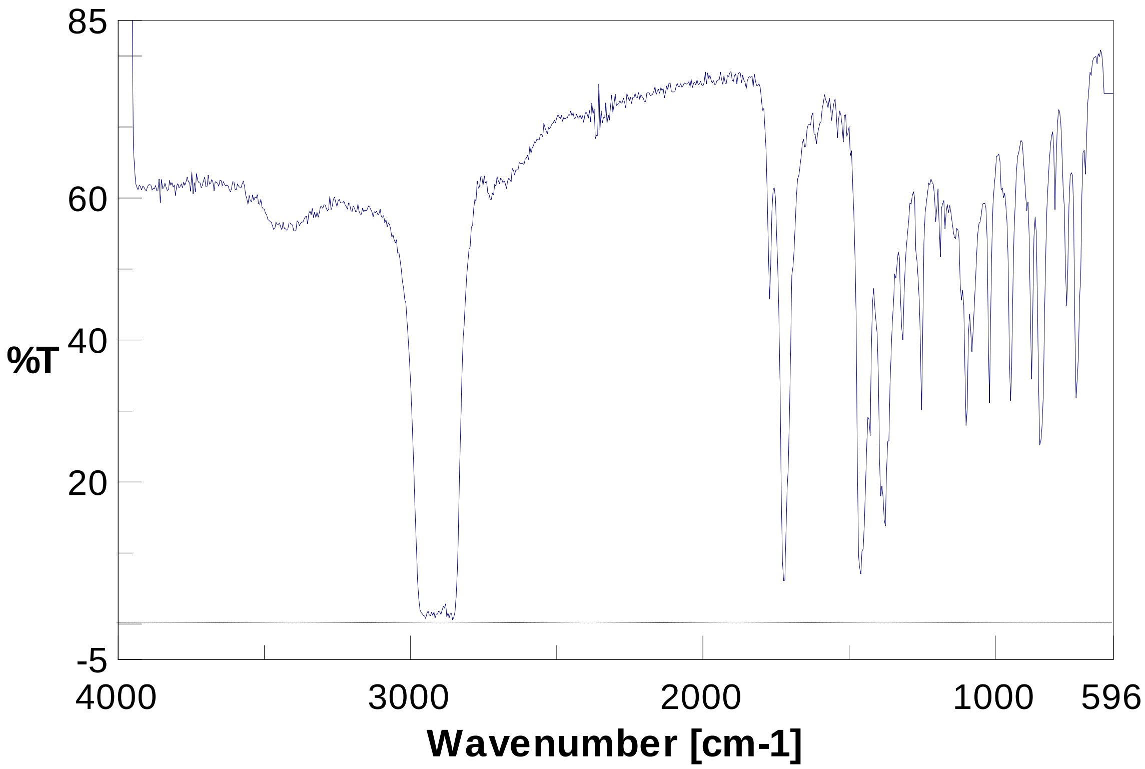

References

1 I. Lavandera, A. Kern, B. Ferreira-Silva, A. Glieder, S. de Wildeman, W. Kroutil, *J. Org. Chem.*, 2008, **73**, 6003–6005.
2 I. Lavandera, A. Kern, V. Resch, B. Ferreira-Silva, A. Glieder, W. M. Fabian, S. de Wildeman, W. Kroutil, *Org. Lett.*, 2008, **10**, 2155–2158.
3 K. Edegger, C. C. Gruber, T. M. Poessl, S. R. Wallner, I. Lavandera, K. Faber, F. Niehaus, J. Eck, R. Oehrlein, A. Hafner, W. Kroutil, *Chem. Commun.*, 2006, 2402–2404.
4 (a) K. Niefind, J. Müller, B. Riebel, W. Hummel, D. Schomburg, *J. Mol. Biol.*, 2003, **327**, 317–328; (b) K. Tauber, M. Fuchs, J. H. Sattler, J. Pitzer, D. Pressnitz, D. Koszelewski, K. Faber, J. Pfeffer, T. Haas, W. Kroutil, *Chem. Eur. J.*, 2013, **19**, 4030–4035.
5 P. Borowiecki, N. Telatycka, M. Tataruch, A. Żądło-Dobrowolska, T. Reiter, K. Schühle, J. Heider, M. Szaleniec, W. Kroutil, *Adv. Synth. Catal.*, 2020, **362**, 2012–2029.
6 (a) A. Weckbecker, W. Hummel, *Biocatal. Biotrans.*, 2009, **24**, 380–389; (b) C. V. Voss, C. C. Gruber, W. Kroutil, *Angew. Chem. Int. Ed. Engl.*, 2008, **47**, 741–745; (c) C. V. Voss, C. C. Gruber, K. Faber, T. Knaus, P. Macheroux, Kroutil, W. *J. Am. Chem. Soc.*, 2008, **130**, 13969–13972.
7 (a) M. A. Emmanuel, N. R. Greenberg, D. G. Oblinsky, T. K. Hyster, *Nature*, 2016, **540**, 414–417; (b) A. Gohel, D. Smith, B. Wong, J. Sukumaran, W. L. Yeo, S. J. Collier, *Biocatalytic process for preparing eslicarbazepine and analogs thereof*, Pat. WO2012142302 (2012).
8 A. Z. Halimehjani, S. E. Hooshmand, E. V. Shamiri, *Tetrahedron Lett.*, 2014, **55**, 5454–5457.
9 S. P. Gupta, S. S. Chatterjee, P. C. Jain, N. Anand, *Synthesis*, 1974, **1974**, 660–661.
10 A. Soldevilla, A. G. Griesbeck, *J. Am. Chem. Soc.*, 2006, **128**, 16472–16473.
11 V. Pace, P. Hoyos, M. Fernández, J. V. Sinisterra, A. R. Alcántara, *Green Chem.*, 2010, **12**, 1380–1382.
12 J. A. Heyes, D. Niculescu-Duvaz, R. G. Cooper, C. J. Springer, *J. Med. Chem.*, 2002, **45**, 99–114.
13 H. R. Ing, W. E Ormerod, *J. Pharm. Pharmacol.*, 1952, **4**, 21–26.
14 V. A. Bondarenko, T. K. Trubitsyna, E. E. Mikhlina, M. D. Mashkovskii, Yakhontov, L. N. *Pharm. Chem. J.*, 1980, **14**, 27–31.
15 G. Madhusudhan, B. Kumar, U. S. Anand Chintamani, M. Rao, D. Narasimha Udaykiran, T. Suresh, V. Kumar, K. Kiran Mukkanti, *Indian J. Chem.*, 2010, **49B**, 606–610.
16 C. D. Lunsford, R. P. Mays, J. A. Richman, R. S. Murphey, *J. Am. Chem. Soc.*, 1960, **82**, 1166–1171.
17 W. L. Nelson, J. E. Wennerstrom, S. R. Sankar, *J. Org. Chem.*, 1977, **42**, 1006–1012.
18 J.-Y. Zhang, H.-M. Liu, H.-W. Xu, L.-H. Shan, *Tetrahedron: Asymm.*, 2008, **19**, 512–517.
19 A. M. Kawamoto, M. Wills, *J. Chem. Soc., Perkin Trans. 1*, 2001, 1916–1928.
20 X.-D. Kong, H.-L. Yu, S. Yang, J. Zhou, B.-B. Zeng, J.-H. Xu, *J. Mol. Catal. B: Enz.*, 2015, **122**, 275–281.
21 I. Kobayashi, K. Hosaka, T. Ueno, H. Maruo, M. Kamiyama, C. Konno, M. Gemba, *Biol. Pharm. Bull.*, 1997, **20**, 421–427.
22 Z. A. Bredikhina, D. V. Savel'ev, A. A. Bredikhin, *Rus. J. Org. Chem.*, 2002, **38**, 213–219.
23 (a) J. Canotilho, R. A. E. Castro, M. T. S. Rosado, S. C. C. Nunes, M. S. C. Cruz, J. S. Redinha, *J. Therm. Anal. Calorim.*, 2010, **100**, 423–429; (b) C. A. S. Bergström, U. Norinder, K. Luthman, P. Artursson, *Pharm. Res.* 2002, **19**, 182–188.
24 A. A. Bredikhin, Z. A. Bredikhina, A. V. Kurenkov, D. B. Krivolapov, *Tetrahedron: Asymm.*, 2017, **28**, 442–446.
25 C. Brutting, R. Hesse, A. Jager, O. Kataeva, A. W. Schmidt, H. J. Knolker, *Chem. Eur. J.*, 2016, **22**, 16897–16911.
26 M. Rudolph, H. Steinhart, B. Helpap, *Carb. Res.*, 1988, **176**, 155–159.
27 C. D. Lunsford, R. P. Mays, J. A. Richman, R. S. Murphey, *J. Am. Chem. Soc.*, 1960, **82**, 1166–1171.
28 J. D. White, S. Shaw, *Org. Lett.*, 2012, **14**, 6270–6273.
29 T. Kai, T. Isami, K. Kobata, Y. Kurosaki, T. Nakayama, T. Kimura, *Chem. Pharm. Bull. (Tokyo)*, 1992, **40**, 2498–2504.
30 J. E. Bäckvall, S. E. Bystroem, *J. Org. Chem.*, 2002, **47**, 1126–1128.
31 S. H. Jung, T. L. Pham, H. K. Lim, H. J. Kim, K. H. Kim, J. S. Kang, *Arch. Pharm. Res.*, 2000, **23**, 226–229.
32 S. Cheng, X. Liu, P. Wang, X. Li, W. He, S. Zhang, *Lett. Org. Chem.*, 2012, **9**, 516–519.
33 S. Gabrial, *J. Chem. Soc., Abstr.*, 1917, **112**, 565–566.
34 C. S. Chen, Y. Fujimoto, G. Girdaukas, C. J. Sih, *J. Am. Chem. Soc.* 1982, **104**, 7294–7299.
35 X.-X. Shi, C.-L. Shen, J.-Z. Yao, L.-D. Nie, N. Quan, *Tetrahedron: Asymm.*, 2010, **21**, 277–284.
36 P. Borowiecki, B. Zdun, M. Dranka, *Mol. Catal.*, 2021, **504**, 111451.
